# Solvent Effects on the Photophysical Properties of Unexplored Imidazo[1,2‑*a*]pyridine Derivatives

**DOI:** 10.1021/acsomega.5c07201

**Published:** 2026-03-13

**Authors:** Victor H. Justino Garcia Praciano, Luan A. Martinho, Guilherme Duarte Ramos Matos, Claudia C. Gatto, Carlos Kleber Z. Andrade

**Affiliations:** † Instituto de Química, Laboratório de Química Metodológica e Orgânica Sintética (LaQMOS), 28127 Universidade de Brasília, Campus Universitário Asa Norte, Brasília, DF 70904-970, Brazil; ‡ Instituto de Química, Laboratório de Modelagem de Sistemas Complexos (LMSC), 28127 Universidade de Brasília, Campus Universitário Asa Norte, Brasília, DF 70904-970, Brazil; § Instituto de Química, Laboratório de Síntese Inorgânica e Cristalografia (LASIC), 28127 Universidade de Brasília, Campus Universitário Asa Norte, Brasília, DF 70904-970, Brazil

## Abstract

Simple imidazo­[1,2-*a*]­pyridine derivatives were synthesized via the GBB reaction, and their photophysical properties were studied for the first time. Fluorescence quantum yields confirmed a push–pull design, and absorption spectra showed a blue shift for bromo- and nitro-substituted derivatives at the *ortho* position (**4b**, **4d**), whereas the cyano-substituted analogs at the *para* position (**4f**, **4p**) exhibited a red shift. Solvent polarity significantly affected the fluorescence spectra, causing a red shift in polar solvents (**4p**, **4r**) and a blue shift in nonpolar solvents (**4i**, **4m**). A significant Stokes shift supported the involvement of an ICT mechanism in most cases. Some of the compounds demonstrated promising properties for use in fluorescent materials.

## Graphical abstract

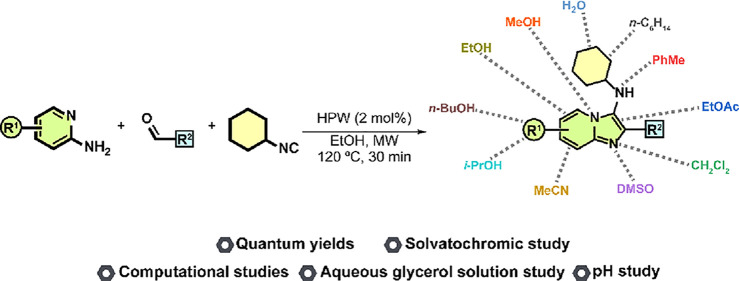

## Introduction

An intriguing class of compounds with a functionalized π-conjugated system is imidazo­[1,2-*a*]­pyridine derivatives. These compounds may present high fluorescence quantum yields,^1^ red shifts,^2^ and intense blue emissions^3^ (Figure 1). Their photophysical properties may arise from an internal charge transfer (ICT) or an excited-state intramolecular proton transfer (ESIPT) process.^4^ As a result, these heterocycles are used in bioimaging,^5^ chemosensors,^6^ and optoelectronics.^7^ Although several synthetic methods exist to access these compounds,^8^ most involve complex structures that are fused or linked to other (hetero)­cycles (Figure 1a),^9^ requiring nontrivial methodologies for their synthesis.

**Figure 1:**
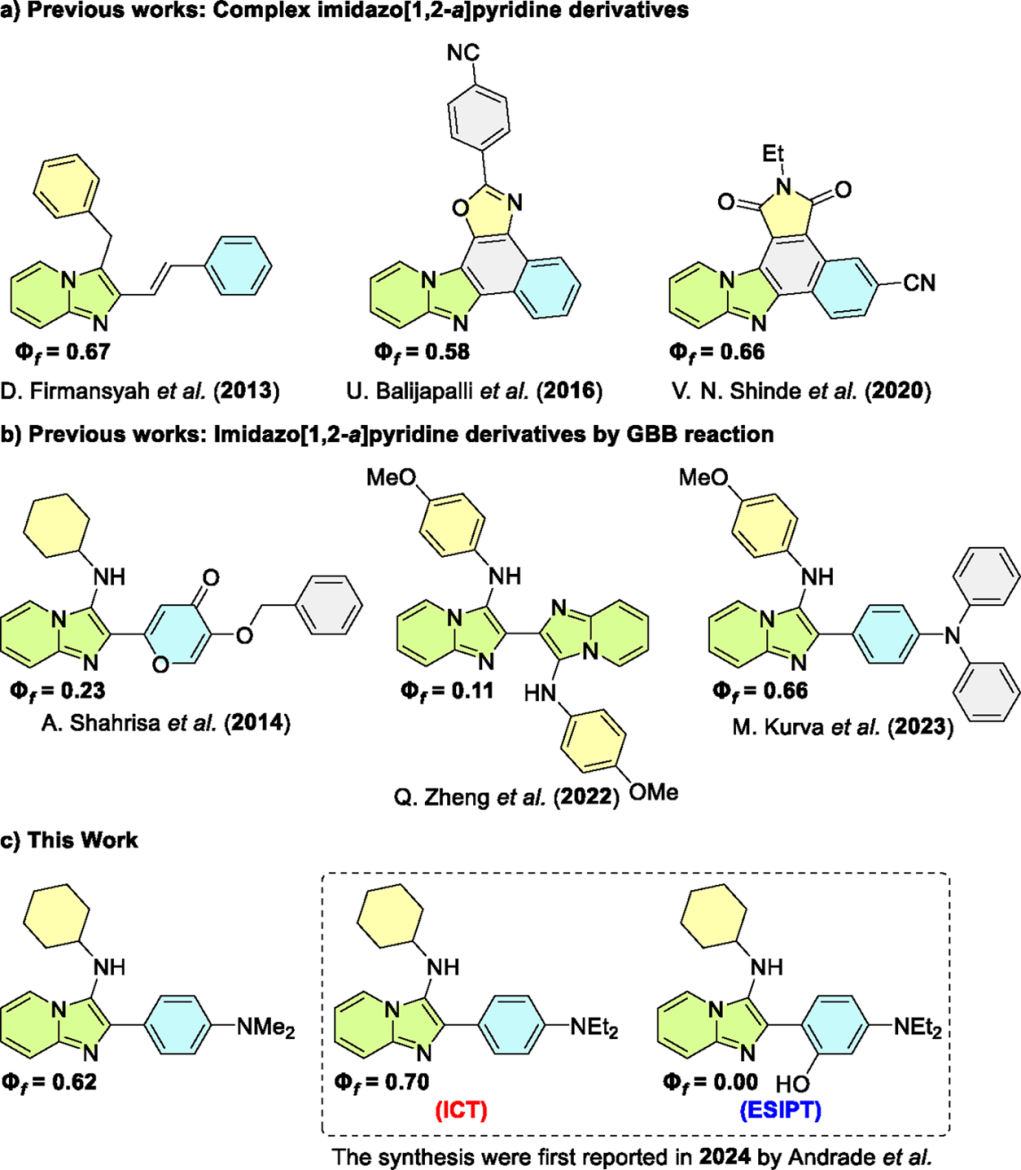
Selected examples of imidazo­[1,2-*a*]­pyridine derivatives with reported photophysical properties.

A remarkable approach to these molecules is the Groebke–Blackburn–Bienaymé three-component reaction (GBB-3CR), using amidines, aldehydes, and isocyanides under acid catalysis.^10^ This reaction offers high atom economy, high efficiency, fast reaction times, and excellent yields.^11^ Although this methodology has already been applied to compounds with notable photophysical properties, it often requires either difficult-to-obtain or very expensive starting materials (BODIPY nucleus,^12^ 4-pyrone linkage^13^ and triphenylamine^14^), or heterodimers^15^ (Figure 1b).

Noting the potential fluorescence properties provided by this nucleus, we explored the possibility of studying the photophysical properties of simple imidazo­[1,2-*a*]­pyridine derivatives synthesized from inexpensive materials by our group using a recent GBB-3CR method (Figure 1c).^16^ Our experiments revealed significant features across several solvents, as indicated by data obtained from quantum yields and the intensities of UV–vis absorption and fluorescence emission spectra. Additionally, interesting results were noted in aqueous media during pH and glycerol studies. The most significant results in this study were obtained from molecules that, to the best of our knowledge, had not been reported in the literature prior to 2024.

## Materials and Methods

### General

All reagents and solvents were purchased from Sigma-Aldrich and used without further purification unless otherwise specified. The aldehydes were distilled prior to their use. Cyclohexyl isocyanide was prepared according to the literature procedure.^17^ The solvents used in the photophysical study, including *n*-hexane, PhMe, EtOAc, CH_2_Cl_2_, DMSO, MeCN, *i*-PrOH, *n*-BuOH, EtOH, MeOH, and glycerol, were spectroscopic-grade. DMSO was previously dried using activated 3 Å molecular sieves. The aqueous solutions were prepared in Milli-Q ultrapure water (Millipore). The products were purified by column chromatography performed on silica gel (Supelco, pore size 60 Å, 230–400 mesh particle size, 40–63 μm particle size), and mixtures of hexane/ethyl acetate were used as eluents. Thin-layer chromatography was used on ultraviolet (UV) fluorescent silica gel Merck 60 F254 plates and visualized by treatment with a 10% solution of phosphomolybdic acid (PMA) in ethanol, followed by heating. The infrared (FT-IR) spectra were recorded on a Bruker Alpha II spectrometer with DLaTGS as the detector in the infrared region (4000–600 cm^–1^) in attenuated total reflection (ATR) mode (4000–500 cm^–1^). Nuclear magnetic resonance (NMR) spectra were obtained on a 600 MHz spectrometer (Bruker Ascend 600). Chemical shifts are given in ppm concerning residual ^1^H signals of CDCl_3_ (δ 7.26 ppm) or DMSO-*d*_6_ (δ 2.50 ppm), and ^13^C signals are referenced to the solvent signal (δ 77.2 ppm) or (δ 39.5 ppm). Exact masses were measured on a Triple Tof 5600 Sciex by flow injection analysis using an Eksigent UltraLC 100 Sciex chromatograph set to a flow rate of 0.3 mL/min. A DuoSpray Ion Source (ESI) was used, and the MS spectra were acquired in positive mode, employing external calibration, in the range of 50–1000 Da and 0.1% (*v/v*) formic acid in methanol as the solvent. The melting points were measured with a capillary in LOGEN Scientific equipment (LS III Plus) and were not corrected.

### Synthesis of Imidazo­[1,2-*a*]­pyridine Derivatives via GBB Three-Component Reaction

A Biotage microwave reaction vial of 0.5–2.0 mL containing a mixture of 2-aminopyridine (0.50 mmol), aldehyde (0.50 mmol), isocyanide (0.50 mmol), and phosphotungstic acid hydrate HPW (0.01 mmol, 2 mol %) in EtOH (0.5 mL) was introduced in the cavity of a microwave reactor (Biotage Initiator^+^) and heated at 120 °C for 30 min under magnetic stirring. The reaction mixture was cooled to room temperature, and reagent consumption was confirmed by TLC analysis (mixture of ethyl acetate/hexane). The reaction mixture was concentrated under vacuum, and the crude product was purified by silica gel column chromatography.^16^

### Crystal Structure Determination

The crystal structure of **4r** was solved using SHELXS^18^ and the refinement was accomplished using SHELXL^19^ with minimization of the least squares. The data collection was performed on a Bruker CCD SMART APEX II diffractometer, in which a graphite monochromator with Mo Kα (0.71073 Å) at 296 K was used. Data from the unit cell were obtained by collecting three matrices, each with 12 images, and the refinement was carried out with anisotropic parameters, using the OLEX2 program.^20^ Molecular graphics were generated via MERCURY software.^21^ Table S1 summarizes experimental details and refinement results. CCDC 2409958 for **4r** contains the supplementary crystallographic data.

### Fluorescence Quantum Yields

The method chosen to estimate the quantum yield of these compounds was a comparative method to a standard compound with a known quantum yield,^22^ employing the following mathematical equation:

$$\Phi_{f}=\Phi_{\mathrm{st}}\left(\frac{{\mathrm{grad}}_{\exp}}{{\mathrm{grad}}_{\mathrm{st}}}\right)\cdot {\left(\frac{\eta_{\exp}}{\eta_{\mathrm{st}}}\right)}^{2}$$ 1

in which Φ_st_ is the quantum yield described for the standard, the term Grad refers to the gradient of the fluorescence integrated area, and η is the refractive index of the solvent. For the determination of the quantum yields by the comparison method, a quinine sulfate solution was used as a standard, which has a known fluorescence quantum yield (Φ_st_ = 0.546).^23^ The solution was prepared from quinine monohydrate and was solubilized in a 0.5 M H_2_SO_4_ solution to obtain a concentration of 10^–5^ M. The stock solutions of the products were diluted in MeOH to obtain solutions with a concentration of 10^–5^ M. The analyses were carried out in a Fluorolog-Horiba spectrofluorometer at room temperature using standard 10 mm cells under emission mode with an excitation source at 366 nm (reference value for quinine sulfate), with a wavelength scan range of 386–700 nm every 1 nm and a slit of 2.0 nm. Data were collected in the form of graphs that were corrected for lamp noise, and the reading was lateral. With the data obtained, integrations of the curves were made and were used in eq 1. The data were analyzed using the OriginPro graphics program (Learning Edition - version 2025).

### Absorbance and Fluorescence Analyses

The UV–vis spectra were obtained from the UV–vis–NIR Cary 5000 spectrophotometer at room temperature using standard 10 mm cells in the double beam mode with the concomitant blank reading correction. The chosen scanning range was 800–200 nm with a data interval of 1.0 nm. Fluorescence emission measurements were performed at room temperature on the Horiba-Fluorologic spectrofluorometer in emission mode with an excitation wavelength from the maximum absorption wavelength of each product obtained from the absorption spectra, standard output: lateral, reading every 1 nm, and a slit of 2.0. Data were collected in the form of graphs, which were corrected for lamp noise. The data were analyzed using the OriginPro graphics program (Learning Edition – version 2025).

### Computational Methods

Molecules **4l**, **4m**, **4q**, and **4s** were built using UCSF Chimera^24^ and were optimized, and single-point energies were calculated using Orca 5.0 ^25^ at the CAM-B3LYP/6-31G* theory level.^26^ Molecular volumes were calculated using UCSF Chimera, and the topological polar surface areas and the Wildman–Crippen LogP were calculated using RDKit.^27^ Molecular orbital surfaces were generated with Multiwfn^28^ using Orca 5.0 output files.

## Results and Discussion

The Imidazo­[1,2-*a*]­pyridine molecules (**4a**–**u**) were synthesized through the GBB-3CR employing a recent methodology reported by our research group, which uses phosphotungstic acid (HPW) as an efficient catalyst in EtOH under microwave (MW) heating (Scheme 1).^16^ In those articles, the full details on the synthesis and characterization of Imidazo­[1,2-*a*]­pyridines **4a**–**i, 4l–o**, and **4q**–**s** are reported. The new compounds **4j**, **4k**, **4p**, **4t**, and **4u** were synthesized following the standard methodology. To better understand the fluorescence processes, X-ray diffraction analysis was performed to verify the spatial arrangement of atoms. For molecule **4r** (Figure 2), the analysis confirmed planarity along the heterocyclic chain, with the cyclohexyl group positioned out of the plane (Figures S23 and S24).

**Scheme 1:**
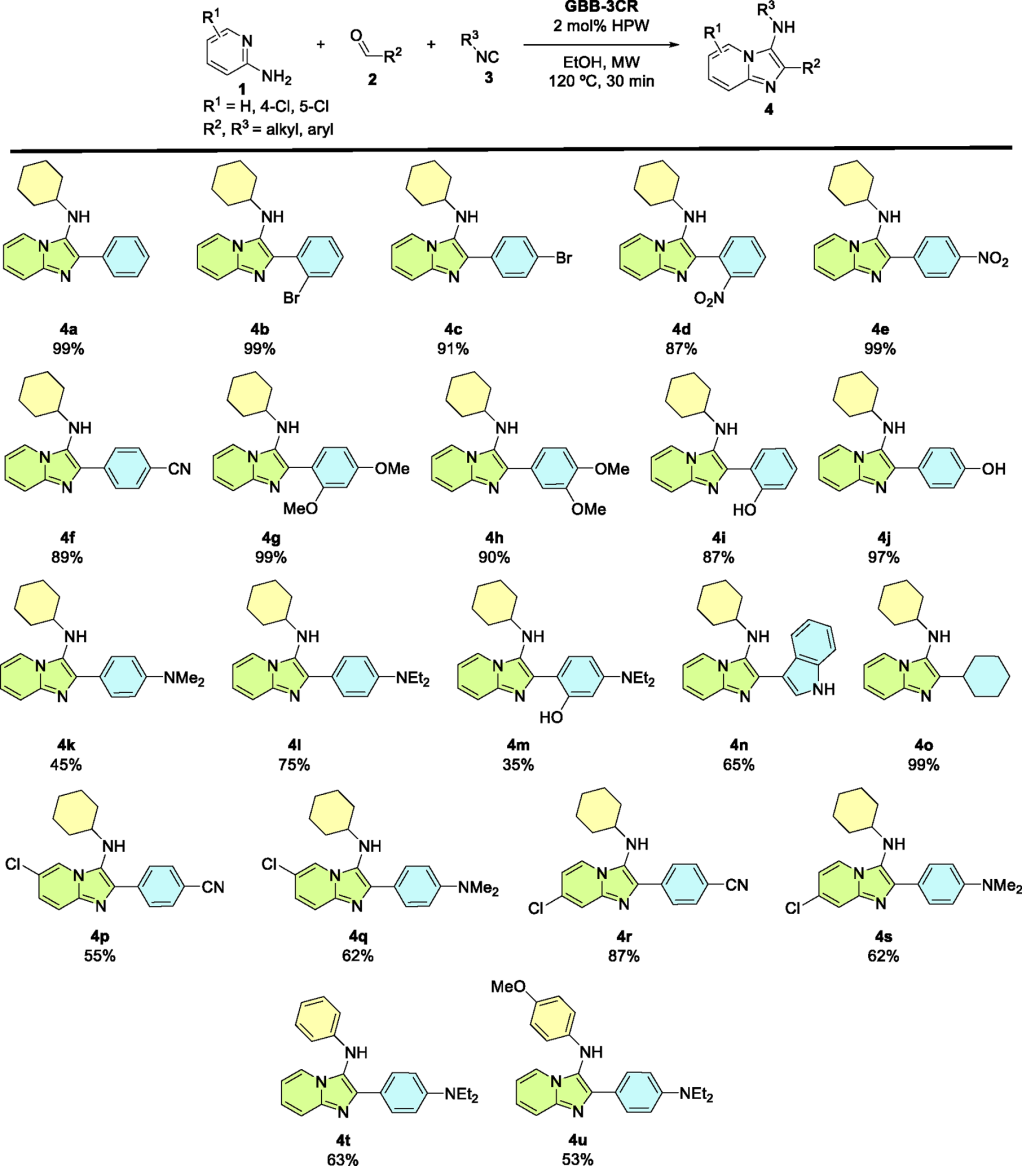
Substrate Scope of the HPW-Catalyzed GBB Multicomponent Reaction for the Synthesis of Imidazo­[1,2-*a*]­pyridines

**Figure 2:**
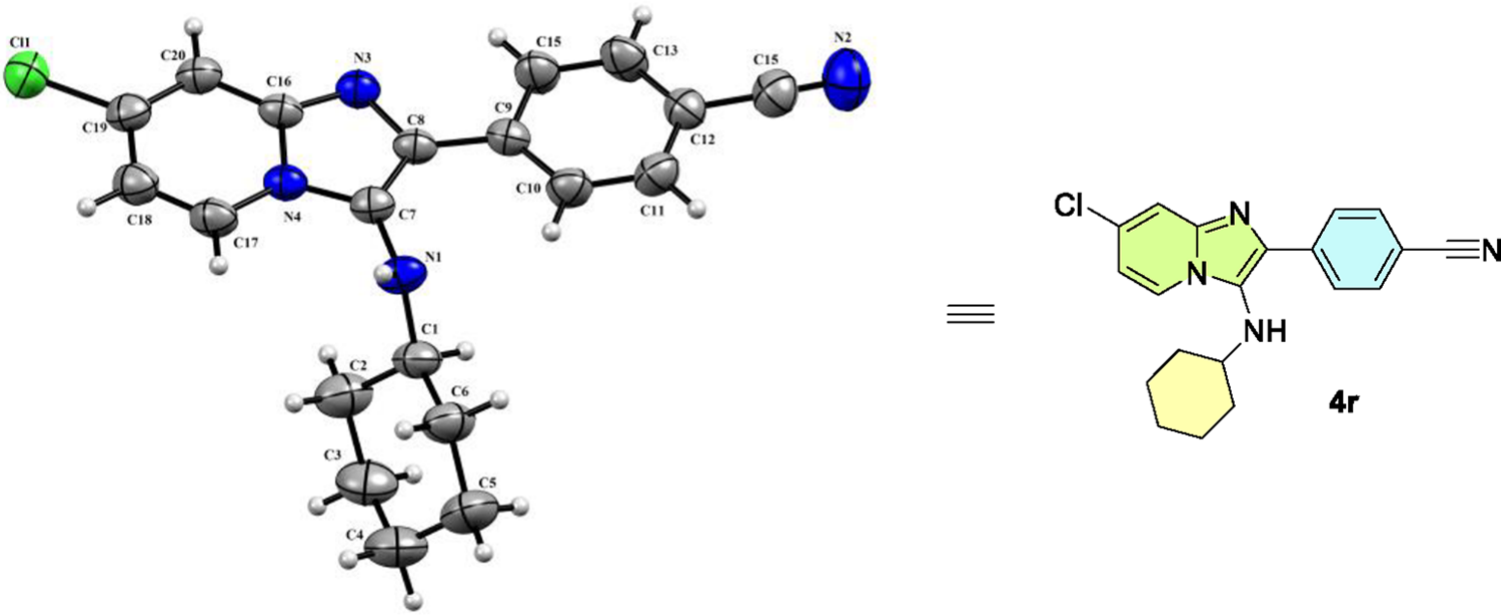
ORTEP diagram of compound **4r** drawn with a 50% ellipsoidal probability. Experimental details and refinement results are given in Table S1 (see the SI for additional details). CCDC 2409958 contains supplementary crystallographic data in a CIF file and can be obtained free of charge via the http://www.ccdc.cam.ac.uk/conts/retrieving.html.

Their fluorescence properties were analyzed by determining the quantum yields (Φ_f_) using quinine sulfate as the standard for a broad range of solvents (see Table S4 for different solvents), including polar aprotic (EtOAc, CH_2_Cl_2_, DMSO, and MeCN), polar protic (*i*-PrOH, *n*-BuOH, EtOH, MeOH, and H_2_O), and nonpolar (*n*-hexane and PhMe).^22^ In general, the use of *n*-hexane and H_2_O as solvents resulted in low quantum yield values, most likely due to a suppression effect associated with the poor solubility of the compounds in these media. This behavior is consistent with increased molecular aggregation, leading to aggregation-induced quenching (AIQ), which promotes nonradiative deactivation pathways.^29^

In this context, CH_2_Cl_2_ proved to be particularly effective, since some compounds exhibited their highest quantum yield values in this solvent (Table 1). Moreover, it is noteworthy that compounds **4k** (Figure S94) and **4l** (Figure S101) consistently exhibited the best quantum yield values across all the solvents investigated in this study.

**Table 1:** Photophysical Properties of All Compounds in CH_2_Cl_2_ ^a^

compounds	λ_abs_, nm^b^	λ_em_, nm^c^	log ε (ε, M^–1^ cm^–1^)^d^	stokes shift, cm^–1^ ^e^	Φ_f_ ^f^
**4a**	340	477	3.73 (5384)	8447	0.39
**4b**	330	469	3.53 (3356)	8981	0.01
**4c**	341	469	3.97 (9290)	8004	0.51
**4d**	319	-^g^	3.73 (5400)	-	0.00
**4e**	381	-^g^	4.00 (9985)	-	0.00
**4f**	351	496	4.01 (10,313)	8329	0.46
**4g**	340	489	3.79 (6220)	8962	0.43
**4h**	341	478	3.94 (8756)	8405	0.40
**4i**	342	478	3.96 (9076)	8319	0.03
**4j**	340	478	3.85 (7056)	8491	0.04
**4k**	351	483	4.17 (14,818)	7786	0.84
**4l**	356	485	4.21 (16,197)	7471	0.68
**4m**	366	551	4.34 (21,655)	9174	0.02
**4n**	345	482	3.80 (6247)	8239	0.31
**4o**	327	475	3.49 (3079)	9528	0.01
**4p**	355	498	4.04 (10,915)	8089	0.65
**4q**	366	511	3.99 (9849)	7753	0.01
**4r**	358	500	4.01 (10,318)	7933	0.56
**4s**	364	502	4.21 (16,110)	7552	0.55
**4t**	351	456	4.24 (17,317)	6560	0.20
**4u**	352	459	4.04 (10,975)	6623	0.28

a Carried out at room temperature (5 × 10^–5^ M).

b λ_abs_ = absorption maxima (nm).

c λ_em_ = emission maxima (nm).

d ε = molar absorptivity (M^–1^ cm^–1^).

e Stokes shifts are the difference between λ_em_ and λ_abs_.

f Φ_f_ = fluorescence quantum yields (10^–5^ M) were measured at room temperature (366 nm) with reference to quinine sulfate in 0.5 M H_2_SO_4_ (Φ_st_ = 0.546).

g Fluorescence was not observed in this solvent.

The unsubstituted compound **4a** (from cyclohexyl isocyanide) showed a Φ_f_ of 39% in CH_2_Cl_2_ (See Table S4 for different solvents). Similar results were obtained for other aliphatic isocyanides, including *tert*-butyl and methyl isocyanoacetate.^30^ Accordingly, cyclohexyl isocyanide was further chosen for this study due to its high availability and cost-effectiveness. Halogen substituents on the aromatic ring were evaluated, revealing that bromine at the *ortho*-position (**4b**) suppressed fluorescence (Φ_f_ = 1%), whereas at the *para*-position (**4c**) it led to an increase in the quantum yield (Φ_f_ = 51%).^31^

Compounds containing nitro groups (**4d**, **4e**) exhibited a quenching effect, with no appreciable Φ_f_ values.^32^ The presence of nitrile or methoxy groups (**4f**–**h**) enhanced fluorescence, with values between 40 and 46%. The hydroxy group at the *ortho-*position (**4i**), whose Φ_f_ value is similar to that of the *para*-substituted analogue (**4j**), promotes ESIPT rather than ICT (Scheme S2a in the Supporting Information).^33^

Electron-rich substituents like dialkylamines (−NMe_2_, −NEt_2_) at the *para-*position (**4k**, **4l**) achieved high quantum yields (up to 84%), promoting an ICT process (Scheme S2b).^1^ Interestingly, a superior result was obtained compared to those reported by Kurva et al.^14^ for triphenylamine derivatives, which used a far more expensive starting material (4-(diphenylamine) benzaldehyde), compared to the 4-diethylamine derivatives. On the other hand, an *ortho-*hydroxy group (**4m**) confirmed its fluorescence-quenching effect (Φ_f_ = 2%). This observation indicates the deactivating effect of this group, facilitating an ESIPT mechanism rather than the anticipated ICT mechanism due to the presence of the dialkylamine group. Derivative **4n** with an indole ring showed a Φ_f_ of 31%. The aliphatic aldehyde derivative **4o** showed a lower quantum yield (Φ_f_ = 1%) compared to aromatic ones. Chlorine at position 5 (**4p**, **4q**) resulted in either an increase or a reduction in fluorescence, depending on the presence of electron-withdrawing or electron-donating substituents, respectively, but had a minimal effect at position 4 (**4r**, **4s**). A low Φ_f_ of 20% was observed for phenyl isocyanides with the 4-NEt_2_ derivative (**4t**). Upon incorporation of a methoxy group in the aromatic ring of the isocyanide, the Φ_f_ increased to 28% (**4u**), a higher value compared to that reported by Zheng et al.^15^ and a lower value than that described by Kurva et al.^14^ for this substituent effect. These findings align with the push–pull donor–acceptor (DA) behavior of fluorophores.^34^

The UV–vis absorbance and fluorescence spectra of the Imidazo­[1,2-*a*]­pyridine derivatives were measured in a broad range of solvents, including polar aprotic (EtOAc, CH_2_Cl_2_, DMSO, and MeCN) and polar protic (*i*-PrOH, *n*-BuOH, EtOH, MeOH, and H_2_O) (Figure 3a). Notably, nonpolar solvents, including *n*-hexane and PhMe, seldom utilized in studies of this nature owing to the limited solubility of the compounds in such media,^14^ were incorporated into the investigation. The spectroscopic data and the absorption spectra for all compounds in CH_2_Cl_2_ were summarized in Table 1 (see SI for the complete set of values and spectra). In general, Imidazo­[1,2-*a*]­pyridine derivatives exhibit similar UV–vis absorption profiles. Compared to the unsubstituted compound **4a**, structures with electron-withdrawing groups in the *ortho* position (**4b**, **4d**) displayed a blue shift, while those with *para* substituents (**4c**, **4e**, **4f**, **4p**, **4r**) showed a red shift, characterized by an intense absorption band centered around 341–381 nm, attributed to π→π* transitions. Compounds with electron-rich substituents, such as −OMe (**4g**, **4h**) and −OH (**4i**, **4j**), showed no significant changes. On the other hand, *para*-substituted groups like −NMe_2_ (**4k**, **4q**, **4s**), −NEt_2_ (**4l**, **4m**), and indole (**4n**) induced a red shift, with maximum UV–vis absorption extending up to 366 nm, accompanied by a remarkable hyperchromic shift (Figure 3b). These findings indicate more delocalized and extended π-conjugated systems that enhance π···π* transitions at longer wavelengths. Overall, the solvatochromic effect did not significantly shift the UV–vis absorption spectra. Notable red shifts were seen for compounds **4f**, **4l**–**n**, **4q**, and **4s** in aqueous medium, likely due to strong hydrogen bonding interactions.

**Figure 3:**
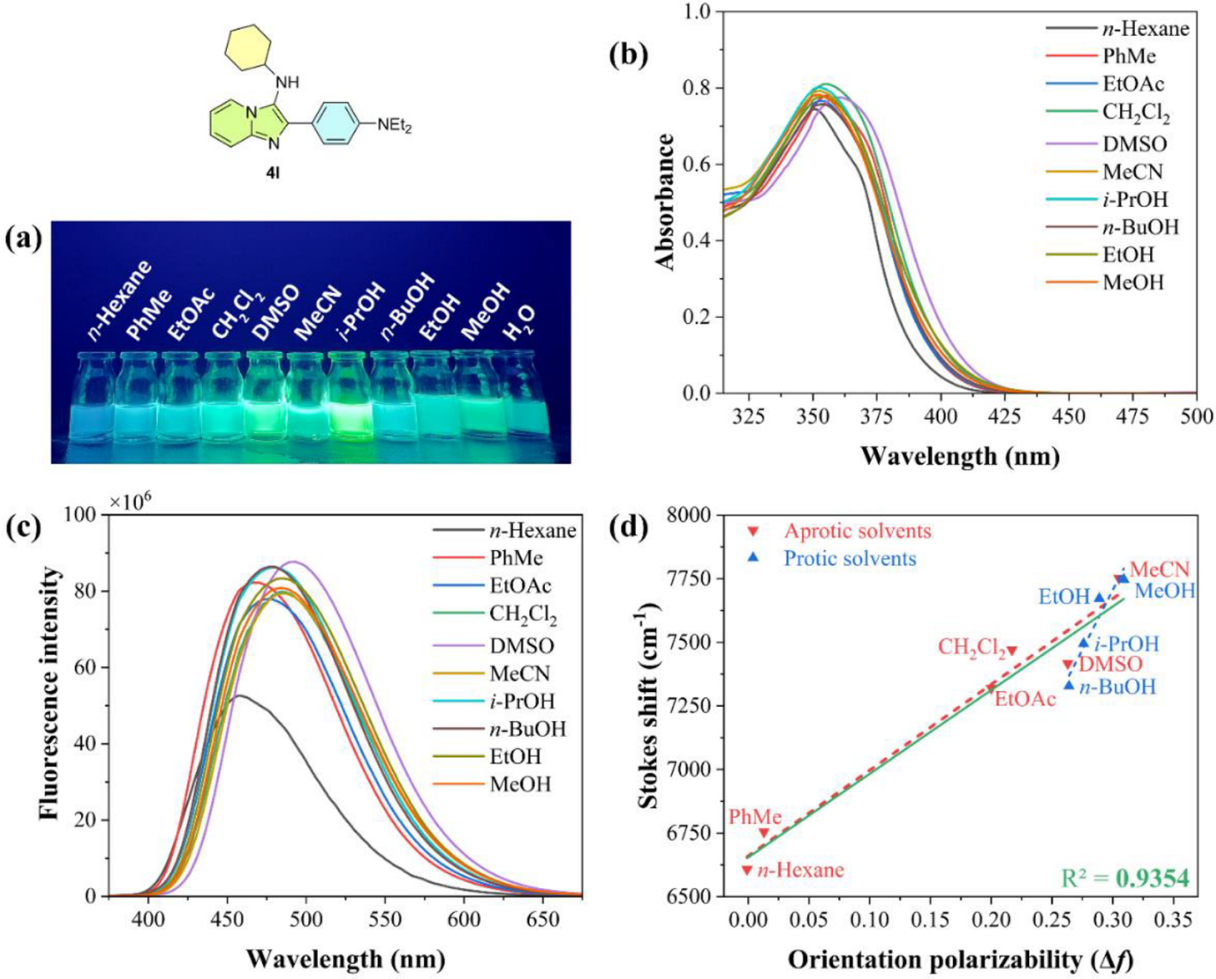
(a) Emission solvatochromism for compound **4l** (5 × 10^–5^ M) at room temperature; λ_exc_ = 365 nm, in a dark chamber with a UV lamp. (b) UV–vis absorption spectra of **4l** in different solvents (5 × 10^–5^ M) at room temperature. (c) Normalized emission spectra of **4l** in different solvents (5 × 10^–5^ M) at room temperature. (d) Lippert–Mataga plot showing Stokes shift as a function of solvent orientation polarizability (Δ*f*) for compound **4l**.

The fluorescence emission spectra of compound **4l** in different solvents are depicted in Figure 3c. Generally, Imidazo­[1,2-*a*]­pyridine derivatives exhibited strong fluorescence emissions ranging from blue to green, with maximum emission peaks varying between 469 and 496 nm for compounds with electron-withdrawing substituents (**4b**, **4c**, **4f**) and 478 to 551 nm for compounds with *para*-substituted electron-donating groups (**4g, 4** **h**, **4j**–**m**) in comparison to the parent compound with an unsubstituted aromatic ring (**4a**).

For the compound containing a bromine substituent in the *ortho* position (**4b**), the electron-withdrawing effect was confirmed, as evidenced by the lower fluorescence intensity and a slight blue shift compared to compound **4a**. The notable difference corresponds to the quenching effect of the bromine in the excited state. Heavier halogens can also speed up the intersystem crossing (ISC) from singlet to triplet states.^35^ Similar results were observed for compounds containing a nitro group (**4d**, **4e**), for which no fluorescence emission was detected. This is consistent with their well-established role as deactivating groups, promoting ISC or internal conversion processes.^36^ Conversely, the presence of nitrile substituents (**4f**, **4p**, **4r**), or electron-donating groups, such as methoxy (**4g**, **4h**), hydroxy (**4i**, **4j**), or dialkylamino (**4k**–**m**, **4q**, **4s**) moieties, induced a red shift relative to compound **4a**, attributed to enhanced π-conjugation in these systems. Therefore, the fluorescence emission spectra exhibited a strong dependence on the solvent polarity. Increased fluorescence and red shifts were noted in polar solvents, while blue shifts and fluorescence suppression occurred in nonpolar solvents due to low solubility and a lack of excited-state stabilization. These changes suggest the intermediacy of an ICT mechanism, as solvent polarity enhances charge separation in the excited state.^37^

Compounds containing nitrile electron-withdrawing groups (**4f**, **4p**, and **4r**) exhibited strong fluorescence in aprotic solvents and fluorescence quenching in polar protic solvents. This behavior suggests that charge stabilization is not a predominant factor in the excited state for these compounds, and an ICT mechanism may not be involved.^38^

Finally, *ortho*-hydroxy groups (**4i**, **4m**) exhibited high Stokes shifts in nonpolar solvents, consistent with the findings of Douhal et al.^2^ These compounds are known to follow an ESIPT-type fluorescence mechanism rather than ICT. Consequently, the use of polar protic solvents disrupts the intramolecular hydrogen bond stabilization between the phenol and amino groups in their ground-state planar conformation.^33^

A comparison of absorption (λ_abs_) and emission (λ_em_) data revealed significant Stokes shifts, indicating excited-state stability.^39^ Apolar or less polar solvents (*n*-Hexane, PhMe, EtOAc, and CH_2_Cl_2_) showed lower Stokes shifts compared to more polar solvents (*i*-PrOH, *n*-BuOH, EtOH, and MeOH), due to varying solvation properties affecting excited-state energy.

The Lippert–Mataga plots (Figure 3d) were obtained for compound **4l** to examine the relationship between the significant Stokes shift values and solvent orientation polarizability (Δ*f*) (see SI for the complete set of spectra).^40^ These plots provide insights into the stabilization mechanism in the excited state in relation to the ICT process and solvent polarity.^41^

The graphs showed linear characteristics, especially for compounds with electron-donating groups in the *para*-position (**4g**, 4**h**, **4j**–**l**). Compounds **4b**, **4i**, **4m**, **4f**, **4p**, and **4r** exhibited nonlinear trends, suggesting relaxation through the ESIPT process. Some slightly lower *R* ^2^ values indicate two distinct linearity trends between protic and aprotic solvents for compounds **4j**, **4k**, **4n**, and **4q**. Similar results, with a slight decrease in the *R* ^2^ value, were also observed in the Dimroth–Reichardt plot against the E_T_(30) parameter, which further highlighted the presence of two more pronounced trends between protic and aprotic solvents.^37^

Water plays a significant role in fluorescence processes through hydrogen bonding and solvation.^42^ These interactions can either facilitate or hinder mechanisms such as ESIPT and ICT, making it crucial to study fluorescence behavior in aqueous media.^43^ This is essential for applications like chemical sensing and bioimaging, due to the ubiquity of water in biological environments and its impact on the fluorescent response of molecular probes.^44^

Given the relevance of aqueous media in such applications, a systematic study was carried out (see SI for the complete set of spectra). The aqueous medium posed challenges due to the low solubility of imidazopyridine compounds, leading to a hypochromic effect in absorption spectra.^43^ In emission spectra, aggregation-caused quenching (ACQ) was observed, likely due to proton transfer between the chromophore and water via hydrogen bond disruption.^45^

A photophysical study was carried out in aqueous solution at varying pH levels for selected compounds **4a**, **4i**, **4k**–**m**, **4q**, and **4s** (see SI for the complete set of spectra), as pH is known to significantly influence absorption and emission processes.^46^ The chosen pH values were acidic (∼1), neutral (∼7), and basic (∼13). Compared to the neutral medium, the absorption spectra revealed a hyperchromic effect and intense blue shifts in acidic conditions, with absorption bands centered at around 311–320 nm, whereas a hypochromic effect and red shifts (350–392 nm) were observed in basic conditions (Table S5).

In the fluorescence spectra, strong emissions in the blue (454–550 nm) were observed in acidic media, while fluorescence quenching occurred in basic conditions. This behavior suggests that protonation of basic sites enhances solubilization, stabilizing charges in the excited state through strong hydrogen-bonding interactions and thereby increasing Stokes shifts. These findings indicate the presence of phototautomerism effects in both the ground and excited states.^47^ Consequently, at the investigated pH levels, at least three species — cationic, neutral, and anionic—exist in equilibrium in their fundamental states (Scheme S3).

Next, glycerol studies on compounds **4a** (see SI) and **4l** (Figure 4a) were carried out using fluorescence spectroscopy in aqueous glycerol solutions (0–100%, *v*/*v*). The results for both compounds aligned with expectations, as literature reports suppression effects or emission variations depending on the glycerol concentration.^48^

**Figure 4:**
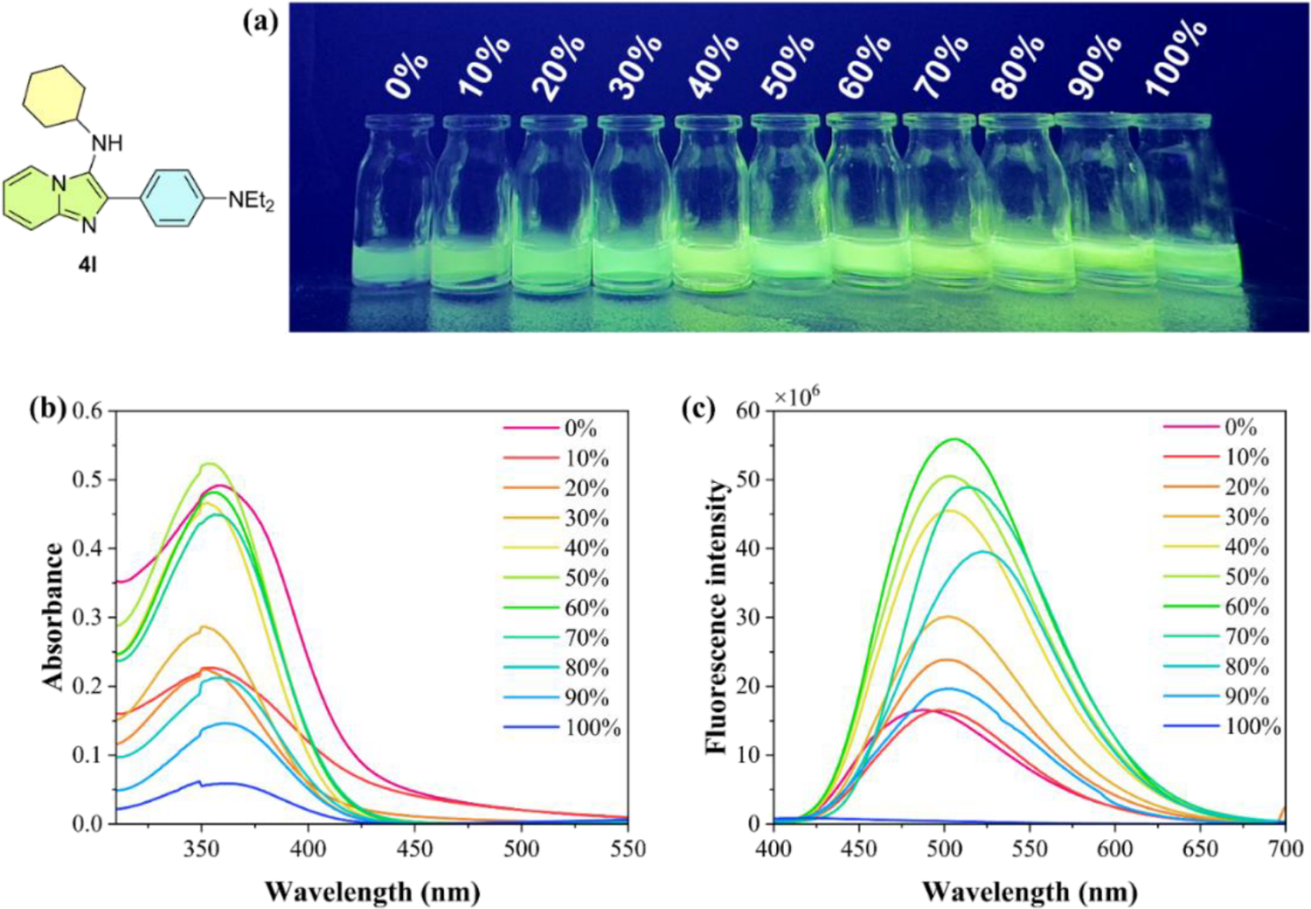
(a) Photophysical study of compound **4l** in glycerol/water with increasing the glycerol content (5 × 10^–5^ M) at room temperature; λ_exc_ = 365 nm, in a dark chamber with a UV lamp. (b) UV–vis absorption spectra. (c) Normalized emission spectra of **4l**.

For instance, in the case of compound **4l**, an increase in glycerol concentration resulted in a slight decrease in absorption intensity (Figure 4b), with a blue shift of about 10 nm (from 359 nm in 100% water to 349 nm in 100% glycerol) (Table S6). The emission spectra display a gradual red shift as the glycerol fraction increases, up to 80%. At 100% glycerol, an abrupt blue shift occurs, accompanied by fluorescence quenching (Figure 4c). The decrease in Stokes shift values with increasing glycerol concentration indicates that glycerol affects photophysical processes by altering viscosity, refractive index, and polarizability, impacting both ground and excited states.^49,50^

A computational study of analogs **4l**, **4m**, **4q**, and **4r** revealed distinct ionization energies, electron affinities, and HOMO–LUMO gaps (Table 2 and Figure S265).^26^ Notably, in the first excited state, the electronic density increases over the Imidazo­[1,2-*a*]­pyridine moiety, especially in the **4l**, **4m,** and **4q**–**s** pairs. Compound **4m** showed a higher polarity and an enhanced hydrogen bonding capacity due to the hydroxy group, supporting the ESIPT fluorescence. Compound **4l** had a higher partition coefficient (cLogP), indicating a lower polarity.

**Table 2:** General Properties at the CAM-B3LYP/6-31G* Theory Level^26^ for the Four Analogs Studied

	**4l**	**4m**	**4q**	**4s**
ionization energy (IE)^a^	5.8783	5.7575	6.1198	6.1018
electron affinity (EA)^a^	–0.6417	–0.3603	–0.2979	–0.3095
HOMO–LUMO gap^a^	6.5200	6.1178	6.4177	6.4113
molecular volume^b^	348.9	354.2	339.5	339.6
TPSA^c^	30.54	50.77	30.54	30.54
cLogP	3.92	2.89	3.36	3.36

a Energies in eV.

b Volumes in Å^3^.

c Areas in Å^2^.

## Conclusions

In summary, we synthesized a variety of simple imidazo­[1,2-*a*]­pyridine derivatives via the GBB reaction and studied their photophysical properties. Unlike prior studies documented in the literature, this research encompasses a broader selection of commercially available and cost-effective aldehydes bearing diverse functional groups. Furthermore, the photophysical behavior of these derivatives was analyzed in a variety of solvents not typically employed in studies of this nature (PhMe, *n*-hexane, *i*-PrOH, and *n*-BuOH). Aqueous media were also explored, given their significance in applications, such as chemical sensing and bioimaging. Selected compounds underwent pH and glycerol variation studies, yielding promising outcomes. Notably, compound **4l** exhibited outstanding photophysical properties.

## Supplementary Material





## Notes

CCDC 2409958 contains supplementary crystallographic data in CIF file and can be obtained free of charge via http://www.ccdc.cam.ac.uk/conts/retrieving.html (CIF)
Experimental procedures and characterization data (PDF)
