# Supplementary material for: Solvent Effects on the Photophysical Properties of Unexplored Imidazo[1,2‑a]pyridine Derivatives
Source: ACS Omega. 2026 Mar 13;11(11):17186–95. doi: 10.1021/acsomega.5c07201 (PMC13019203; doi:10.1021/acsomega.5c07201)
Supplement: Supplementary file 2 [file ao5c07201_si_002.pdf]

Solvent effects on the photophysical properties of unexplored imidazo[1,2-*a*]pyridine derivatives

Victor H. J. G. Praciano,[a] Luan A. Martinho,[a] Guilherme D. R. Matos,[b] Claudia C. Gatto,[c] and Carlos Kleber Zago de Andrade[a]*

[a]Instituto de Química, Laboratório de Química Metodológica e Orgânica Sintética (LaQMOS), Universidade de Brasília, Campus Universitário Asa Norte, 70904-970, Brasília, DF, Brazil.

[b]Instituto de Química, Laboratório de Modelagem de Sistemas Complexos (LMSC), Universidade de Brasília, Campus Universitário Asa Norte, 70904-970, Brasília, DF, Brazil.

[c]Instituto de Química, Laboratório de Síntese Inorgânica e Cristalografia (LASIC), Universidade de Brasília, Campus Universitário Asa Norte, 70904-970, Brasília, DF, Brazil.

*email: ckleber@unb.br

Supporting Information

Table of contents

1. Materials and methods S2
1.1 General S2
1.2 Synthesis of imidazo[1,2-*a*]pyridine derivatives via GBB three-component reaction S2
1.3 Crystal structure determination S3
1.5 Absorbance and fluorescence analyses S4
1.6 Computational methods S4
2. Synthesis of imidazo[1,2-*a*]pyridine derivatives via GBB-3CR S5
2.1 Spectroscopic data for new compounds S7
3. Crystal structure determination of compound 4r S20
4. Solvatochromic study S24
5. Photophysical study in aqueous medium S103
5.1 Influence of pH on the absorption and fluorescence spectra in aqueous solution S120
5.2 Aqueous glycerol solution study S147
6. Computational studies S156
References S157

1. Materials and methods

1.1 General

All reagents and solvents were purchased from Sigma-Aldrich Merck and used without further purification unless otherwise specified. The aldehydes were distilled prior to their use. Cyclohexyl isocyanide was prepared according to literature procedure.[1] The solvents used in the photophysical study, including *n*-hexane, PhMe, EtOAc, CH_2Cl_2, DMSO, MeCN, *i*-PrOH, *n*-BuOH, EtOH, MeOH, and glycerol, were of spectroscopic grade. DMSO was previously dried using activated 3 Å molecular sieves. The aqueous solutions were prepared in Milli-Q ultrapure water (Millipore). The products were purified by column chromatography performed on silica gel (Supelco, pore size 60 Å, 230−400 mesh particle size, 40−63 μm particle size), and mixtures of hexane/ethyl acetate were used as eluents. Thin-layer chromatography was used on the ultraviolet (UV) fluorescent silica gel Merck 60 F254 plates and visualized by treatment with a 10% solution of phosphomolybdic acid (PMA) in ethanol, followed by heating. The infrared (FT-IR) spectra were performed on a Bruker Alpha II spectrometer with DLaTGS as the detector in the infrared region (4000−600 cm^{-1}) in Attenuated Total Reflection (ATR) mode (4000−500 cm^{-1}). Nuclear magnetic resonance (NMR) spectra were obtained on a 600 MHz spectrometer (Bruker Ascend 600). Chemical shifts are given in ppm concerning residual ^1H signals of $CDCl_3$ (δ 7.26 ppm) or DMSO-d_6 (δ 2.50 ppm), and ^{13}C signals are referenced to the solvent signal (δ 77.2 ppm) or (δ 39.5 ppm). Exact masses were measured on a Triple Tof 5600 Sciex by flow injection analysis using an Eksigent UltraLC 100 Sciex chromatograph set to a flow rate of 0.3 mL/min. A DuoSpray Ion Source (ESI) was used, and the MS spectra were acquired in positive mode, employing external calibration, in the range of 50−1000 Da and 0.1% (*v/v*) formic acid in methanol as solvent. The melting points were measured with a capillary in LOGEN Scientific equipment (LS III Plus) and were not corrected.

1.2 Synthesis of imidazo[1,2-*a*]pyridine derivatives via GBB three-component reaction

A Biotage microwave reaction vial of 0.5–2.0 mL containing a mixture of 2-aminopyridine (0.50 mmol), aldehyde (0.50 mmol), isocyanide (0.50 mmol), and phosphotungstic acid hydrate (HPW) (0.01 mmol, 2 mol%) in EtOH (0.5 mL) was introduced into in the cavity of a microwave reactor (Biotage Initiator$^+$) and heated at 120 °C for 30 min under magnetic stirring. The reaction mixture was cooled to room temperature and reagents consumption was confirmed by TLC analysis (mixture of ethyl acetate/hexane). The reaction mixture was concentrated under vacuum, and the crude product was purified by silica gel column chromatography.[2]

1.3 Crystal structure determination

The crystal structure of **4r** was solved using SHELXS[3] and the refinement was accomplished using SHELXL[4] with minimization of least squares. The data collection was performed on a Bruker CCD SMART APEX II diffractometer, in which a graphite monochromator with a Mo-Kα (0.71073 Å) at 296 K was used. Data from the unit cell was obtained by collecting three matrices, each with twelve images and the refinement was carried out with anisotropic parameters, using the OLEX2 program.[5] Molecular graphics were generated via MERCURY software.[6] Table S1 summarizes experimental details and refinement results. CCDC number 2409958 for **4r** contains supplementary crystallographic data.

1.4 Fluorescence Quantum yields

The method chosen to estimate the quantum yield of these compounds was a comparative method to a standard compound with a known quantum yield,[7] employing the following mathematical equation:

$$\Phi_f = \Phi_{st}\left(\frac{Grad_{exp}}{Grad_{st}}\right) \cdot \left(\frac{\eta_{exp}}{\eta_{st}}\right)^2 \qquad \text{(Eq. 1)}$$

in which Φ_{st} is the quantum yield described for the standard, the term Grad refers to the gradient of the fluorescence integrated area, and η is the refractive index of the solvent.

For determination of the quantum yields by the comparison method, a quinine sulfate solution was used as standard, which has a known fluorescence quantum yield (Φ_{st} = 0.546).[8] The solution was prepared from quinine monohydrate, and solubilized in a 0.5 M H_2SO_4 solution, to obtain a concentration of 10^{-5} M. The stock solutions of the products were diluted in MeOH to obtain solutions with a concentration of 10^{-5} M. The analyses were carried out in a Fluorolog-Horiba spectrofluorometer at room temperature using standard 10 mm cells under emission mode with an excitation source at 366 nm (reference value for quinine sulfate), reading with a wavelength scan range of 386-700 nm every 1 nm and slit of 2.0. Data was collected in the form of graphs that were corrected for lamp noise, and the reading was lateral. With the data obtained, integrations of the curves were made and used in Equation 1. The data were analyzed using the OriginPro graphics program (Learning Edition - version 2025).

1.5 Absorbance and fluorescence analyses

The UV-Vis spectra were obtained from the UV-Vis-NIR – Cary 5000 spectrophotometer at room temperature and standard 10 mm cells, using the double beam mode with the concomitant blank reading correction. The chosen scanning range was 800 to 200 nm with a data interval of 1.0 nm. Emission of fluorescence measurements were performed at room temperature on the Horiba-Fluorologic spectrofluorometer in emission mode with an excitation wavelength from the maximum wavelength absorption of each product obtained from the absorption spectra, standard output: lateral, reading every 1 nm and slit of 2.0. Data was collected in the form of graphs, which were corrected for lamp noise. The data were analyzed using the OriginPro graphics program (Learning Edition - version 2025).

1.6 Computational methods

Molecules **4l**, **4m**, **4q**, and **4s** were built using UCSF Chimera[9] and were optimized and single point energies were calculated using Orca 5.0[10] at the CAM-B3LYP/6-31G* theory level.[11] Molecular volumes were calculated using UCSF Chimera, and the topological polar surface areas and the Wildman-Crippen LogP were calculated using RDKit.[12] Molecular orbitals surfaces were generated with Multiwfn[13] using Orca 5.0 output files.

2. Synthesis of imidazo[1,2-*a*]pyridine derivatives via GBB-3CR

The imidazo[1,2-*a*]pyridine molecules (**4a-u**) were synthesized through GBB-3CR employing a recent methodology reported by our research group, which uses phosphotungstic acid (HPW) as an efficient catalyst in EtOH under microwave (MW) heating.[2] In those articles, the full details on the synthesis and characterization of imidazo[1,2-*a*]pyridines **4a-i, 4l-o, 4q-s** are reported. The new compounds **4j**, **4k**, **4p**, **4t** and **4u** were synthesized following the standard methodology and were characterized herein.

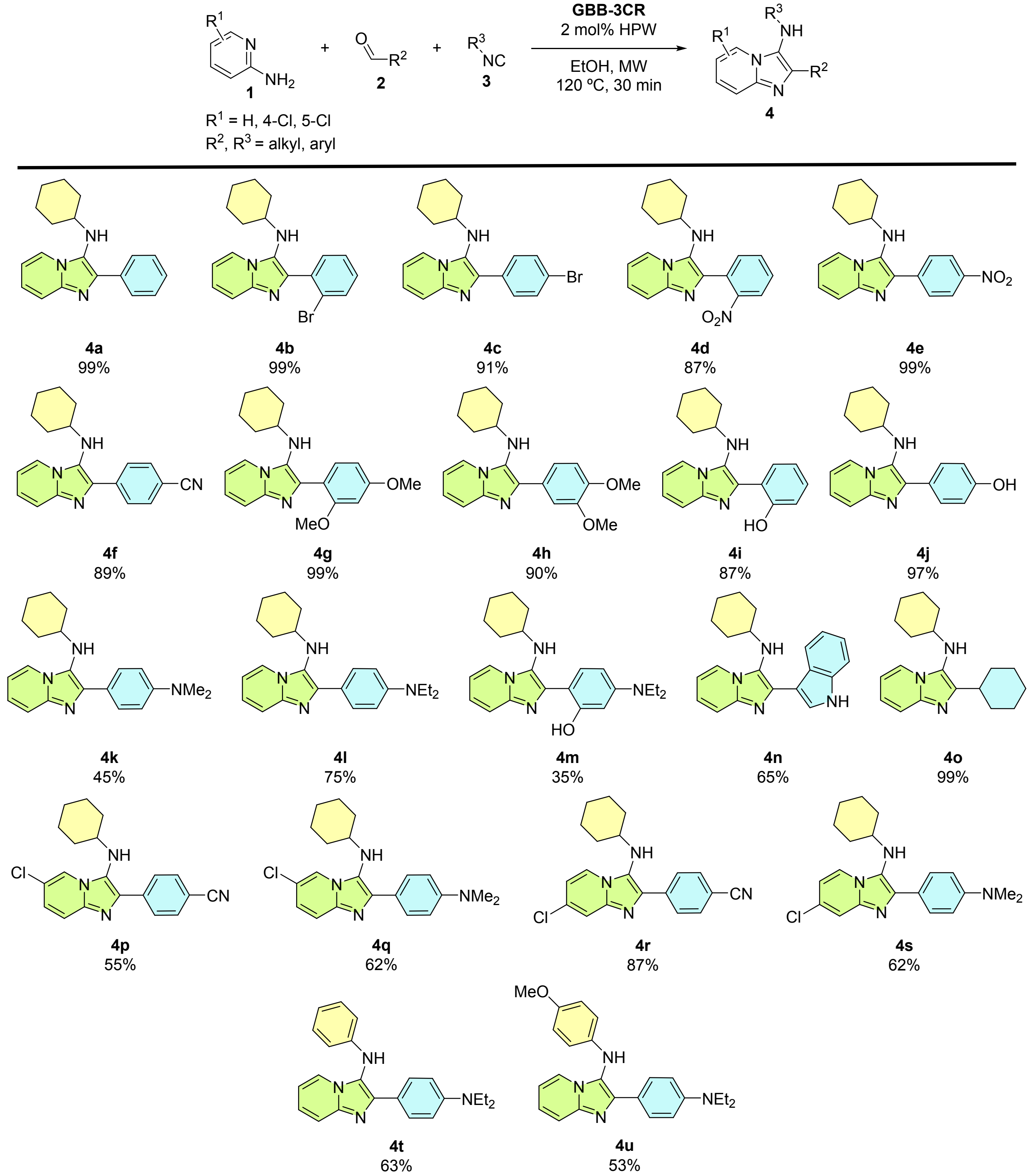

Scheme S1. Substrate scope of the HPW-catalyzed GBB multicomponent reaction for the synthesis of imidazo[1,2-*a*]pyridines. Reaction conditions: 2-aminopyridine (0.50 mmol), aldehyde (0.50 mmol), isocyanide (0.50 mmol), and HPW (0.01 mmol, 2 mol%) in EtOH (0.5 mL), under microwave (MW) heating. The yields refer to isolated yields and the structures were confirmed by FT-IR, NMR, and HRMS.

2.1 Spectroscopic data for new compounds

4-(3-(cyclohexylamino)imidazo[1,2-*a*]pyridin-2-yl)phenol **(4j)**

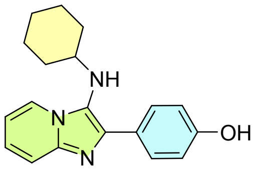

4j

4j was obtained from 2-aminopyridine (0.047 g; 0.50 mmol), 4-hydroxybenzaldehyde (0.061 g; 0.50 mmol), HPW (0.029 g; 2 mol%), cyclohexyl isocyanide (0.062 mL; 0.50 mmol) in EtOH (0.5 mL), in 97% yield as a light-yellow solid (m.p.: 241-242 ºC). $R_f = 0.47$ (80% EtOAc/Hexane).

FT-IR (ATR): 3293, 2928, 2854, 1633, 1608, 1588, 1565, 1510, 1500, 1444, 1387, 1364, 1272, 1225, 1196, 1167, 1102, 837, 749, 732, 552 cm^{-1}.

^{1}H NMR (600 MHz, DMSO-d_6): δ 9.47 (s, 1H), 8.26 (dt, J = 6.8, 1.2 Hz, 1H), 8.05 – 7.99 (m, 2H), 7.41 (dt, J = 9.0, 1.2 Hz, 1H), 7.12 (ddd, J = 9.0, 6.8, 1.2 Hz, 1H), 6.84 (td, J = 6.8, 1.2 Hz, 1H), 6.82 – 6.78 (m, 2H), 4.63 (d, J = 5.5 Hz, 1H), 2.85 – 2.76 (m, 1H), 1.71 – 1.66 (m, 2H), 1.65 – 1.60 (m, 2H), 1.51 – 1.47 (m, 1H), 1.28 – 1.19 (m, 2H), 1.13 – 1.03 (m, 3H) ppm.

^{13}C NMR (151 MHz, DMSO-d_6): δ 156.5, 140.3, 135.4, 127.9, 125.7, 124.3, 123.3, 123.1, 116.4, 115.1, 111.1, 56.3, 33.6, 25.5, 24.5 ppm.

HRMS (ESI-QTOF): *m/z* calculated for $C_{19}H_{22}N_3O^+$, 308.1757 $[M+H]^+$; found: 308.1760.

N-cyclohexyl-2-(4-(dimethylamino)phenyl)imidazo[1,2-*a*]pyridin-3-amine **(4k)**

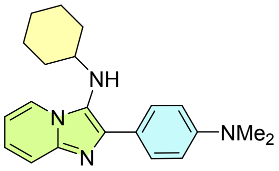

4k

4k was obtained from 2-aminopyridine (0.047 g; 0.50 mmol), 4-dimethylaminobenzaldehyde (0.075 g; 0.50 mmol), HPW (0.029 g; 2 mol%), cyclohexyl isocyanide (0.062 mL; 0.50 mmol) in EtOH (0.5 mL), in 45% yield as a white solid (m.p.: 180-181 ºC). $R_f = 0.40$ (30% EtOAc/Hexane).

FT-IR (ATR): 3272, 2919, 2852, 1613, 1551, 1514, 1440, 1393, 1366, 1338, 1229, 1196, 1108, 1065, 950, 810, 751, 734, 702, 601, 550 cm^{-1}.

^{1}H NMR (600 MHz, $CDCl_3$): δ 8.09 (dt, J = 6.8, 1.2 Hz, 1H), 7.97 – 7.91 (m, 2H), 7.52 (dt, J = 9.0, 1.2 Hz, 1H), 7.08 (ddd, J = 9.0, 6.8, 1.2 Hz, 1H), 6.84 – 6.79 (m, 2H), 6.74 (td, J = 6.8, 1.2 Hz, 1H), 3.07 (s, 1H), 3.01 (s, 6H), 3.00 – 2.96 (m, 1H), 1.86 – 1.79 (m, 2H), 1.73 – 1.66 (m, 2H), 1.60 – 1.55 (m, 1H), 1.31 – 1.10 (m, 5H) ppm.

^{13}C NMR (151 MHz, $CDCl_3$): δ 149.7, 141.4, 137.1, 127.9, 123.6, 123.3, 122.6, 122.5, 116.9, 112.4, 111.2, 56.9, 40.5, 34.2, 25.8, 24.9 ppm.

HRMS (ESI-QTOF): *m/z* calculated for $C_{21}H_{27}N_4^+$, 335.2230 $[M+H]^+$; found: 335.2235.

4-(6-chloro-3-(cyclohexylamino)imidazo[1,2-*a*]pyridin-2-yl)benzonitrile **(4p)**

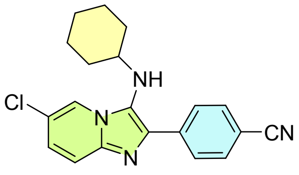

4p

4p was obtained from 2-amino-5-chloropyridine (0.064 g; 0.50 mmol), 4-cyanobenzaldehyde (0.066 g; 0.50 mmol), HPW (0.029 g; 2 mol%), cyclohexyl isocyanide (0.062 mL; 0.50 mmol) in EtOH (0.5 mL), in 55% yield as a green solid (m.p.: 250-252 ºC). $R_f = 0.45$ (20% EtOAc/Hexane).

FT-IR (ATR): 3297, 2932, 2852, 2220, 1606, 1576, 1502, 1411, 1368, 1325, 1219, 1100, 1077, 843, 804, 737, 710, 693, 550 cm^{-1}.

^{1}H NMR (600 MHz, DMSO-*d*$_6$): δ 8.43 (d, *J* = 2.0 Hz, 1H), 8.26 (d, *J* = 8.2 Hz, 2H), 7.77 (d, *J* = 8.2 Hz, 2H), 7.44 (d, *J* = 9.5 Hz, 1H), 7.14 (dd, *J* = 9.5, 2.0 Hz, 1H), 4.91 (d, *J* = 6.9 Hz, 1H), 2.73 – 2.66 (m, 1H), 1.66 – 1.56 (m, 2H), 1.55 – 1.49 (m, 2H), 1.21 – 1.10 (m, 2H), 1.04 – 0.92 (m, 3H), ppm.

^{13}C NMR (151 MHz, DMSO-*d*$_6$): δ 139.2, 138.9, 133.1, 132.4, 128.5, 126.7, 125.4, 121.3, 119.2, 119.1, 118.1, 109.0, 56.9, 33.5, 25.3, 24.6 ppm.

HRMS (ESI-QTOF): *m*/*z* calculated for $C_{20}H_{20}ClN_4^+$, 351.1371 $[M+H]^+$; found: 351.1372.

2-(4-(diethylamino)phenyl)-*N*-phenylimidazo[1,2-*a*]pyridin-3-amine **(4t)**

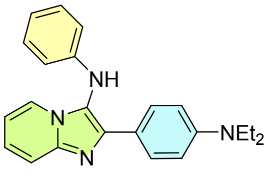

4t

4t was obtained from 2-aminopyridine (0.047 g; 0.50 mmol), 4-diethylaminobenzaldehyde (0.089 g; 0.50 mmol), HPW (0.029 g; 2 mol%), phenyl isocyanide (0.050 mL; 0.50 mmol) in EtOH (0.5 mL), in 63% yield as a white solid (m.p.: > 300 ºC). $R_f = 0.53$ (70% EtOAc/Hexane).

FT-IR (ATR): 3198, 3164, 3086, 2973, 2926, 1613, 1598, 1555, 1528, 1494, 1403, 1344, 1270, 1196, 1083, 1016, 812, 751, 734, 693, 505 cm^{-1}.

^{1}H NMR (600 MHz, $CDCl_3$): δ 7.89 – 7.83 (m, 2H), 7.79 (dt, *J* = 6.8, 1.2 Hz, 1H), 7.60 (dt, *J* = 9.0, 1.2 Hz, 1H), 7.22 – 7.18 (m, 2H), 7.16 (ddd, *J* = 9.0, 6.8, 1.2 Hz, 1H), 6.85 (tt, *J* = 7.2, 1.2 Hz, 1H), 6.72 (td, *J* = 6.8, 1.2 Hz, 1H), 6.69 – 6.63 (m, 2H), 6.62 – 6.56 (m, 2H), 5.60 (s, 1H), 3.35 (q, *J* = 7.2 Hz, 4H), 1.15 (t, *J* = 7.2 Hz, 6H) ppm.

^{13}C NMR (151 MHz, $CDCl_3$): δ 147.5, 145.0, 142.5, 129.8, 128.2, 124.5, 122.5, 119.6, 117.0, 116.3, 113.4, 111.8, 111.6, 44.3, 12.7 ppm.

HRMS (ESI-QTOF): *m*/*z* calculated for $C_{23}H_{25}N_4^+$, 357.2074 $[M+H]^+$; found: 357.2075.

2-(4-(diethylamino)phenyl)-*N*-(4-methoxyphenyl)imidazo[1,2-*a*]pyridin-3-amine **(4u)**

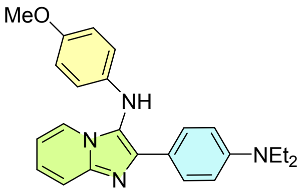

4u

4u was obtained from 2-aminopyridine (0.047 g; 0.50 mmol), 4-diethylaminobenzaldehyde (0.089 g; 0.50 mmol), HPW (0.029 g; 2 mol%), 4-methoxyphenyl isocyanide (0.058 mL; 0.50 mmol) in EtOH (0.5 mL), in 53% yield as a white solid (m.p.: > 300 ºC). R_f = 0.50 (70% EtOAc/Hexane).

FT-IR (ATR): 3153, 3075, 2967, 2926, 2889, 2825, 1613, 1502, 1401, 1372, 1342, 1266, 1233, 1192, 1079, 1036, 814, 753, 734, 669, 572, 515 cm^{-1}.

^{1}H NMR (600 MHz, DMSO-*d*$_6$): δ 7.89 – 7.83 (m, 3H), 7.77 (s, 1H), 7.52 (dt, *J* = 9.0, 1.2 Hz, 1H), 7.22 (ddd, *J* = 9.0, 6.7, 1.2 Hz, 1H), 6.84 (td, *J* = 6.7, 1.2 Hz, 1H), 6.77 – 6.72 (m, 2H), 6.67 – 6.62 (m, 2H), 6.45 – 6.39 (m, 2H), 3.62 (s, 3H), 3.33 (q, *J* = 7.0 Hz, 5H), 1.07 (t, *J* = 7.0 Hz, 6H) ppm.

^{13}C NMR (151 MHz, DMSO-*d*$_6$): δ 152.2, 146.8, 141.5, 139.6, 138.5, 127.7, 124.2, 122.6, 120.6, 117.7, 116.5, 115.0, 113.7, 111.6, 111.2, 55.3, 43.6, 12.5 ppm.

HRMS (ESI-QTOF): *m*/*z* calculated for $C_{24}H_{27}N_4O^+$, 387.2179 [M+H]$^+$; found: 387.2182.

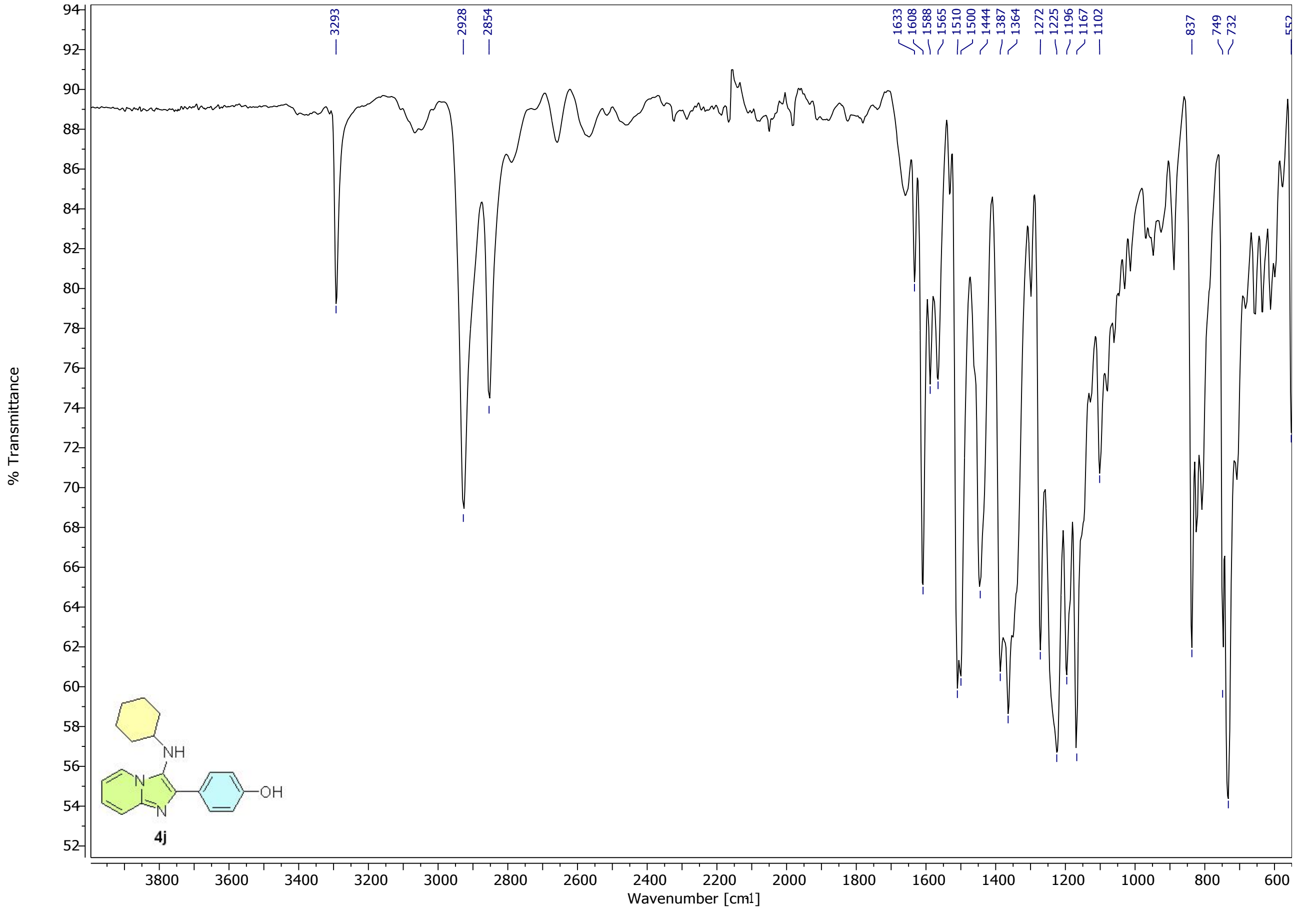

Figure S1. FT-IR (ATR) of compound **4j**.

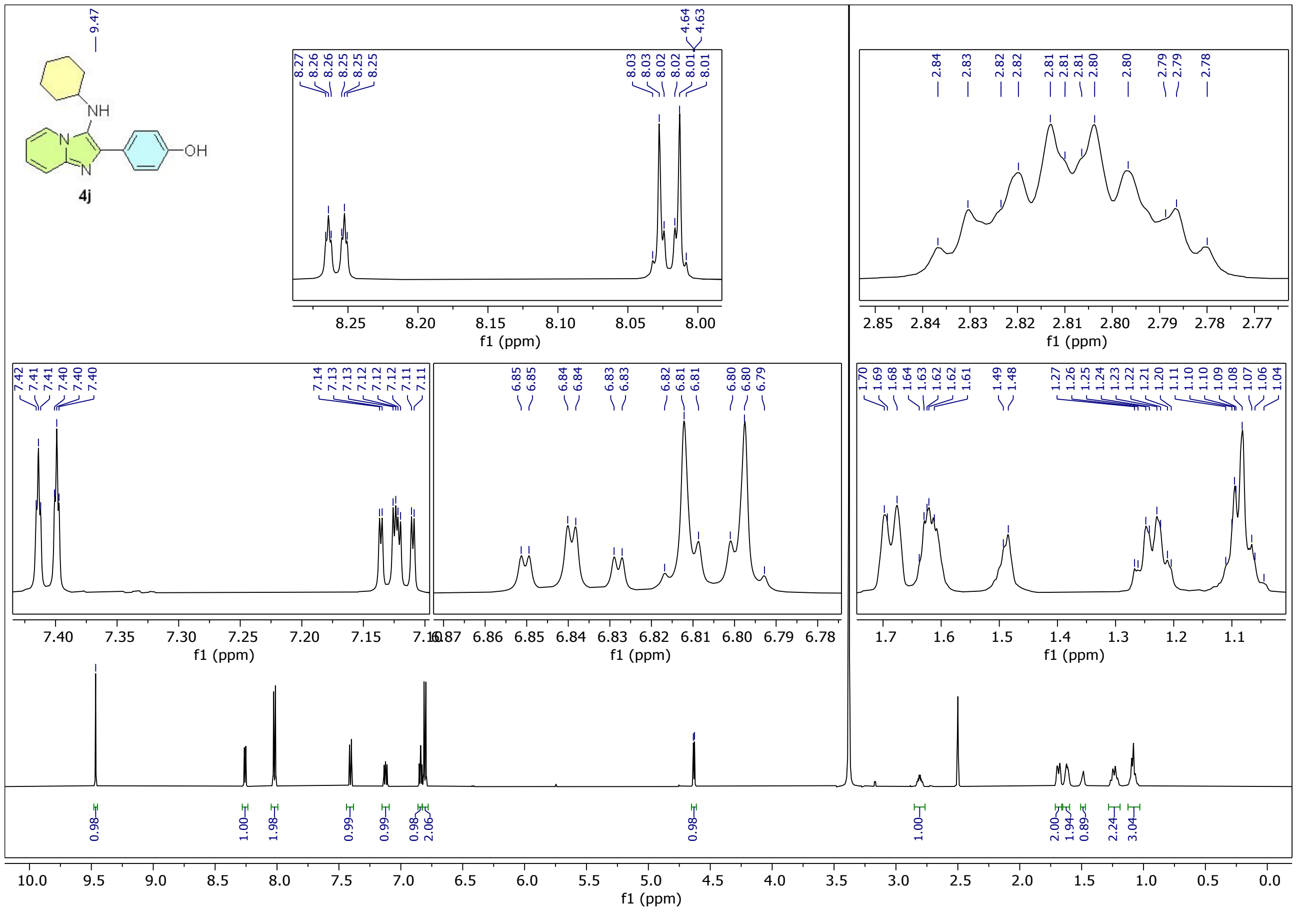

Figure S2. ^{1}H NMR spectrum (600 MHz, DMSO-d_6) of compound **4j**.

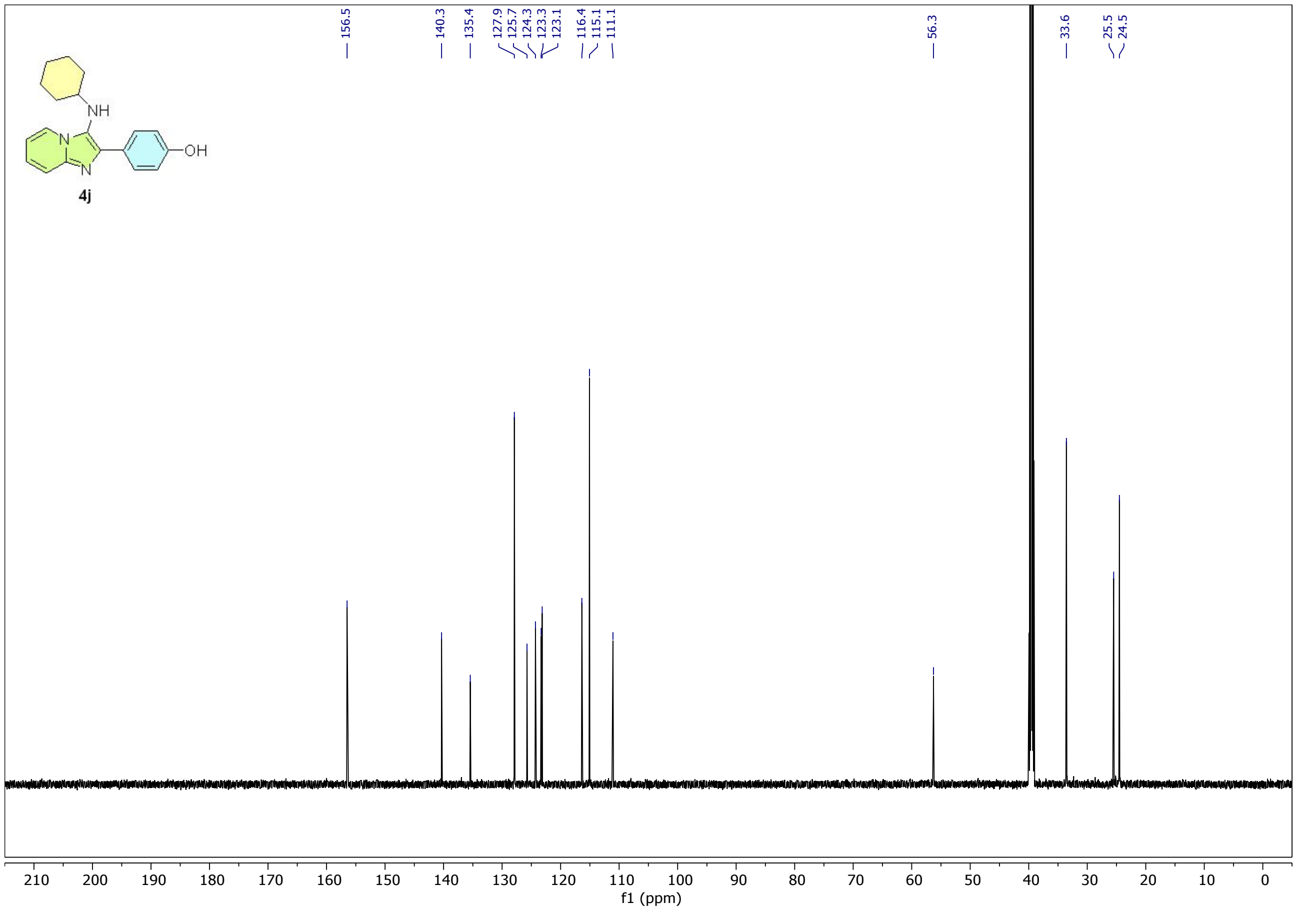

Figure S3. ^{13}C NMR spectrum (151 MHz, DMSO-d_6) of compound **4j**.

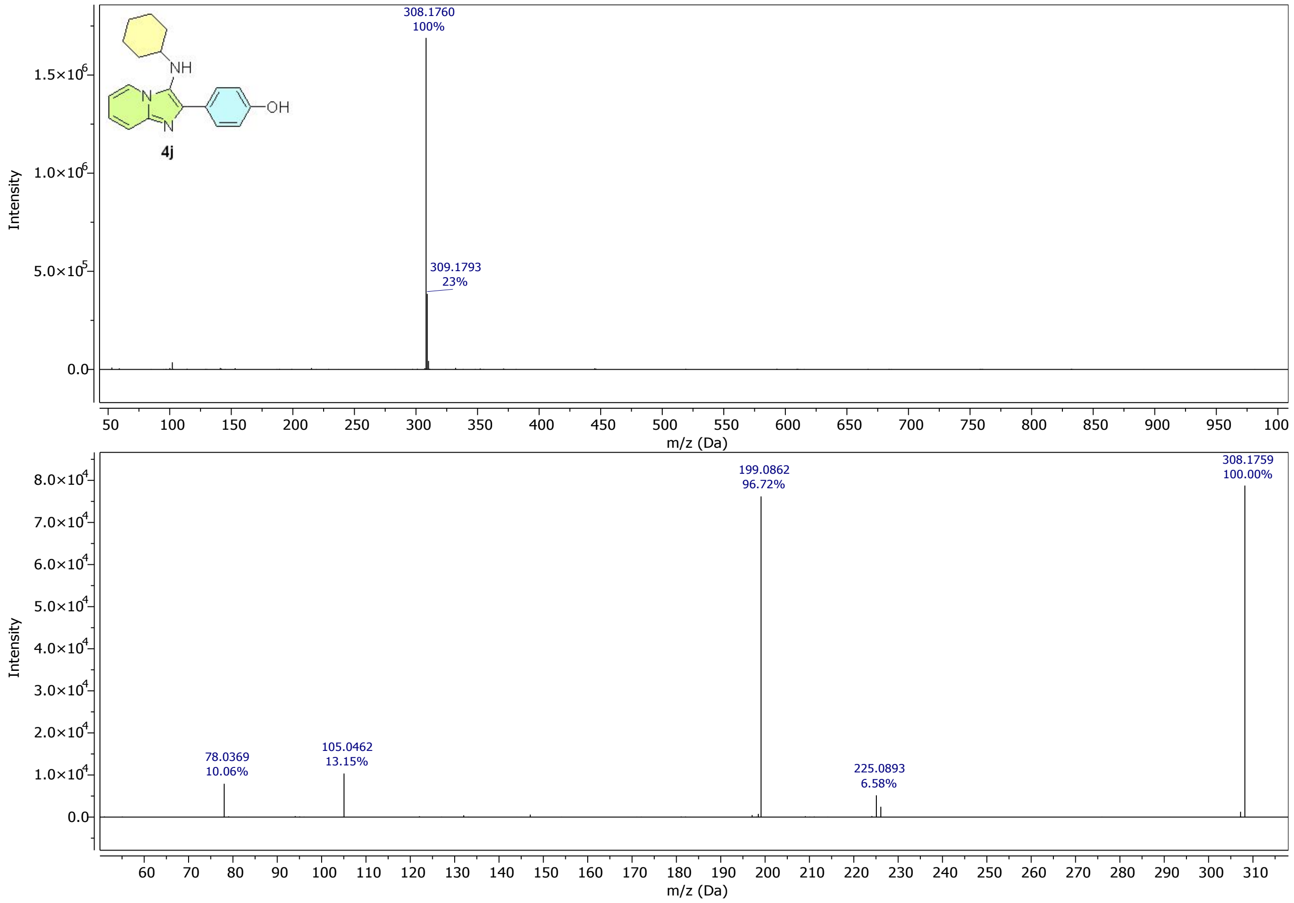

Figure S4. HRMS (ESI-QTOF) of compound **4j** and HRMS/MS for $[M+H]^+$.

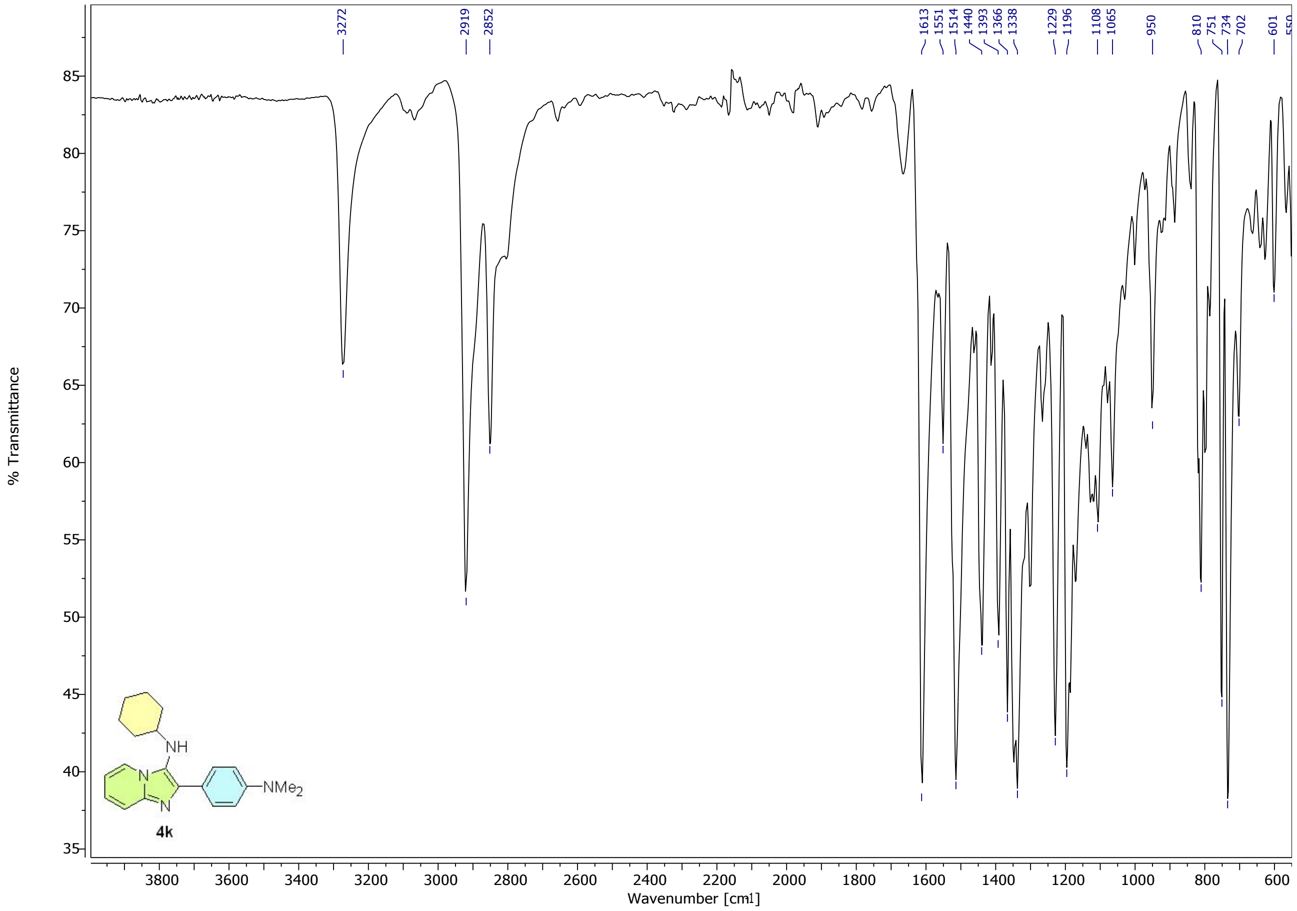

Figure S5. FT-IR (ATR) of compound **4k**.

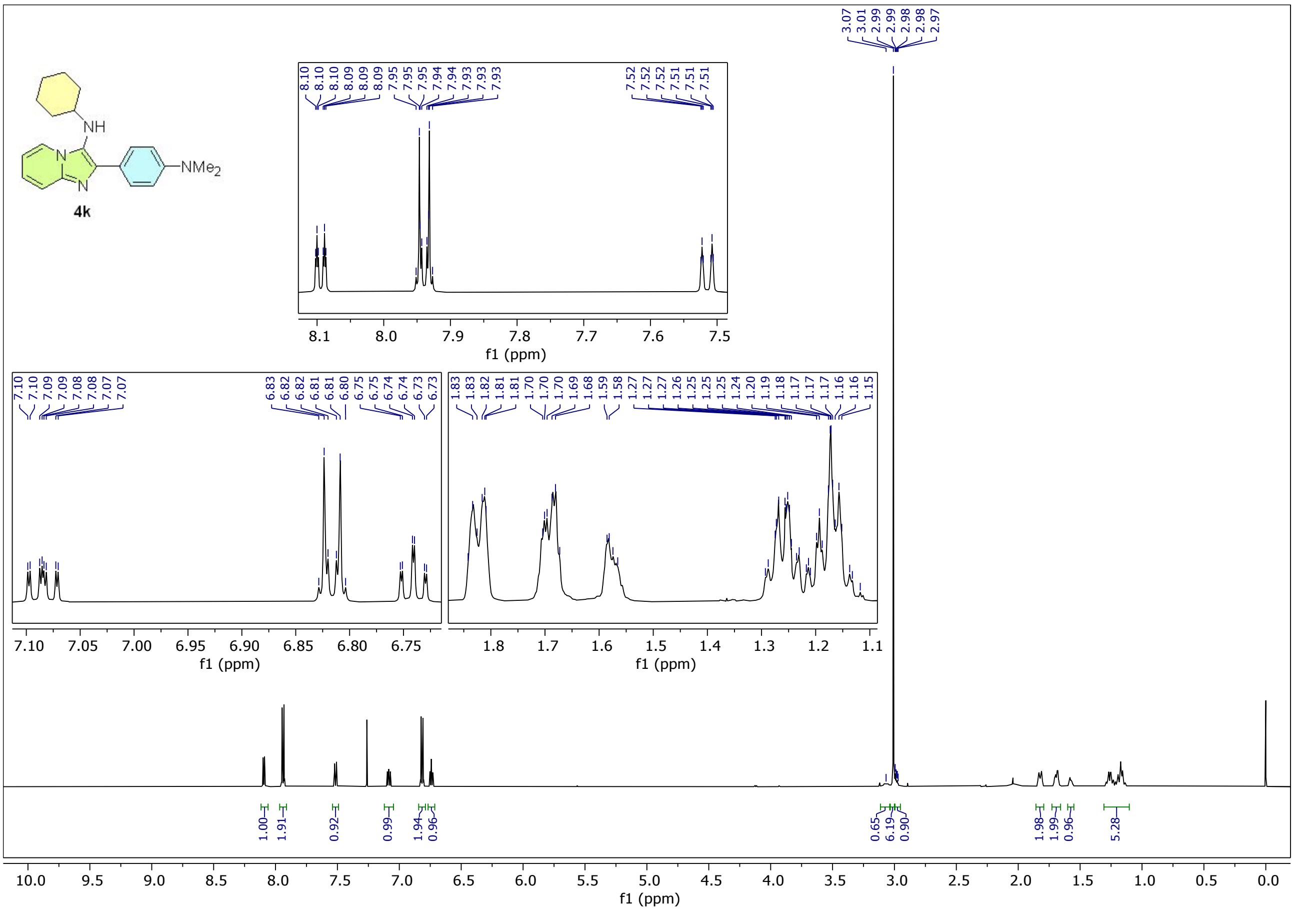

Figure S6. ^{1}H NMR spectrum (600 MHz, $CDCl_3$) of compound **4k**.

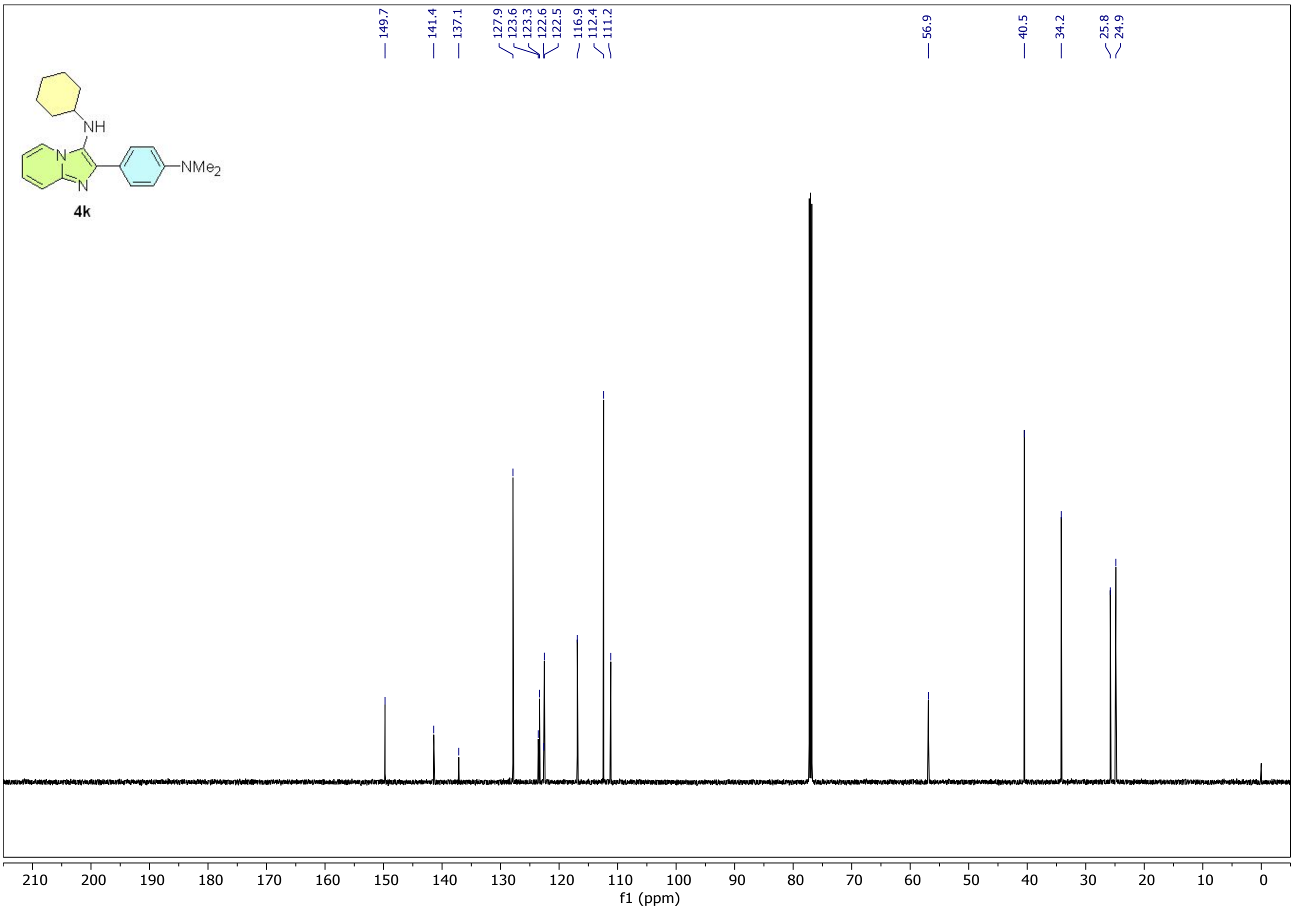

Figure S7. ^{13}C NMR spectrum (151 MHz, $CDCl_3$) of compound **4k**.

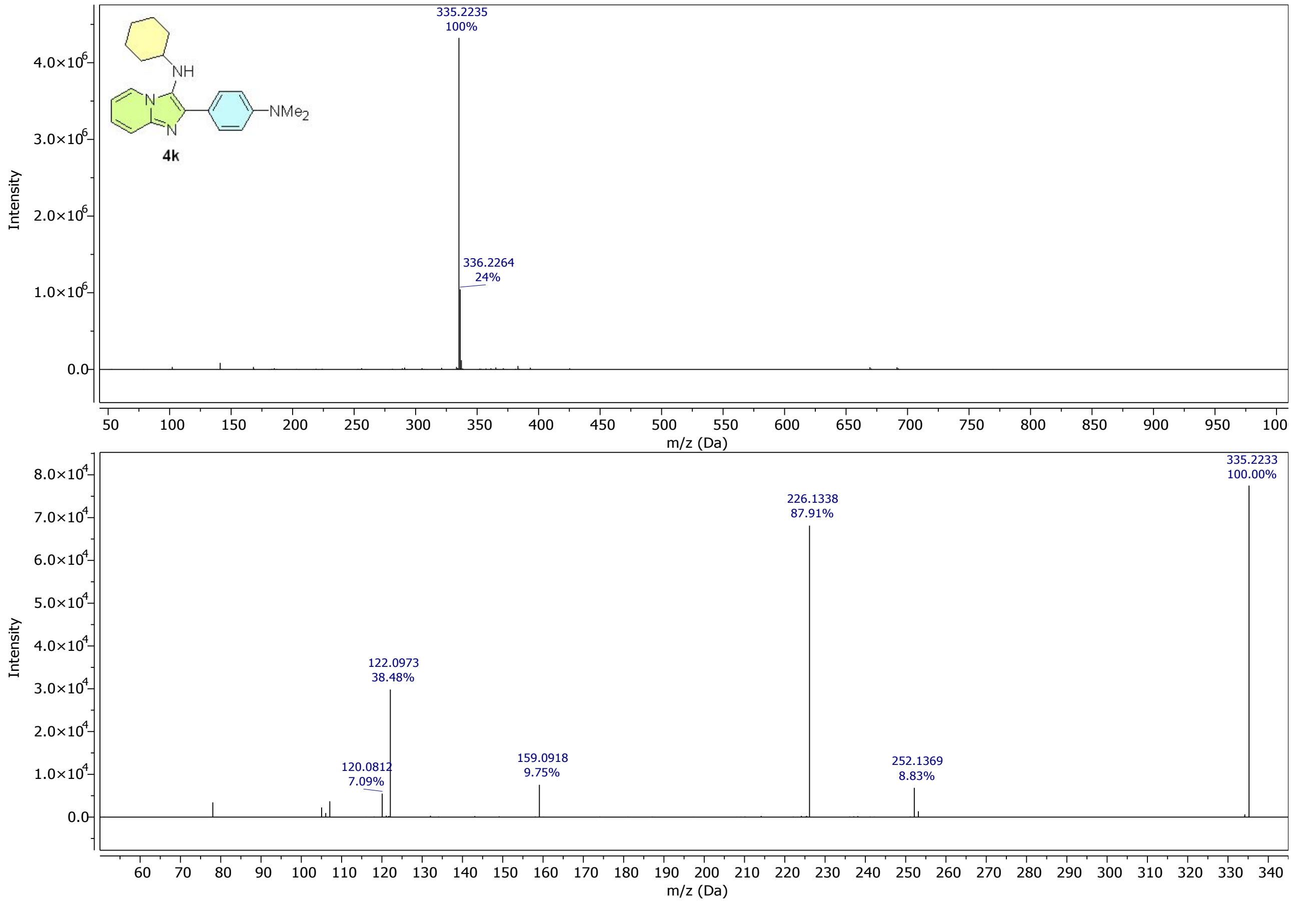

Figure S8. HRMS (ESI-QTOF) of compound **4k** and HRMS/MS for $[M+H]^+$.

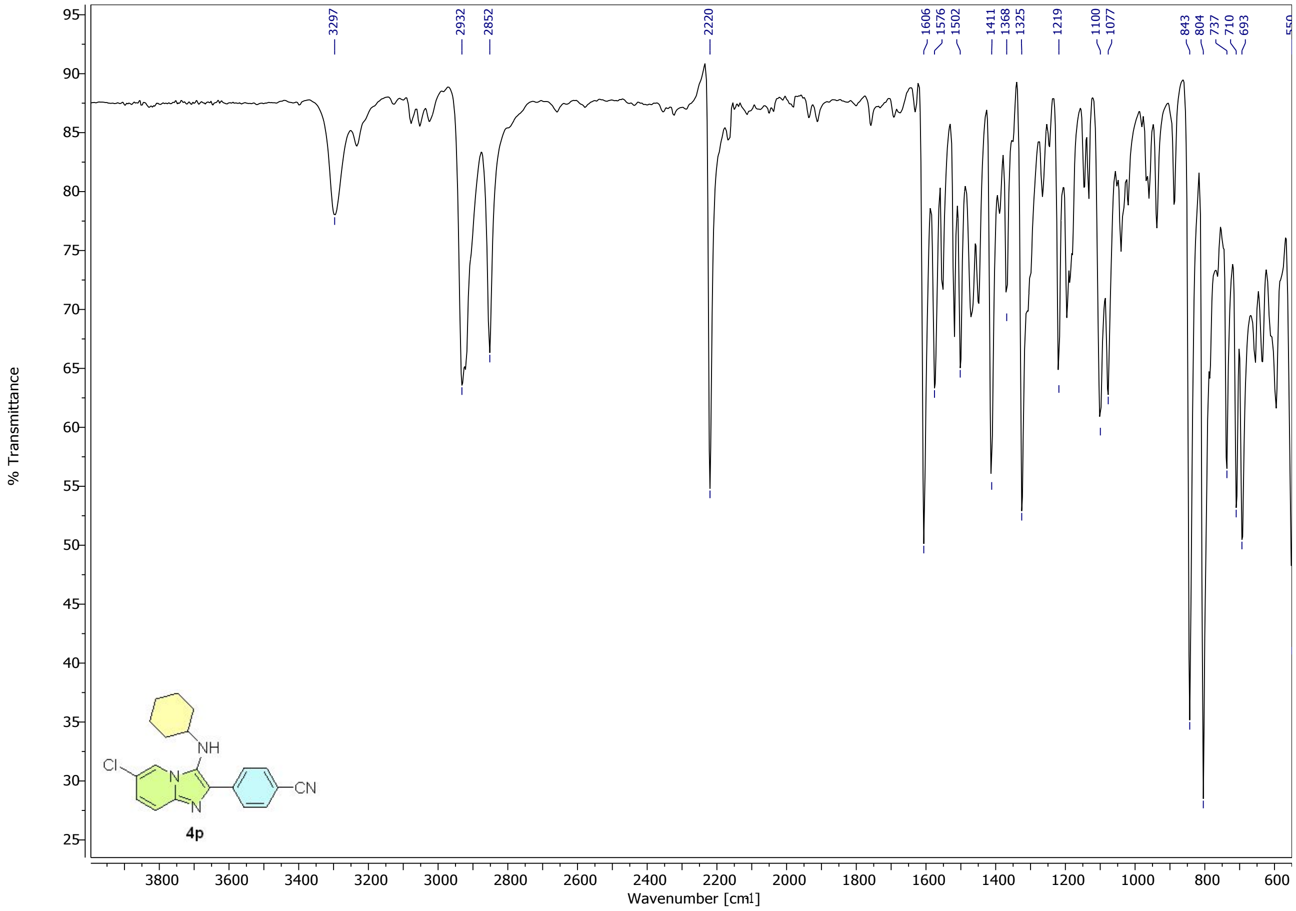

Figure S9. FT-IR (ATR) of compound **4p**.

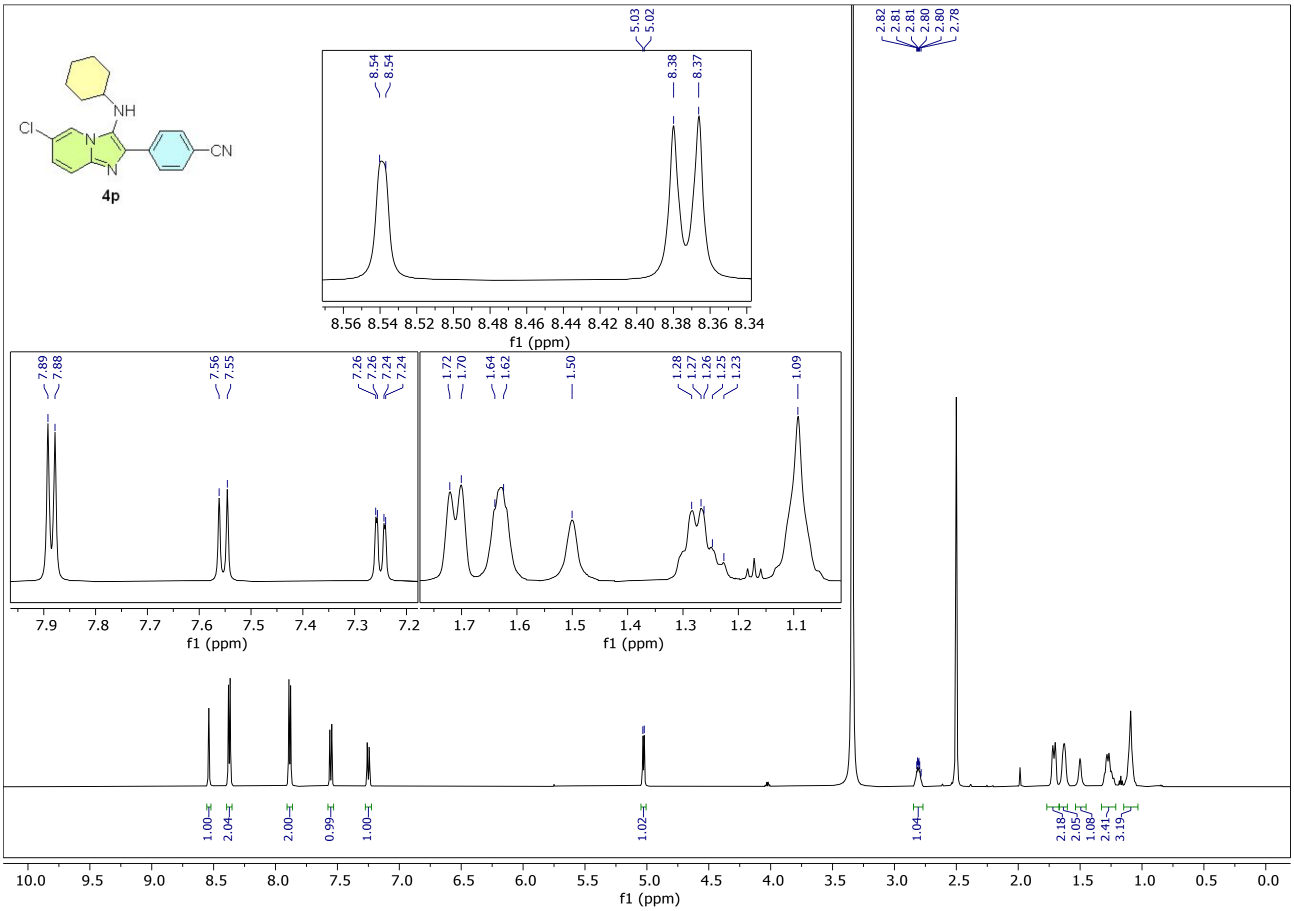

Figure S10. ^{1}H NMR spectrum (600 MHz, DMSO-d_6) of compound **4p**.

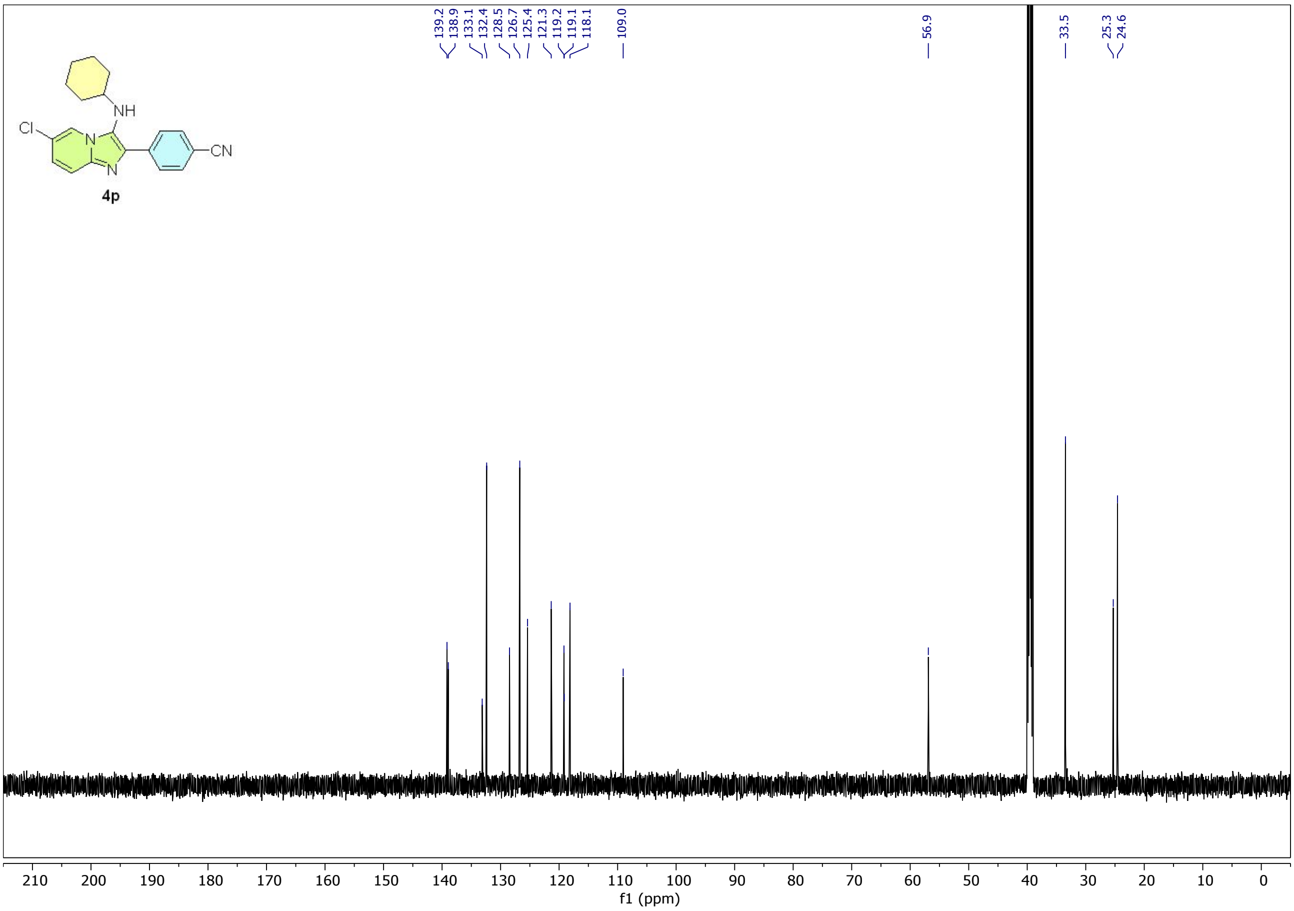

Figure S11. ^{13}C NMR spectrum (151 MHz, DMSO-d_6) of compound **4p**.

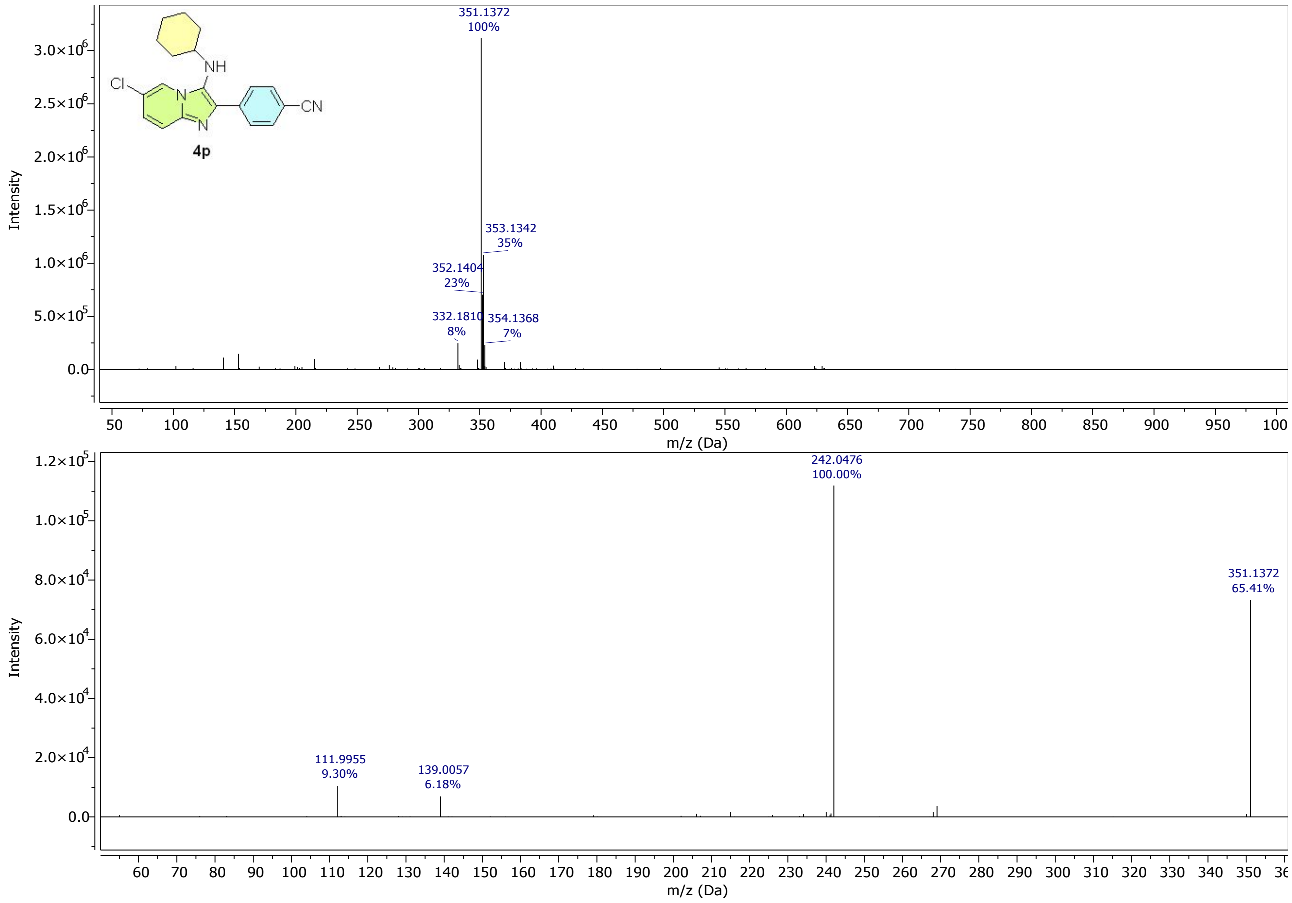

Figure S12. HRMS (ESI-QTOF) of compound **4p** and HRMS/MS for $[M+H]^+$.

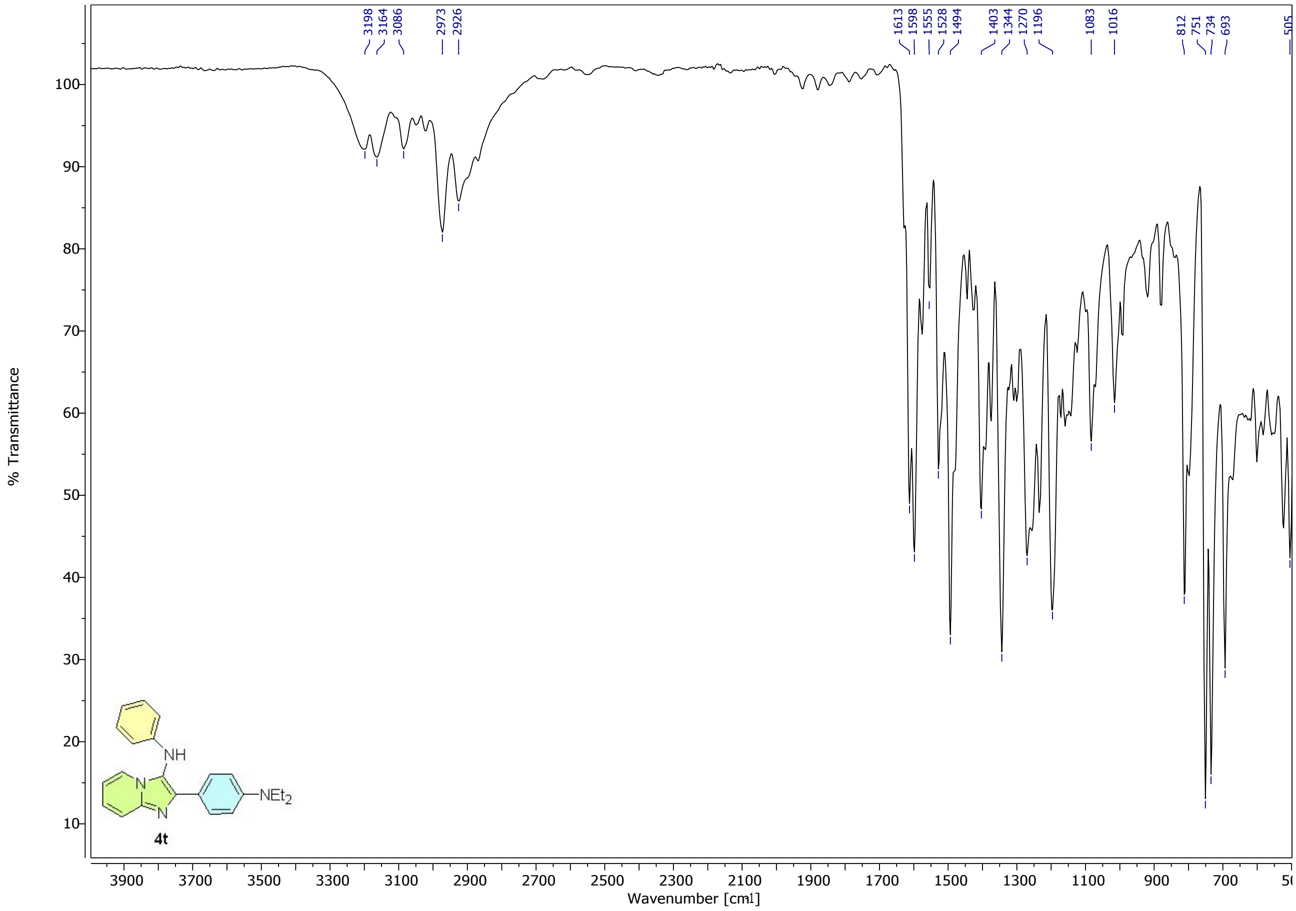

Figure S13. FT-IR (ATR) of compound **4t**.

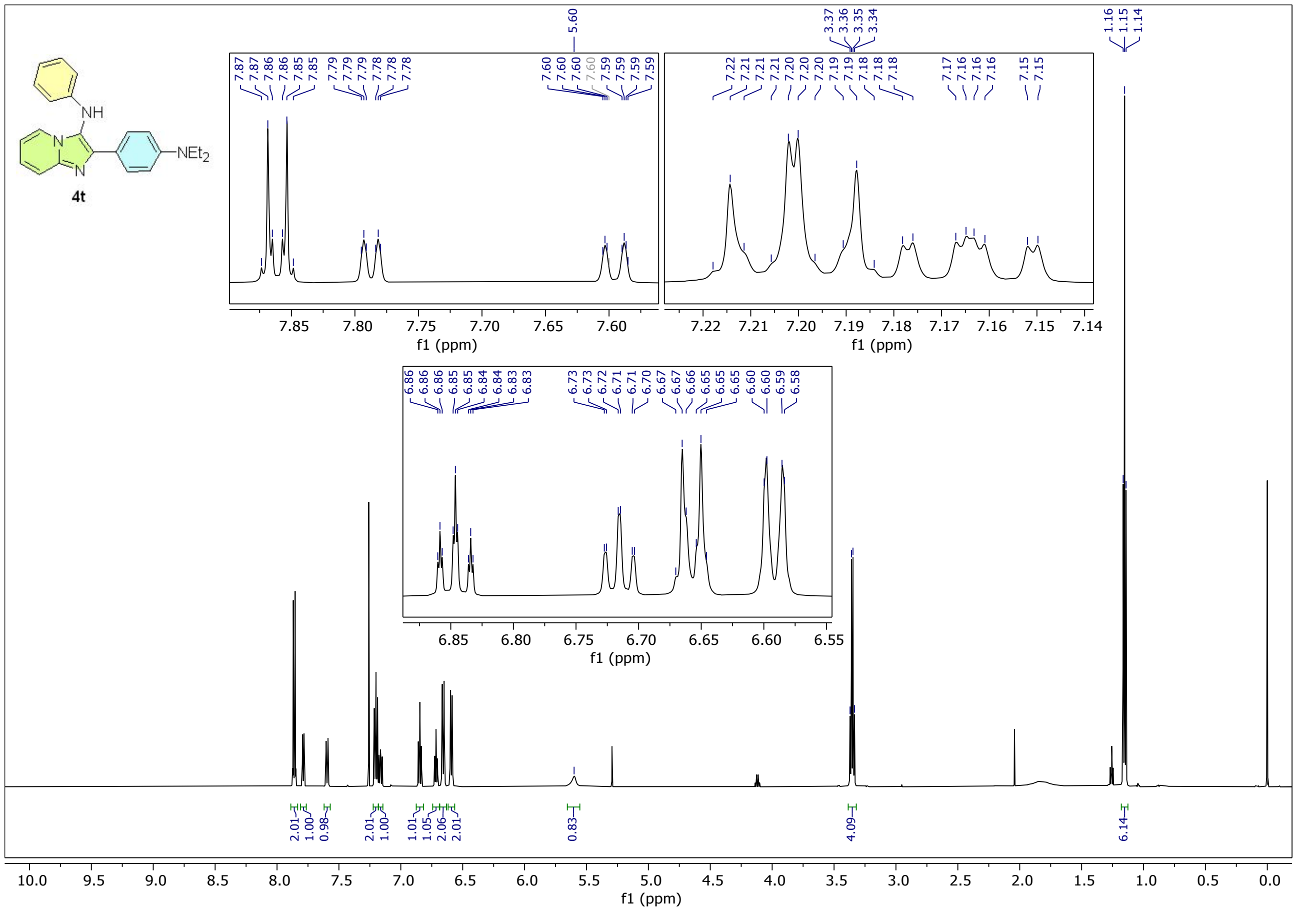

Figure S14. ^{1}H NMR spectrum (600 MHz, $CDCl_3$) of compound **4t**.

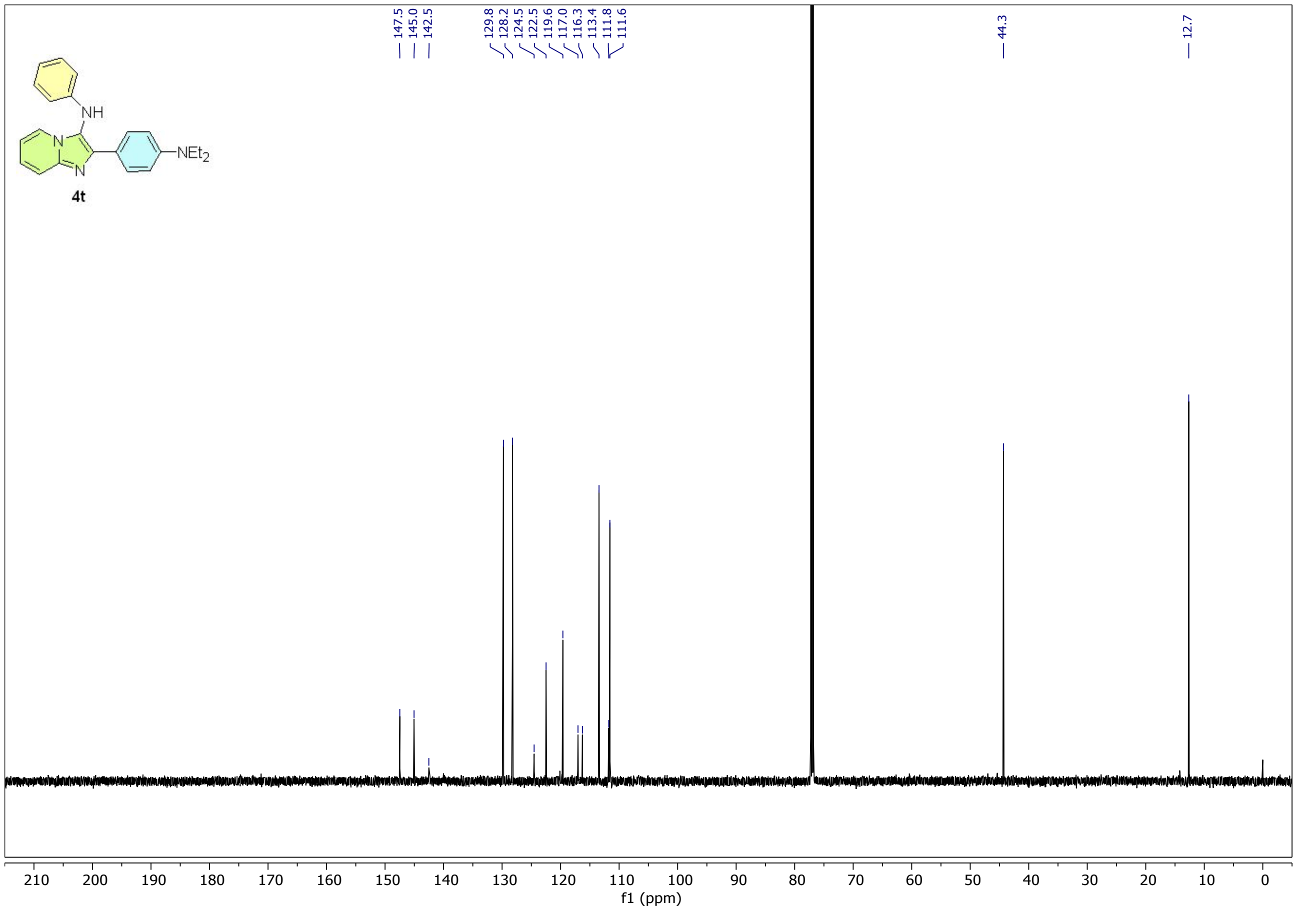

Figure S15. ^{13}C NMR spectrum (151 MHz, $CDCl_3$) of compound **4t**.

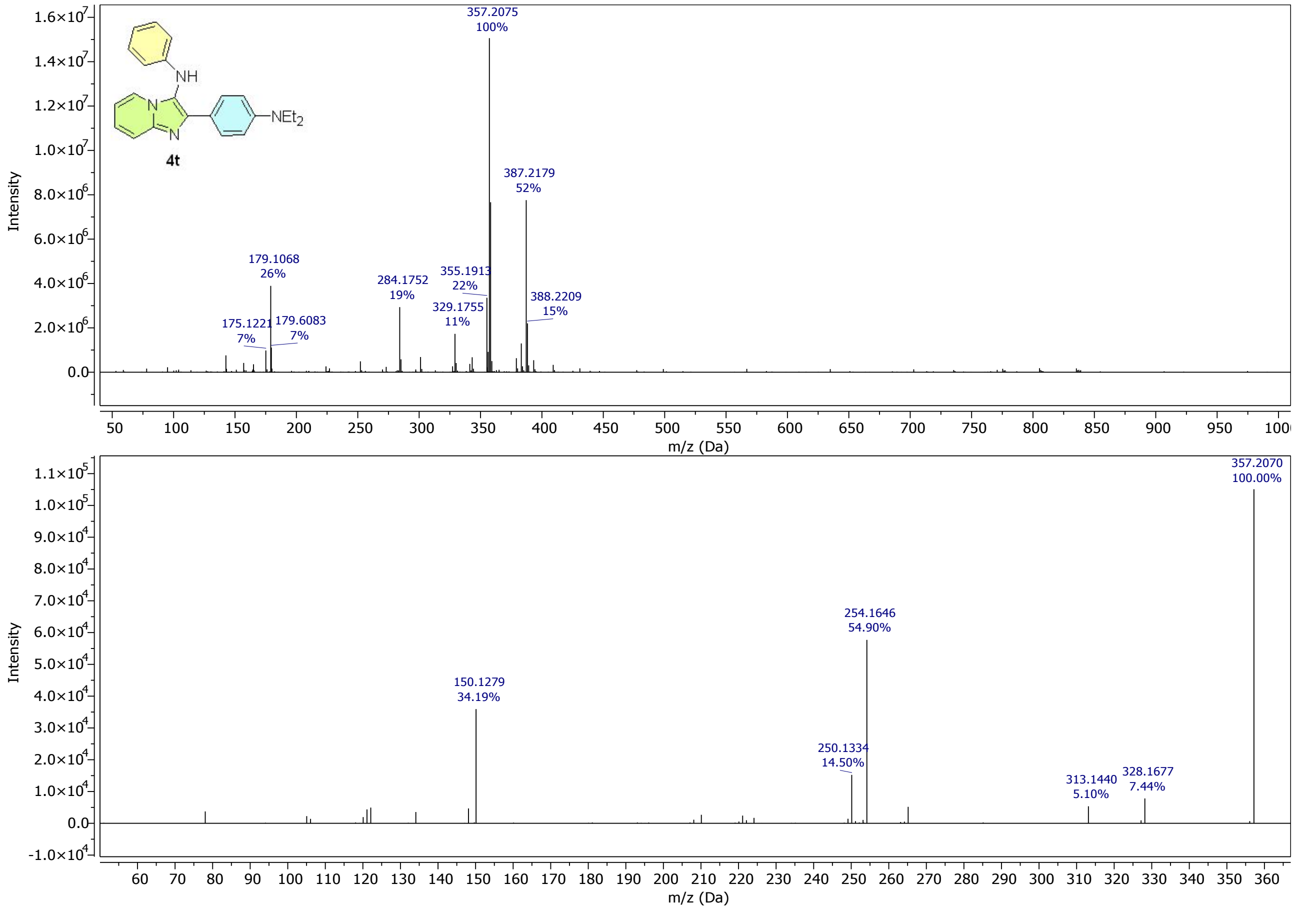

Figure S16. HRMS (ESI-QTOF) of compound **4t** and HRMS/MS for $[M+H]^+$.

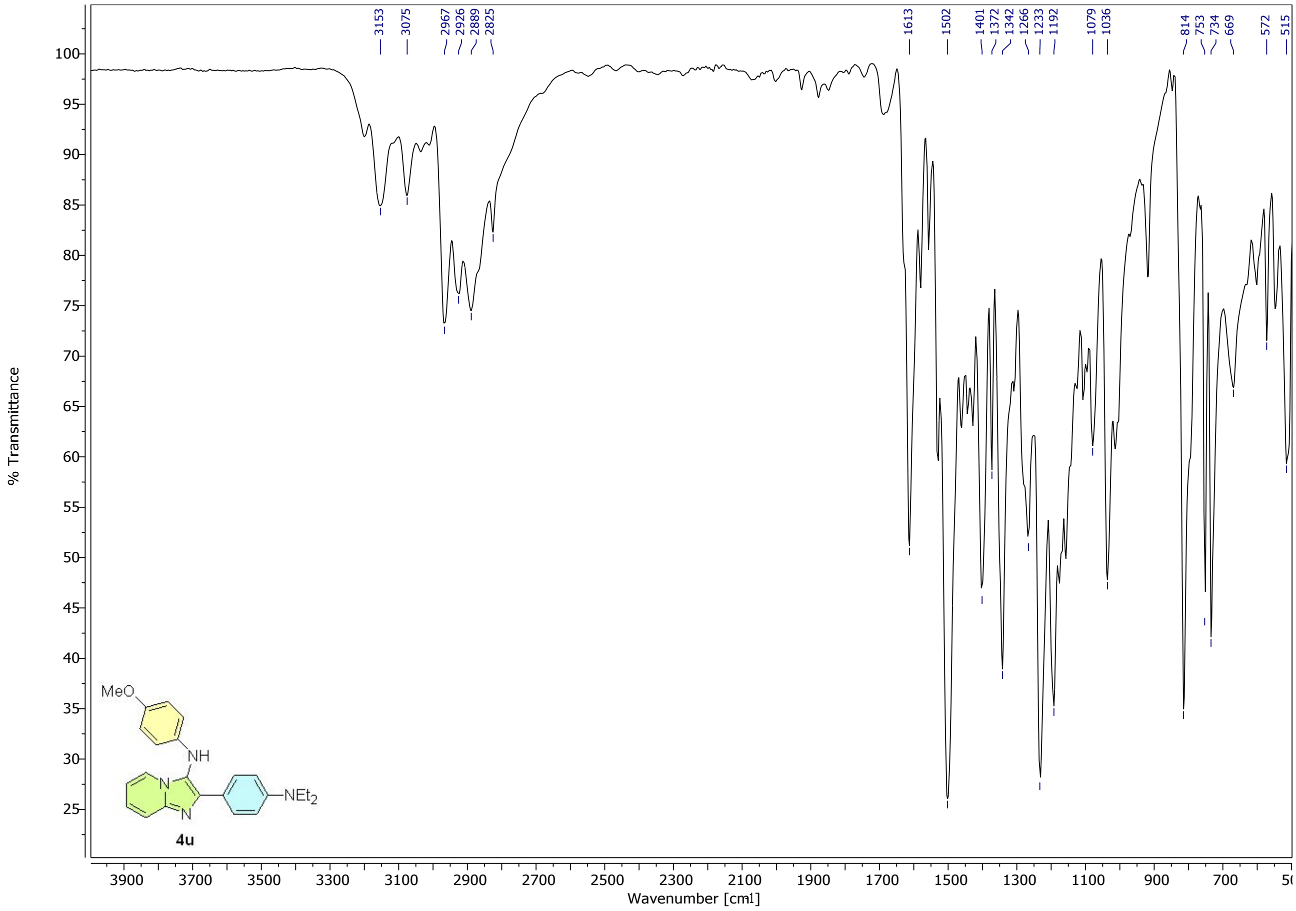

Figure S17. FT-IR (ATR) of compound **4u**.

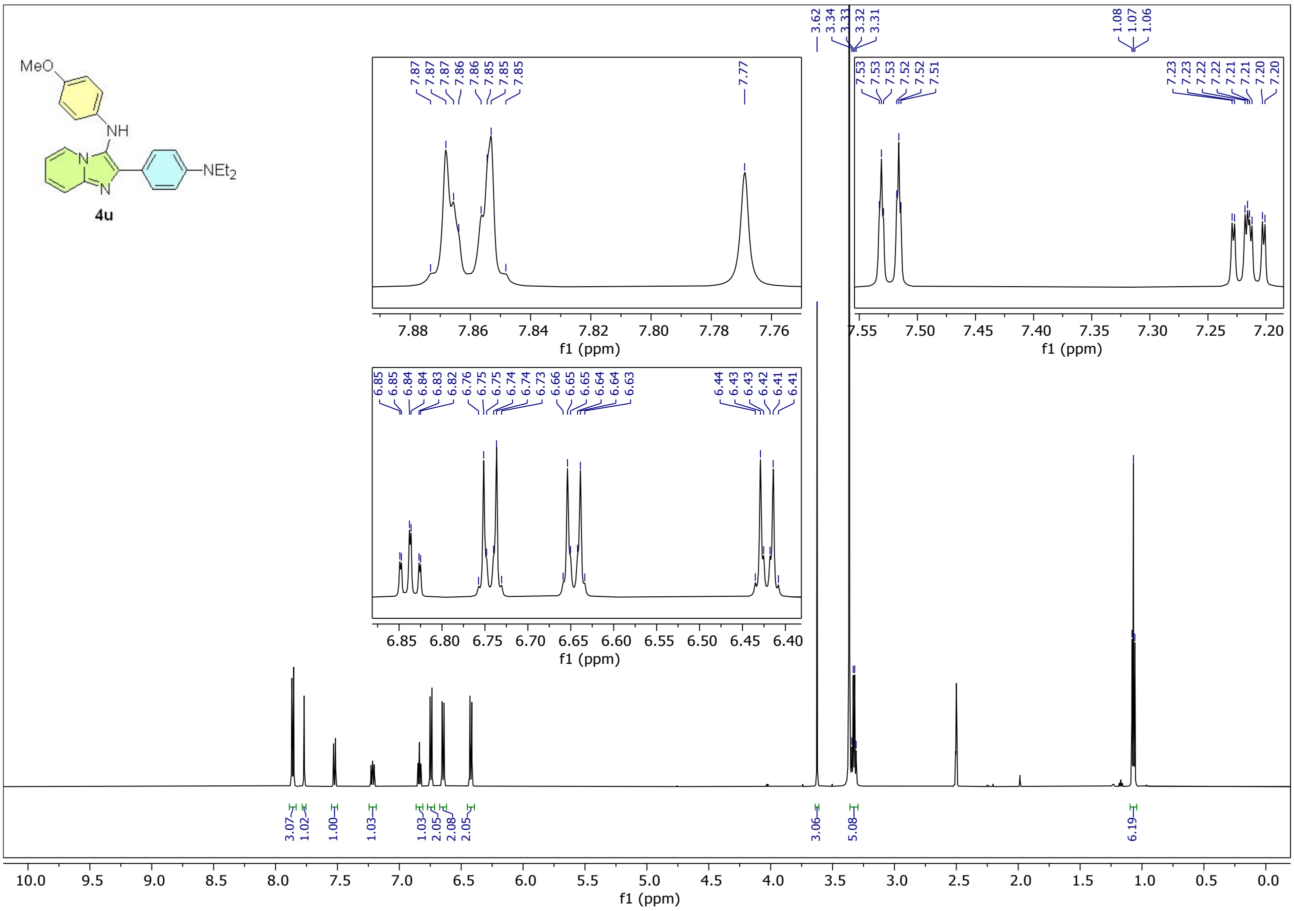

Figure S18. ^{1}H NMR spectrum (600 MHz, DMSO-d_6) of compound **4u**.

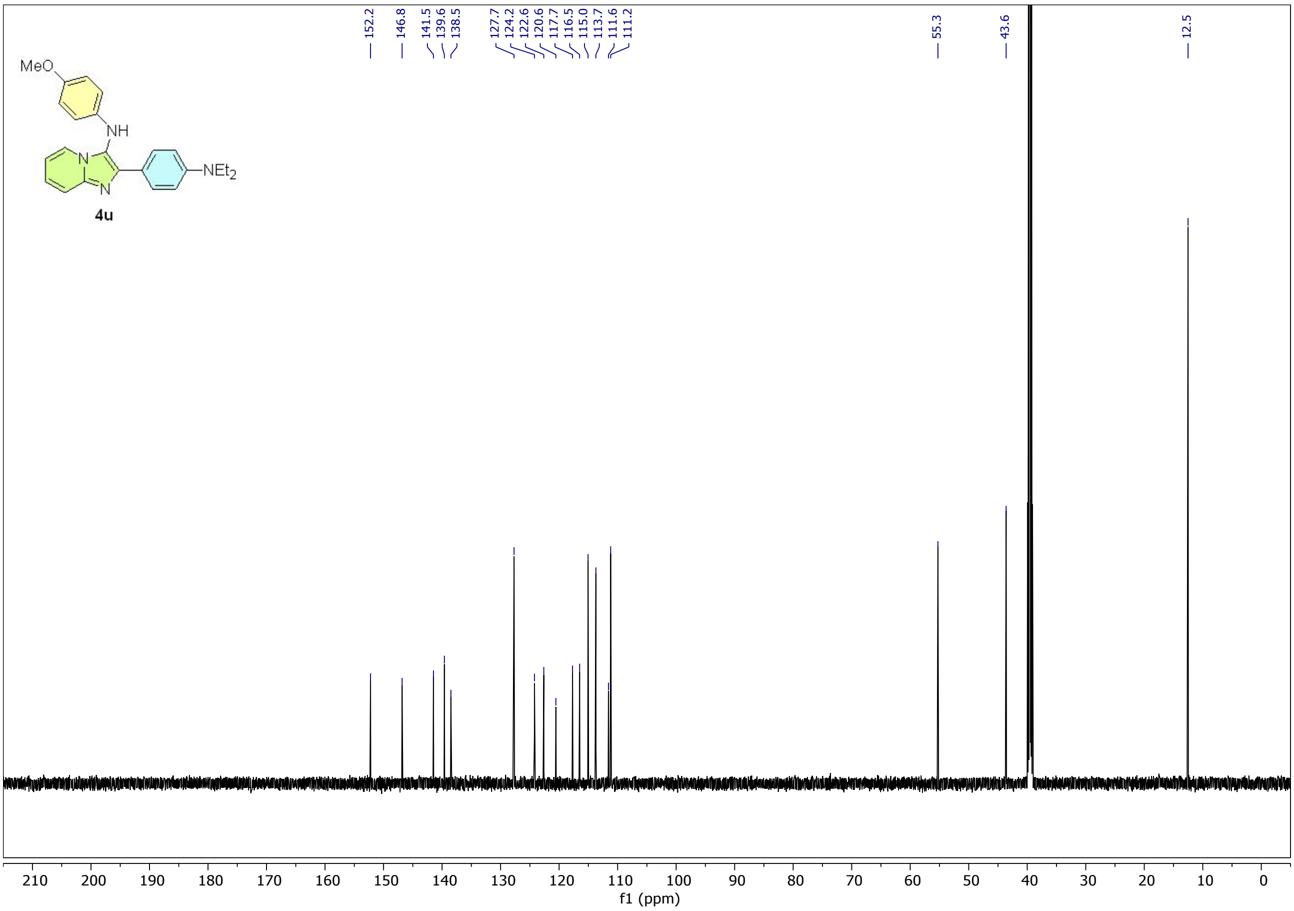

Figure S19. ^{13}C NMR spectrum (151 MHz, DMSO-d_6) of compound **4u**.

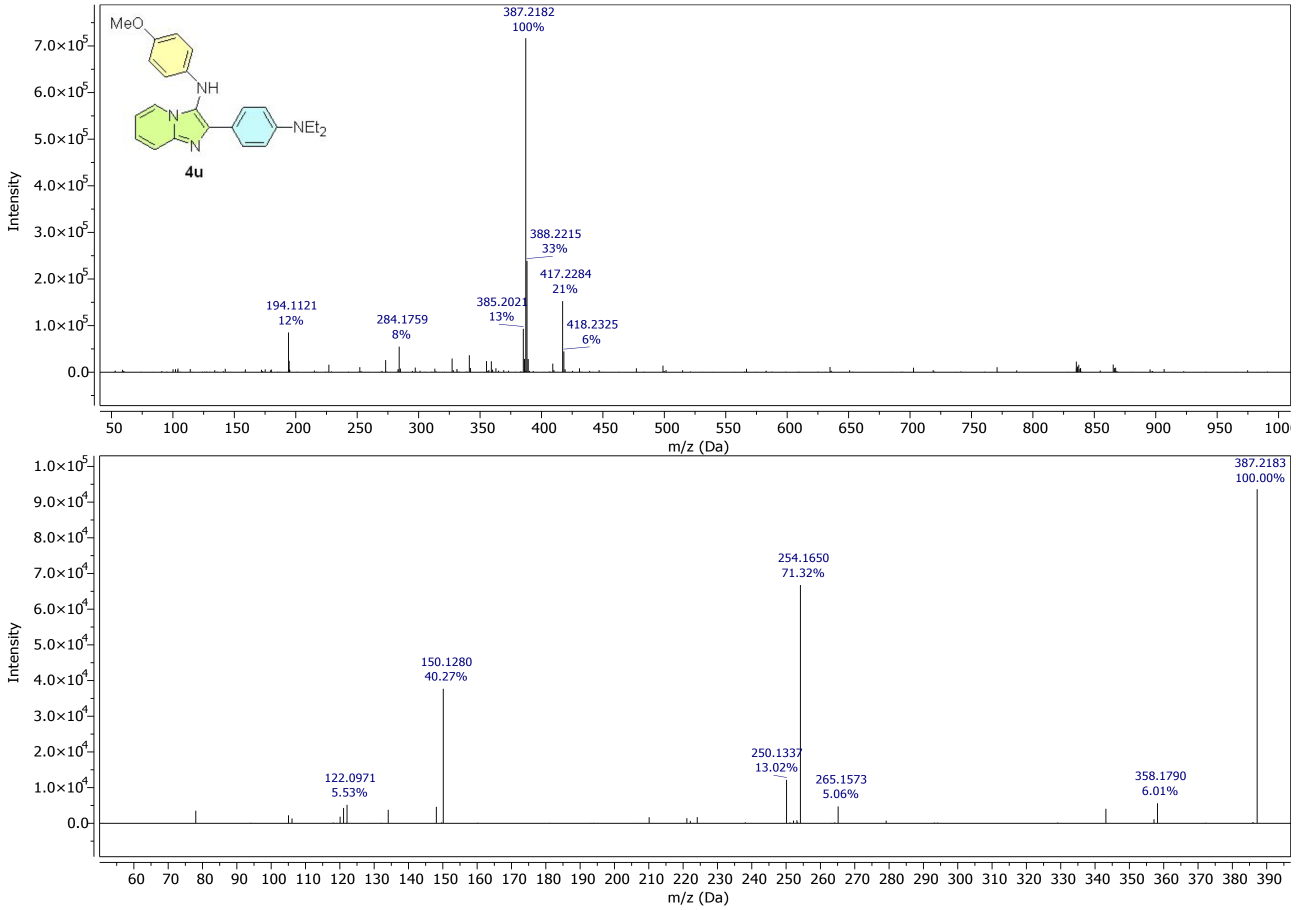

Figure S20. HRMS (ESI-QTOF) of compound **4u** and HRMS/MS for $[M+H]^+$.

3. Crystal structure determination of compound 4r

To better understand the fluorescence processes, a final spectroscopic analysis was conducted using X-ray diffraction to verify the spatial arrangement of atoms. During the crystallization attempts, only the crystals of product **4r** were successfully obtained and analyzed. Compound **4r** provided suitable crystals for X-ray structural analysis resulting in a novel crystal structure (Figure S21). Single crystal X-ray diffraction analysis demonstrated that the compound crystallizes in the monoclinic crystal system with four molecules in the asymmetric unit. The observed bond distance for N2≡C15 is 1.137(3) Å and bond angle N2-C15-C12 of 178.9(3)°, while the bond distance C11-C19 is 1.733(3) Å (Table S2). This compound presents intermolecular hydrogen bonds between N1-H1···N3' [d(H···O) = 2.45(3) Å, d(N···O) = 3.239(3) Å, \(N1-H1···N3) = 163(2)°, symmetry operation ('): x-1, y, z], forming a one-dimensional chain, as represented in Figure S22. For molecule **4r**, the analysis confirmed planarity along the heterocyclic chain, with the cyclohexyl group positioned out of the plane (Figure S23 and Figure S24).

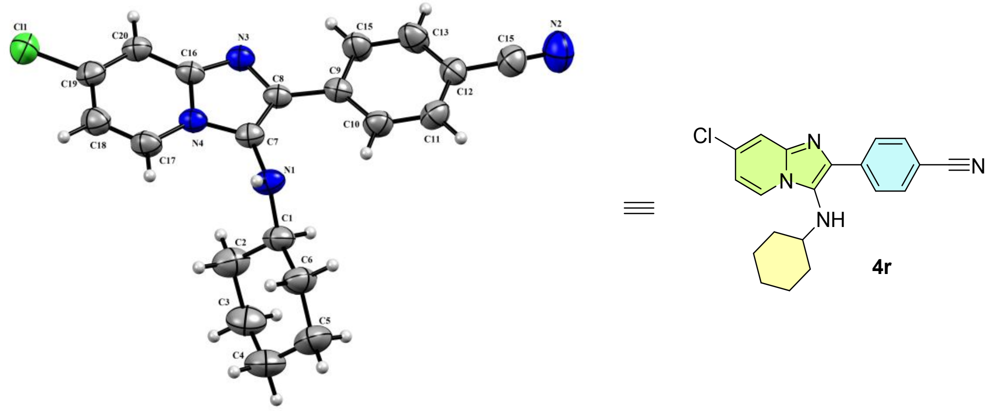

Figure S21. Molecular structure of **4r** with crystallographic labeling (50% probability displacement).

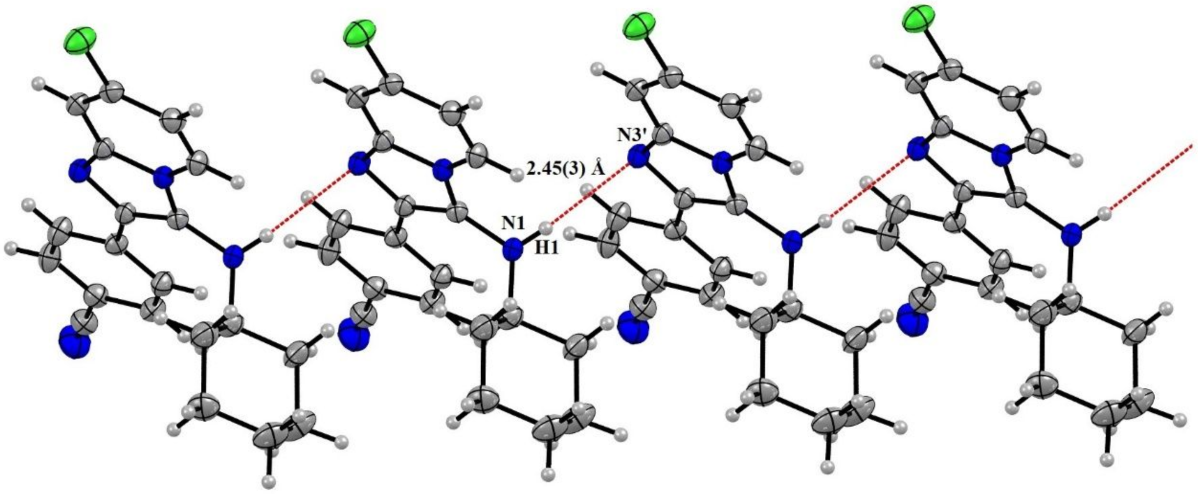

Figure S22. Compound **4r** shows the intermolecular hydrogen bond (dotted lines) forming a 1D chain. Symmetry operation ('): x-1, y, z.

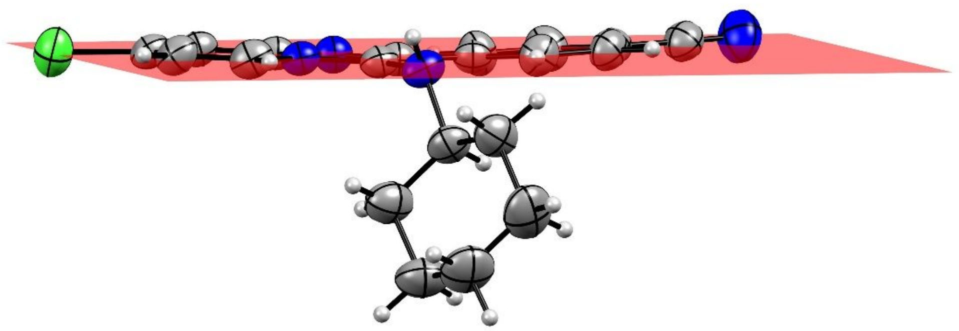

Figure S23. Perspective view of the almost planar structure of **4r**.

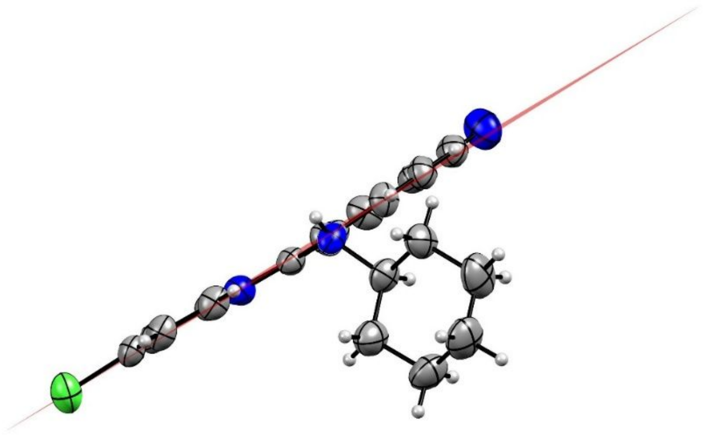

Figure S24. Another perspective view of the almost planar structure of **4r**.

Table S1. X-ray diffraction data collection and refinement parameters for **4r**.

	**4r**
Chemical formula	$C_{20}H_{19}N_4Cl$
M (g·mol^{-1})	350.84
Crystal system	Monoclinic
Space group	*P2$_1$/c*
Unit cell	
a (Å)	5.766(12)
b (Å)	10.533(2)
c (Å)	28.763(6)
b	92.781(5)
V (Å^3)	1744.7(6)
Z	4
Dc /g cm^{-3}	1.336
Index ranges	$-6 \leq h \leq 6$
	$-12 \leq k \leq 12$
	$-34 \leq l \leq 34$
Absorption coefficient /mm^{-1}	0.229
Absorption correction	multi-scan
Max/min transmission	0.94 / 0.87
Measured reflections	16818
Independent reflections / Rint	3204 / 0.056
Refined parameters	231
R1 (F) / wR2 (F^2) (I > 2s(I))	0.048 / 0.127
GooF	1.081
Largest diff. peak and hole (eÅ^{-3})	0.416 and -0.237
Deposit number CCDC	2409958

Table S2. Selected bond distances (Å) and bond angles (°) for **4r**.

**Bond distances (Å)**		**Bond angles (º)**	
Cl(1)-C(19)	1.733(3)	C(20)-C(19)-Cl(1)	120.2(2)
N(2)-C(15)	1.137(3)	C(18)-C(19)-Cl(1)	118.2(2)
N(1)-C(7)	1.390(3)	N(3)-C(8)-C(9)	120.3(2)
N(1)-C(1)	1.474(3)	C(8)-C(7)-N(1)	131.9(2)
N(3)-C(16)	1.321(3)	C(8)-C(7)-N(4)	105.0(2)
N(3)-C(8)	1.385(3)	N(1)-C(7)-N(4)	123.1(2)
N(4)-C(17)	1.368(3)	N(1)-C(1)-C(2)	115.6(2)
N(4)-C(16)	1.393(3)	N(1)-C(1)-C(6)	108.3(2)
N(4)-C(7)	1.394(3)	C(2)-C(1)-C(6)	111.9(2)
C(15)-C(12)	1.434(4)	N(2)-C(15)-C(12)	178.9(3)
C(12)-C(13)	1.380(4)	C(7)-C(8)-N(3)	111.2(2)
C(12)-C(11)	1.392(3)	C(8)-C(7)-N(1)	131.9(2)
C(13)-C(14)	1.379(4)	C(8)-C(7)-N(4)	105.0(2)

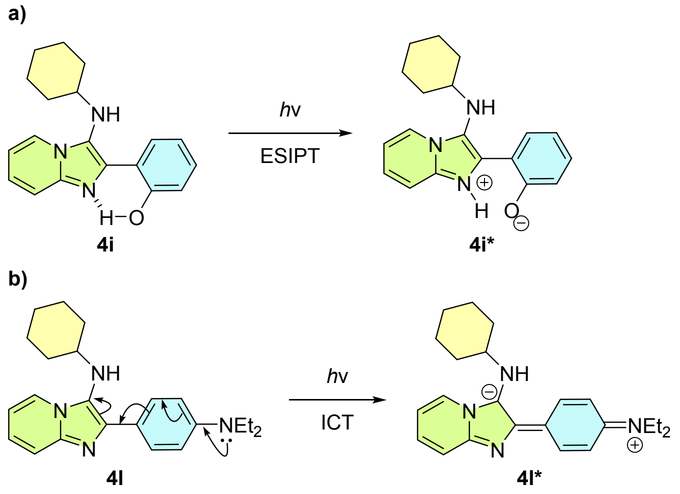

Scheme S2. Proposed **a)** ESIPT and **b)** ICT-type mechanism for the fluorescence process.

4. Solvatochromic study

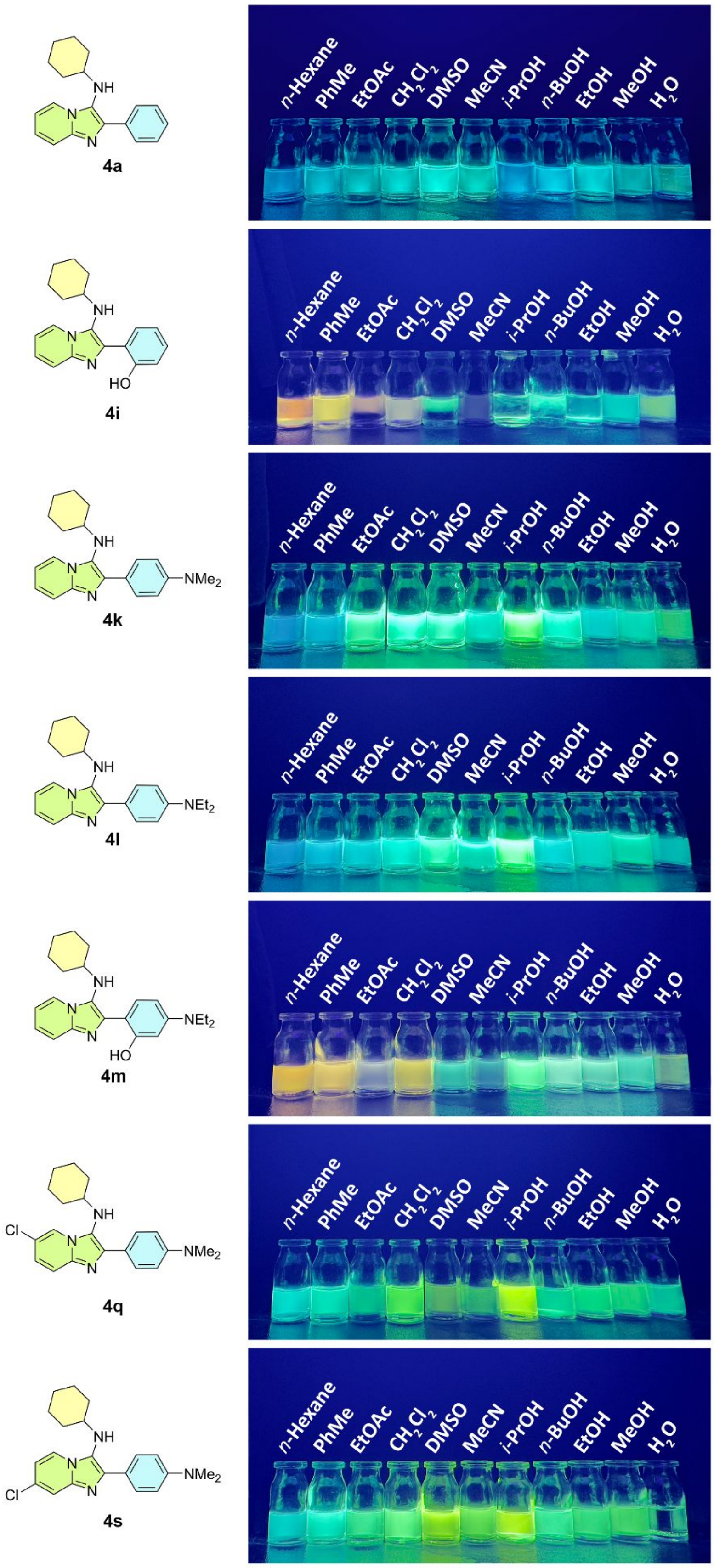

Figure S25. Emission solvatochromism for selected compounds ($5 \cdot 10^{-5}$ M) at room temperature; λ_{exc} = 365 nm, in a dark chamber with an UV lamp.

Table S3. Solvents and their physical and solvatochromic parameters used in this study.[14]

Solvent	Type of Solvent[a]	ε_r[b]	*n*[c]	Δf[d]	π^*[e]	β[f]	α[g]	$E_T(30)$[h]
n-Hexane	NHB	1.880	1.375	-0.001	-0.08	0.00	0.00	31.0
PhMe	Ar-NHB-HBA	2.380	1.497	0.013	0.54	0.11	0.00	33.9
EtOAc	HBA	6.020	1.372	0.200	0.55	0.45	0.00	38.1
CH_2Cl_2	NHB	8.930	1.424	0.217	0.82	0.00	0.30	40.7
DMSO	HBA	46.45	1.479	0.263	1.00	0.76	0.00	45.1
MeCN	HBA-HBD	35.94	1.344	0.305	0.75	0.31	0.19	45.6
i-PrOH	HBA-D	19.92	1.377	0.276	0.48	0.95	0.76	48.4
n-BuOH	HBA-D	17.51	1.399	0.264	0.47	0.88	0.79	49.7
EtOH	HBA-D	24.55	1.361	0.289	0.54	0.77	0.83	51.9
MeOH	HBA-D	32.66	1.328	0.309	0.60	0.62	0.93	55.4
H_2O	HBA-D	78.36	1.333	0.320	1.09	0.18	1.17	63.1

[a] NHB = non-hydrogen-bonding solvent; HBA = hydrogen bond acceptor; HBD = hydrogen bond donor; HBA-D = amphiprotic hydrogen bond acceptor-donor; Ar = aromatic solvents. [b] ε_r = dielectric constant. [c] *n* = solvent refractive index. [d] Lippert–Mataga solvent polarity. [e] π^* = the solvent's dipolarity/polarizability. [f] β = the solvent's hydrogen bond acceptor basicity. [g] α = the solvent's hydrogen bond donor acidity. [h] Empirical Parameters of Solvent Polarity $E_T(30)$ (kcal·mol^{-1}) values, derived from the transition of energy at 25 ºC of the long-wavelength visible absorption of a standard pyridinium *N*-phenolate betaine dye polarity scale.

Table S4. Photophysical properties of all compounds in different solvents.[a]

Comp.	Solvent	λ_{abs}, nm[b]	λ_{em}, nm[c]	log ε (ε, $M^{-1}\cdot cm^{-1}$)[d]	Stokes Shift, cm^{-1}[e]	Φ_f[f]
**4a**	*n*-Hexane	336	455	3.63 (4306)	7784	0.02
	PhMe	340	464	3.79 (6193)	7860	0.33
	EtOAc	341	476	3.77 (5898)	8317	0.26
	CH_2Cl_2	340	477	3.73 (5384)	8447	0.39
	DMSO	344	487	3.73 (5354)	8536	0.48
	MeCN	340	482	3.70 (5066)	8665	0.13
	i-PrOH	337	474	3.63 (4260)	8577	0.22
	n-BuOH	337	469	3.70 (5063)	8352	0.03
	EtOH	336	473	3.76 (5693)	8620	0.15
	MeOH	334	476	3.53 (3410)	8932	0.18
	H_2O	326	488	3.64 (4397)	10183	0.06
**4b**	*n*-Hexane	329	460	3.60 (3947)	8656	0.00
	PhMe	332	470	3.61 (4076)	8844	0.02
	EtOAc	332	471	3.53 (3406)	8889	0.01
	CH_2Cl_2	330	469	3.53 (3356)	8981	0.01
	DMSO	336	484	3.53 (3415)	9101	0.02
	MeCN	331	464	3.50 (3186)	8660	0.02
	i-PrOH	327	458	3.56 (3635)	8747	0.07
	n-BuOH	326	466	3.56 (3622)	9216	0.04
	EtOH	326	469	3.57 (3697)	9353	0.02
	MeOH	325	477	3.55 (3515)	9805	0.02
	H_2O	315	471	3.47 (2922)	10515	0.02
**4c**	*n*-Hexane	339	456	3.81 (6426)	7569	0.04
	PhMe	343	464	3.94 (8798)	7603	0.45
	EtOAc	343	473	3.93 (8468)	8013	0.04
	CH_2Cl_2	341	469	3.97 (9290)	8004	0.51
	DMSO	346	485	3.91 (8152)	8283	0.18
	MeCN	341	478	3.92 (8355)	8405	0.16
	i-PrOH	338	468	3.79 (6215)	8218	0.35
	n-BuOH	338	469	3.96 (9223)	8264	0.03
	EtOH	337	473	3.92 (8369)	8532	0.32

	MeOH	336	472	3.94 (8777)	8575	0.25
	H_2O	345	470	3.91 (8140)	7709	0.07
**4d**	*n*-Hexane	318	-[g]	3.57 (3741)	-	0.00
	PhMe	318	-[g]	3.73 (5411)	-	0.01
	EtOAc	326	-[g]	3.67 (4697)	-	0.00
	CH_2Cl_2	319	-[g]	3.73 (5400)	-	0.00
	DMSO	320	-[g]	3.63 (4231)	-	0.00
	MeCN	326	-[g]	3.68 (4789)	-	0.00
	i-PrOH	317	-[g]	3.65 (4436)	-	0.00
	n-BuOH	319	-[g]	3.61 (4030)	-	0.02
	EtOH	318	-[g]	3.68 (4757)	-	0.00
	MeOH	315	-[g]	3.73 (5359)	-	0.00
	H_2O	-[h]	-	-	-	0.00
**4e**	*n*-Hexane	-[h]	-	-	-	0.01
	PhMe	376	-[g]	4.01 (10292)	-	0.01
	EtOAc	381	-[g]	4.00 (10044)	-	0.00
	CH_2Cl_2	381	-[g]	4.00 (9985)	-	0.00
	DMSO	399	-[g]	3.94 (8650)	-	0.00
	MeCN	384	-[g]	3.99 (9667)	-	0.00
	i-PrOH	376	-[g]	3.97 (9295)	-	0.00
	n-BuOH	375	-[g]	4.06 (11533)	-	0.02
	EtOH	374	-[g]	3.94 (8741)	-	0.00
	MeOH	372	-[g]	4.00 (10057)	-	0.00
	H_2O	-[h]	-	-	-	0.00
**4f**	*n*-Hexane	347	476	4.01 (10180)	7810	0.00
	PhMe	352	482	4.02 (10499)	7662	0.07
	EtOAc	352	493	4.02 (10466)	8125	0.51
	CH_2Cl_2	351	496	4.01 (10313)	8329	0.46
	DMSO	358	521	4.01 (10205)	8739	0.01
	MeCN	351	499	4.02 (10587)	8450	0.02
	i-PrOH	350	496	3.94 (8678)	8410	0.05
	n-BuOH	350	496	4.04 (11075)	8410	0.06
	EtOH	348	498	4.04 (10915)	8655	0.01
	MeOH	347	487	3.96 (9094)	8285	0.00

	H_2O	343	500	3.91 (8126)	9155	0.00
**4g**	*n*-Hexane	337	469	3.81 (6522)	8352	0.04
	PhMe	341	482	3.87 (7337)	8579	0.40
	EtOAc	340	486	3.82 (6642)	8836	0.30
	CH_2Cl_2	340	489	3.79 (6220)	8962	0.43
	DMSO	344	498	3.85 (7105)	8989	0.34
	MeCN	340	492	3.74 (5525)	9087	0.19
	i-PrOH	336	481	3.89 (7739)	8972	0.28
	n-BuOH	336	481	3.88 (7590)	8972	0.25
	EtOH	334	482	3.85 (7106)	9193	0.19
	MeOH	332	484	3.82 (6587)	9459	0.18
	H_2O	324	495	3.83 (6727)	10662	0.07
**4h**	*n*-Hexane	340	457	3.84 (6840)	7530	0.01
	PhMe	343	466	3.95 (8842)	7695	0.41
	EtOAc	343	476	3.93 (8495)	8146	0.33
	CH_2Cl_2	341	478	3.94 (8756)	8405	0.40
	DMSO	346	490	3.87 (7347)	8494	0.52
	MeCN	340	483	3.88 (7591)	8708	0.22
	i-PrOH	340	474	3.93 (8467)	8315	0.46
	n-BuOH	340	475	3.94 (8766)	8359	0.25
	EtOH	338	476	3.90 (7989)	8577	0.26
	MeOH	336	478	3.87 (7404)	8841	0.31
	H_2O	330	491	3.84 (6927)	9936	0.09
**4i**	*n*-Hexane	345	572	4.03 (10752)	11503	0.01
	PhMe	346	565	4.03 (10830)	11203	0.07
	EtOAc	343	473	3.99 (9735)	8013	0.02
	CH_2Cl_2	342	478	3.96 (9076)	8319	0.03
	DMSO	343	495	3.98 (9497)	8952	0.21
	MeCN	340	480	3.94 (8761)	8578	0.01
	i-PrOH	337	486	3.96 (9099)	9097	0.01
	n-BuOH	336	488	3.97 (9400)	9270	0.13
	EtOH	335	485	3.88 (7640)	9232	0.01
	MeOH	332	486	3.94 (8717)	9544	0.05
	H_2O	324	492	3.87 (7437)	10539	0.02

**4j**	*n*-Hexane	340	473	3.73 (5326)	8270	0.00
	PhMe	342	468	3.91 (8151)	7872	0.50
	EtOAc	342	477	3.88 (7565)	8275	0.40
	CH_2Cl_2	340	478	3.85 (7056)	8491	0.04
	DMSO	346	490	3.98 (9550)	8494	0.46
	MeCN	340	483	3.91 (8046)	8708	0.15
	i-PrOH	340	475	3.76 (5720)	8359	0.39
	n-BuOH	339	476	3.63 (4246)	8490	0.27
	EtOH	338	478	3.87 (7353)	8665	0.27
	MeOH	336	480	3.84 (6981)	8929	0.23
	H_2O	329	490	3.81 (6484)	9987	0.11
**4k**	*n*-Hexane	348	461	4.18 (15115)	7044	0.05
	PhMe	352	468	4.18 (14993)	7042	0.58
	EtOAc	350	476	4.19 (15464)	7563	0.55
	CH_2Cl_2	351	483	4.17 (14818)	7786	0.84
	DMSO	356	491	4.17 (14810)	7723	0.38
	MeCN	350	485	4.19 (15660)	7953	0.54
	i-PrOH	350	478	4.19 (15472)	7651	0.62
	n-BuOH	350	475	4.14 (13683)	7519	0.73
	EtOH	350	480	4.16 (14572)	7738	0.48
	MeOH	348	490	4.19 (15485)	8327	0.53
	H_2O	340	503	3.98 (9626)	9531	0.15
**4l**	*n*-Hexane	351	457	4.17 (14895)	6608	0.13
	PhMe	355	467	4.19 (15596)	6756	0.74
	EtOAc	353	476	4.19 (15337)	7320	0.65
	CH_2Cl_2	356	485	4.21 (16197)	7471	0.68
	DMSO	361	493	4.19 (15515)	7417	0.45
	MeCN	353	486	4.20 (15860)	7752	0.70
	i-PrOH	353	480	4.20 (16031)	7495	0.81
	n-BuOH	354	478	4.18 (15154)	7328	0.83
	EtOH	354	486	4.19 (15548)	7672	0.78
	MeOH	352	484	4.19 (15640)	7748	0.71
	H_2O	359	487	3.99 (9837)	7321	0.17
**4m**	*n*-Hexane	364	551	4.32 (20946)	9324	0.04

	PhMe	368	560	4.29 (19510)	9317	0.31
	EtOAc	365	469	4.29 (19279)	6075	0.13
	CH_2Cl_2	366	551	4.34 (21655)	9174	0.02
	DMSO	367	491	4.27 (18720)	6881	0.23
	MeCN	361	487	4.32 (20707)	7167	0.00
	i-PrOH	359	509	4.20 (15975)	8209	0.72
	n-BuOH	359	517	4.29 (19314)	8513	0.60
	EtOH	358	499	4.25 (17816)	7893	0.34
	MeOH	356	507	4.27 (18690)	8366	0.30
	H_2O	359	529	3.77 (5832)	8952	0.05
**4n**	*n*-Hexane	344	462	3.81 (6478)	7425	0.00
	PhMe	349	473	3.87 (7399)	7512	0.05
	EtOAc	350	479	3.90 (8028)	7695	0.48
	CH_2Cl_2	345	482	3.80 (6247)	8239	0.31
	DMSO	356	495	3.85 (7132)	7888	0.52
	MeCN	350	486	3.85 (7003)	7995	0.25
	i-PrOH	350	482	3.82 (6557)	7825	0.59
	n-BuOH	350	481	3.87 (7423)	7781	0.34
	EtOH	350	484	3.83 (6719)	7910	0.30
	MeOH	343	484	3.84 (6878)	8493	0.27
	H_2O	353	489	3.65 (4436)	7879	0.12
**4o**	*n*-Hexane	325	457	3.45 (2824)	8887	0.01
	PhMe	328	468	3.44 (2756)	9120	0.07
	EtOAc	330	476	3.41 (2563)	9295	0.04
	CH_2Cl_2	327	475	3.49 (3079)	9528	0.01
	DMSO	335	494	3.43 (2673)	9608	0.04
	MeCN	329	487	3.48 (3041)	9861	0.02
	i-PrOH	324	478	3.53 (3425)	9944	0.03
	n-BuOH	325	478	3.49 (3118)	9849	0.05
	EtOH	325	479	3.45 (2849)	9892	0.00
	MeOH	323	482	3.40 (2487)	10213	0.01
	H_2O	290	481	3.43 (2693)	13693	0.00
**4p**	*n*-Hexane	-[h]	-	-	-	0.05
	PhMe	358	499	3.99 (9749)	7893	0.05

	EtOAc	357	497	4.00 (10050)	7890	0.03
	CH_2Cl_2	355	498	4.04 (10915)	8089	0.65
	DMSO	363	522	3.96 (9189)	8391	0.01
	MeCN	356	510	4.00 (10015)	8482	0.29
	i-PrOH	354	498	4.01 (10173)	8168	0.20
	n-BuOH	354	499	4.02 (10586)	8209	0.25
	EtOH	352	502	3.99 (9765)	8489	0.10
	MeOH	350	505	3.99 (9750)	8769	0.02
	H_2O	-[h]	-	-	-	0.04
**4q**	*n*-Hexane	359	476	3.99 (9807)	6847	0.05
	PhMe	367	487	4.00 (10095)	6714	0.17
	EtOAc	366	496	3.97 (9414)	7161	0.09
	CH_2Cl_2	366	511	3.99 (9849)	7753	0.01
	DMSO	376	509	3.98 (9528)	6949	0.00
	MeCN	364	506	4.01 (10309)	7710	0.04
	i-PrOH	363	494	4.01 (10312)	7305	0.24
	n-BuOH	365	495	3.99 (9691)	7195	0.15
	EtOH	365	498	3.96 (9122)	7317	0.11
	MeOH	364	498	3.95 (8851)	7392	0.09
	H_2O	367	494	3.56 (3663)	7005	0.01
**4r**	*n*-Hexane	-[h]	-	-	-	0.04
	PhMe	360	501	4.02 (10551)	7818	0.70
	EtOAc	359	499	4.04 (10968)	7815	0.65
	CH_2Cl_2	358	500	4.01 (10318)	7933	0.56
	DMSO	364	522	4.07 (11623)	8315	0.10
	MeCN	359	511	4.04 (11002)	8286	0.11
	i-PrOH	356	497	4.06 (11491)	7969	0.17
	n-BuOH	356	500	4.05 (11167)	8090	0.23
	EtOH	354	503	4.05 (11106)	8368	0.09
	MeOH	354	503	4.04 (10895)	8368	0.02
	H_2O	-[h]	-	-	-	0.00
**4s**	*n*-Hexane	360	480	4.18 (15232)	6944	0.12
	PhMe	366	491	4.21 (16038)	6956	0.81
	EtOAc	364	500	4.18 (15090)	7473	0.53

	CH_2Cl_2	364	502	4.21 (16110)	7552	0.55
	DMSO	371	517	4.20 (15848)	7612	0.43
	MeCN	363	509	4.21 (16167)	7902	0.28
	i-PrOH	362	498	4.13 (13464)	7544	0.65
	n-BuOH	363	498	4.13 (13380)	7468	0.60
	EtOH	363	499	4.18 (15138)	7508	0.52
	MeOH	362	508	4.21 (16164)	7939	0.33
	H_2O	364	496	3.52 (3288)	7311	0.05
**4t**	*n*-Hexane	350	435	4.19 (15375)	5583	0.01
	PhMe	353	435	4.22 (16775)	5340	0.07
	EtOAc	350	447	4.21 (16132)	6200	0.42
	CH_2Cl_2	351	456	4.24 (17317)	6560	0.20
	DMSO	354	460	4.20 (15850)	6509	0.32
	MeCN	347	457	4.23 (16947)	6937	0.24
	i-PrOH	352	453	4.21 (16123)	6334	0.21
	n-BuOH	351	453	4.18 (15118)	6415	0.23
	EtOH	350	455	4.20 (15701)	6593	0.22
	MeOH	350	457	4.21 (16148)	6690	0.20
	H_2O	359	447	4.16 (14358)	5484	0.06
**4u**	*n*-Hexane	350	444	4.11 (12819)	6049	0.02
	PhMe	354	449	4.14 (13653)	5977	0.44
	EtOAc	350	457	4.11 (12966)	6690	0.39
	CH_2Cl_2	352	459	4.04 (10975)	6623	0.28
	DMSO	354	477	4.09 (12235)	7284	0.40
	MeCN	350	466	4.10 (12495)	7112	0.35
	i-PrOH	353	461	4.09 (12188)	6637	0.28
	n-BuOH	352	458	4.10 (12660)	6575	0.42
	EtOH	351	460	4.11 (12818)	6751	0.37
	MeOH	350	468	4.10 (12618)	7204	0.29
	H_2O	365	455	4.33 (21237)	5419	0.11

[a] Carried out at room temperature ($5{\cdot}10^{-5}$ M). [b] λ_{abs} = absorption maxima (nm). [c] λ_{em} = emission maxima (nm). [d] ε = molar absorptivity ($M^{-1}{\cdot}cm^{-1}$). [e] Stokes shifts difference between λ_{em} and λ_{abs}. [f] Φ_f = fluorescence quantum yields (10^{-5} M) were measured at room temperature (366 nm) with reference to quinine sulfate in 0.5 M H_2SO_4 (Φ_{st} = 0.546). [g] Fluorescence was not observed in this solvent. [h] The compound was not soluble in this solvent.

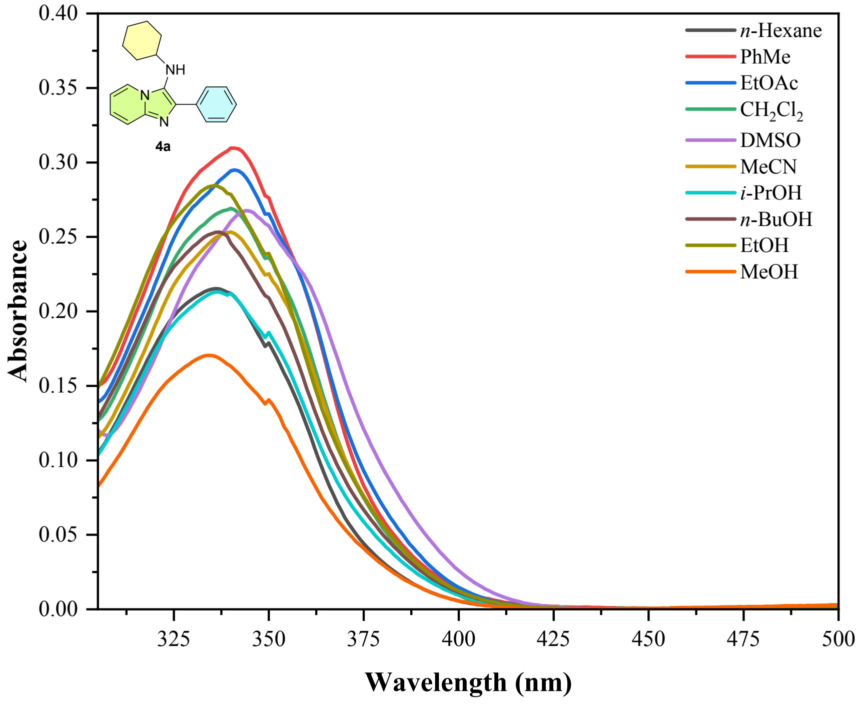

Figure S26. UV-Vis absorption spectra of **4a** in different solvents ($5{\cdot}10^{-5}$ M) at room temperature.

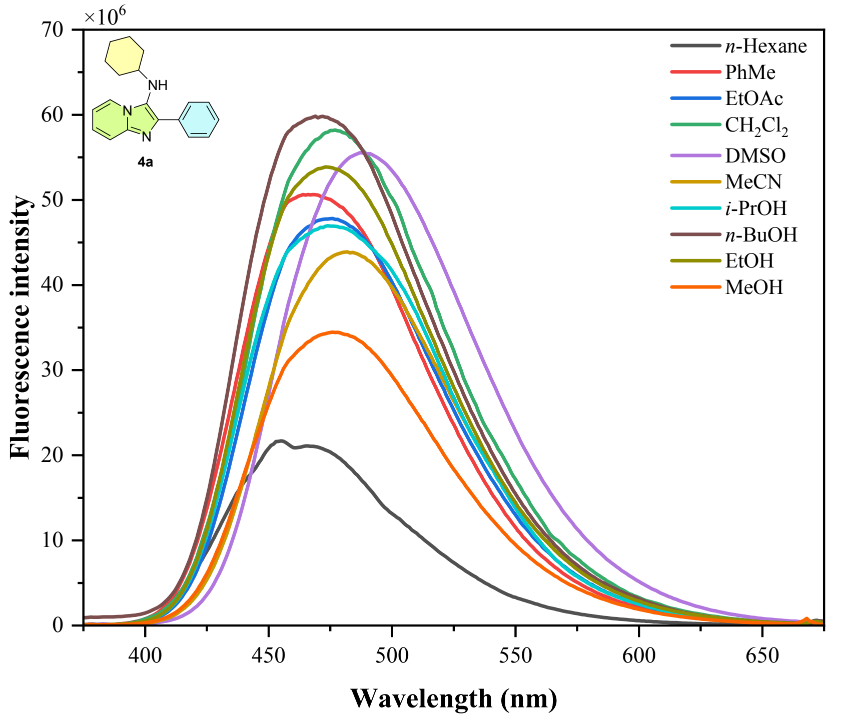

Figure S27. Normalized emission spectra of **4a** in different solvents ($5{\cdot}10^{-5}$ M) at room temperature.

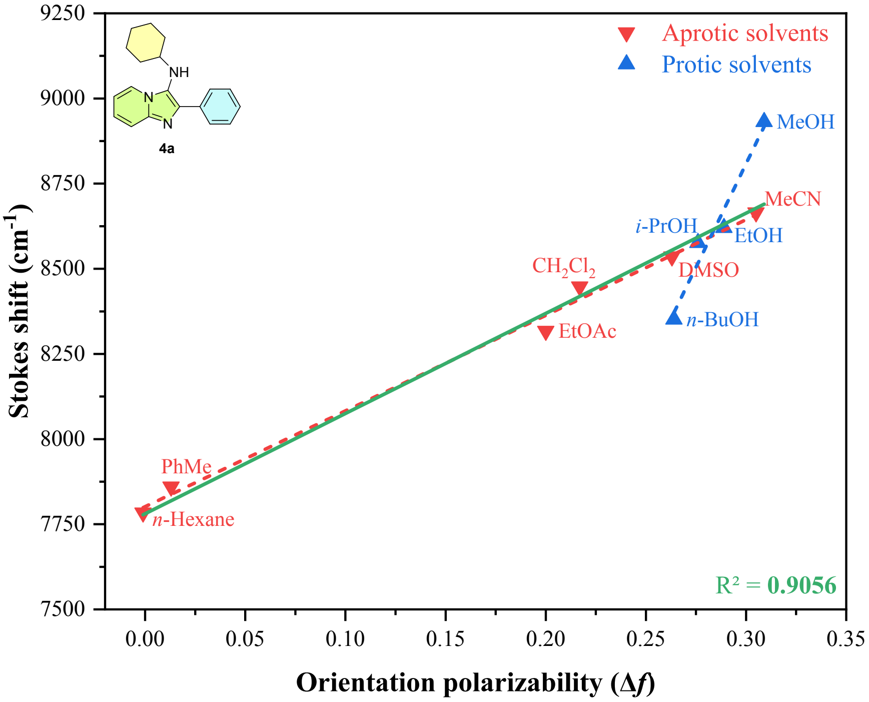

Figure S28. Lippert–Mataga plot showing Stokes shift as a function of solvent orientation polarizability (Δ*f*) for compound **4a**.

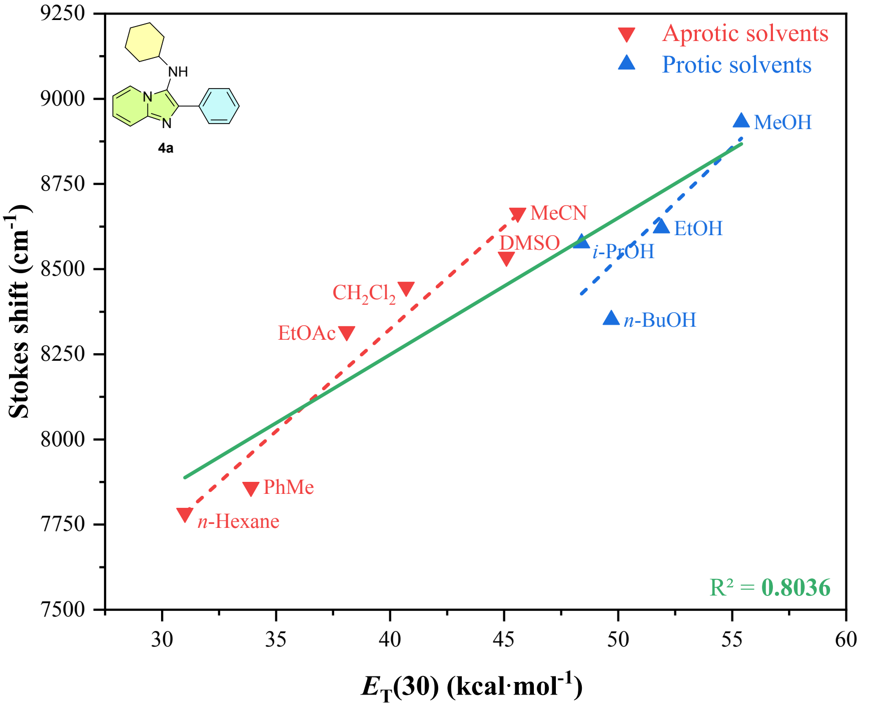

Figure S29. Dimroth-Reichardt plot showing Stokes shift against $E_T(30)$ parameter for compound **4a**.

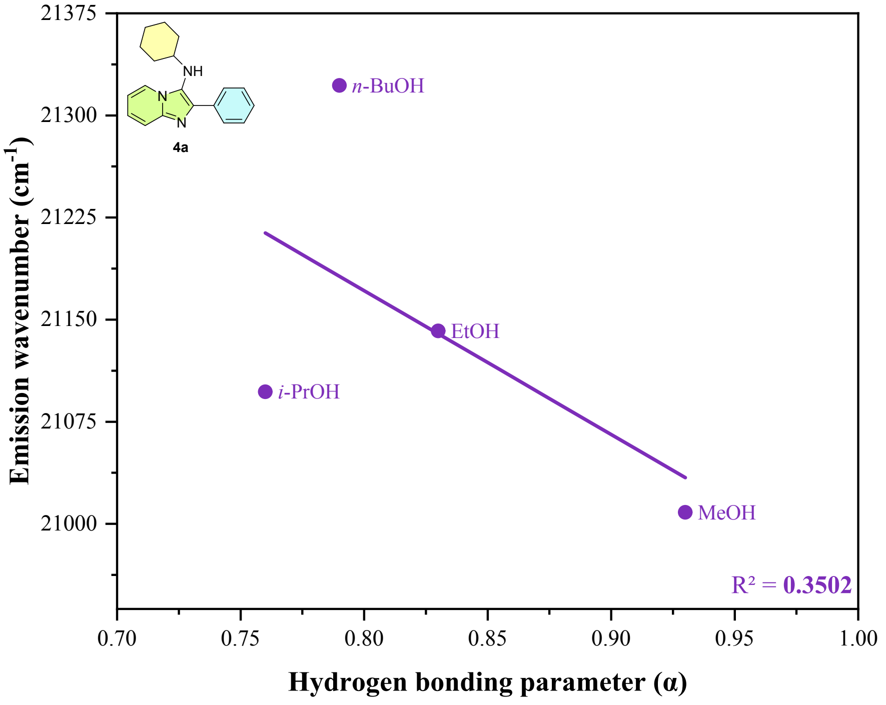

Figure S30. Plot showing hydrogen bonding parameter (α) as a function of emission wavenumber (cm^{-1}) for compound **4a**.

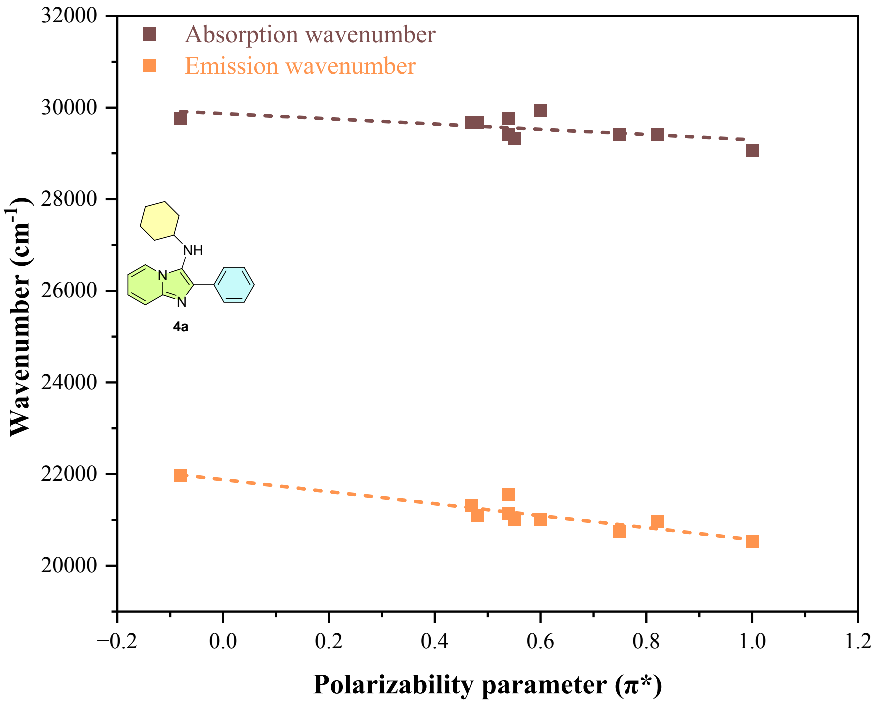

Figure S31. Plot showing polarizability parameter (π*) as a function of absorption and emission wavenumber (cm^{-1}) for compound **4a**.

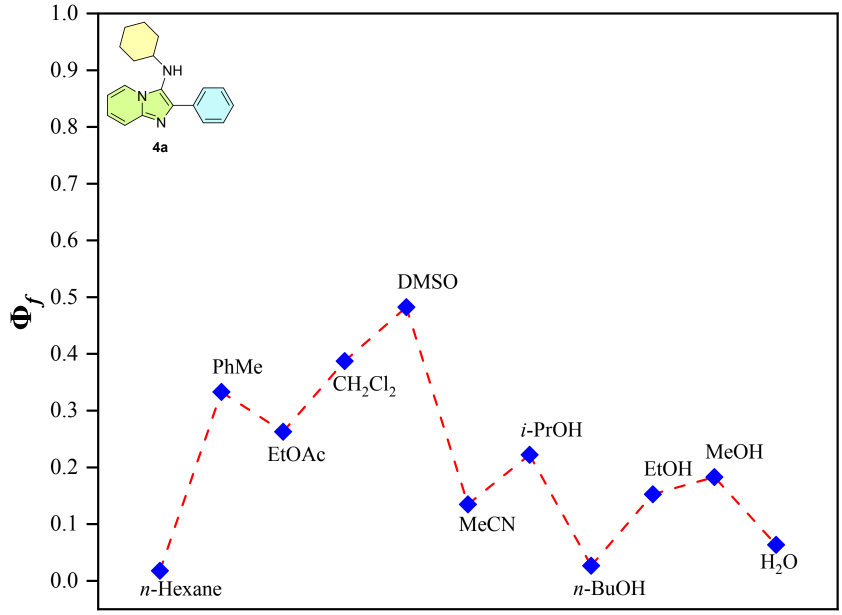

Figure S32. Variation of quantum yields of fluorescence (Φ_f) in different solvents for compound **4a**.

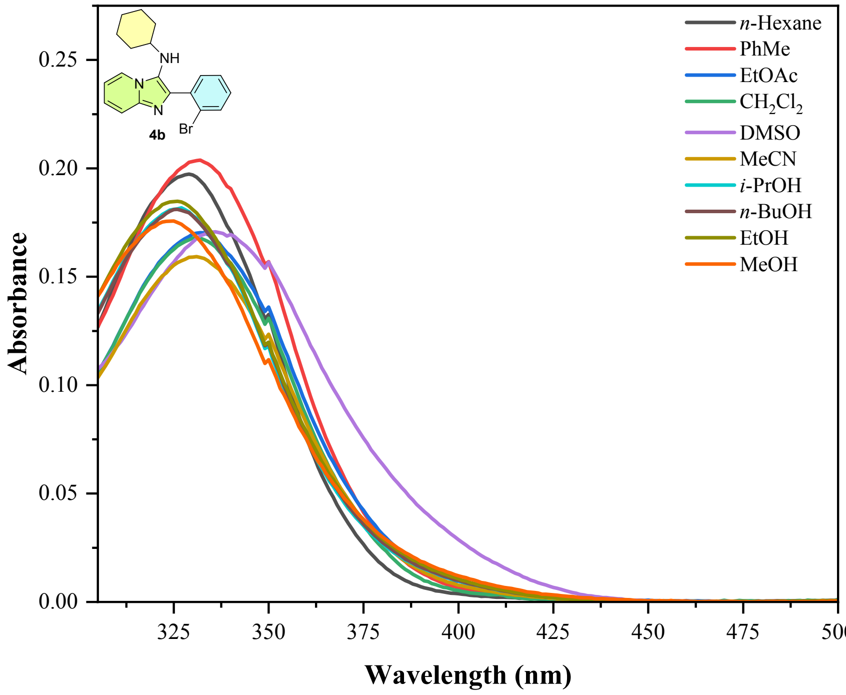

Figure S33. UV-Vis absorption spectra of **4b** in different solvents ($5{\cdot}10^{-5}$ M) at room temperature.

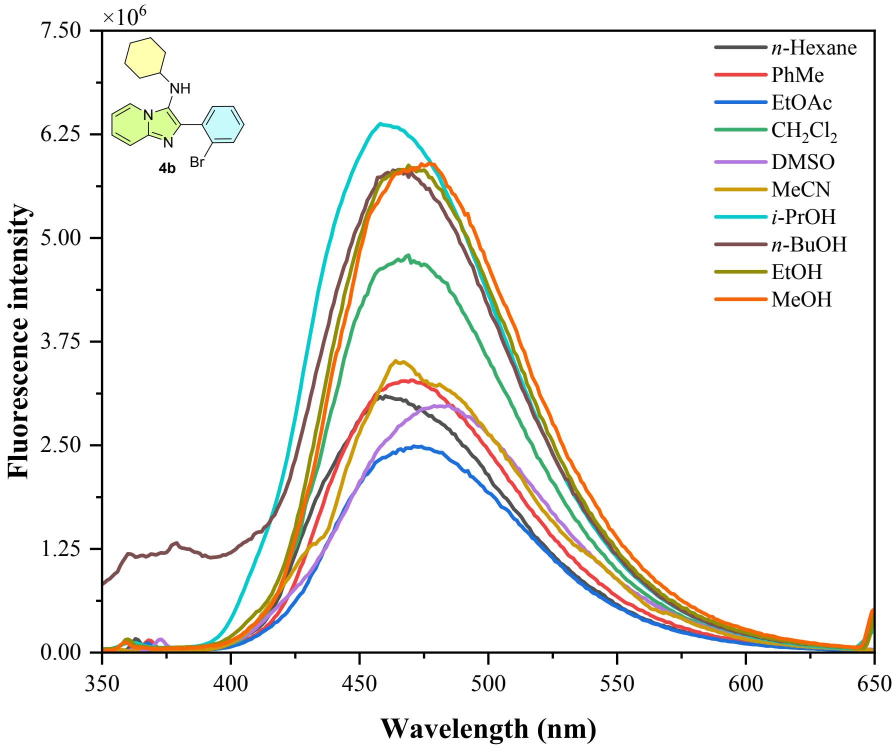

Figure S34. Normalized emission spectra of **4b** in different solvents ($5{\cdot}10^{-5}$ M) at room temperature.

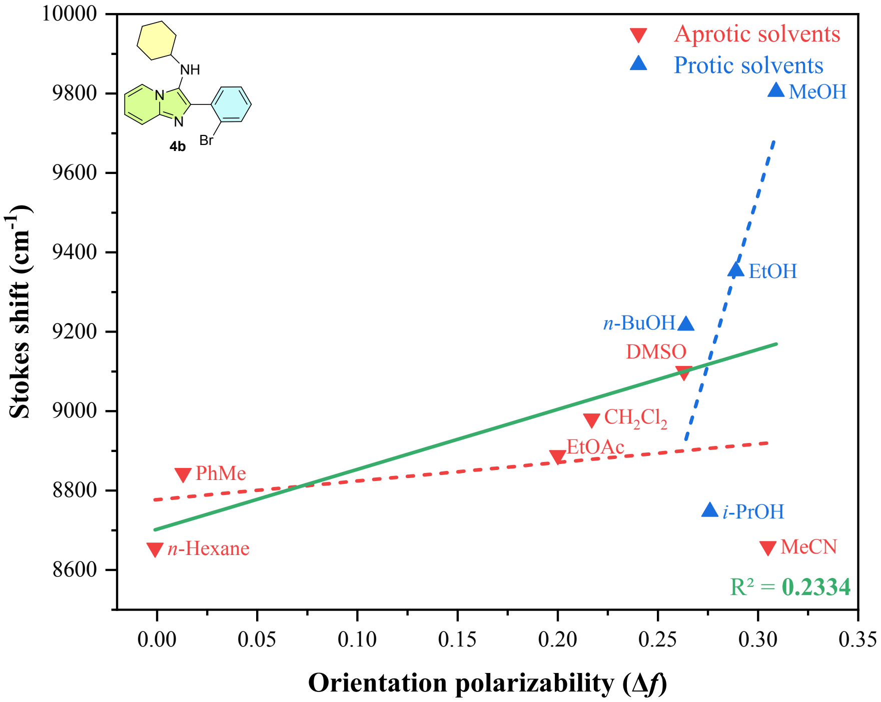

Figure S35. Lippert–Mataga plot showing Stokes shift as a function of solvent orientation polarizability (Δf) for compound **4b**.

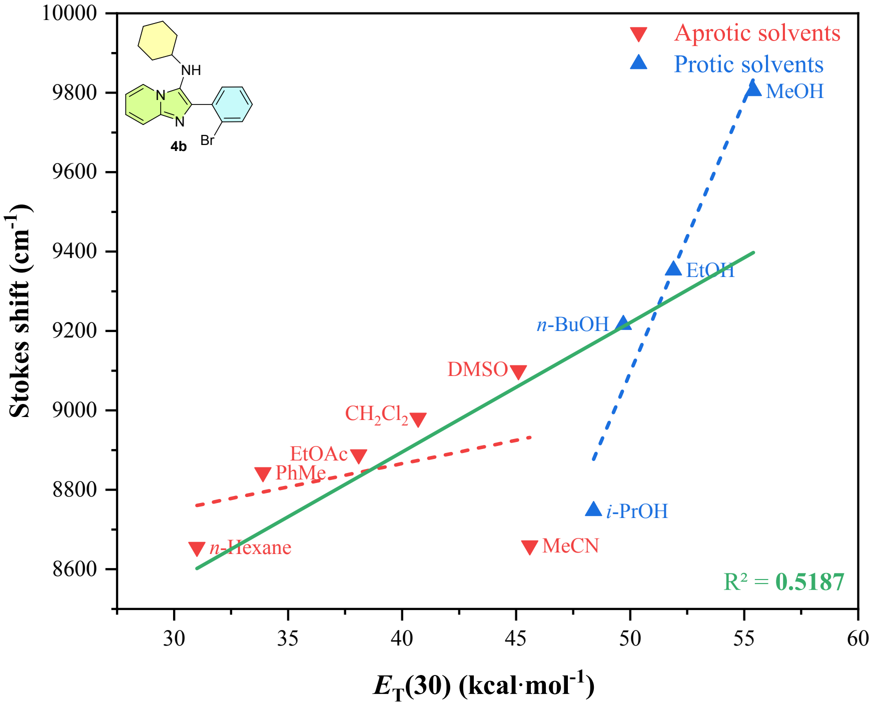

Figure S36. Dimroth-Reichardt plot showing Stokes shift against $E_T(30)$ parameter for compound **4b**.

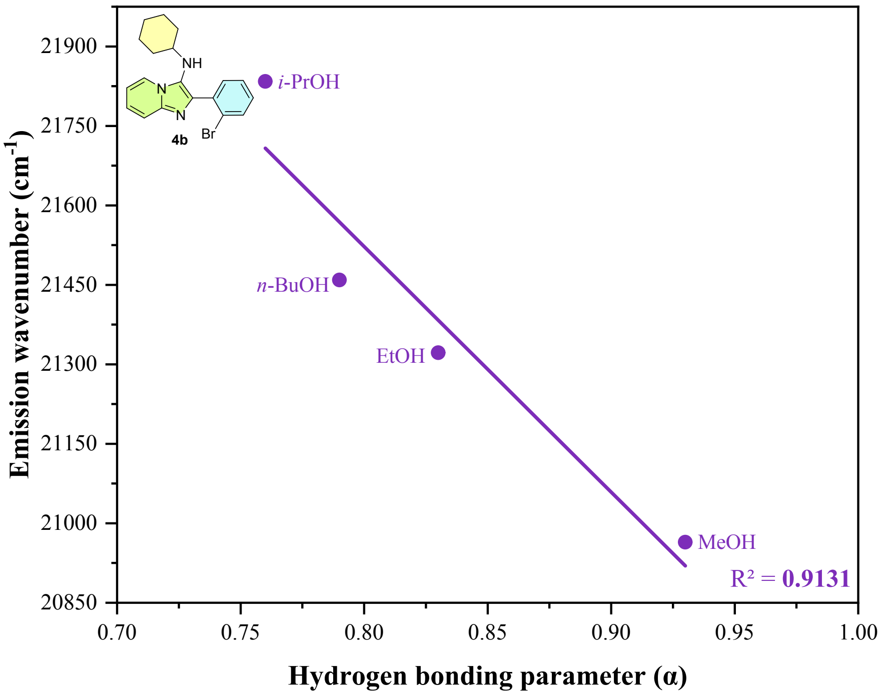

Figure S37. Plot showing hydrogen bonding parameter (α) as a function of emission wavenumber (cm^{-1}) for compound **4b**.

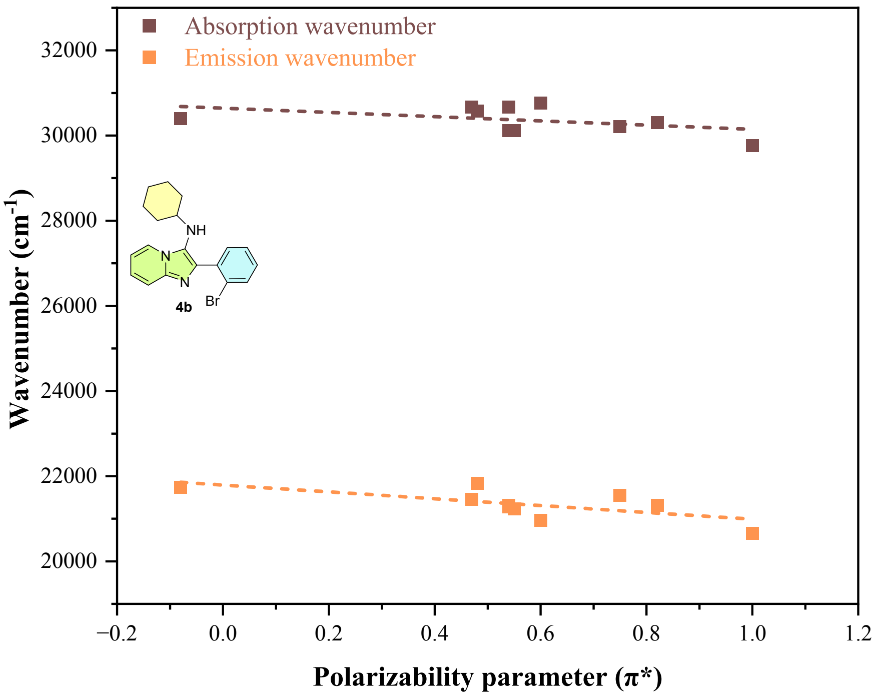

Figure S38. Plot showing polarizability parameter (π*) as a function of absorption and emission wavenumber (cm^{-1}) for compound **4b**.

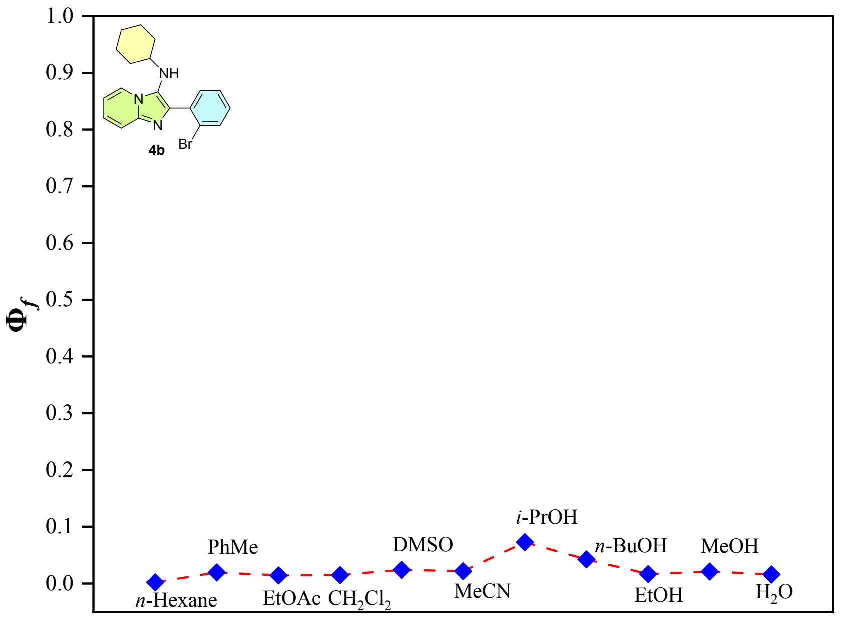

Figure S39. Variation of quantum yields of fluorescence (Φ_f) in different solvents for compound **4b**.

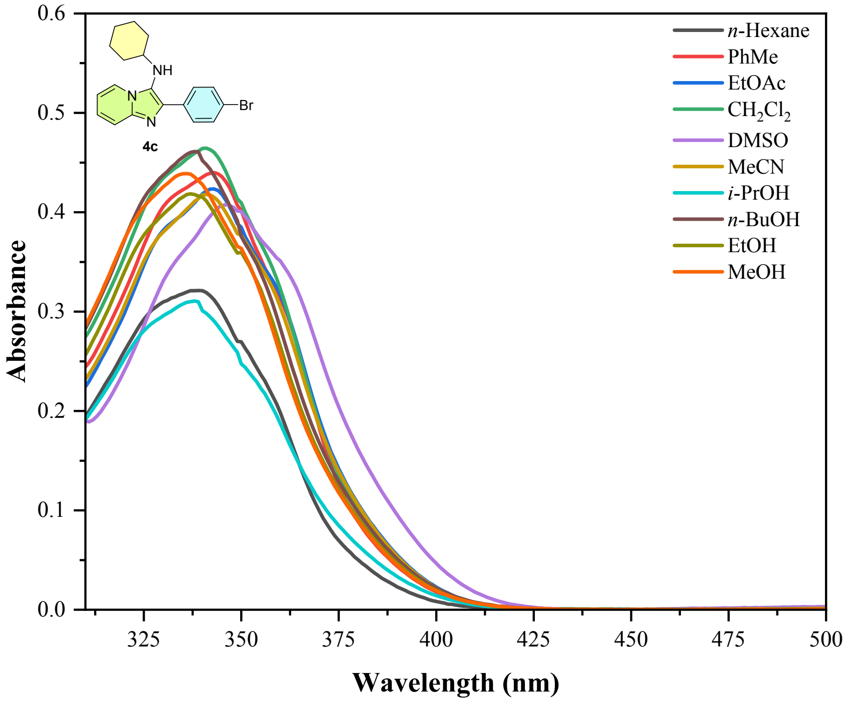

Figure S40. UV-Vis absorption spectra of **4c** in different solvents ($5{\cdot}10^{-5}$ M) at room temperature.

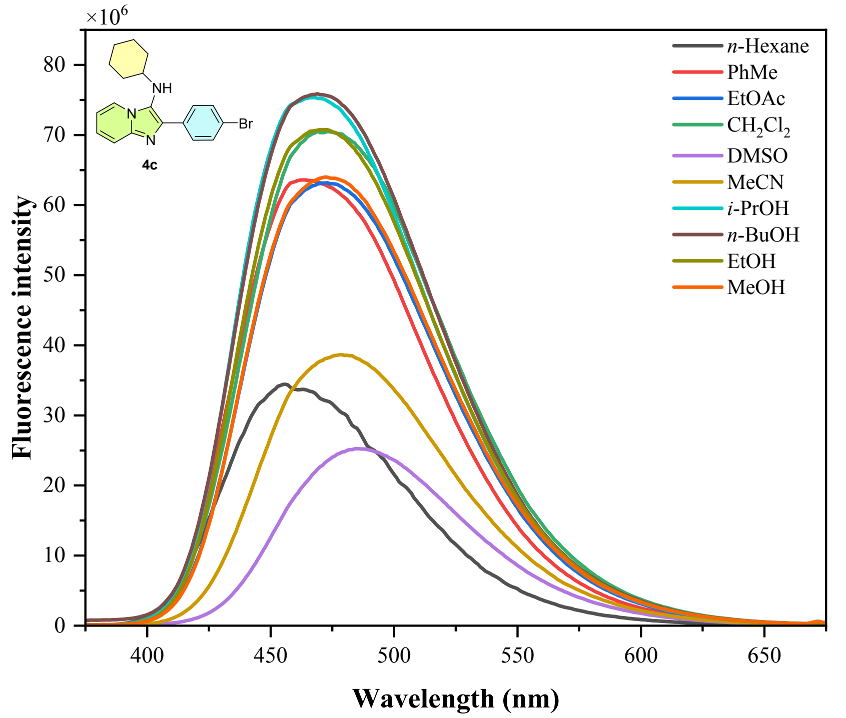

Figure S41. Normalized emission spectra of **4c** in different solvents ($5{\cdot}10^{-5}$ M) at room temperature.

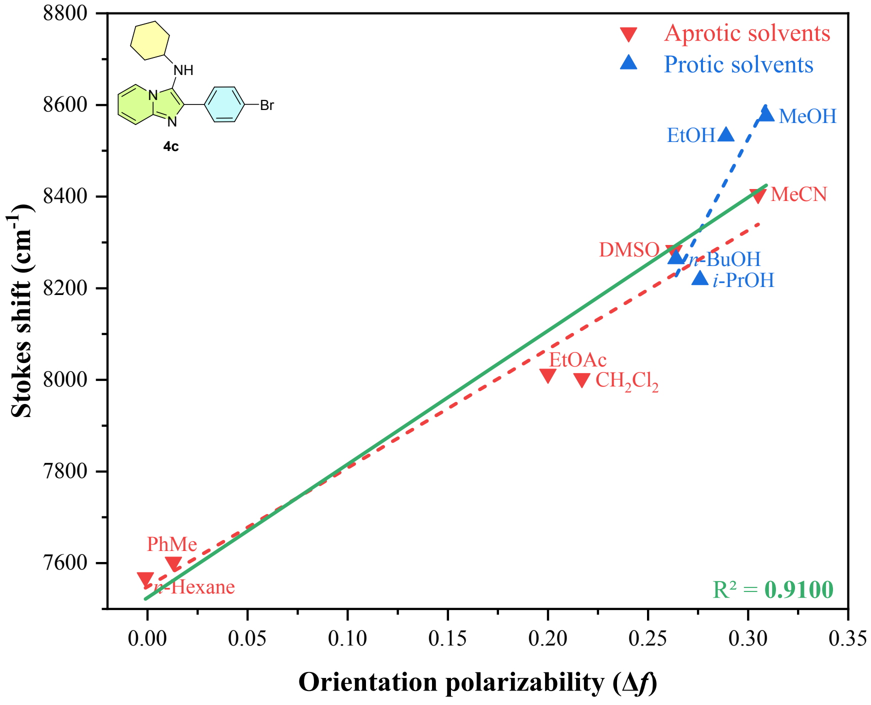

Figure S42. Lippert–Mataga plot showing Stokes shift as a function of solvent orientation polarizability (Δ*f*) for compound **4c**.

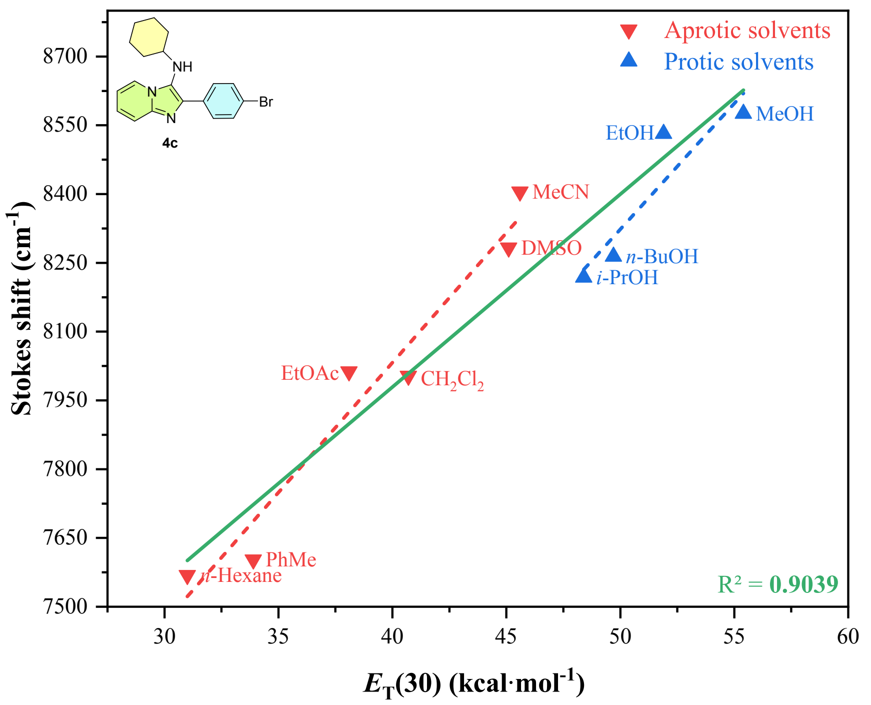

Figure S43. Dimroth-Reichardt plot showing Stokes shift against $E_T(30)$ parameter for compound **4c**.

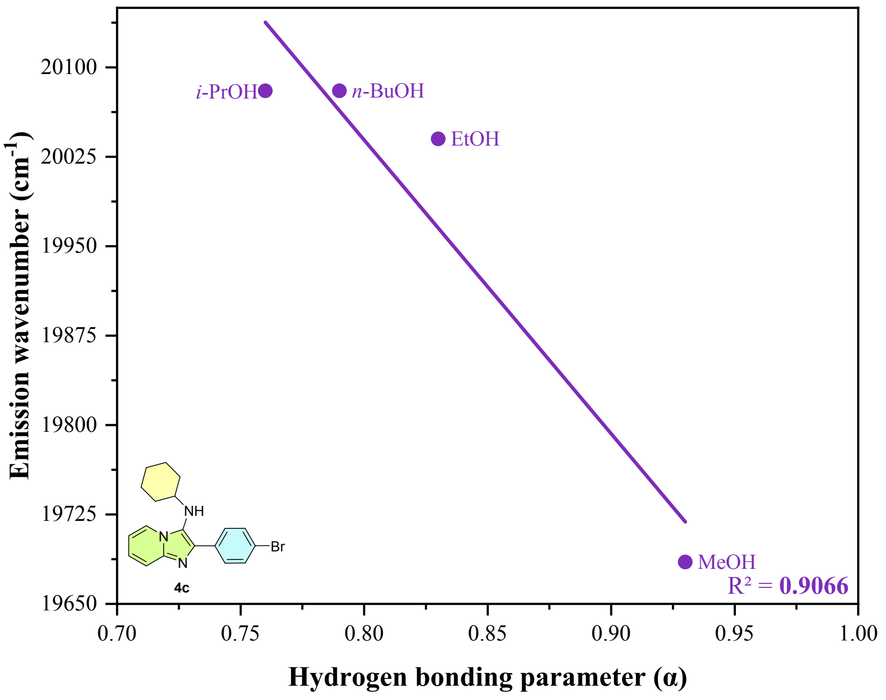

Figure S44. Plot showing hydrogen bonding parameter (α) as a function of emission wavenumber (cm^{-1}) for compound **4c**.

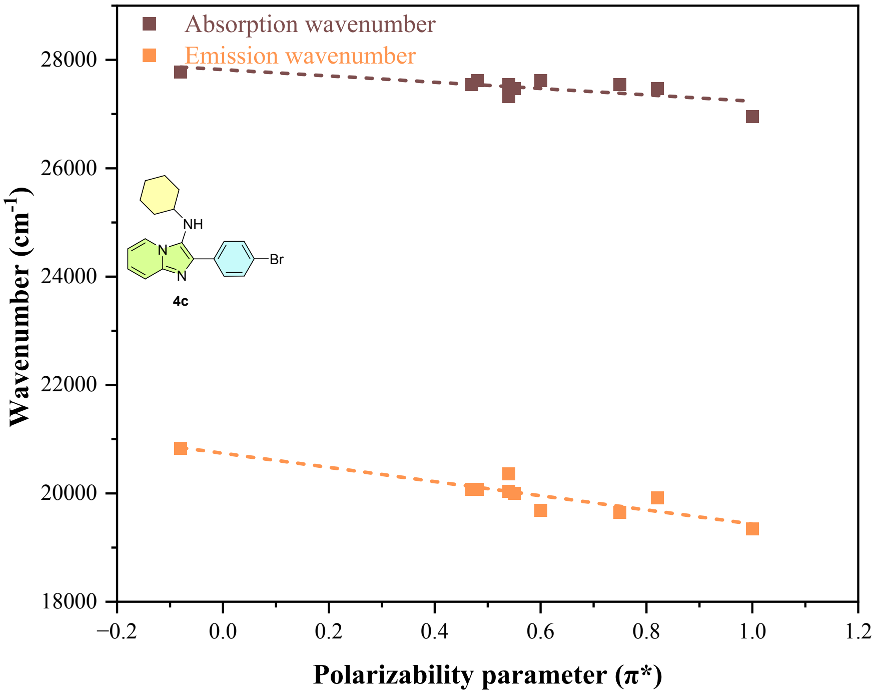

Figure S45. Plot showing polarizability parameter (π*) as a function of absorption and emission wavenumber (cm^{-1}) for compound **4c**.

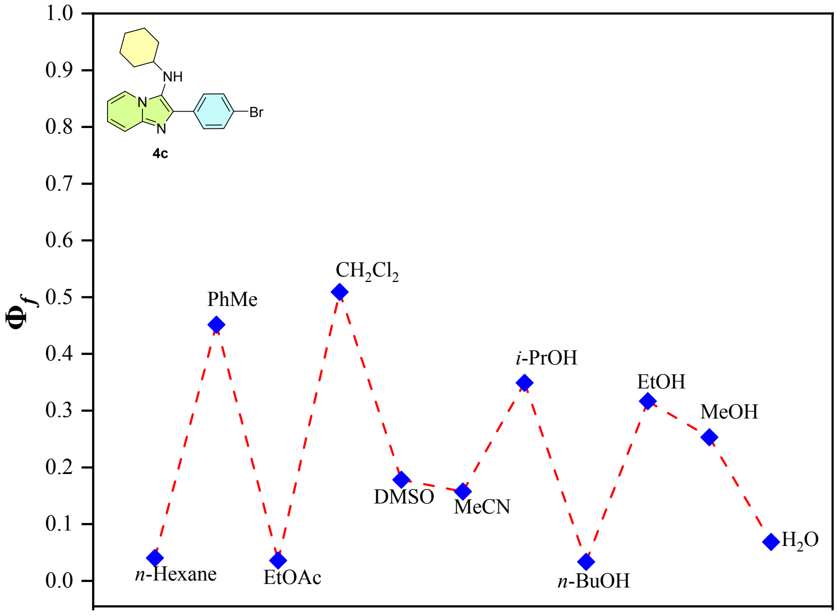

Figure S46. Variation of quantum yields of fluorescence (Φ_f) in different solvents for compound **4c**.

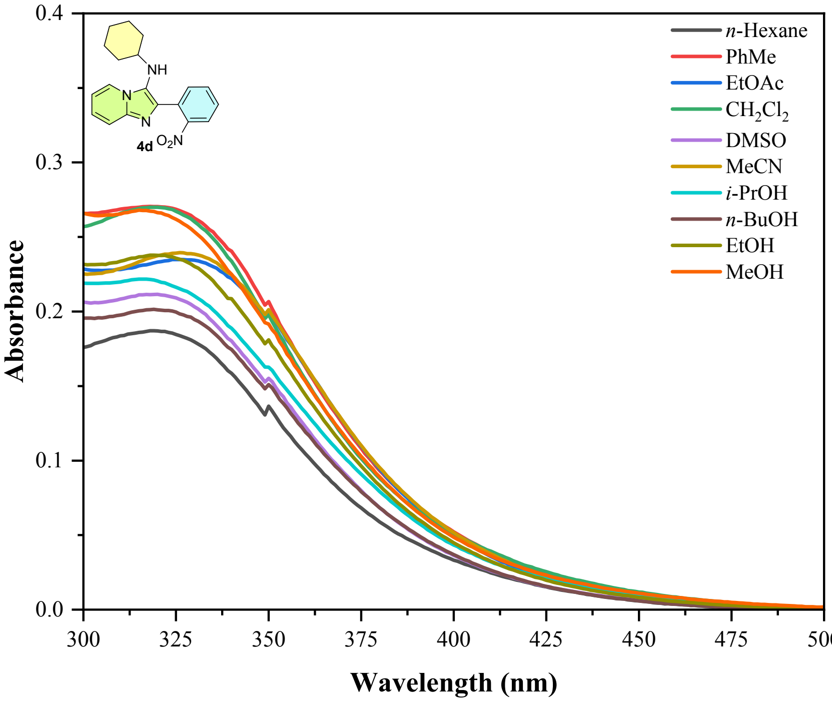

Figure S47. UV-Vis absorption spectra of **4d** in different solvents ($5 \cdot 10^{-5}$ M) at room temperature.

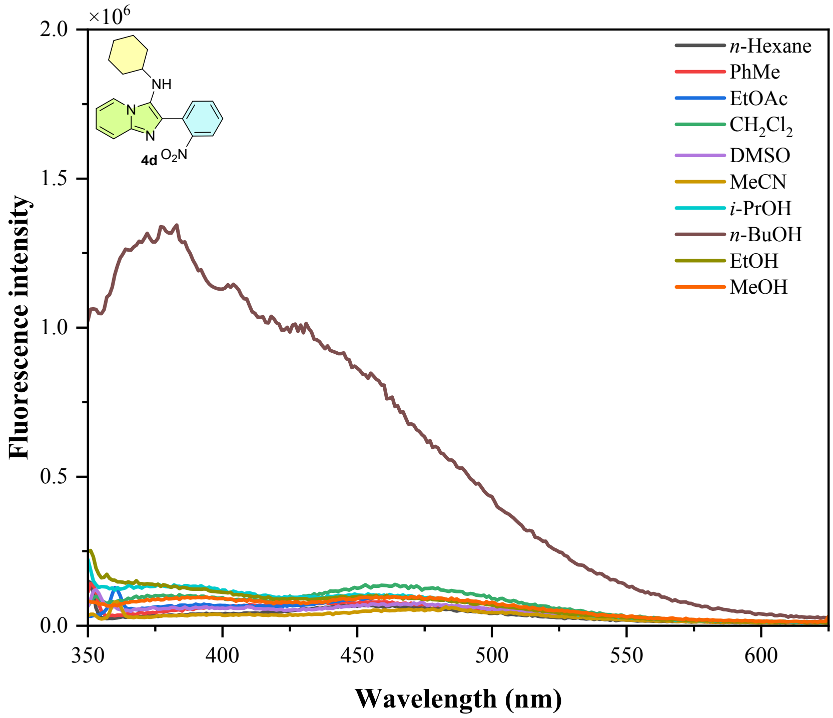

Figure S48. Normalized emission spectra of **4d** in different solvents ($5{\cdot}10^{-5}$ M) at room temperature.

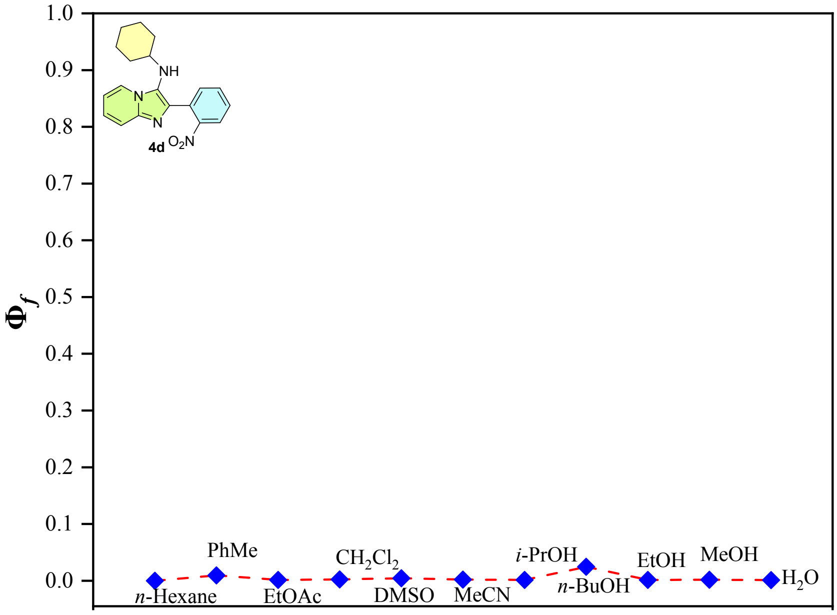

Figure S49. Variation of quantum yields of fluorescence (Φ_f) in different solvents for compound **4d**.

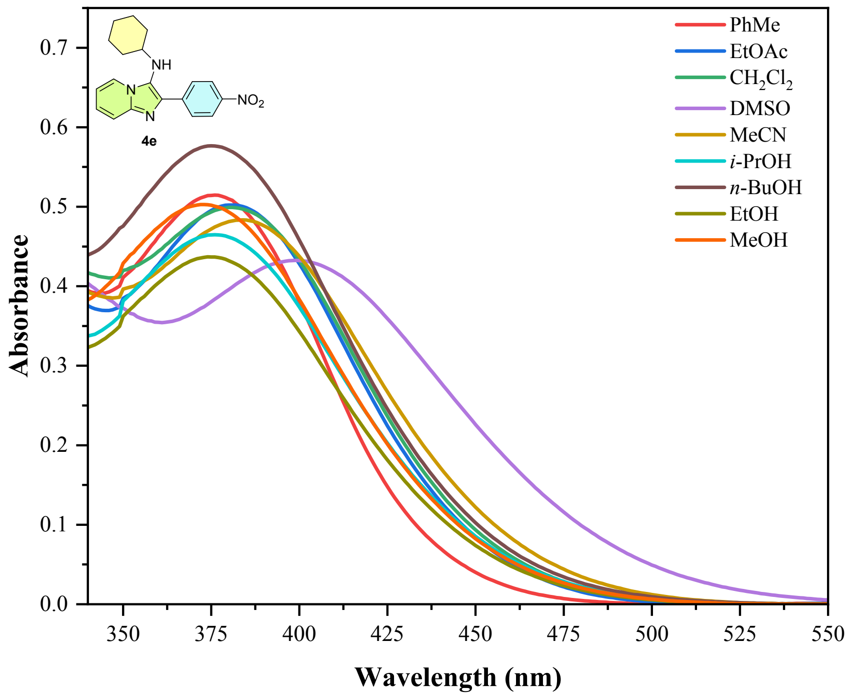

Figure S50. UV-Vis absorption spectra of **4e** in different solvents ($5 \cdot 10^{-5}$ M) at room temperature.

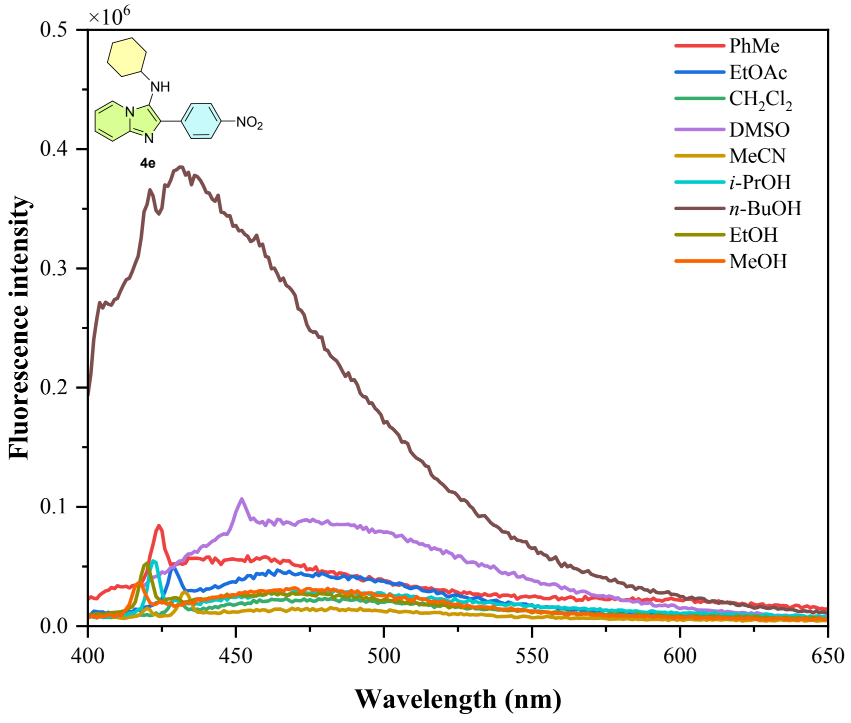

Figure S51. Normalized emission spectra of **4e** in different solvents ($5 \cdot 10^{-5}$ M) at room temperature.

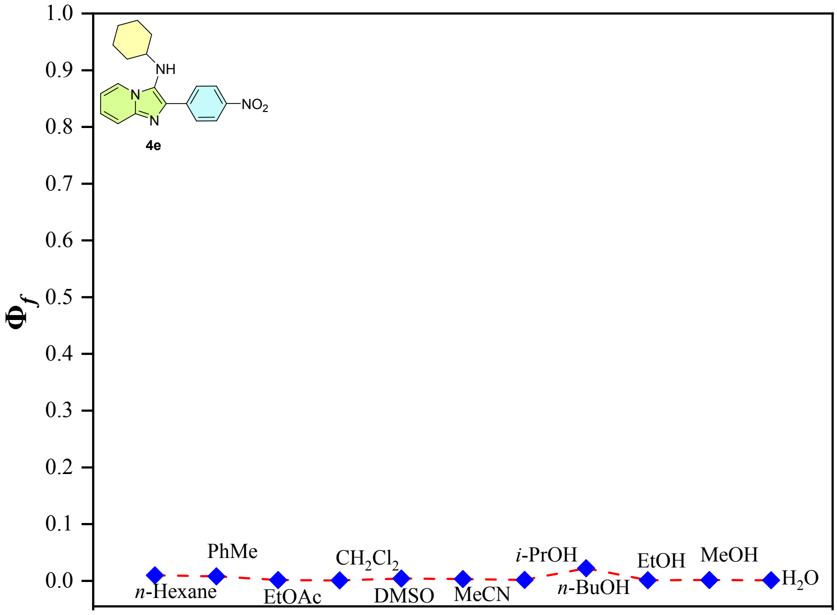

Figure S52. Variation of quantum yields of fluorescence (Φ_f) in different solvents for compound **4e**.

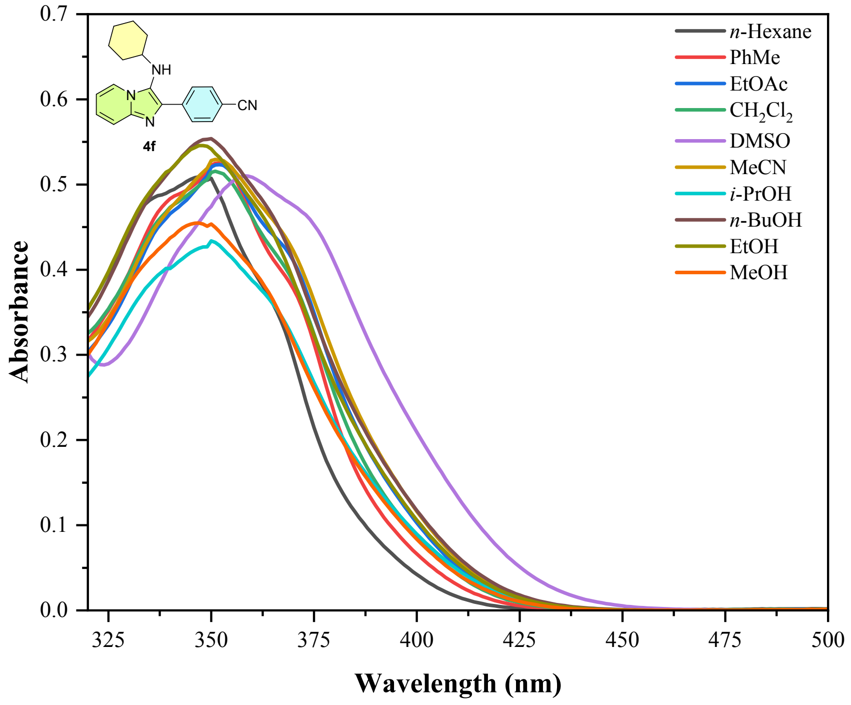

Figure S53. UV-Vis absorption spectra of **4f** in different solvents ($5{\cdot}10^{-5}$ M) at room temperature.

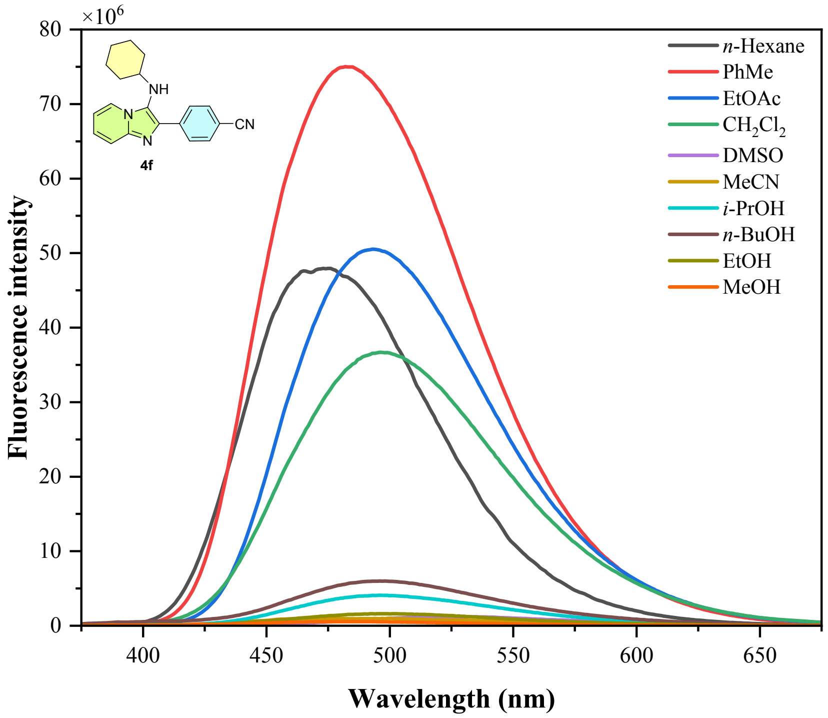

Figure S54. Normalized emission spectra of **4f** in different solvents ($5 \cdot 10^{-5}$ M) at room temperature.

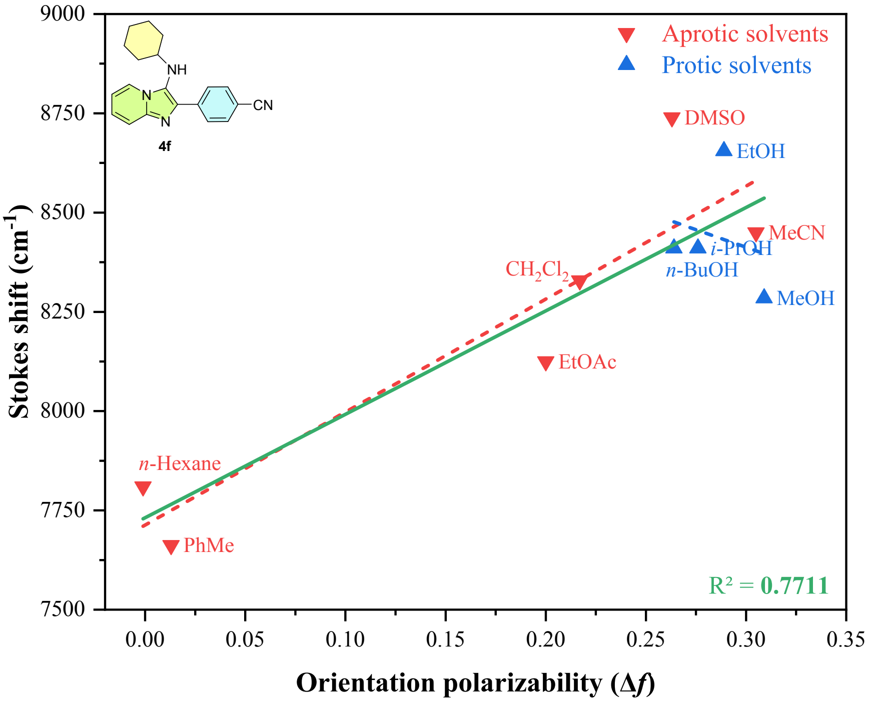

Figure S55. Lippert–Mataga plot showing Stokes shift as a function of solvent orientation polarizability (Δf) for compound **4f**.

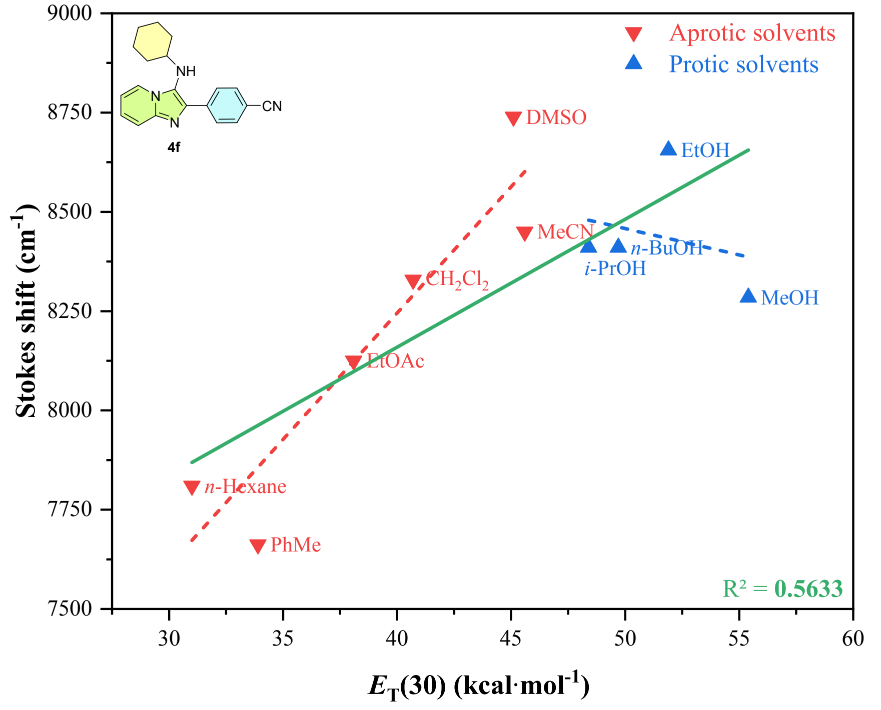

Figure S56. Dimroth-Reichardt plot showing Stokes shift against $E_T(30)$ parameter for compound **4f**.

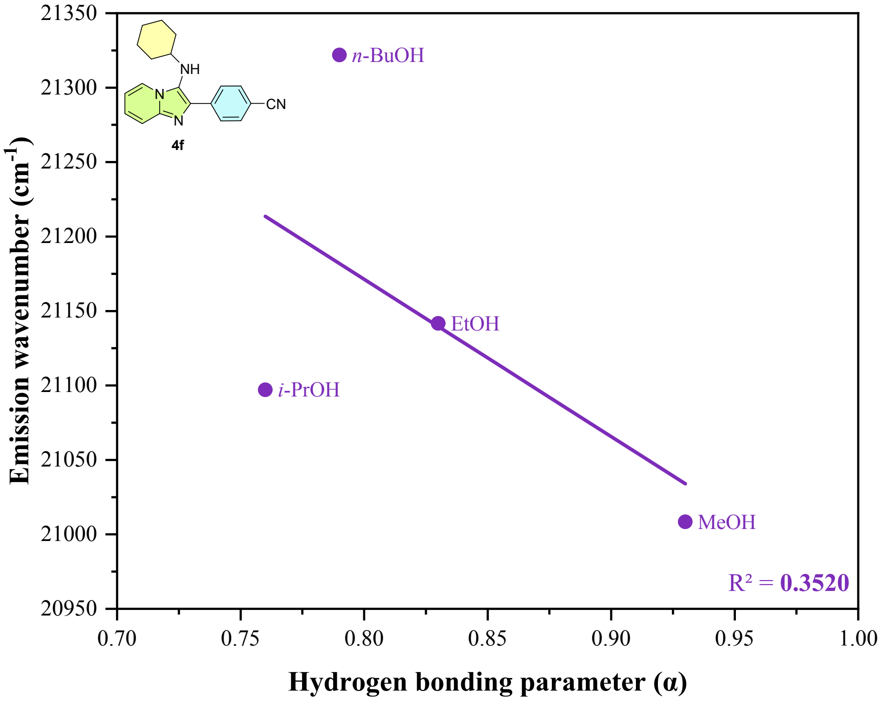

Figure S57. Plot showing hydrogen bonding parameter (α) as a function of emission wavenumber (cm^{-1}) for compound **4f**.

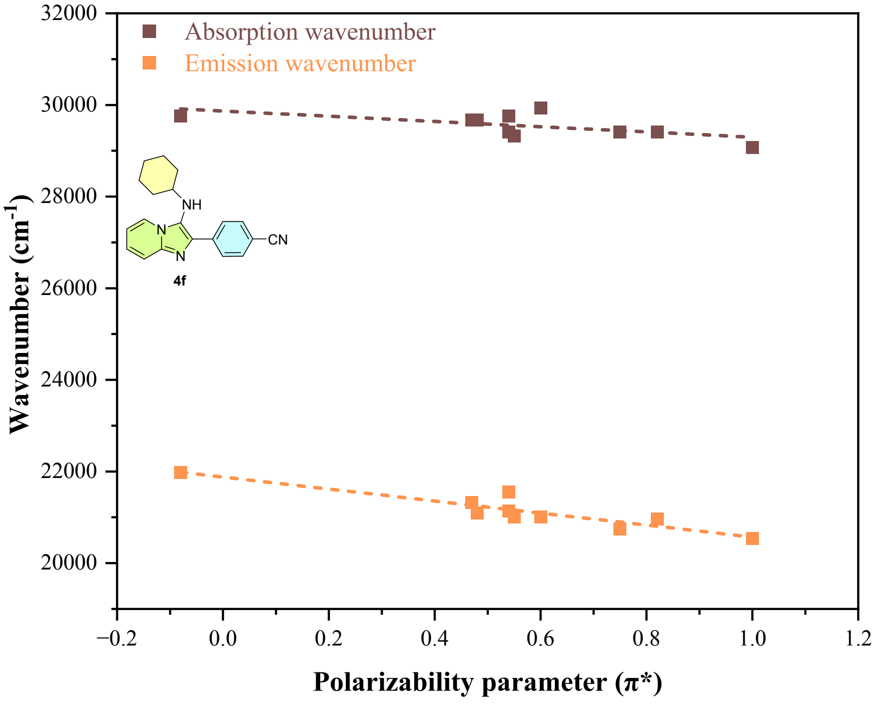

Figure S58. Plot showing polarizability parameter (π*) as a function of absorption and emission wavenumber (cm^{-1}) for compound **4f**.

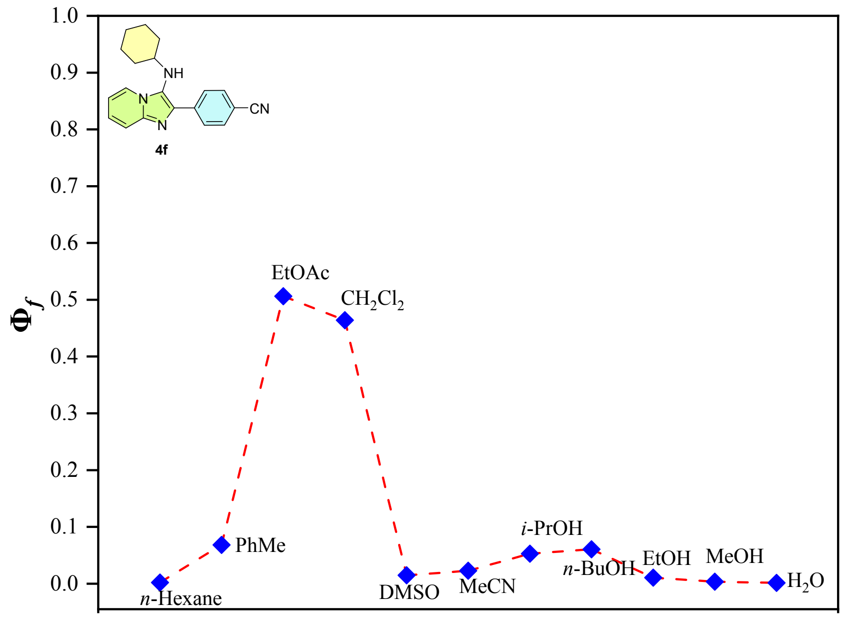

Figure S59. Variation of quantum yields of fluorescence (Φ_f) in different solvents for compound **4f**.

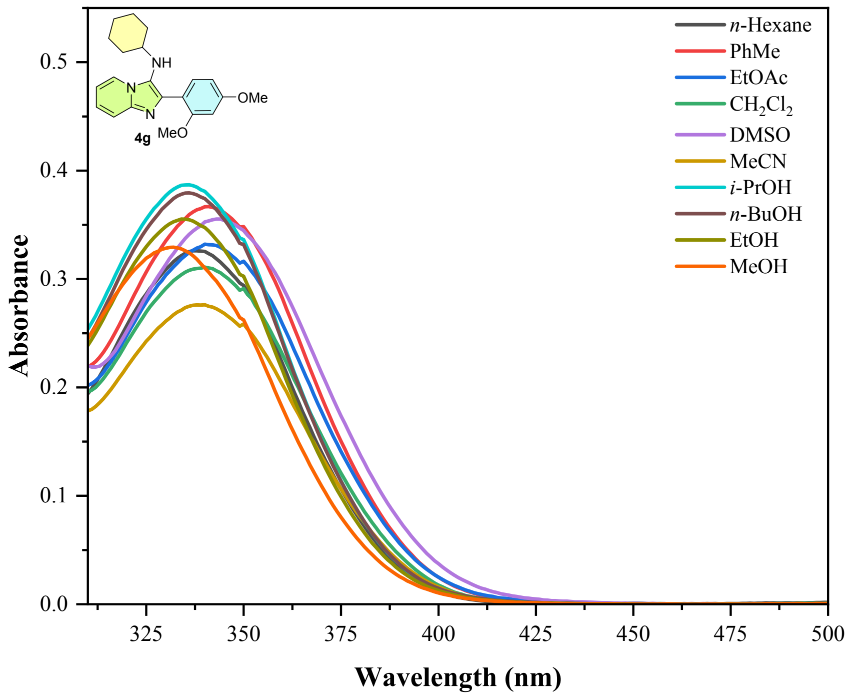

Figure S60. UV-Vis absorption spectra of **4g** in different solvents ($5{\cdot}10^{-5}$ M) at room temperature.

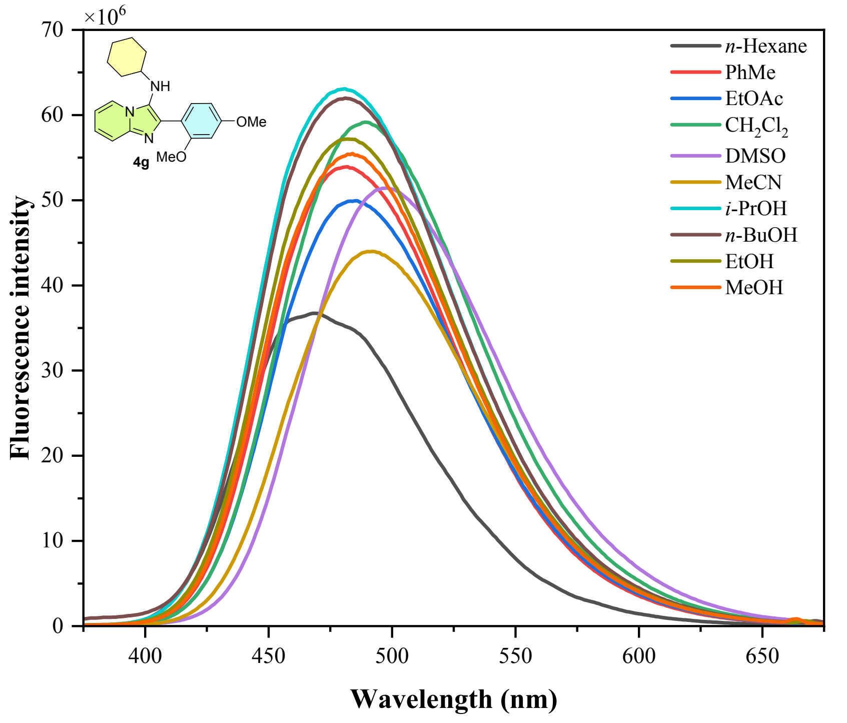

Figure S61. Normalized emission spectra of **4g** in different solvents ($5{\cdot}10^{-5}$ M) at room temperature.

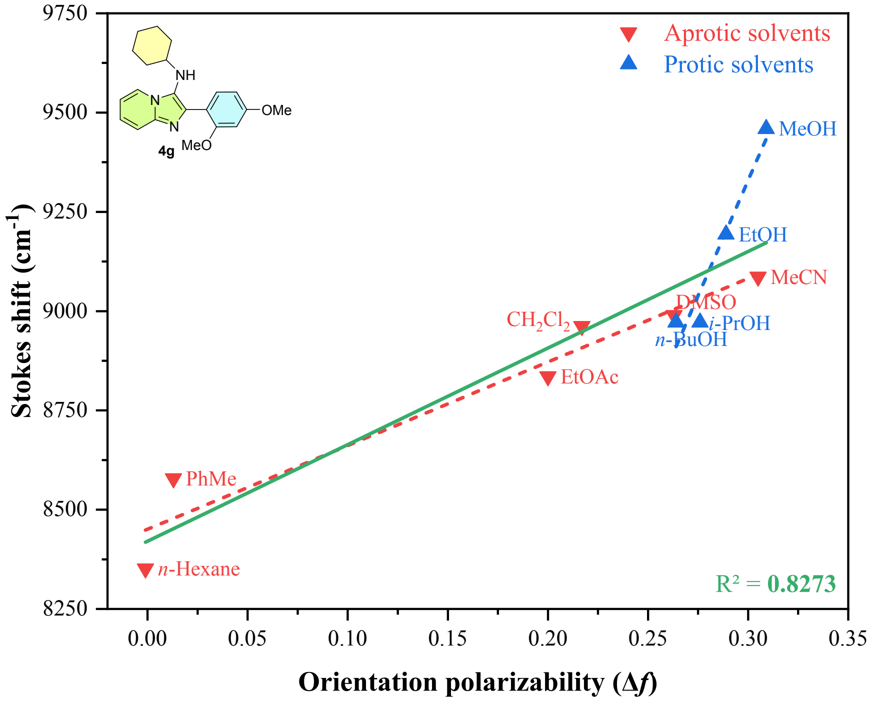

Figure S62. Lippert–Mataga plot showing Stokes shift as a function of solvent orientation polarizability (Δf) for compound **4g**.

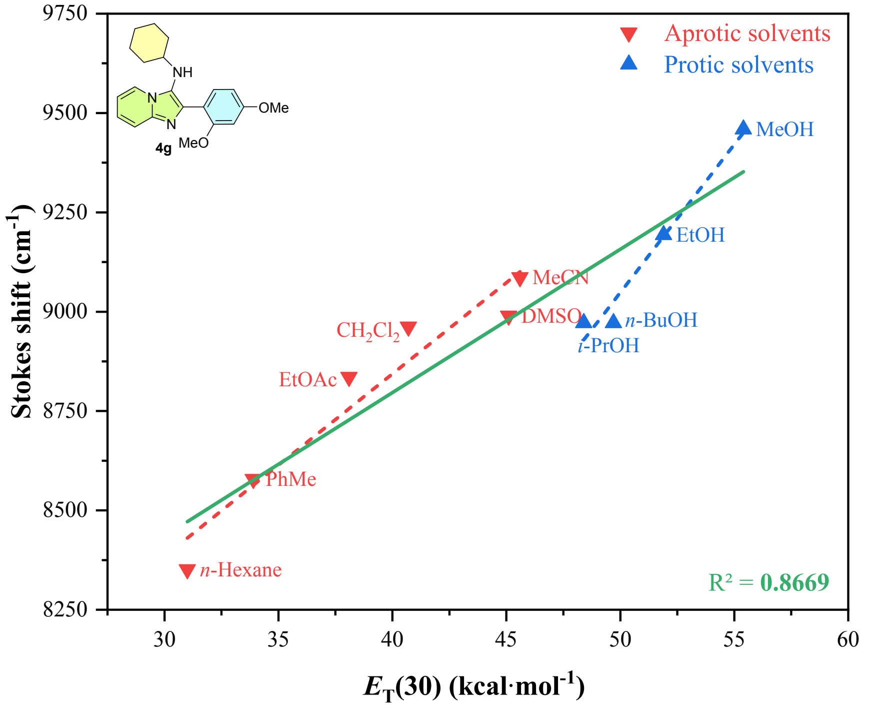

Figure S63. Dimroth-Reichardt plot showing Stokes shift against $E_T(30)$ parameter for compound **4g**.

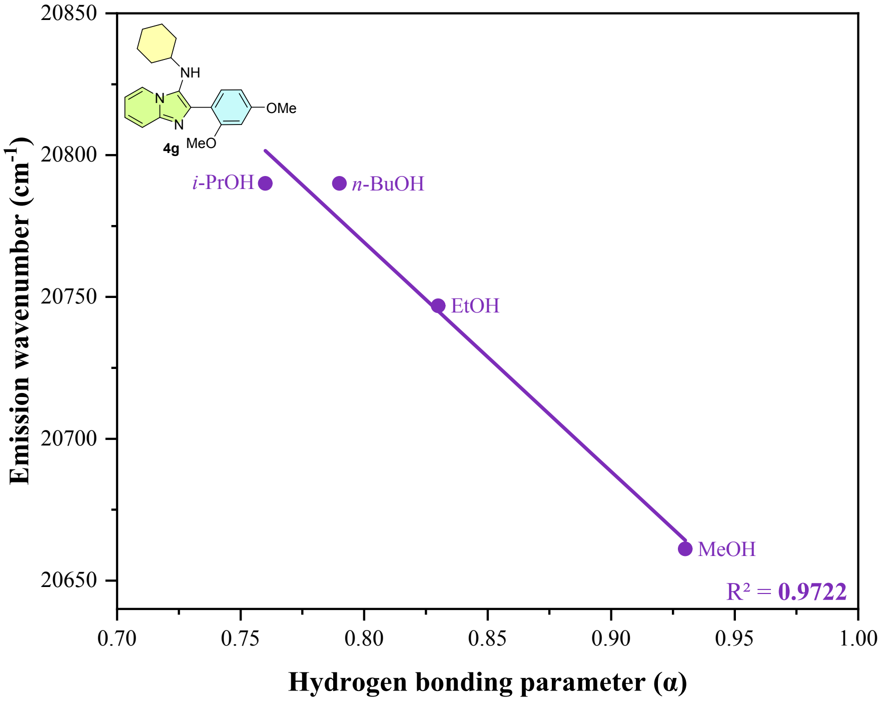

Figure S64. Plot showing hydrogen bonding parameter (α) as a function of emission wavenumber (cm^{-1}) for compound **4g**.

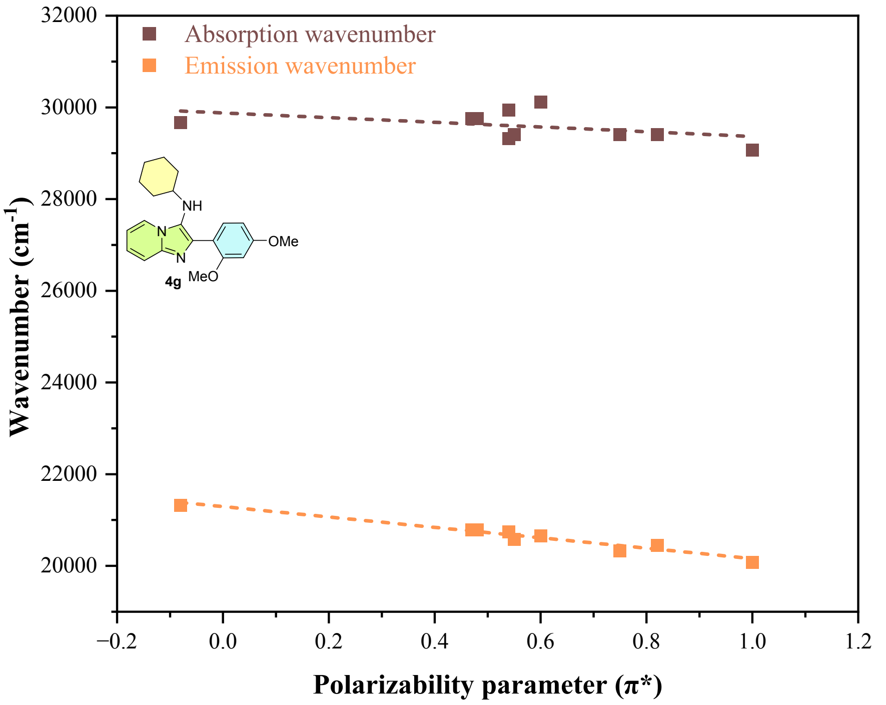

Figure S65. Plot showing polarizability parameter (π*) as a function of absorption and emission wavenumber (cm^{-1}) for compound **4g**.

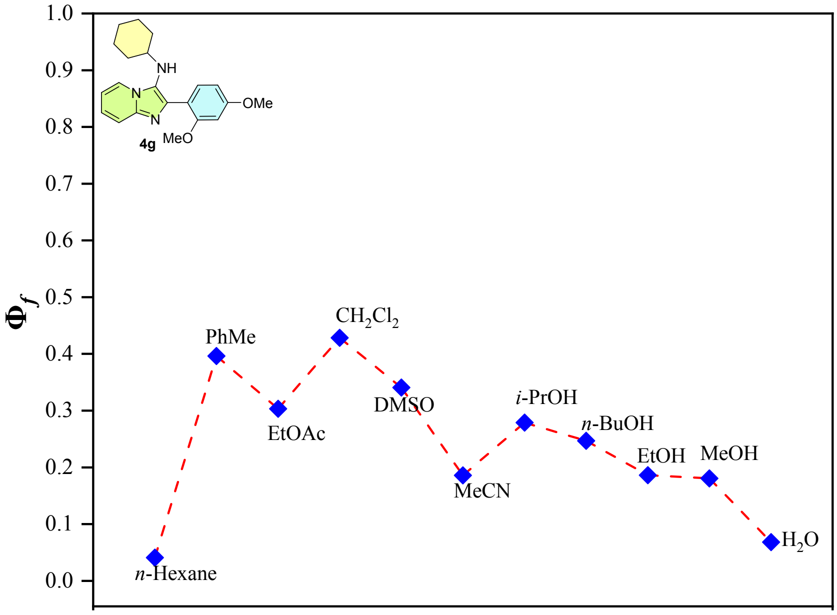

Figure S66. Variation of quantum yields of fluorescence (Φ_f) in different solvents for compound **4g**.

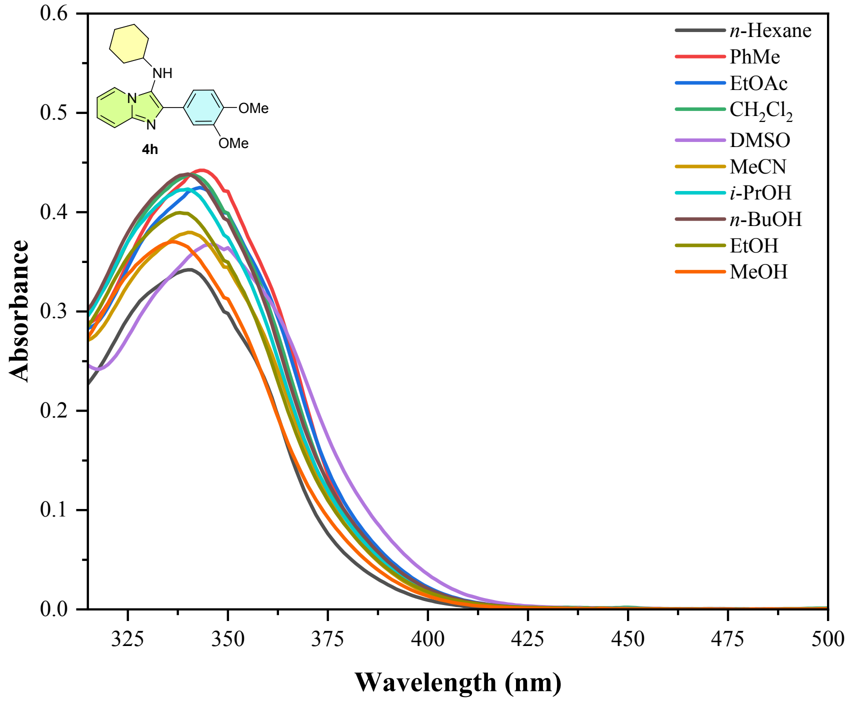

Figure S67. UV-Vis absorption spectra of **4h** in different solvents ($5 \cdot 10^{-5}$ M) at room temperature.

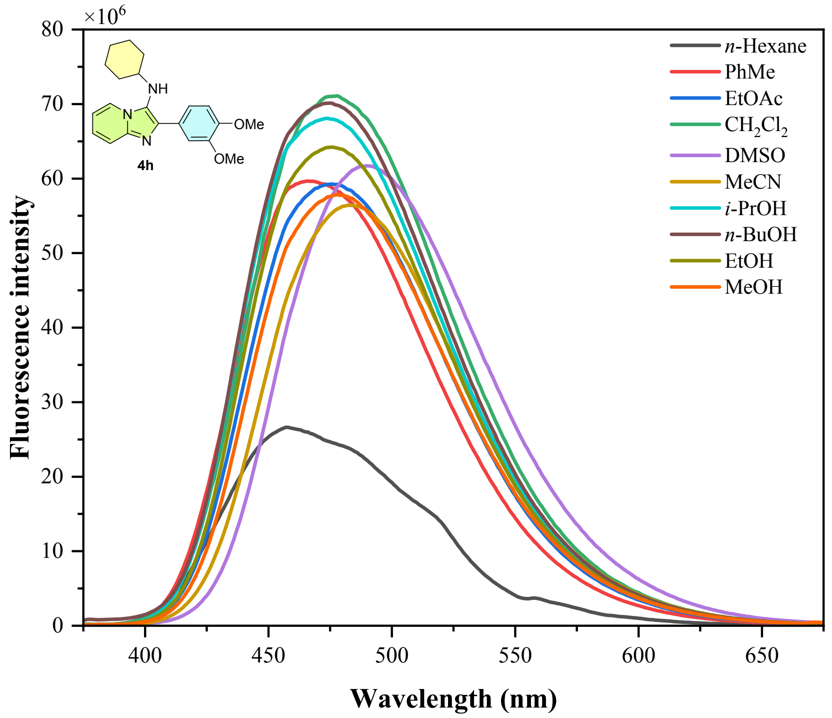

Figure S68. Normalized emission spectra of **4h** in different solvents ($5 \cdot 10^{-5}$ M) at room temperature.

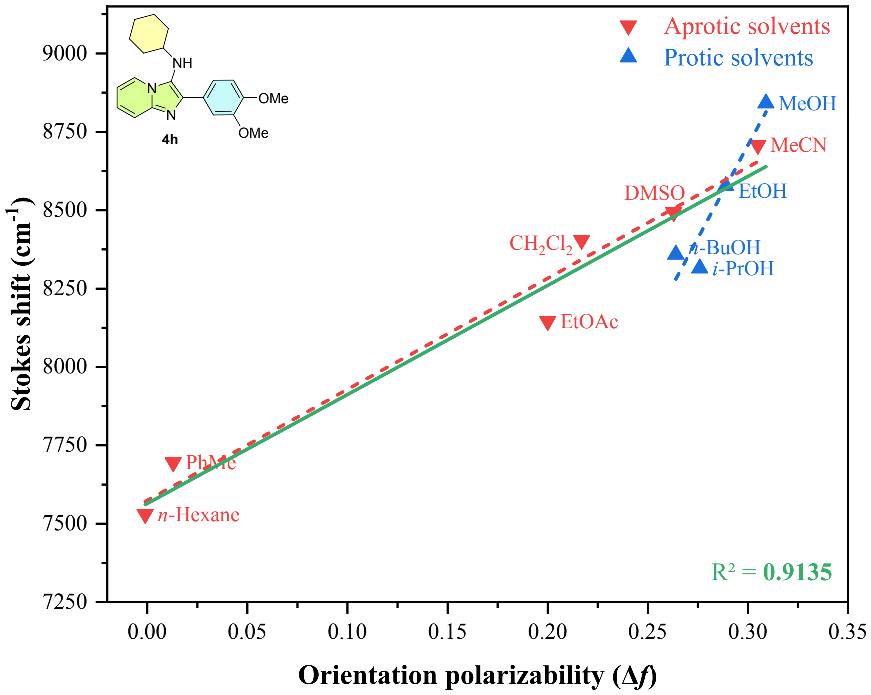

Figure S69. Lippert–Mataga plot showing Stokes shift as a function of solvent orientation polarizability (Δf) for compound **4h**.

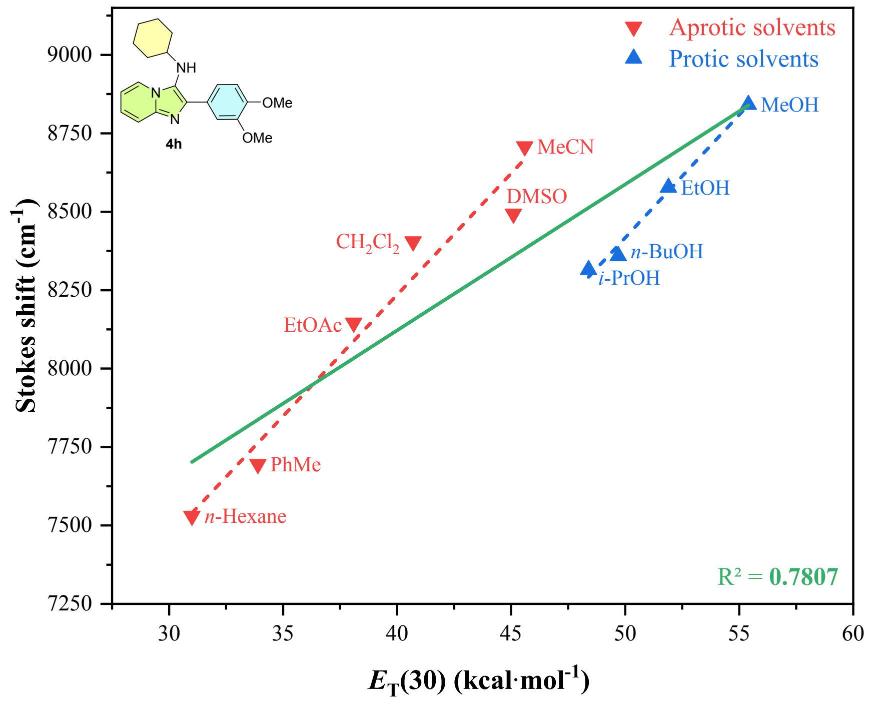

Figure S70. Dimroth-Reichardt plot showing Stokes shift against $E_T(30)$ parameter for compound **4h**.

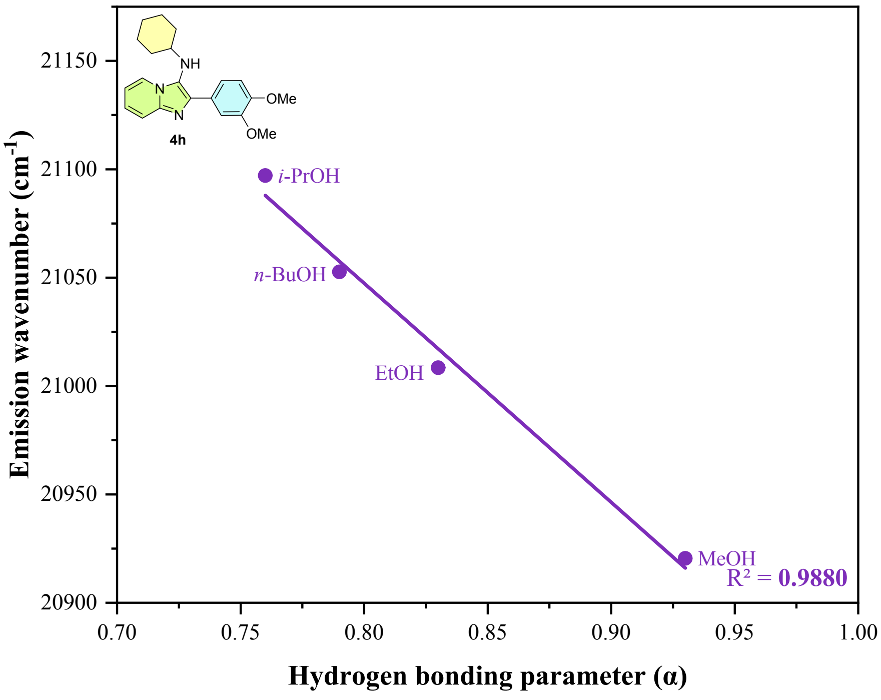

Figure S71. Plot showing hydrogen bonding parameter (α) as a function of emission wavenumber (cm^{-1}) for compound **4h**.

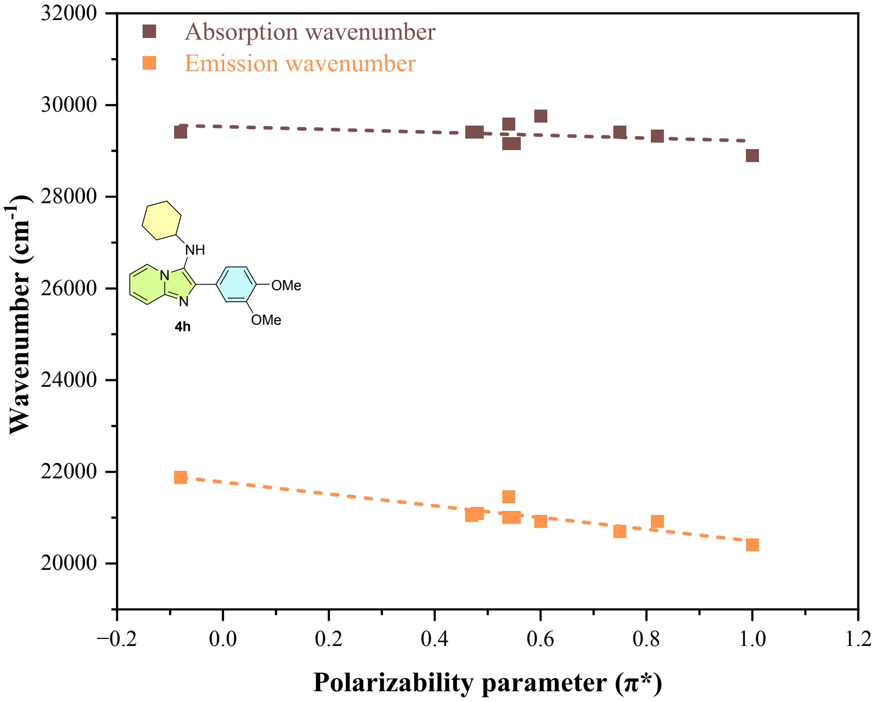

Figure S72. Plot showing polarizability parameter (π*) as a function of absorption and emission wavenumber (cm^{-1}) for compound **4h**.

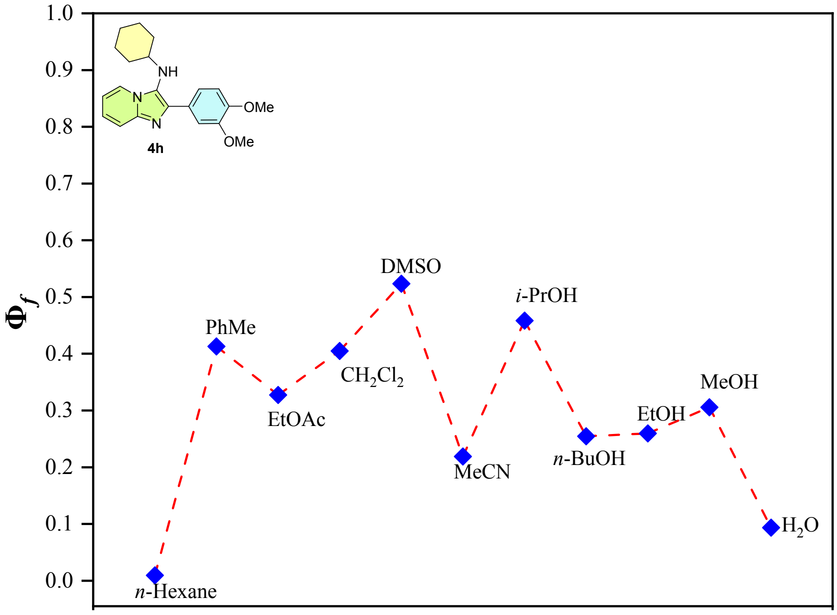

Figure S73. Variation of quantum yields of fluorescence (Φ_f) in different solvents for compound **4h**.

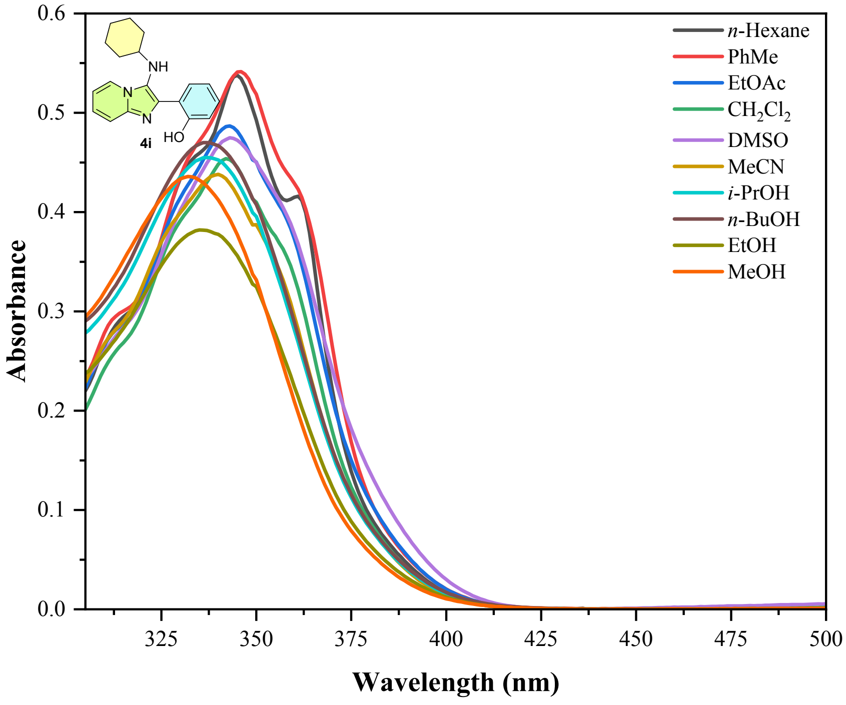

Figure S74. UV-Vis absorption spectra of **4i** in different solvents ($5{\cdot}10^{-5}$ M) at room temperature.

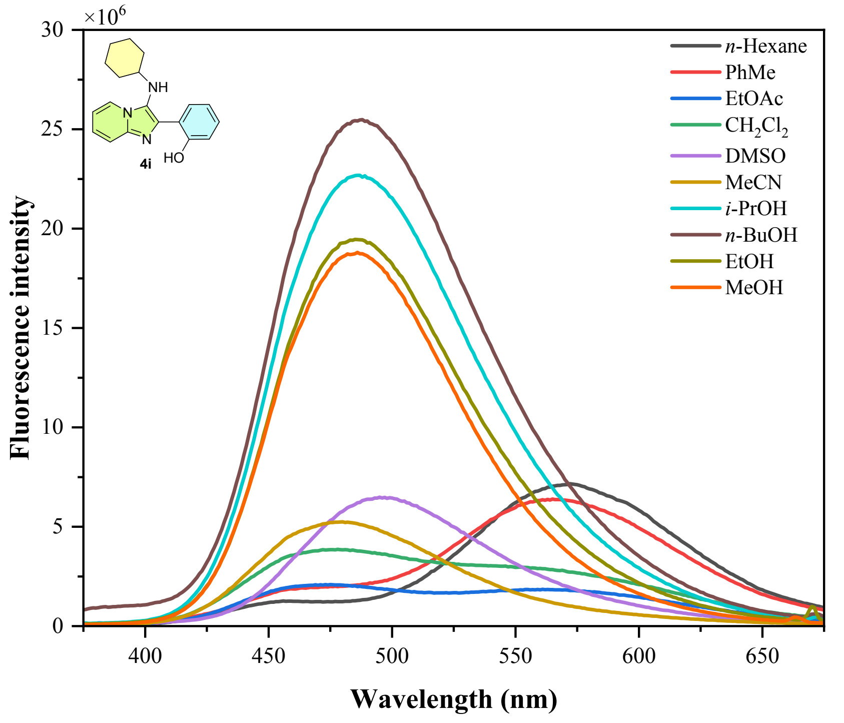

Figure S75. Normalized emission spectra of **4i** in different solvents ($5{\cdot}10^{-5}$ M) at room temperature.

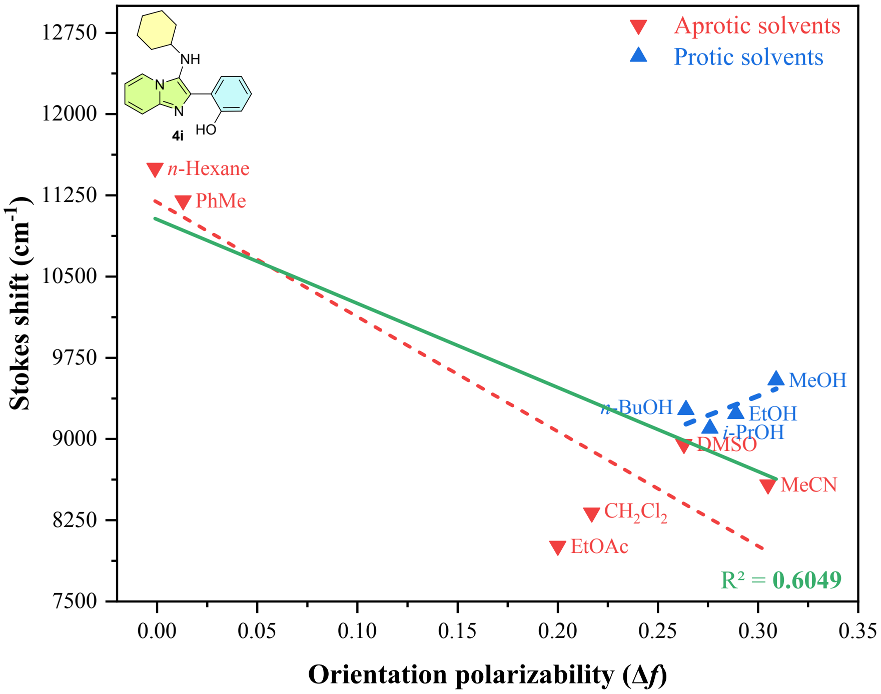

Figure S76. Lippert–Mataga plot showing Stokes shift as a function of solvent orientation polarizability (Δ*f*) for compound **4i**.

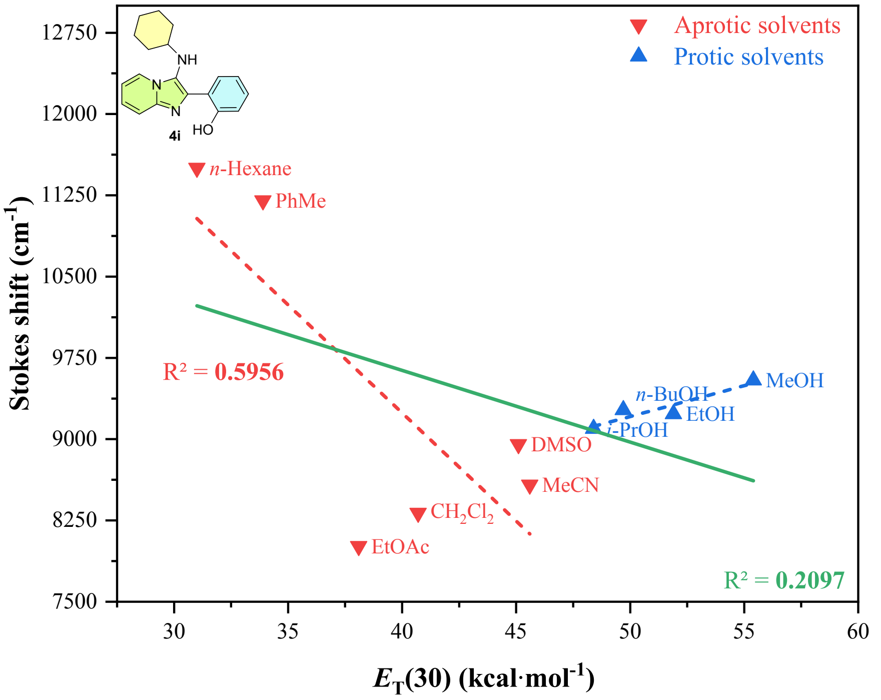

Figure S77. Dimroth-Reichardt plot showing Stokes shift against $E_T(30)$ parameter for compound **4i**.

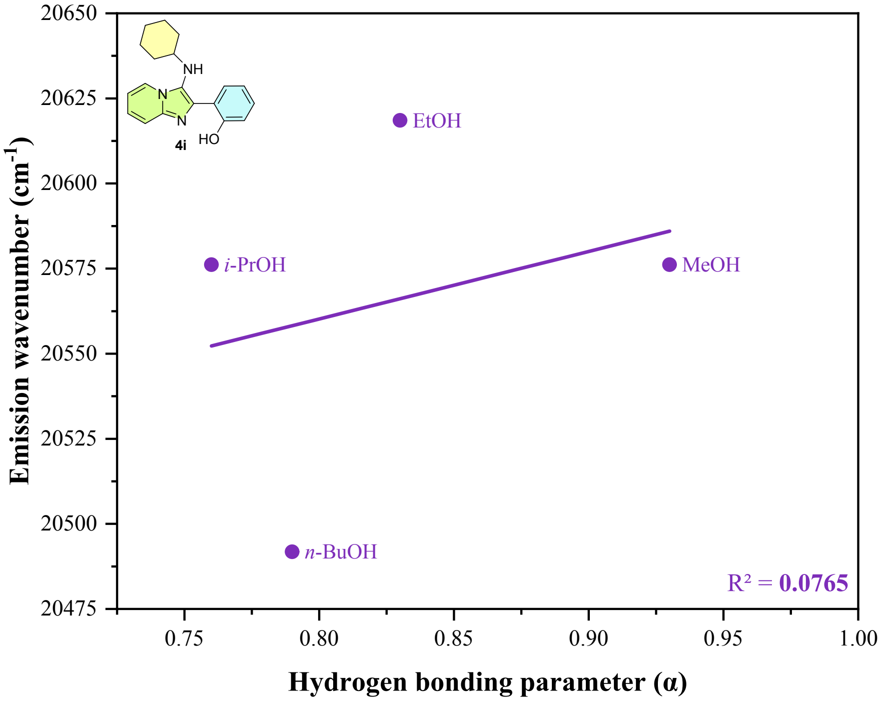

Figure S78. Plot showing hydrogen bonding parameter (α) as a function of emission wavenumber (cm^{-1}) for compound **4i**.

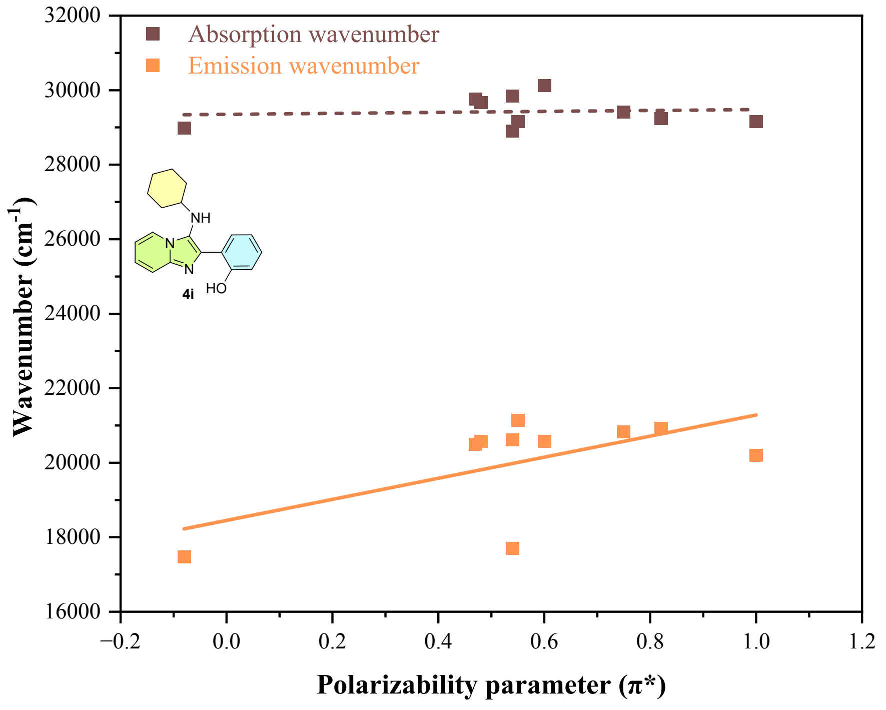

Figure S79. Plot showing polarizability parameter (π*) as a function of absorption and emission wavenumber (cm^{-1}) for compound **4i**.

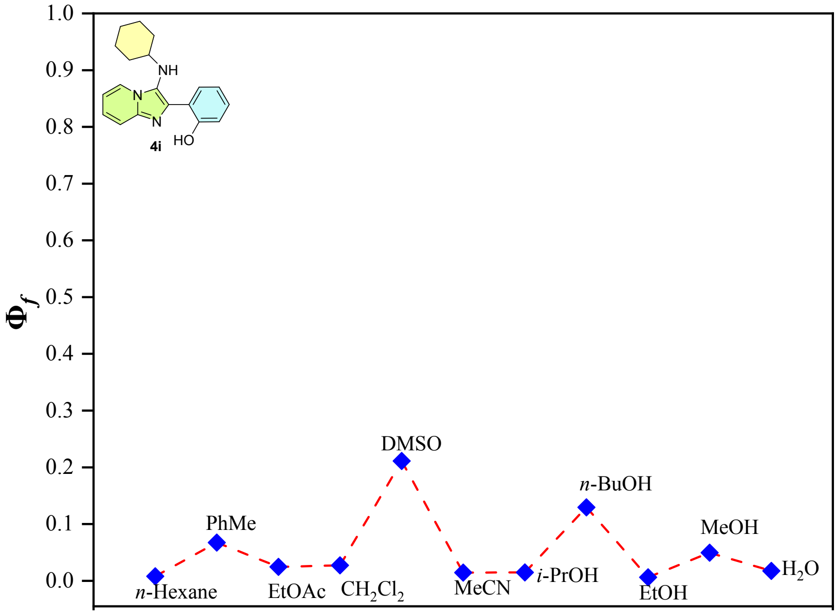

Figure S80. Variation of quantum yields of fluorescence (Φ_f) in different solvents for compound **4i**.

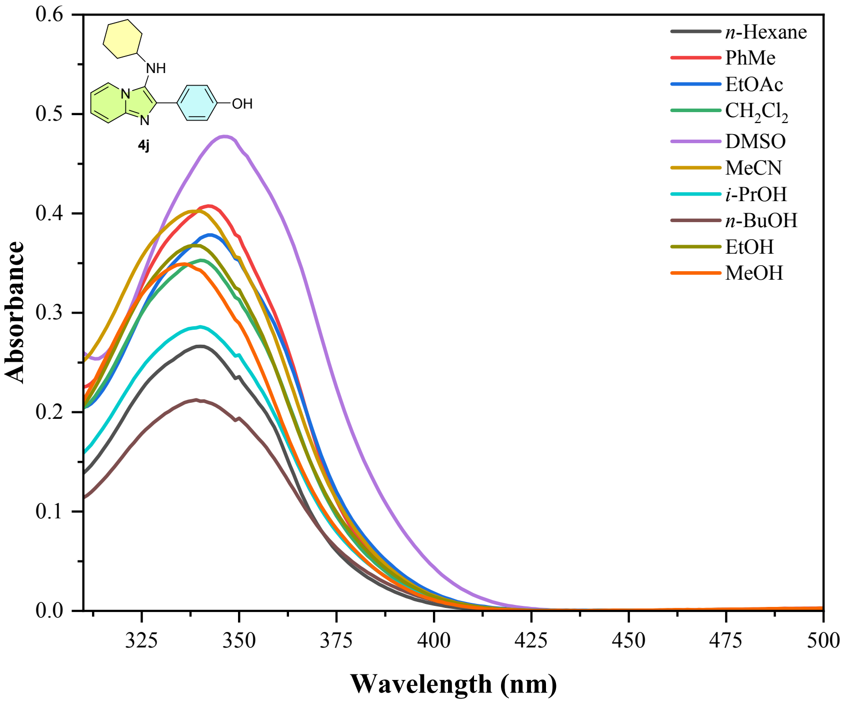

Figure S81. UV-Vis absorption spectra of **4j** in different solvents ($5{\cdot}10^{-5}$ M) at room temperature.

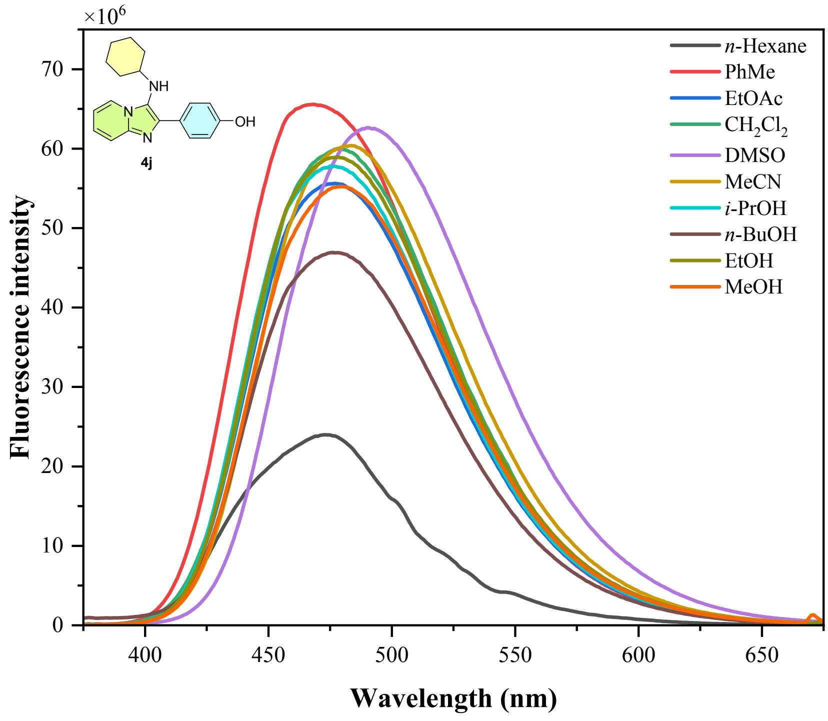

Figure S82. Normalized emission spectra of **4j** in different solvents ($5 \cdot 10^{-5}$ M) at room temperature.

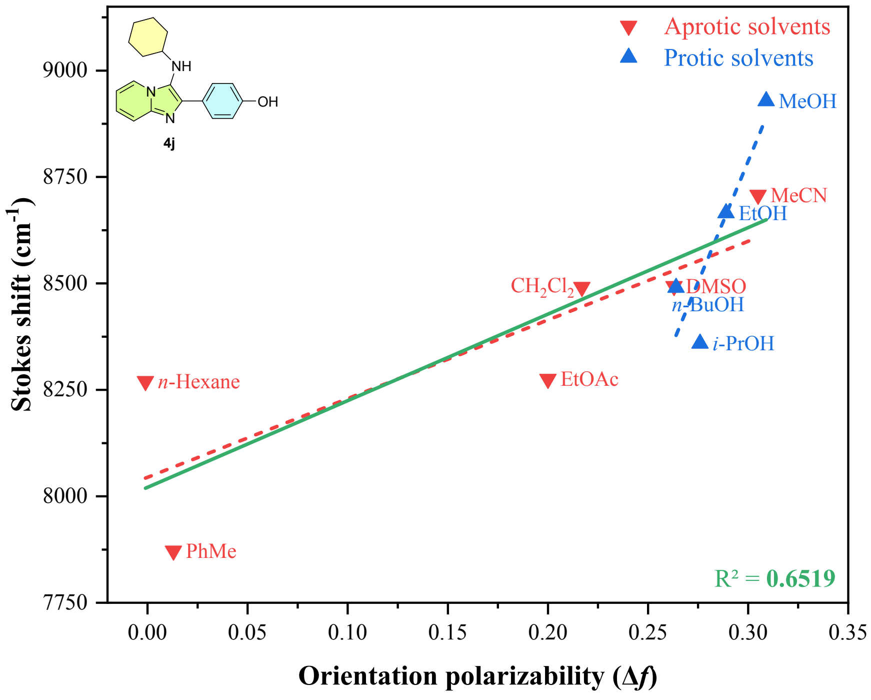

Figure S83. Lippert–Mataga plot showing Stokes shift as a function of solvent orientation polarizability (Δf) for compound **4j**.

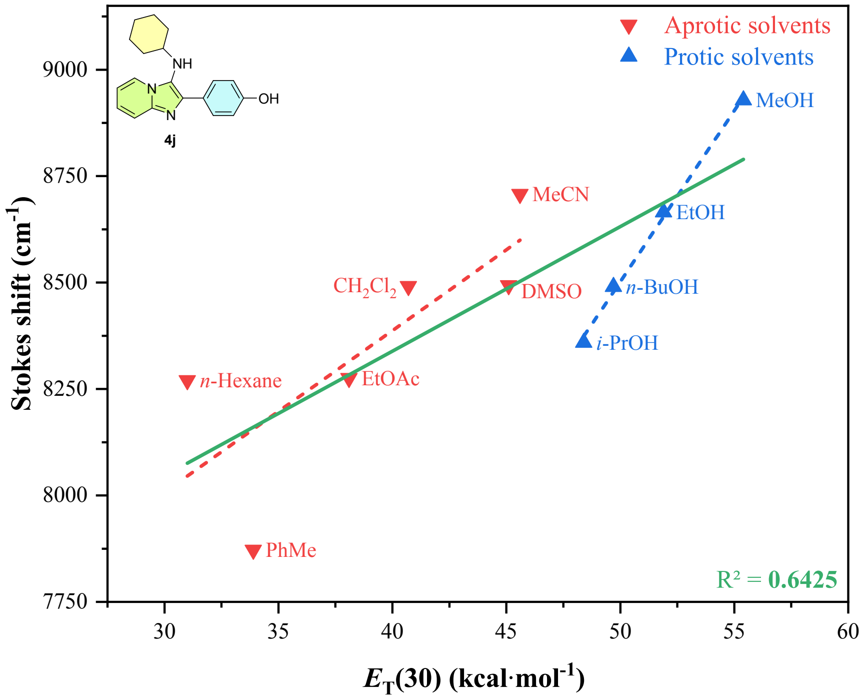

Figure S84. Dimroth-Reichardt plot showing Stokes shift against $E_T(30)$ parameter for compound **4j**.

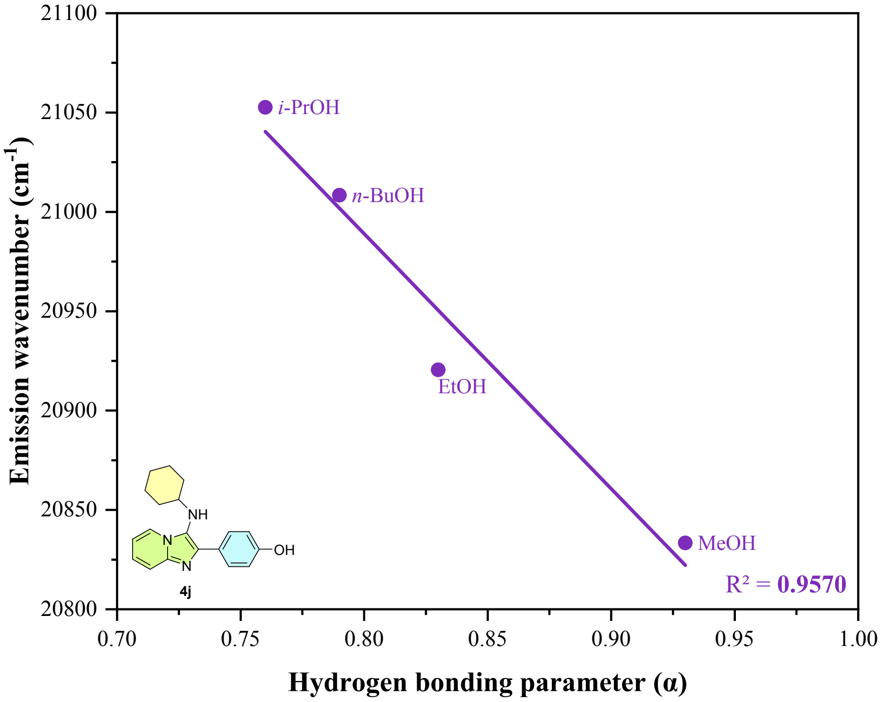

Figure S85. Plot showing hydrogen bonding parameter (α) as a function of emission wavenumber (cm^{-1}) for compound **4j**.

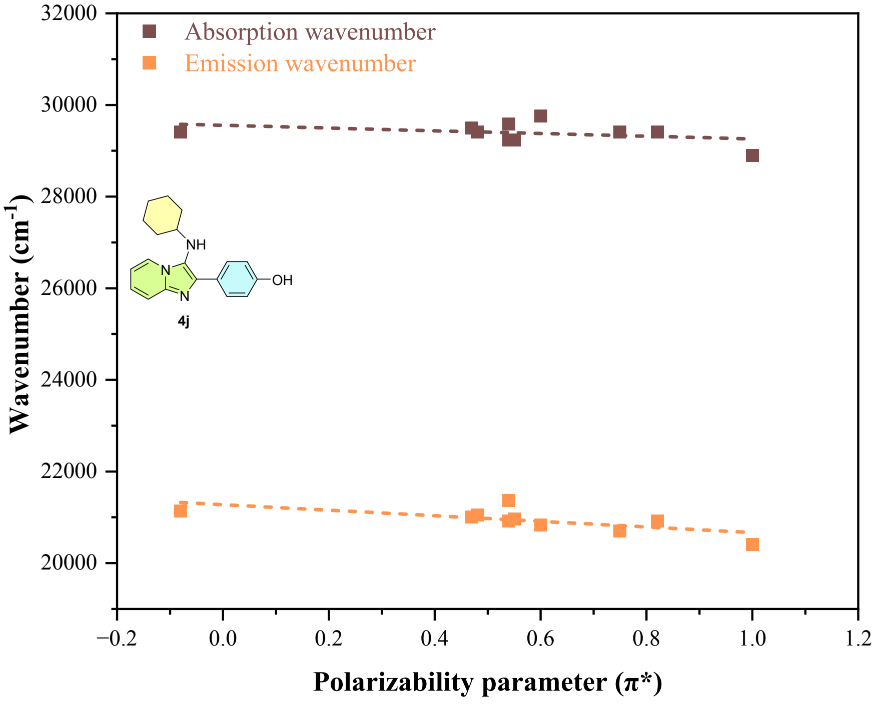

Figure S86. Plot showing polarizability parameter (π^*) as a function of absorption and emission wavenumber (cm^{-1}) for compound **4j**.

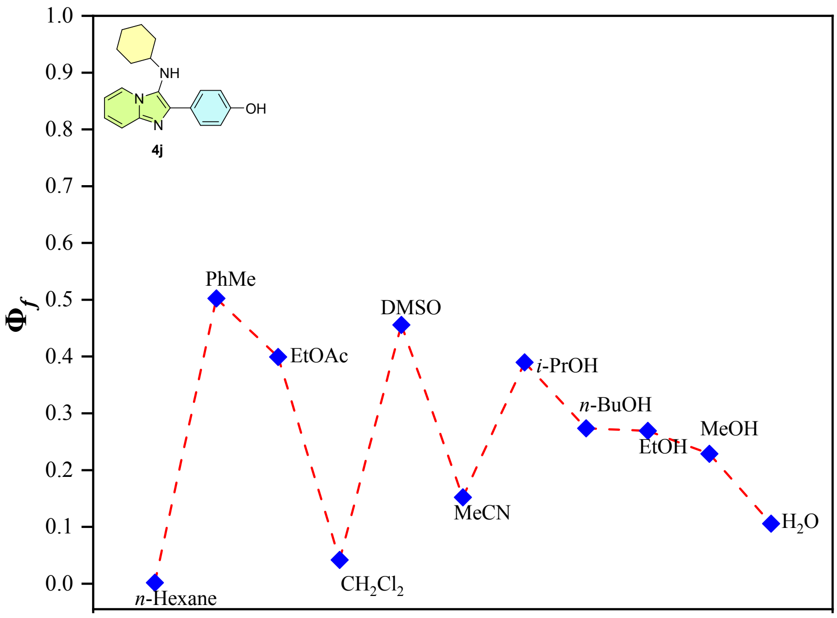

Figure S87. Variation of quantum yields of fluorescence (Φ_f) in different solvents for compound **4j**.

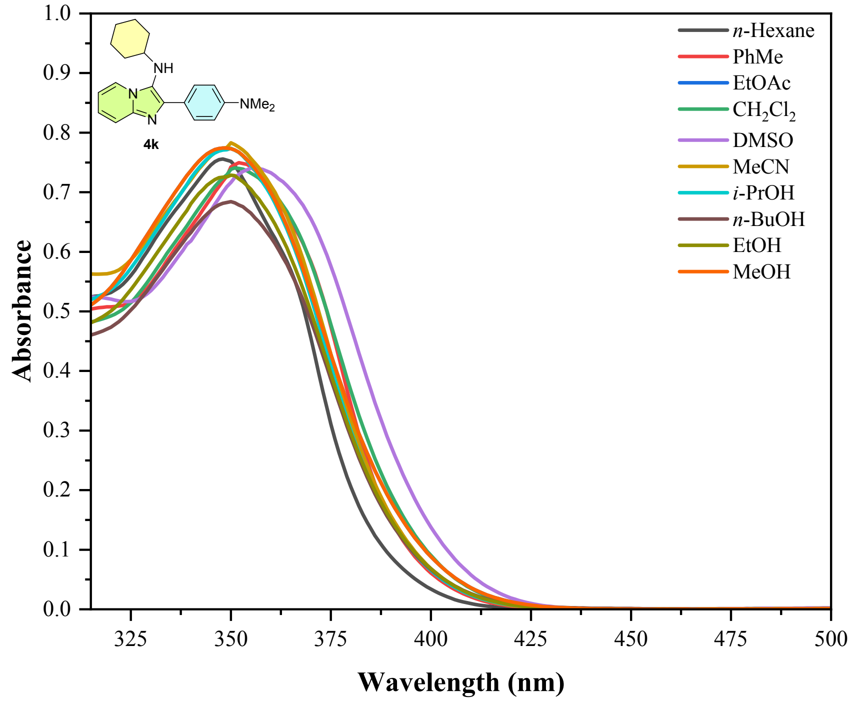

Figure S88. UV-Vis absorption spectra of **4k** in different solvents ($5{\cdot}10^{-5}$ M) at room temperature.

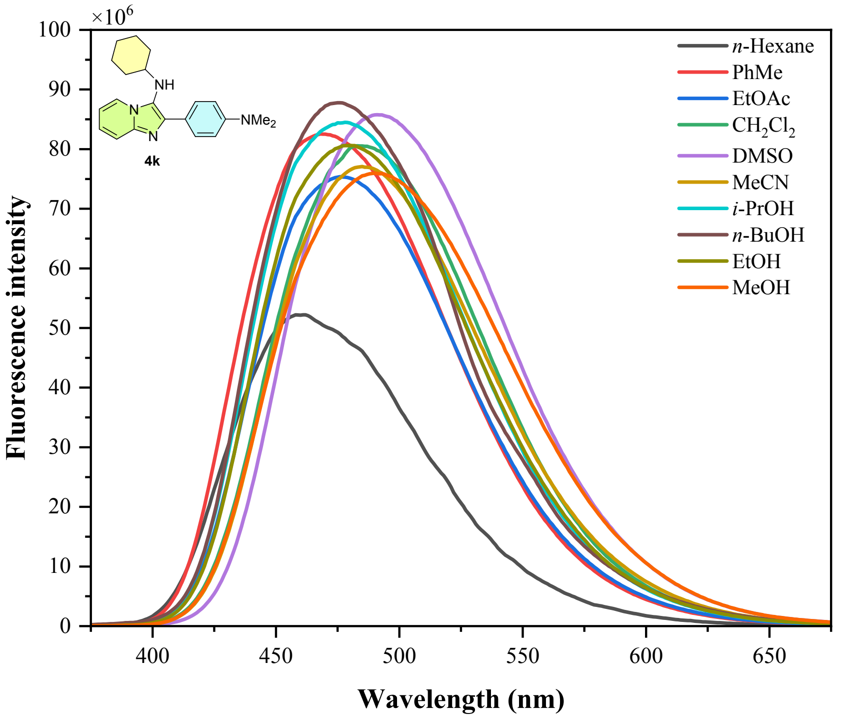

Figure S89. Normalized emission spectra of **4k** in different solvents ($5{\cdot}10^{-5}$ M) at room temperature.

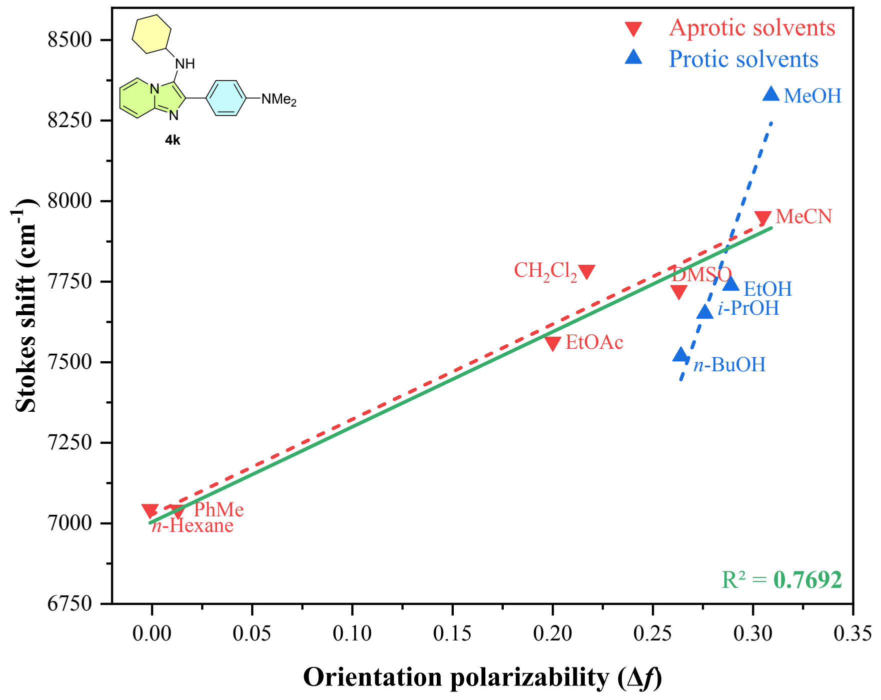

Figure S90. Lippert–Mataga plot showing Stokes shift as a function of solvent orientation polarizability (Δf) for compound **4k**.

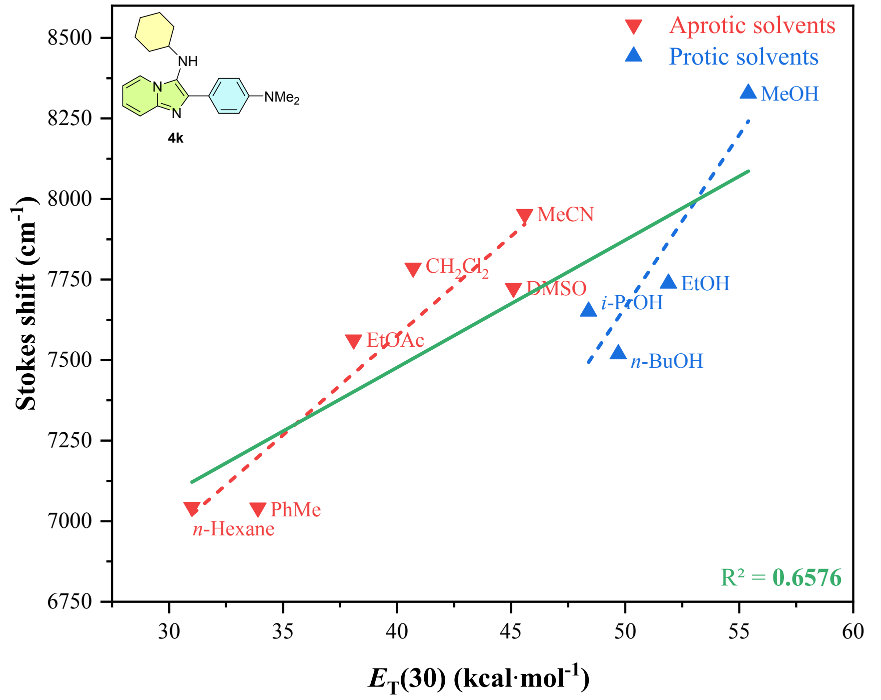

Figure S91. Dimroth-Reichardt plot showing Stokes shift against $E_T(30)$ parameter for compound **4k**.

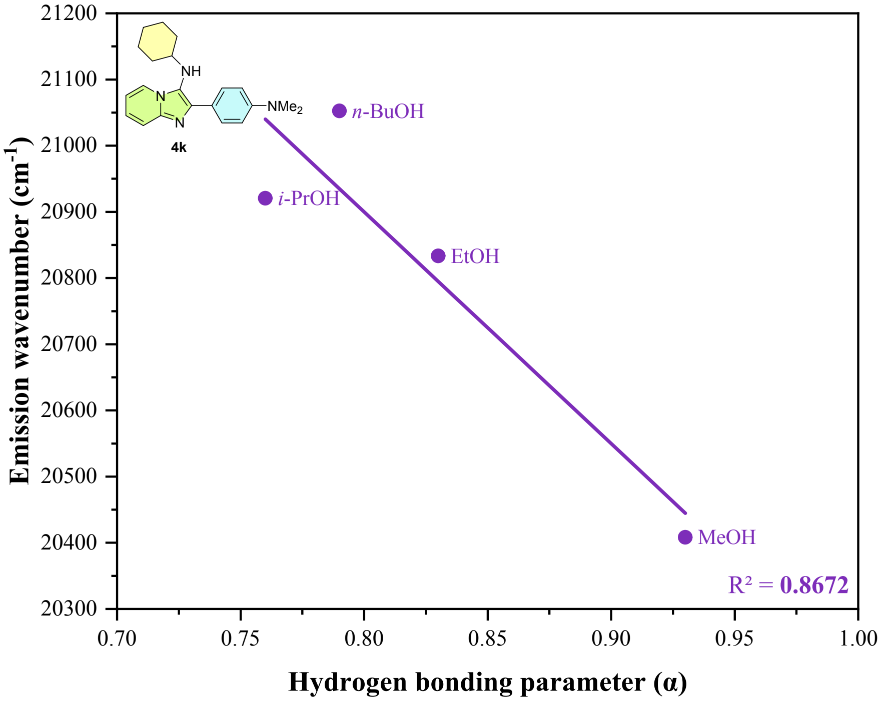

Figure S92. Plot showing hydrogen bonding parameter (α) as a function of emission wavenumber (cm^{-1}) for compound **4k**.

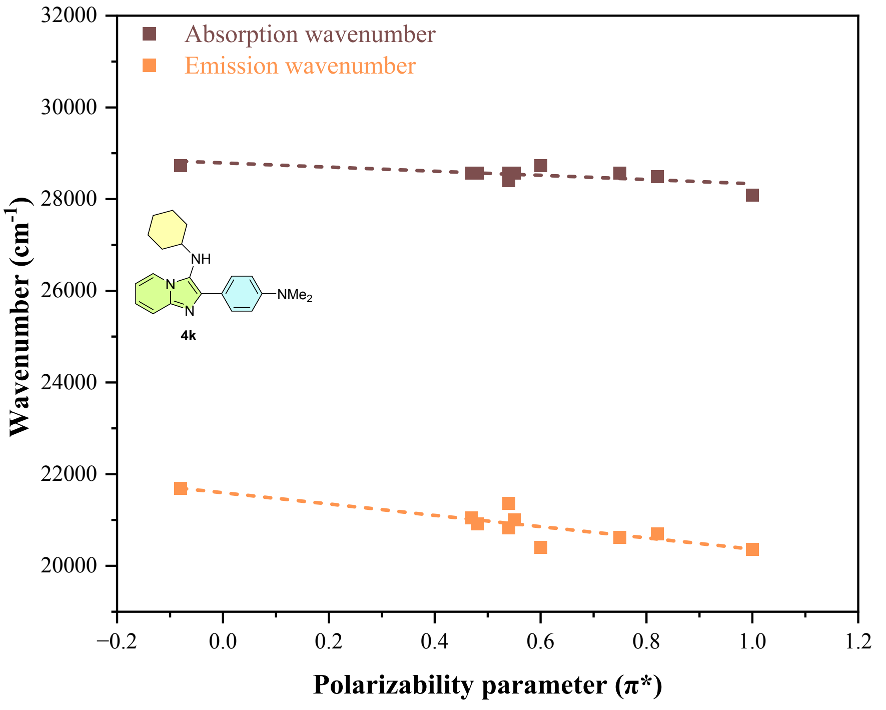

Figure S93. Plot showing polarizability parameter (π*) as a function of absorption and emission wavenumber (cm^{-1}) for compound **4k**.

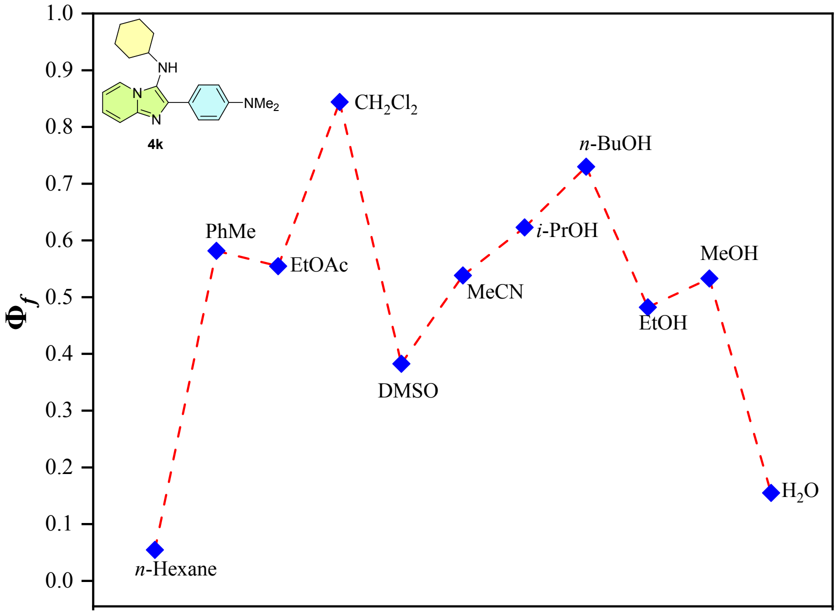

Figure S94. Variation of quantum yields of fluorescence (Φ_f) in different solvents for compound **4k**.

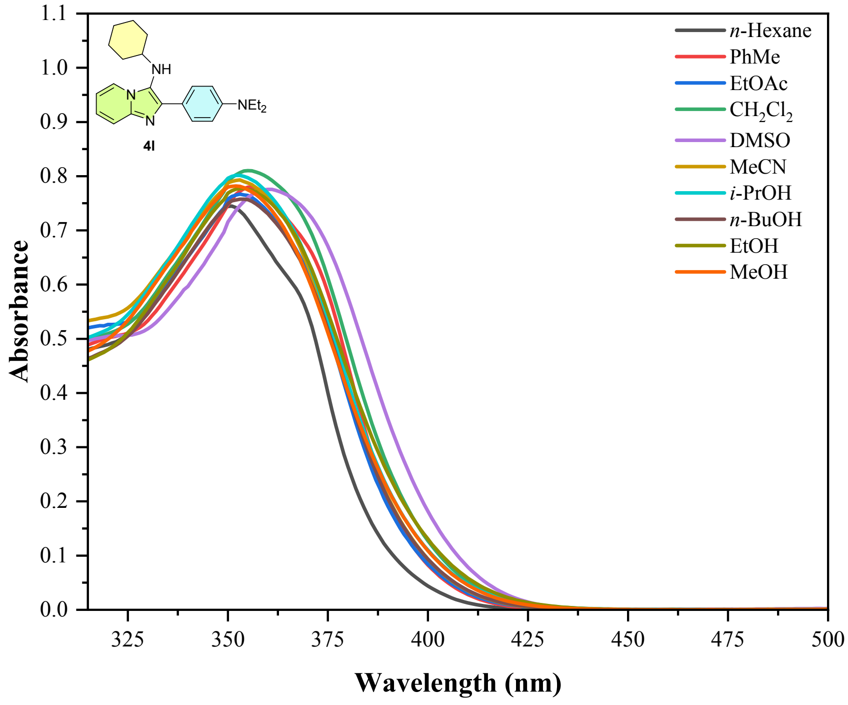

Figure S95. UV-Vis absorption spectra of **4l** in different solvents ($5 \cdot 10^{-5}$ M) at room temperature.

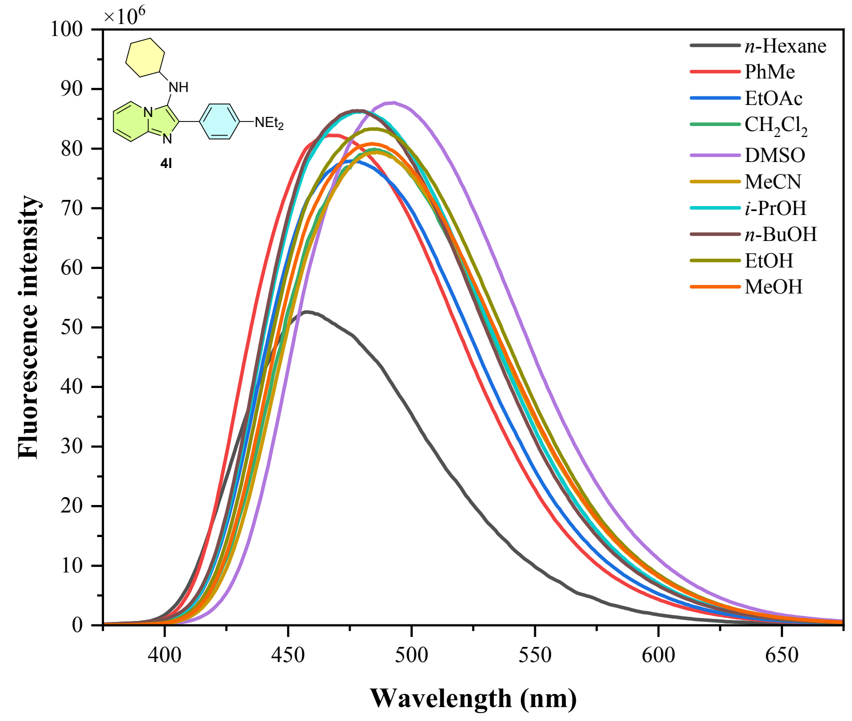

Figure S96. Normalized emission spectra of **4l** in different solvents (5·10^{-5} M) at room temperature.

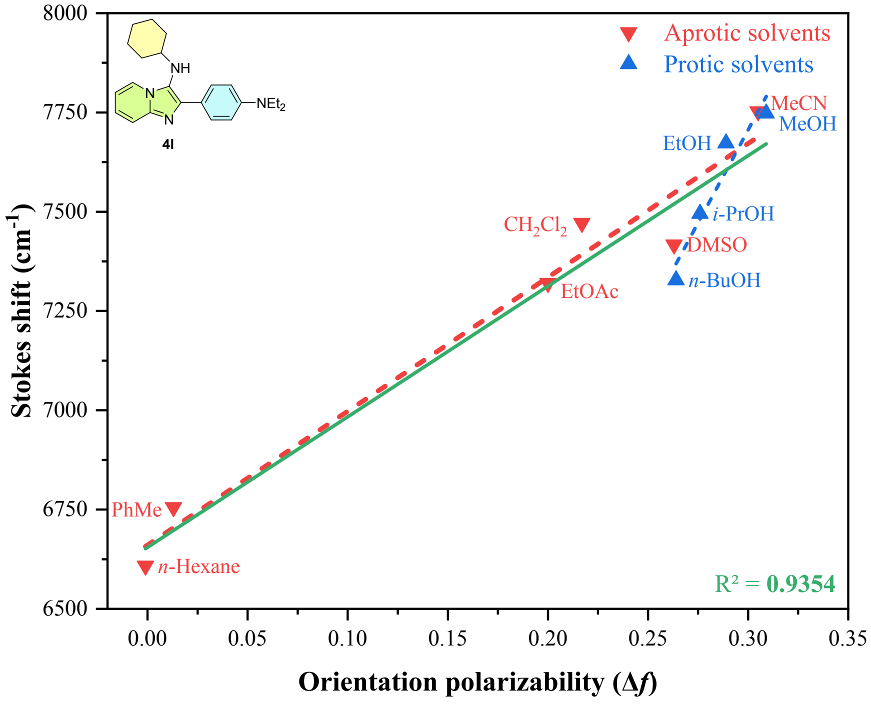

Figure S97. Lippert–Mataga plot showing Stokes shift as a function of solvent orientation polarizability (Δ*f*) for compound **4l**.

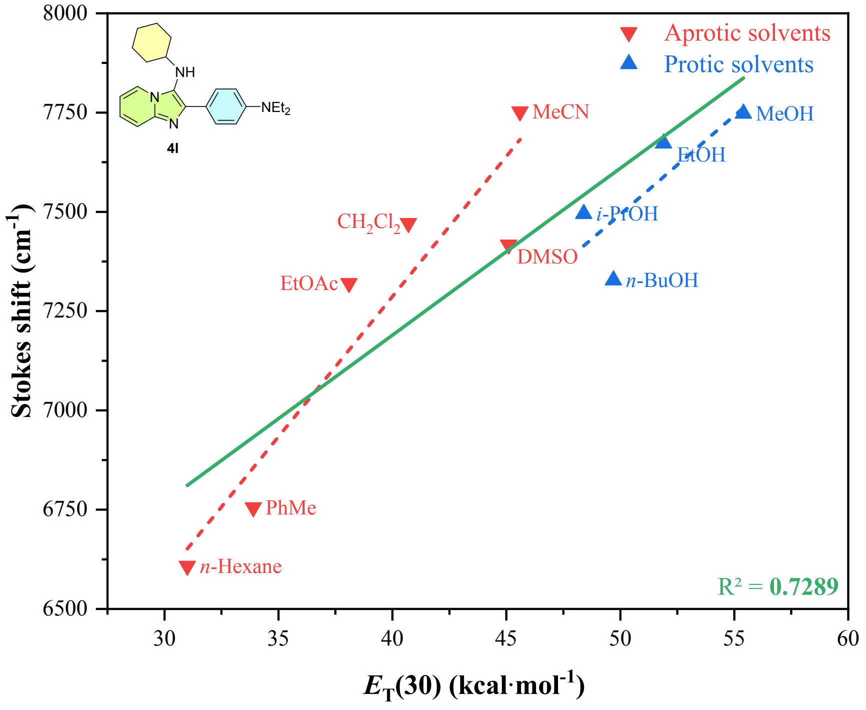

Figure S98. Dimroth-Reichardt plot showing Stokes shift against $E_T(30)$ parameter for compound **4l**.

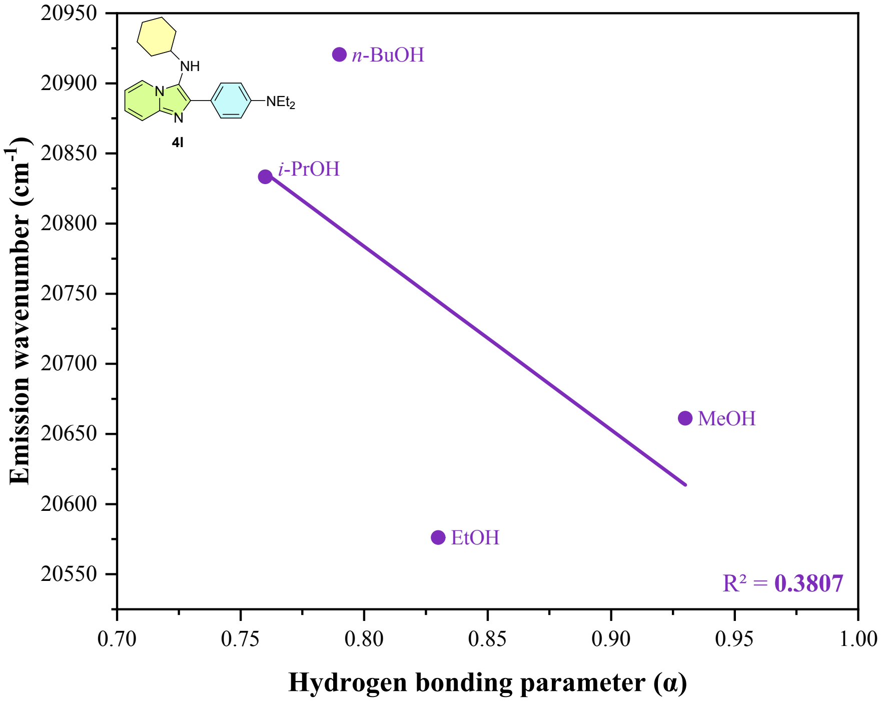

Figure S99. Plot showing hydrogen bonding parameter (α) as a function of emission wavenumber (cm^{-1}) for compound **4l**.

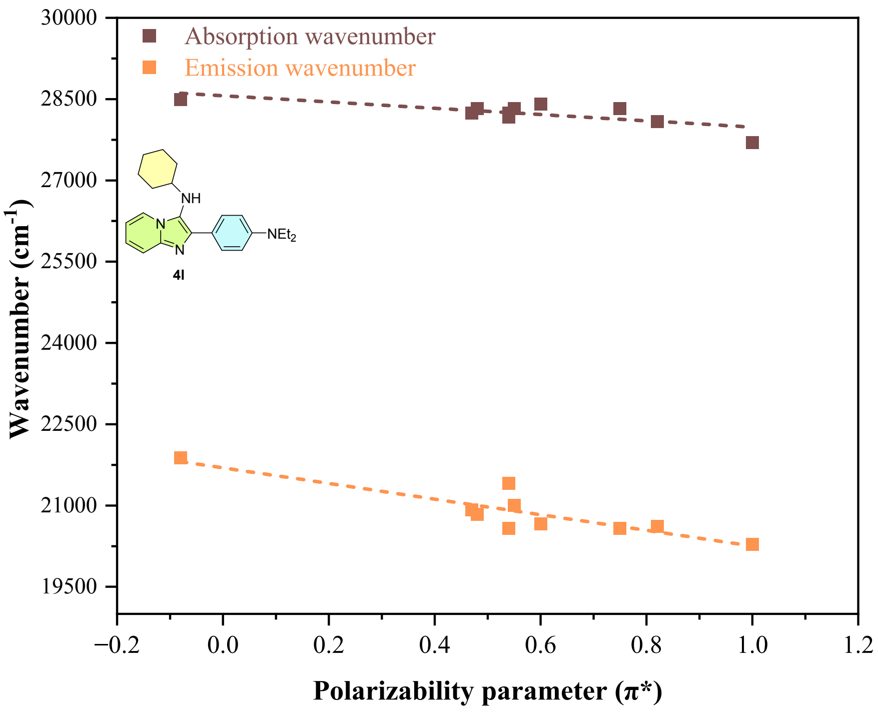

Figure S100. Plot showing polarizability parameter (π*) as a function of absorption and emission wavenumber (cm^{-1}) for compound **4l**.

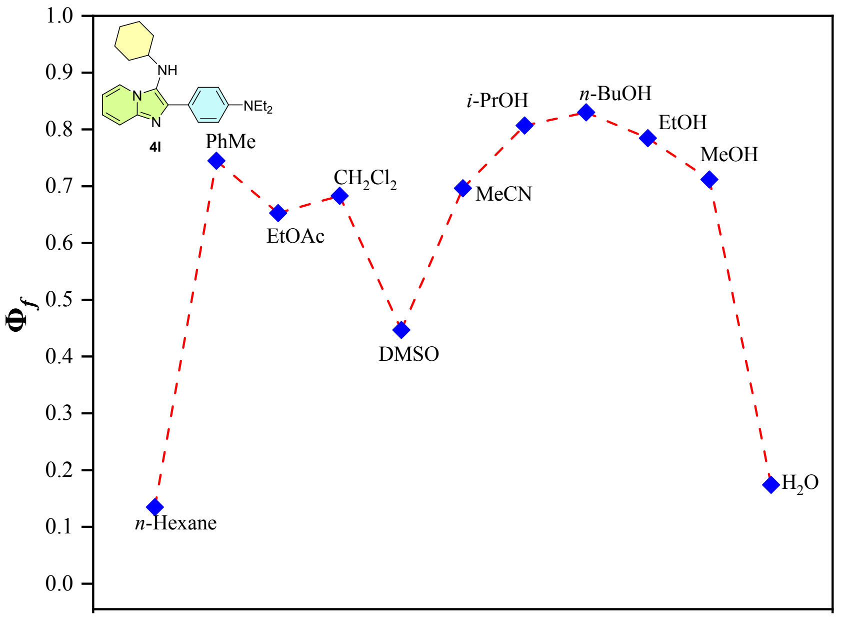

Figure S101. Variation of quantum yields of fluorescence (Φ_f) in different solvents for compound **4l**.

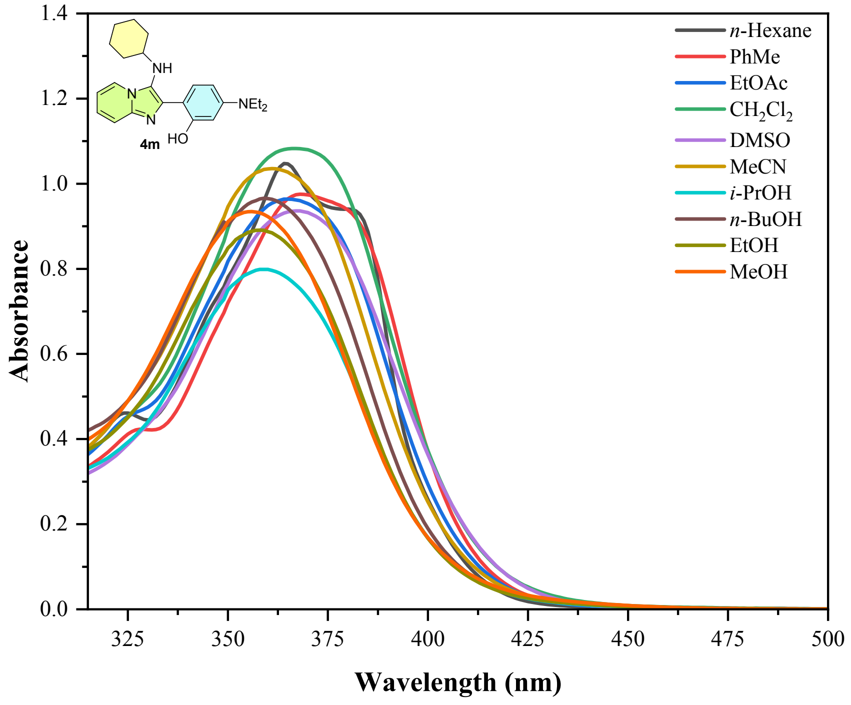

Figure S102. UV-Vis absorption spectra of **4m** in different solvents ($5 \cdot 10^{-5}$ M) at room temperature.

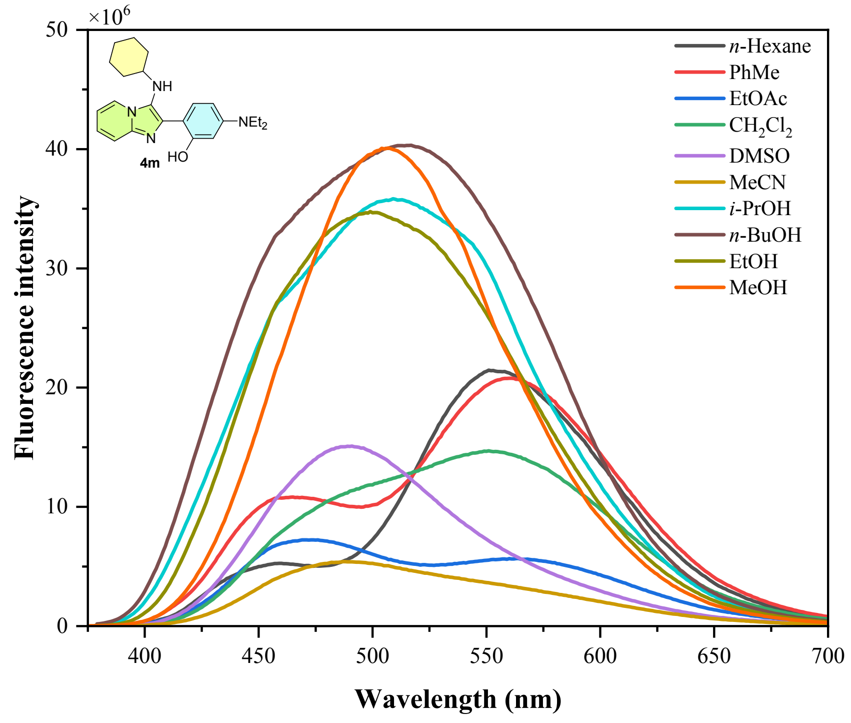

Figure S103. Normalized emission spectra of **4m** in different solvents ($5 \cdot 10^{-5}$ M) at room temperature.

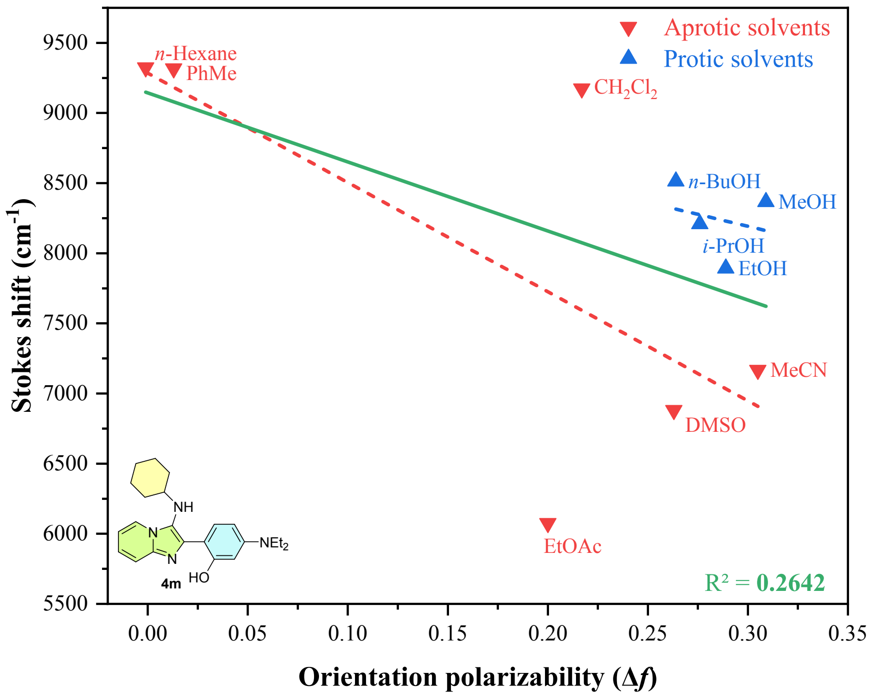

Figure S104. Lippert–Mataga plot showing Stokes shift as a function of solvent orientation polarizability (Δ*f*) for compound **4m**.

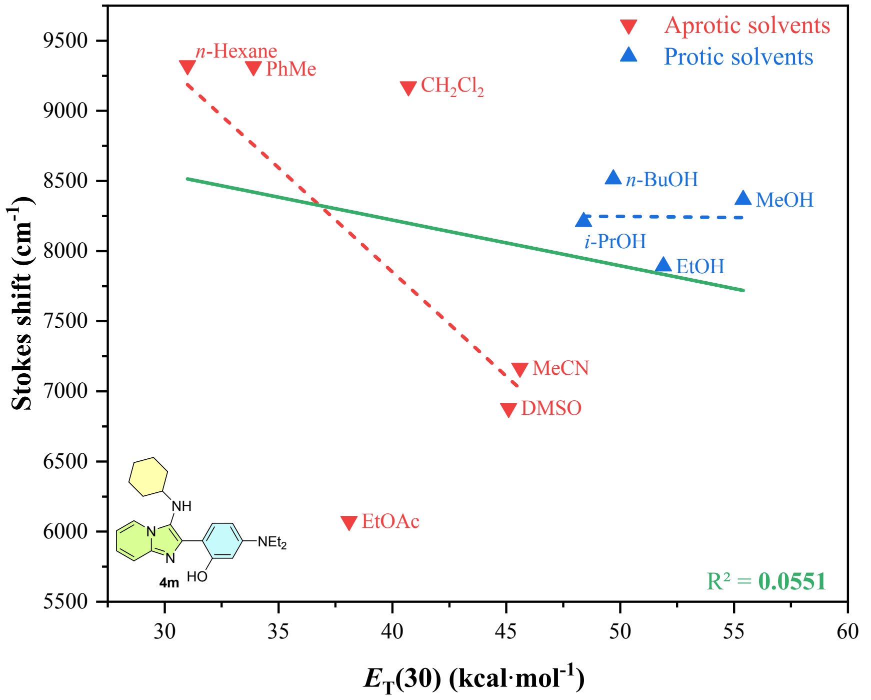

Figure S105. Dimroth-Reichardt plot showing Stokes shift against $E_T(30)$ parameter for compound **4m**.

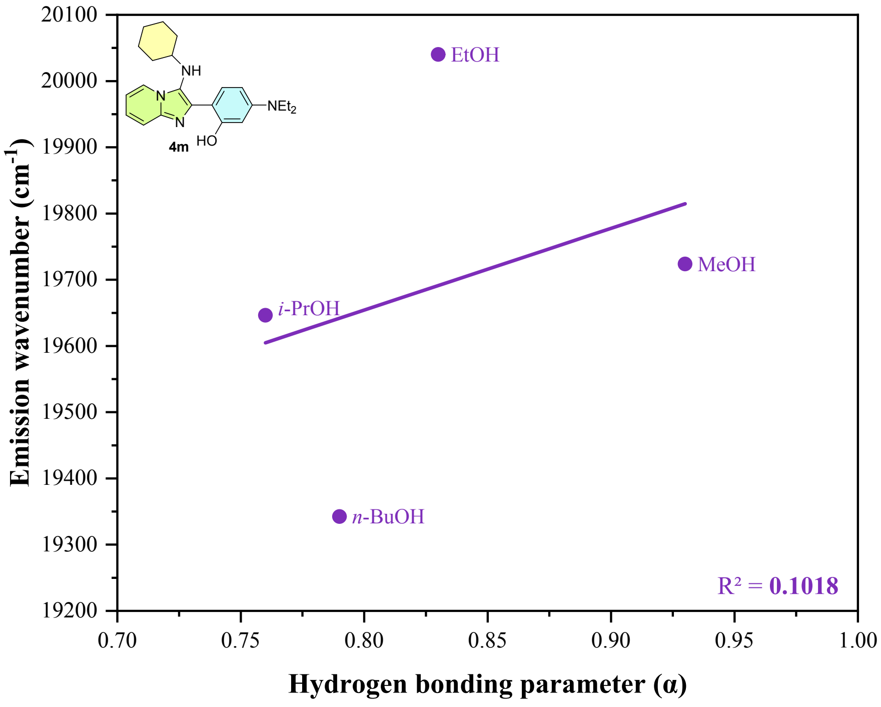

Figure S106. Plot showing hydrogen bonding parameter (α) as a function of emission wavenumber (cm^{-1}) for compound **4m**.

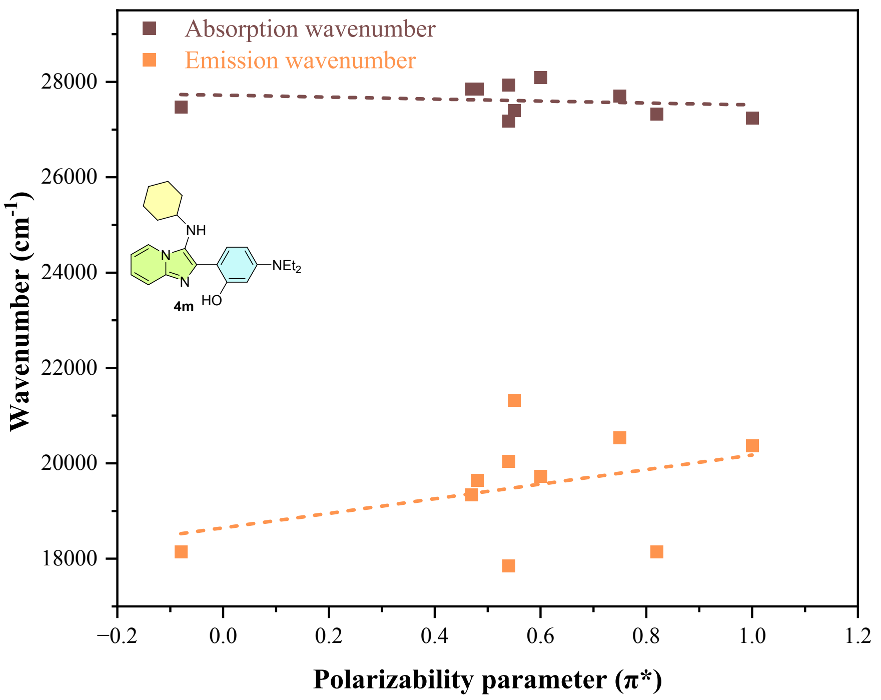

Figure S107. Plot showing polarizability parameter (π*) as a function of absorption and emission wavenumber (cm^{-1}) for compound **4m**.

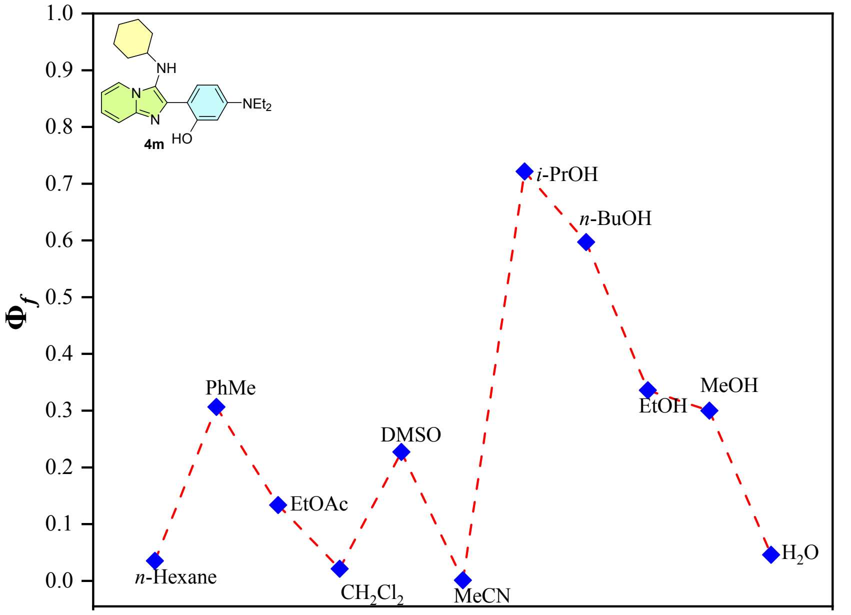

Figure S108. Variation of quantum yields of fluorescence (Φ_f) in different solvents for compound **4m**.

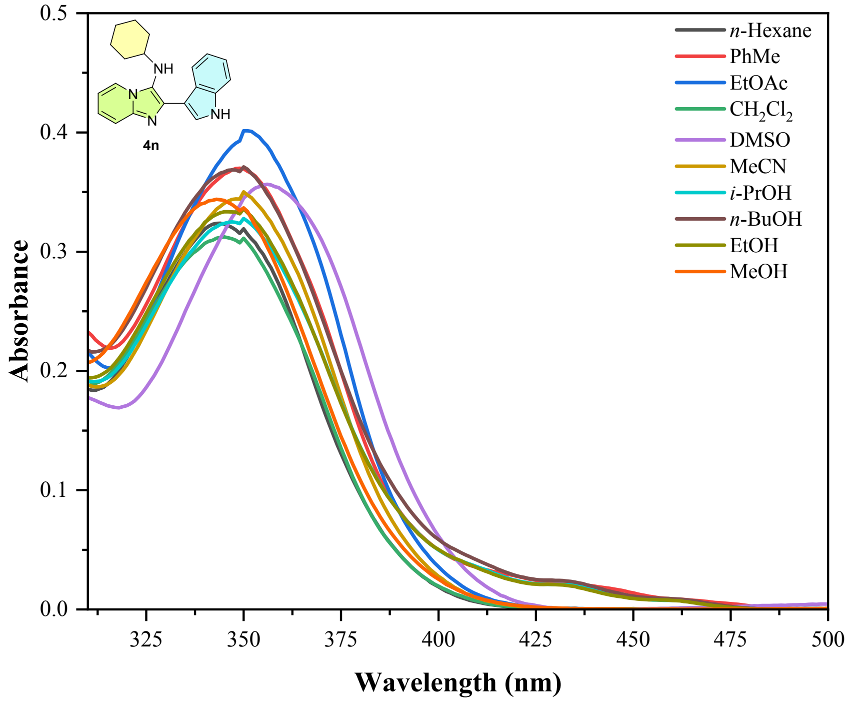

Figure S109. UV-Vis absorption spectra of **4n** in different solvents ($5{\cdot}10^{-5}$ M) at room temperature.

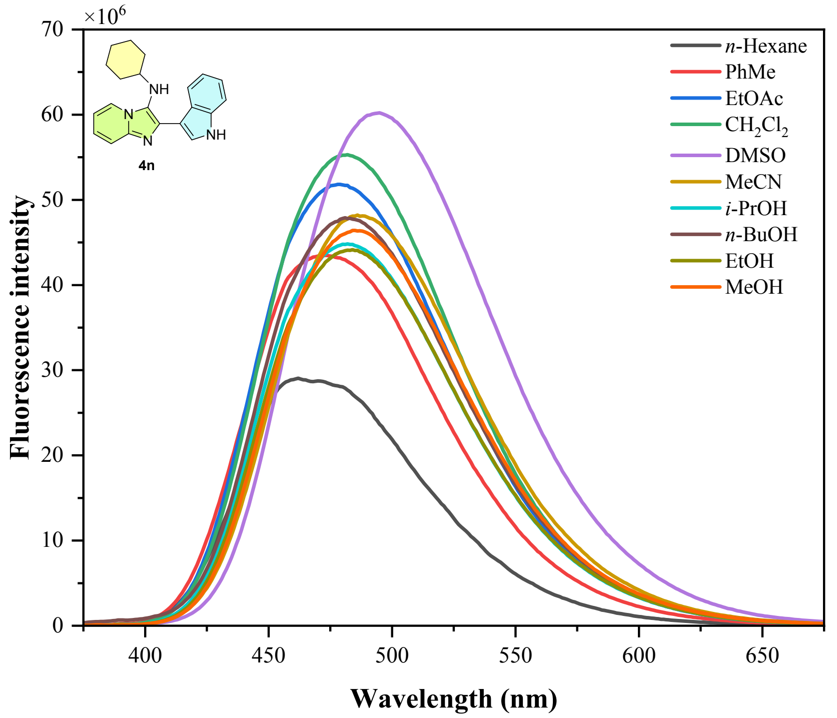

Figure S110. Normalized emission spectra of **4n** in different solvents ($5{\cdot}10^{-5}$ M) at room temperature.

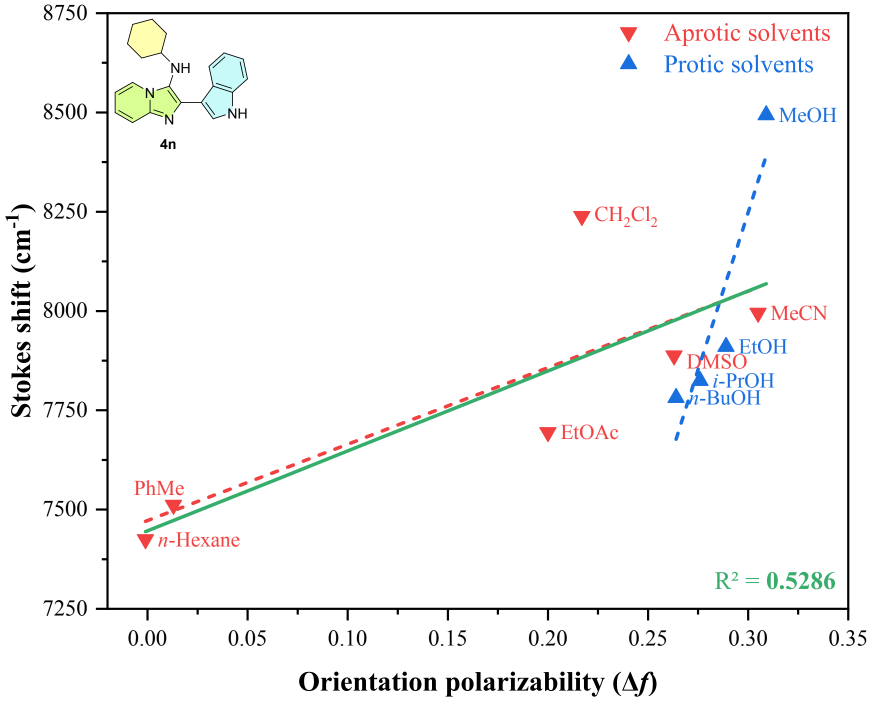

Figure S111. Lippert–Mataga plot showing Stokes shift as a function of solvent orientation polarizability (Δf) for compound **4n**.

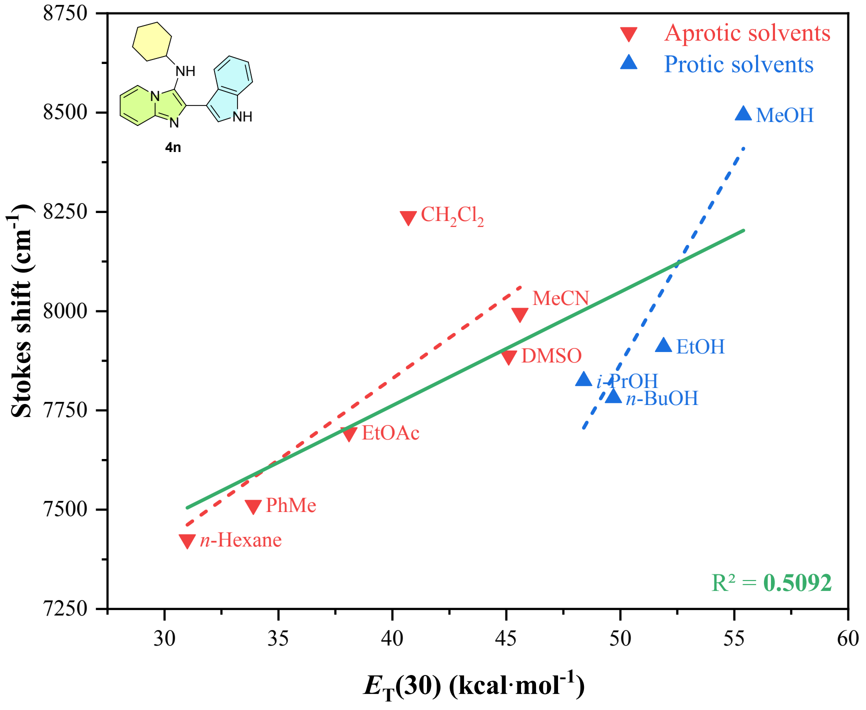

Figure S112. Dimroth-Reichardt plot showing Stokes shift against $E_T(30)$ parameter for compound **4n**.

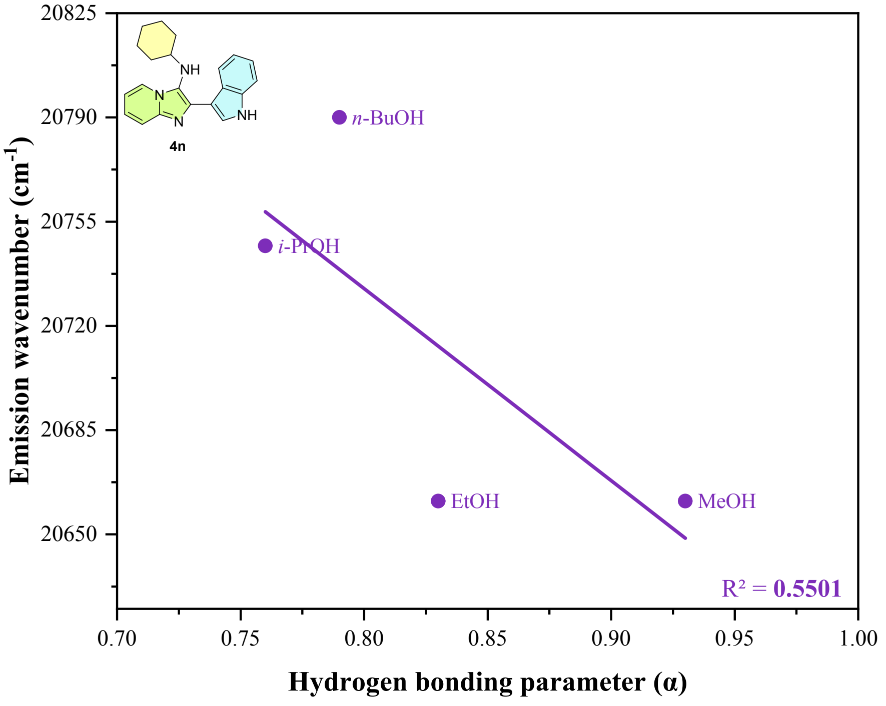

Figure S113. Plot showing hydrogen bonding parameter (α) as a function of emission wavenumber (cm^{-1}) for compound **4n**.

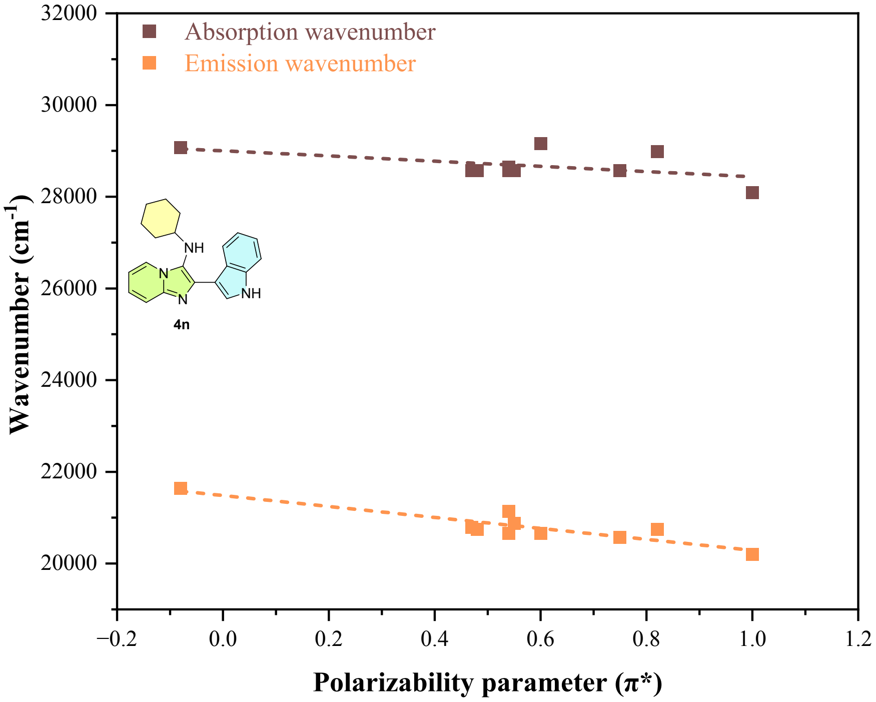

Figure S114. Plot showing polarizability parameter (π*) as a function of absorption and emission wavenumber (cm^{-1}) for compound **4n**.

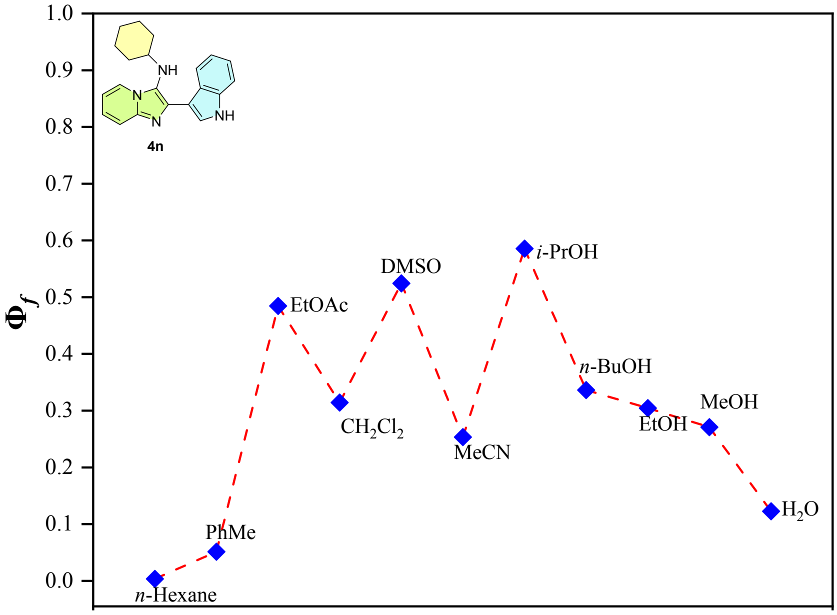

Figure S115. Variation of quantum yields of fluorescence (Φ_f) in different solvents for compound **4n**.

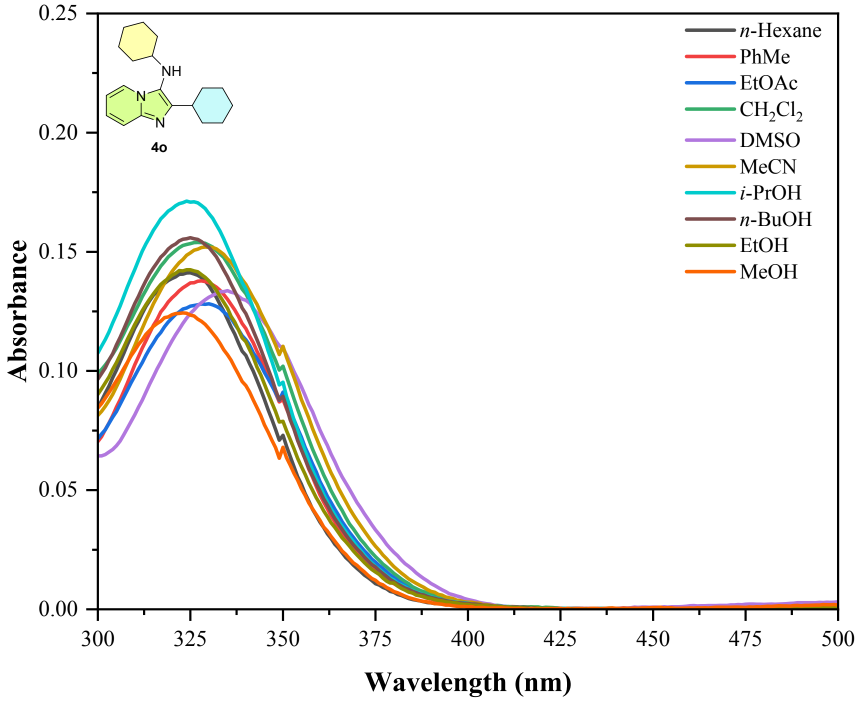

Figure S116. UV-Vis absorption spectra of **4o** in different solvents ($5 \cdot 10^{-5}$ M) at room temperature.

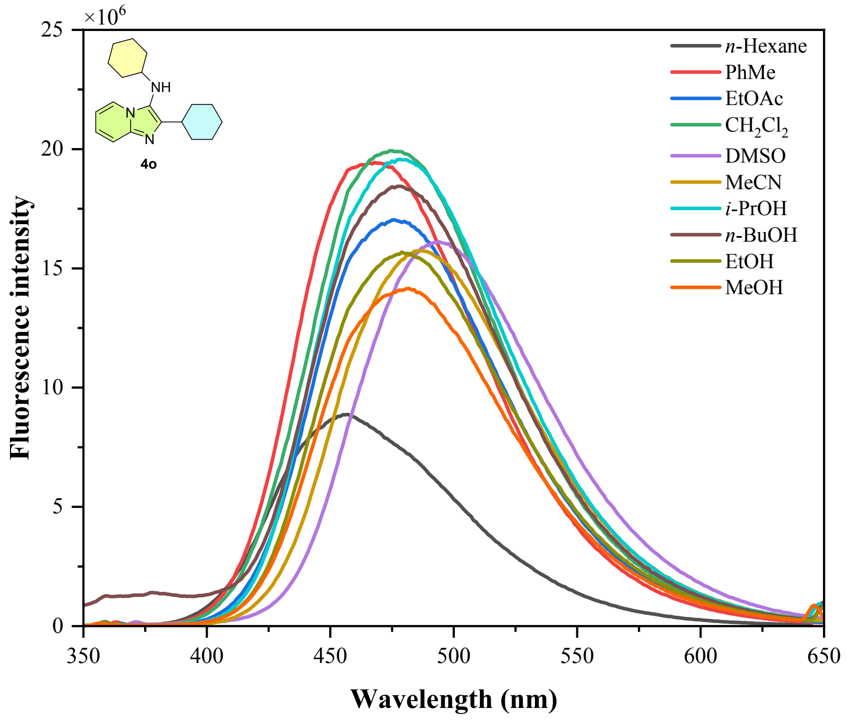

Figure S117. Normalized emission spectra of **4o** in different solvents ($5 \cdot 10^{-5}$ M) at room temperature.

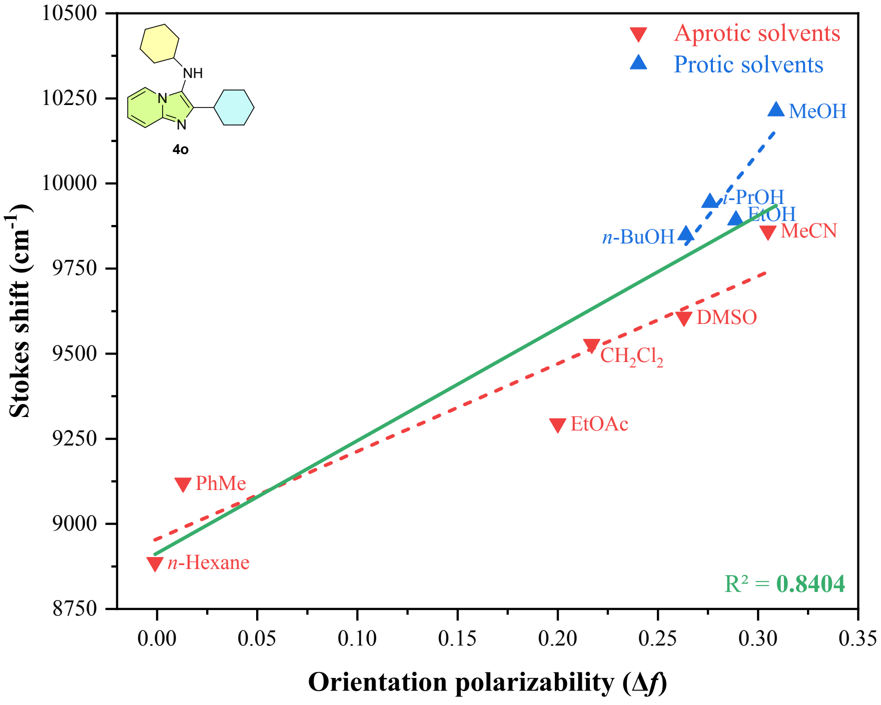

Figure S118. Lippert–Mataga plot showing Stokes shift as a function of solvent orientation polarizability (Δ*f*) for compound **4o**.

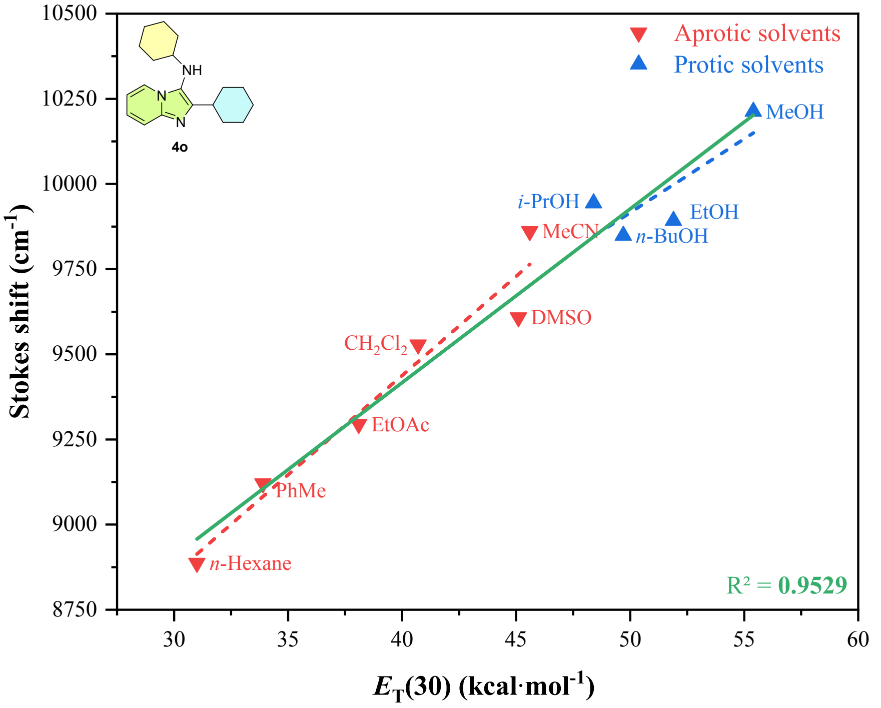

Figure S119. Dimroth-Reichardt plot showing Stokes shift against $E_T(30)$ parameter for compound **4o**.

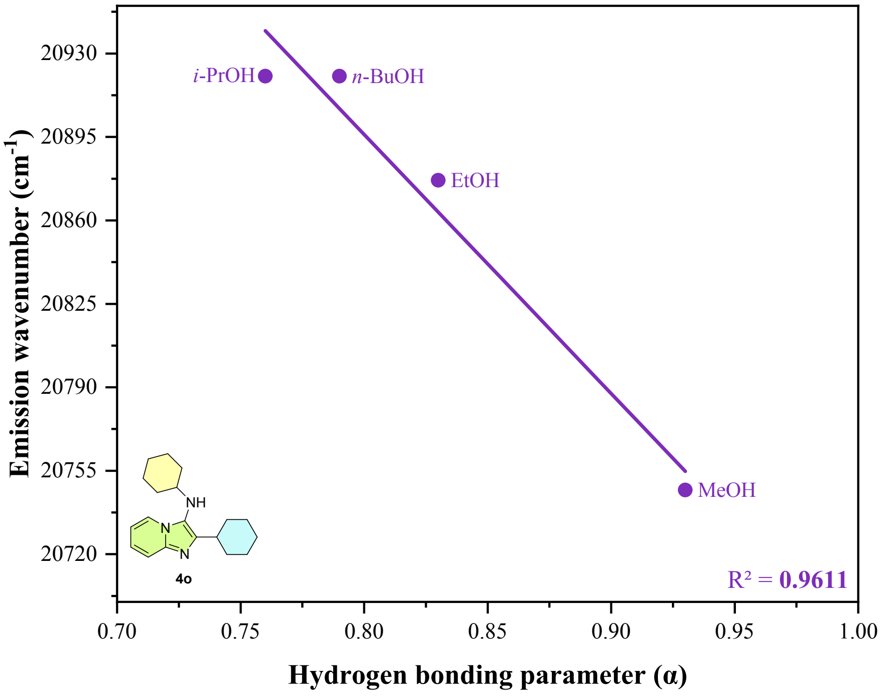

Figure S120. Plot showing hydrogen bonding parameter (α) as a function of emission wavenumber (cm^{-1}) for compound **4o**.

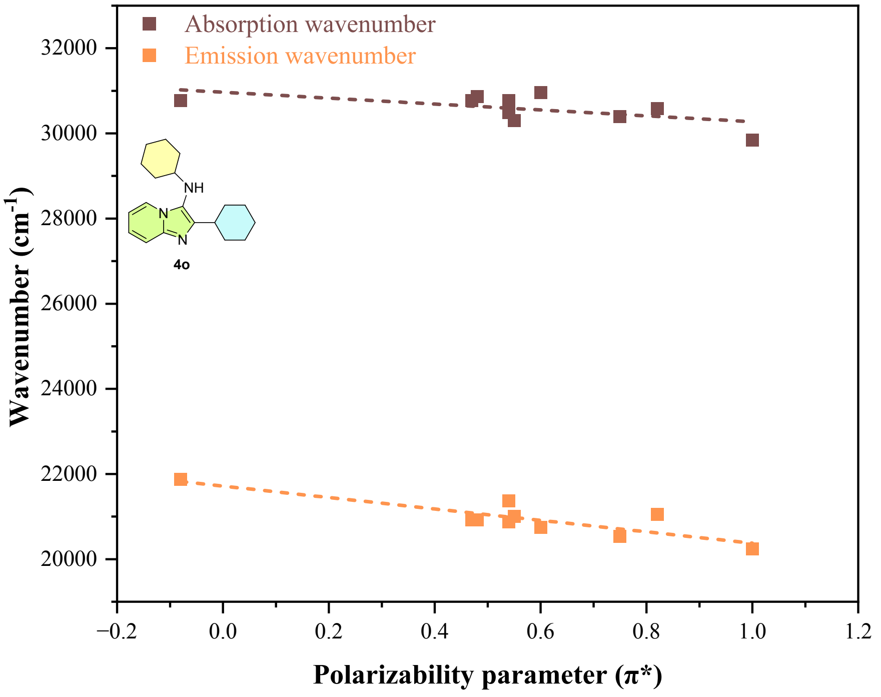

Figure S121. Plot showing polarizability parameter (π*) as a function of absorption and emission wavenumber (cm^{-1}) for compound **4o**.

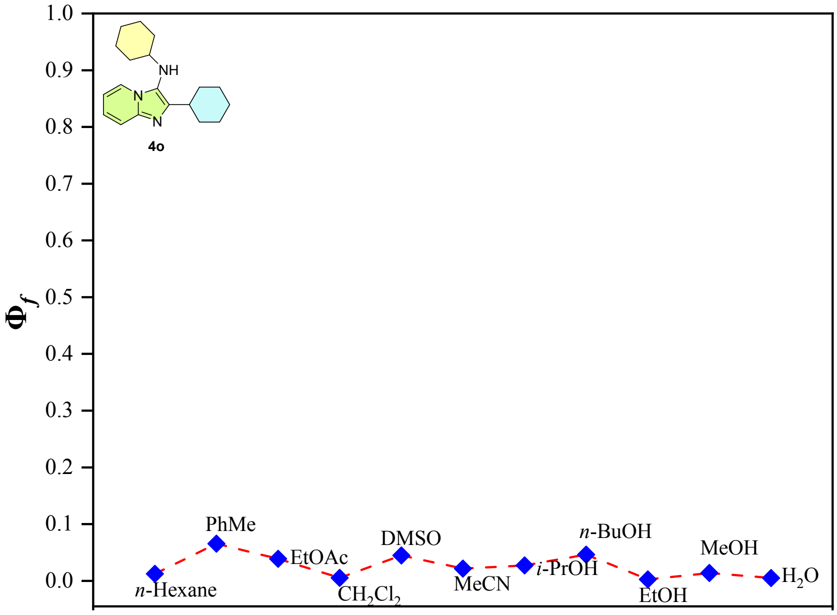

Figure S122. Variation of quantum yields of fluorescence (Φ_f) in different solvents for compound **4o**.

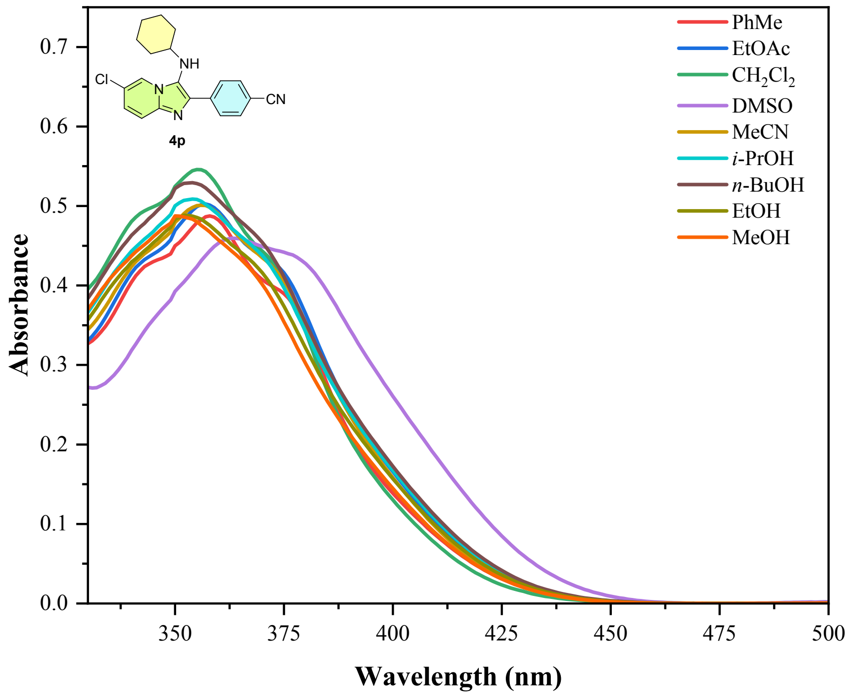

Figure S123. UV-Vis absorption spectra of **4p** in different solvents ($5 \cdot 10^{-5}$ M) at room temperature.

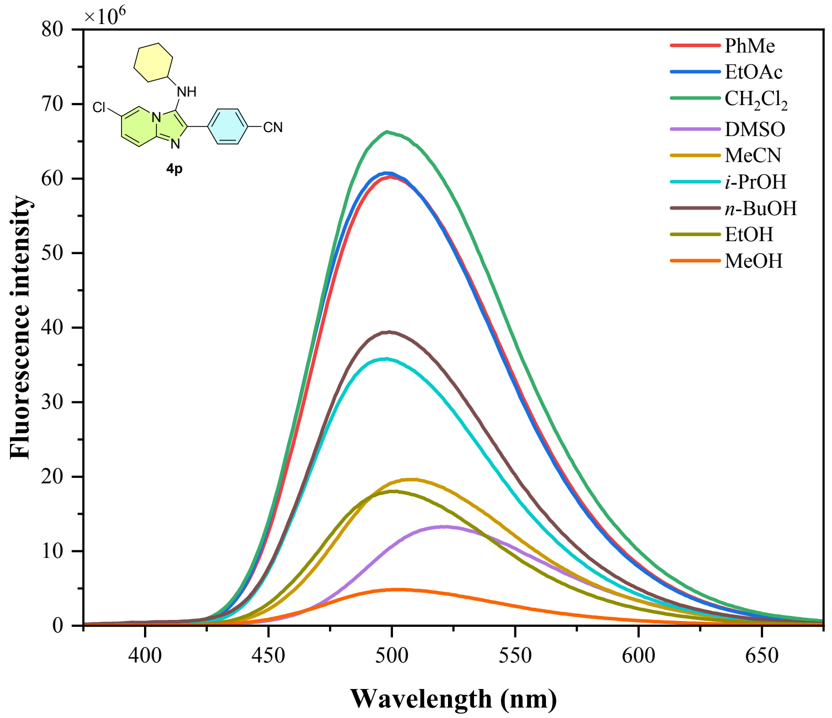

Figure S124. Normalized emission spectra of **4p** in different solvents (5·10^{-5} M) at room temperature.

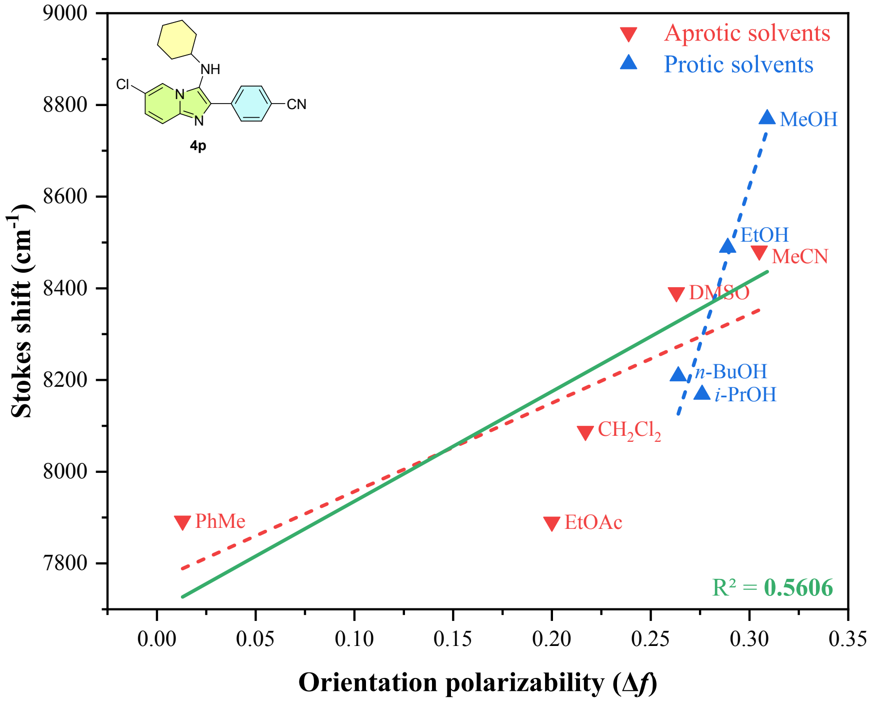

Figure S125. Lippert–Mataga plot showing Stokes shift as a function of solvent orientation polarizability (Δ*f*) for compound **4p**.

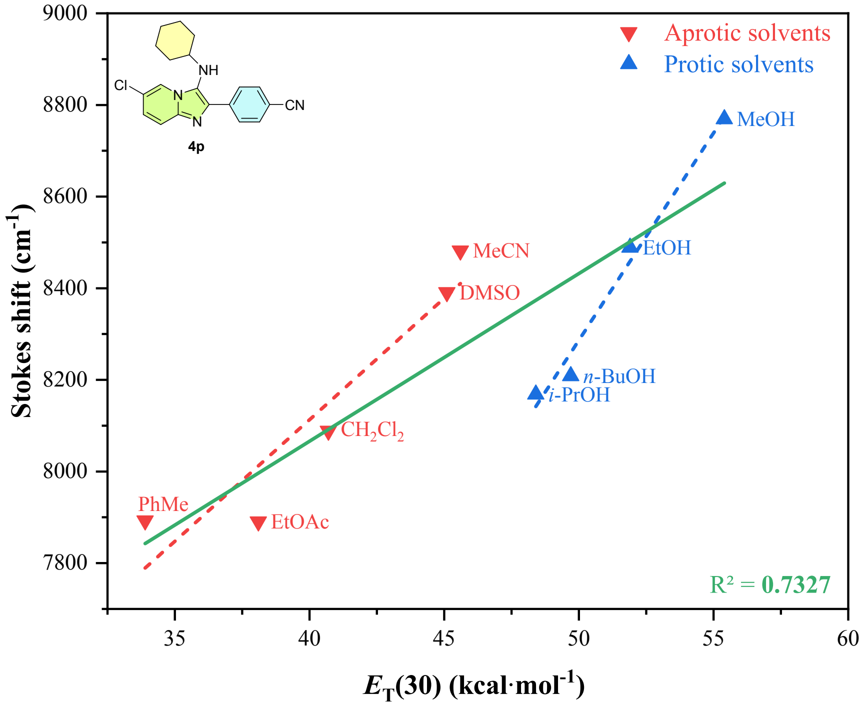

Figure S126. Dimroth-Reichardt plot showing Stokes shift against $E_T(30)$ parameter for compound **4p**.

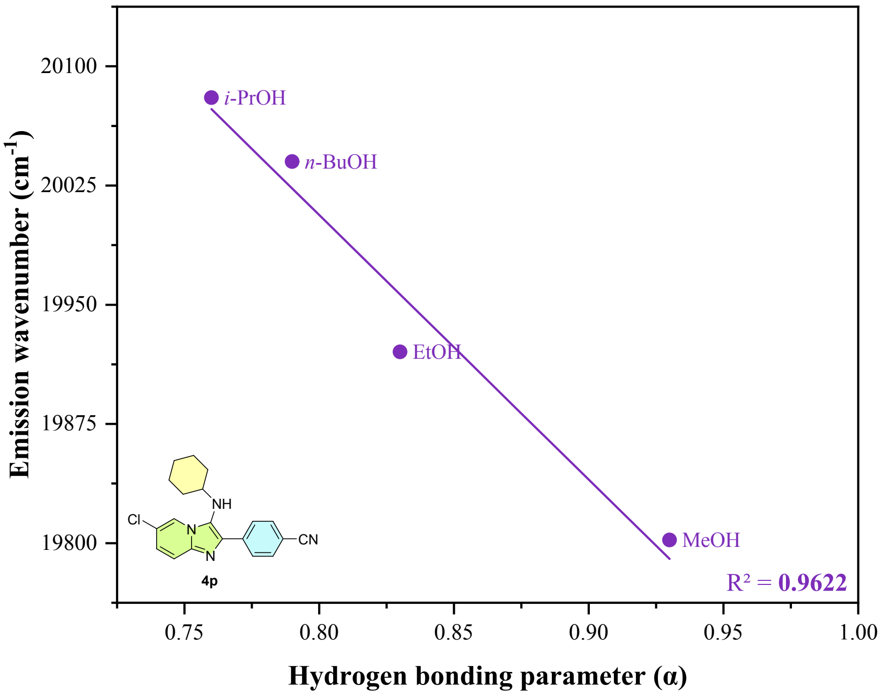

Figure S127. Plot showing hydrogen bonding parameter (α) as a function of emission wavenumber (cm^{-1}) for compound **4p**.

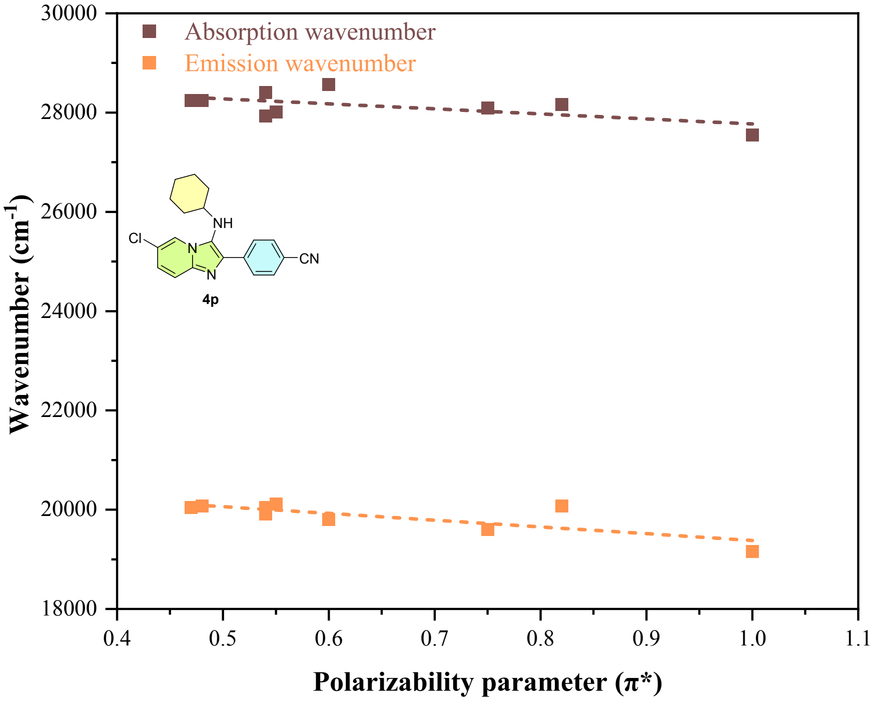

Figure S128. Plot showing polarizability parameter (π*) as a function of absorption and emission wavenumber (cm^{-1}) for compound **4p**.

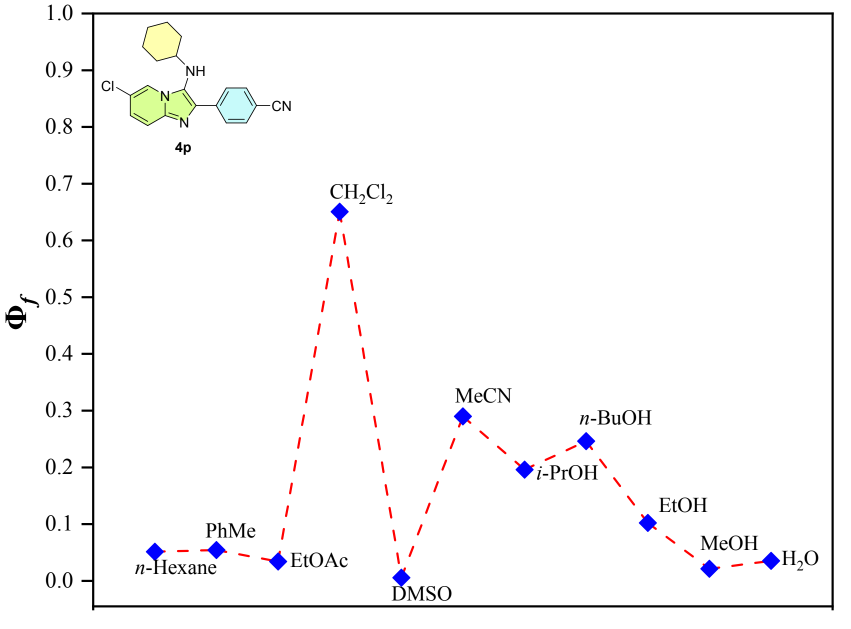

Figure S129. Variation of quantum yields of fluorescence (Φ_f) in different solvents for compound **4p**.

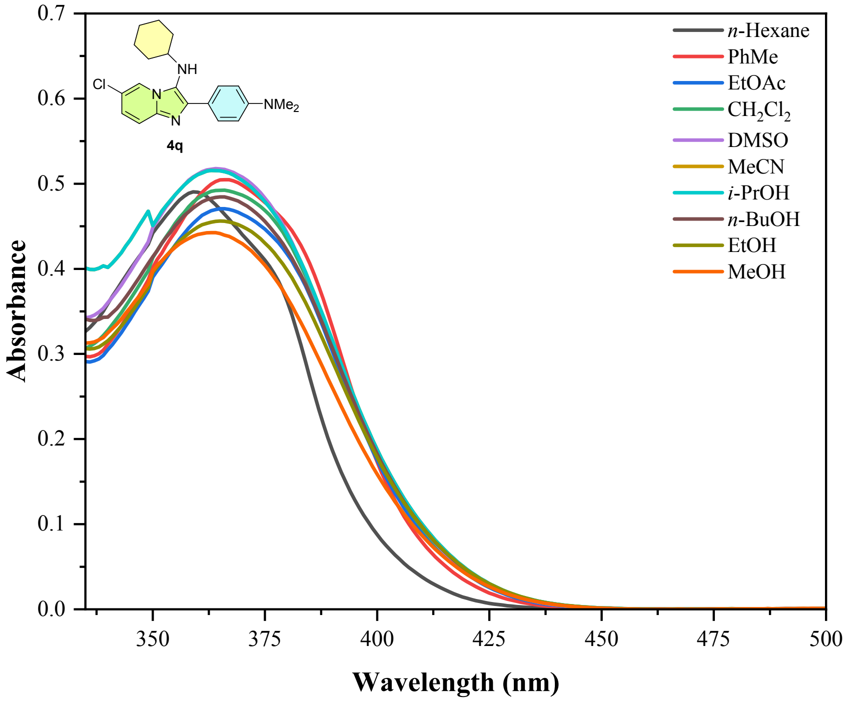

Figure S130. UV-Vis absorption spectra of **4q** in different solvents ($5{\cdot}10^{-5}$ M) at room temperature.

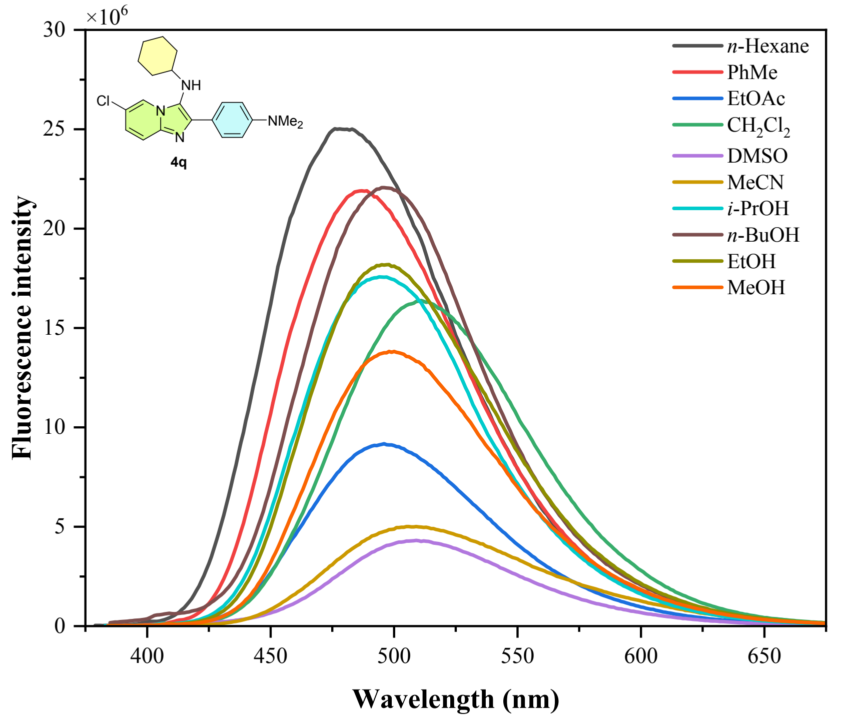

Figure S131. Normalized emission spectra of **4q** in different solvents ($5{\cdot}10^{-5}$ M) at room temperature.

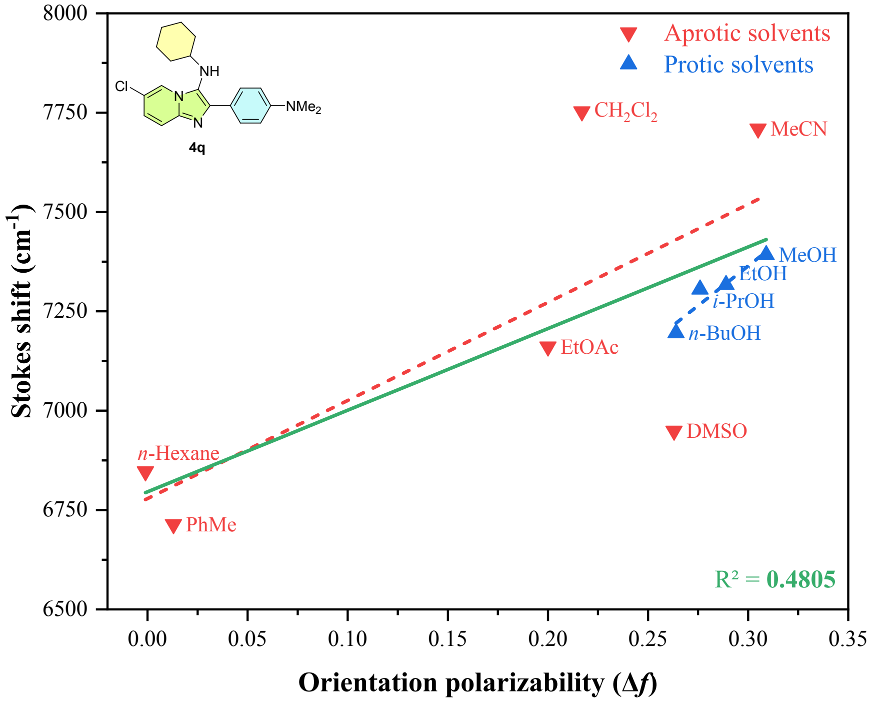

Figure S132. Lippert–Mataga plot showing Stokes shift as a function of solvent orientation polarizability (Δ*f*) for compound **4q**.

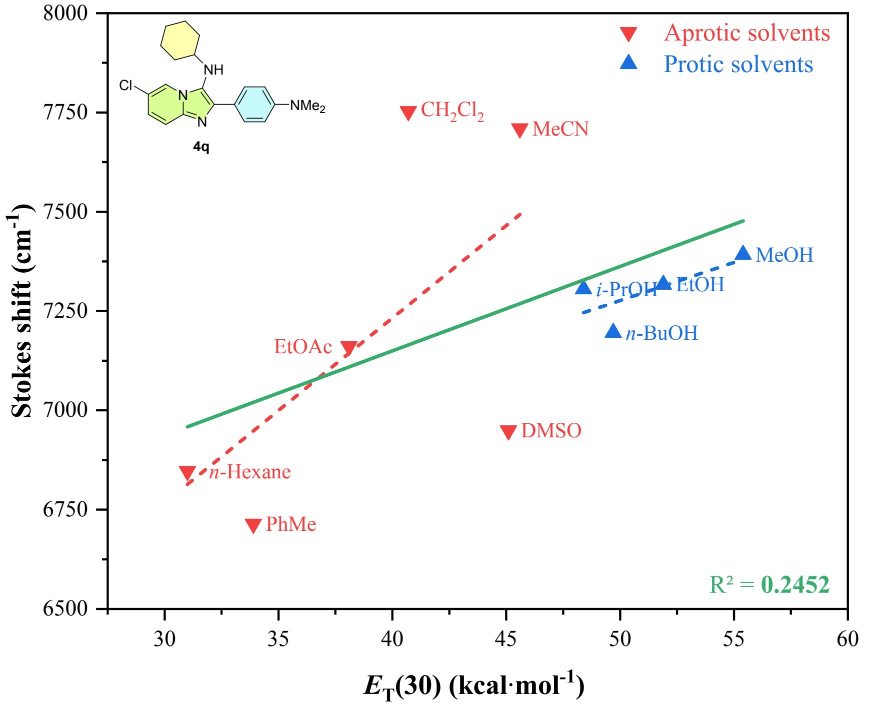

Figure S133. Dimroth-Reichardt plot showing Stokes shift against $E_T(30)$ parameter for compound **4q**.

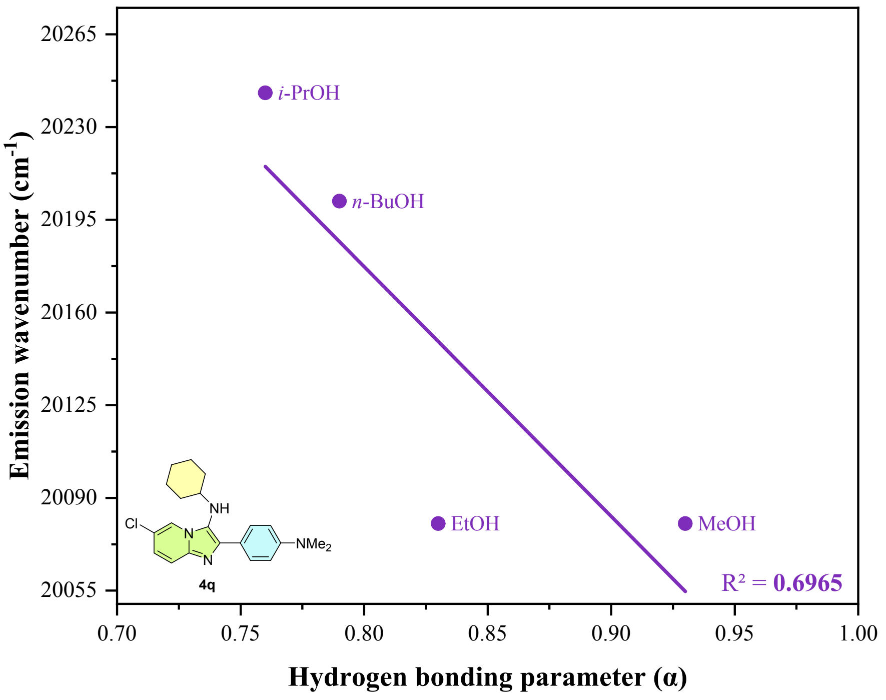

Figure S134. Plot showing hydrogen bonding parameter (α) as a function of emission wavenumber (cm^{-1}) for compound **4q**.

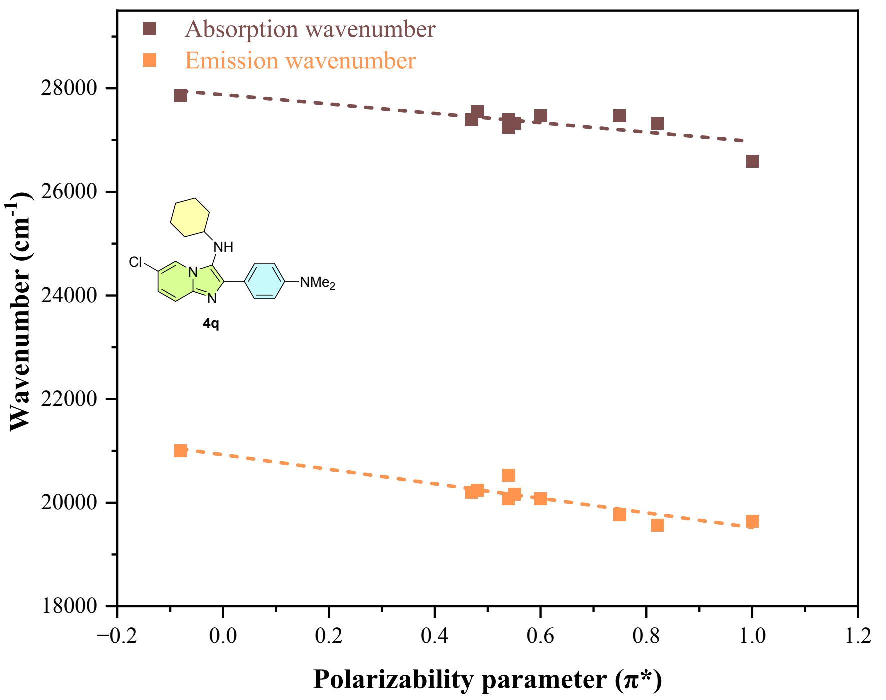

Figure S135. Plot showing polarizability parameter (π*) as a function of absorption and emission wavenumber (cm^{-1}) for compound **4q**.

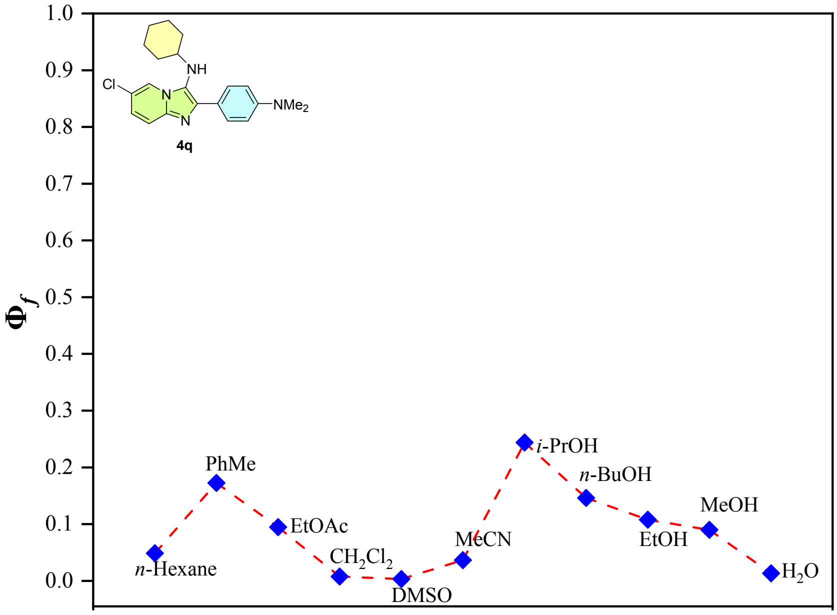

Figure S136. Variation of quantum yields of fluorescence (Φ_f) in different solvents for compound **4q**.

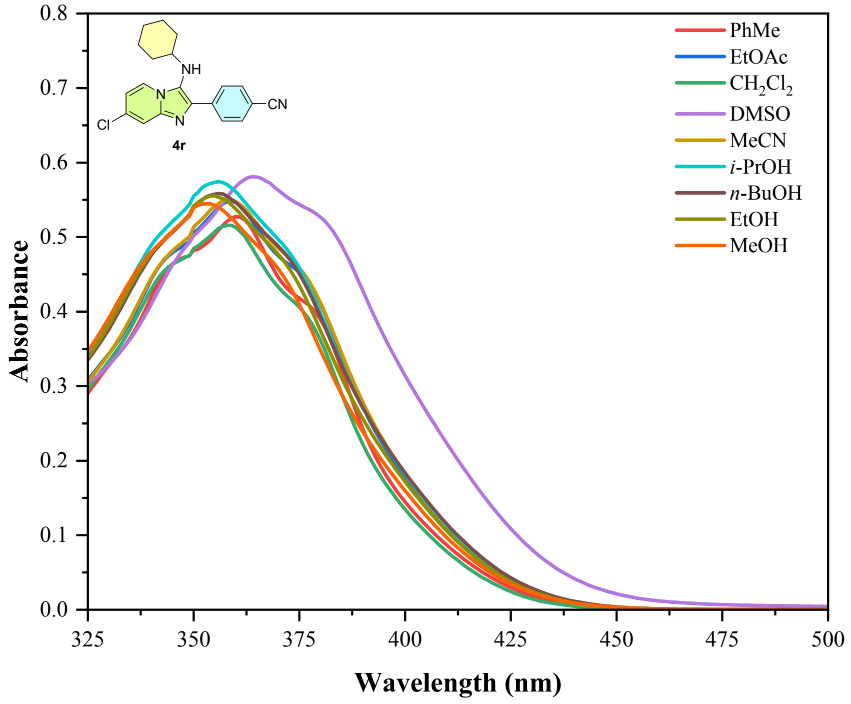

Figure S137. UV-Vis absorption spectra of **4r** in different solvents ($5{\cdot}10^{-5}$ M) at room temperature.

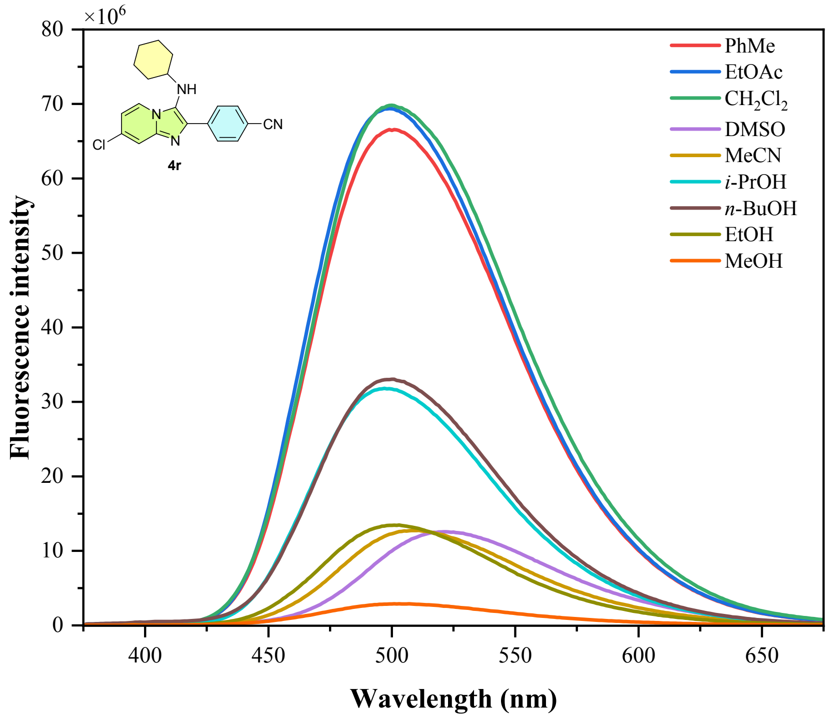

Figure S138. Normalized emission spectra of **4r** in different solvents (5·10^{-5} M) at room temperature.

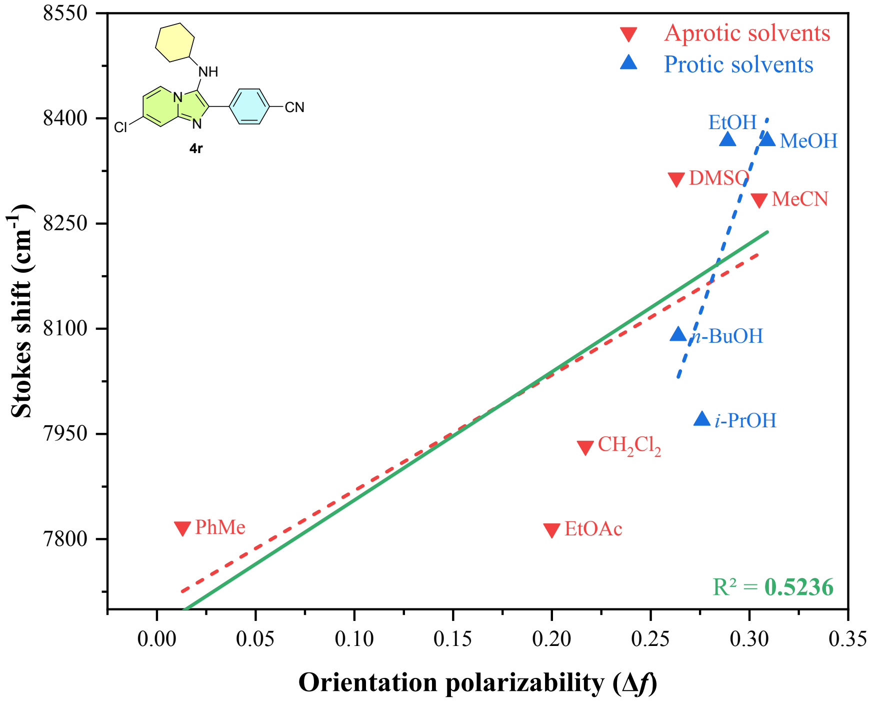

Figure S139. Lippert–Mataga plot showing Stokes shift as a function of solvent orientation polarizability (Δf) for compound **4r**.

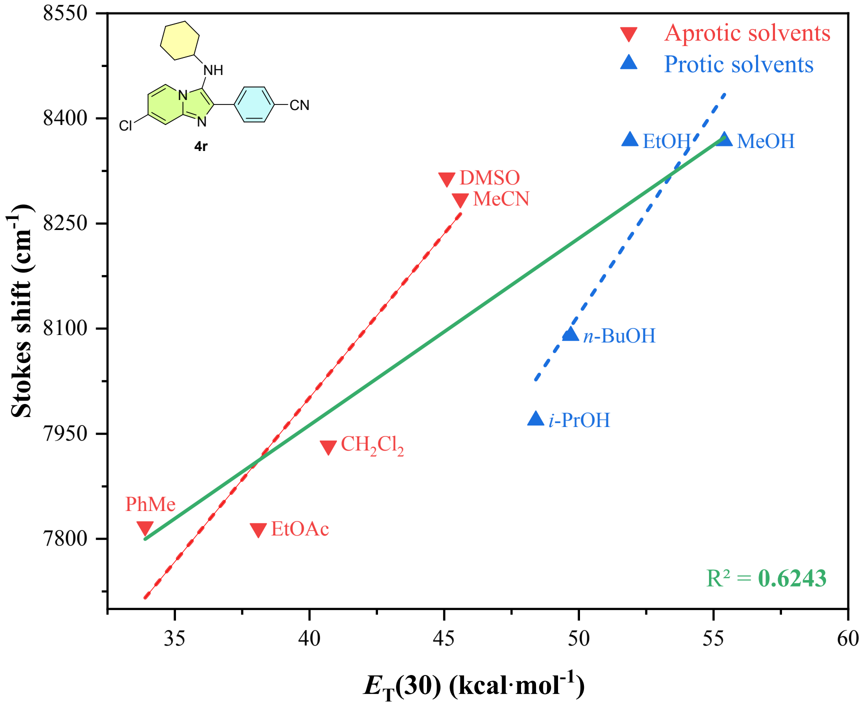

Figure S140. Dimroth-Reichardt plot showing Stokes shift against $E_T(30)$ parameter for compound **4r**.

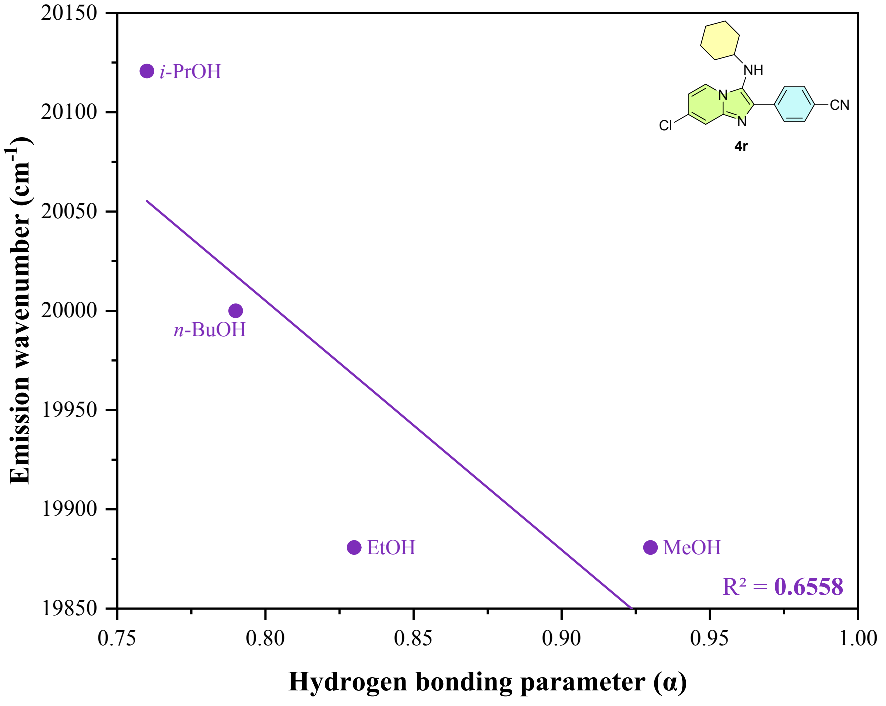

Figure S141. Plot showing hydrogen bonding parameter (α) as a function of emission wavenumber (cm^{-1}) for compound **4r**.

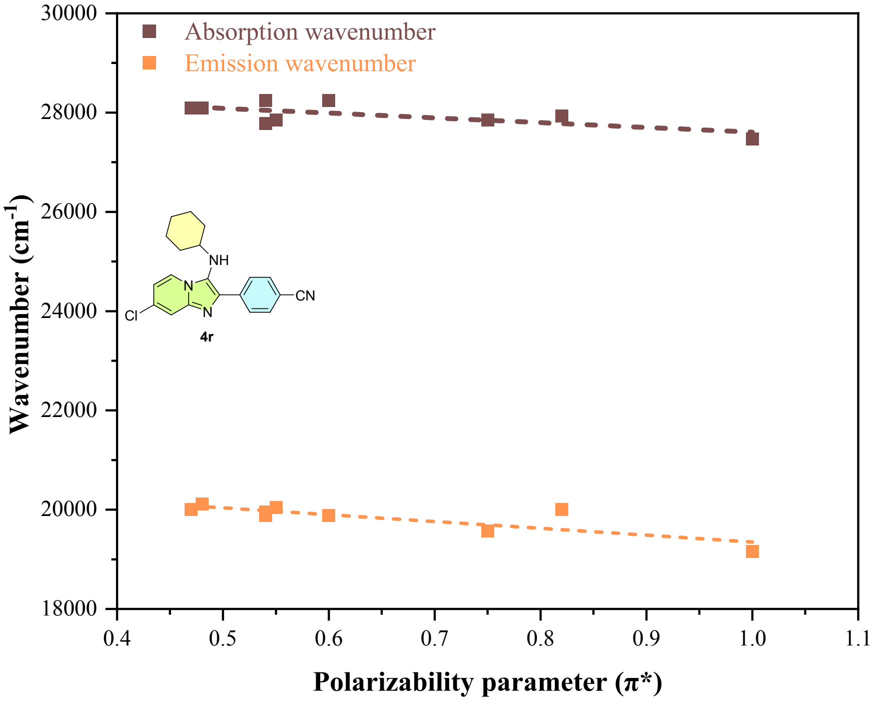

Figure S142. Plot showing polarizability parameter (π*) as a function of absorption and emission wavenumber (cm^{-1}) for compound **4r**.

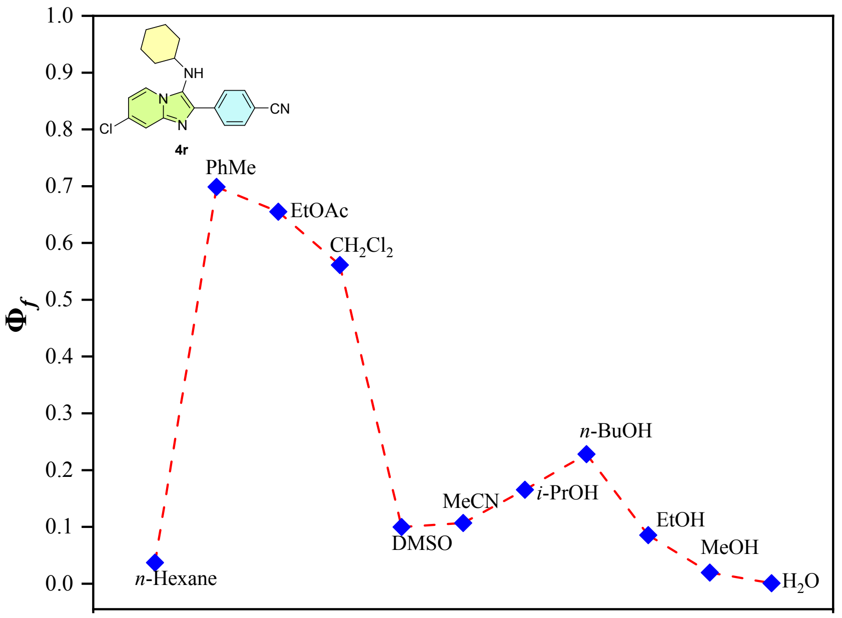

Figure S143. Variation of quantum yields of fluorescence (Φ_f) in different solvents for compound **4r**.

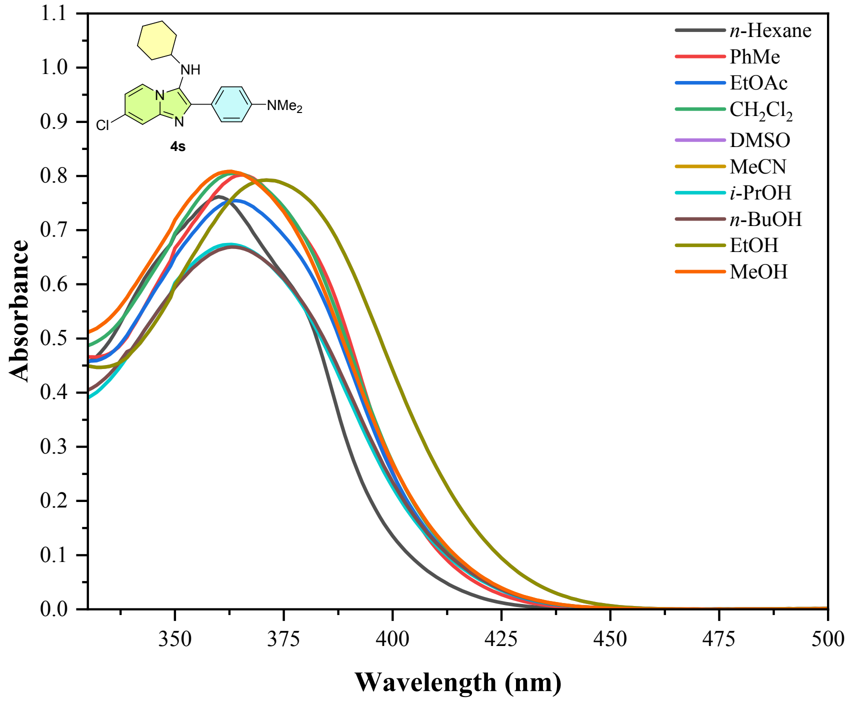

Figure S144. UV-Vis absorption spectra of **4s** in different solvents ($5{\cdot}10^{-5}$ M) at room temperature.

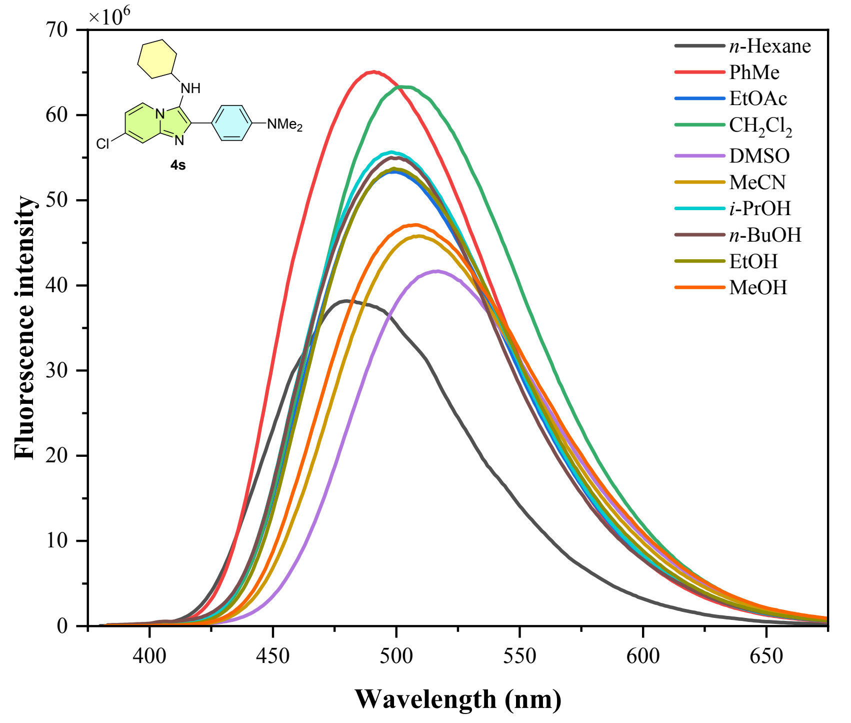

Figure S145. Normalized emission spectra of **4s** in different solvents ($5{\cdot}10^{-5}$ M) at room temperature.

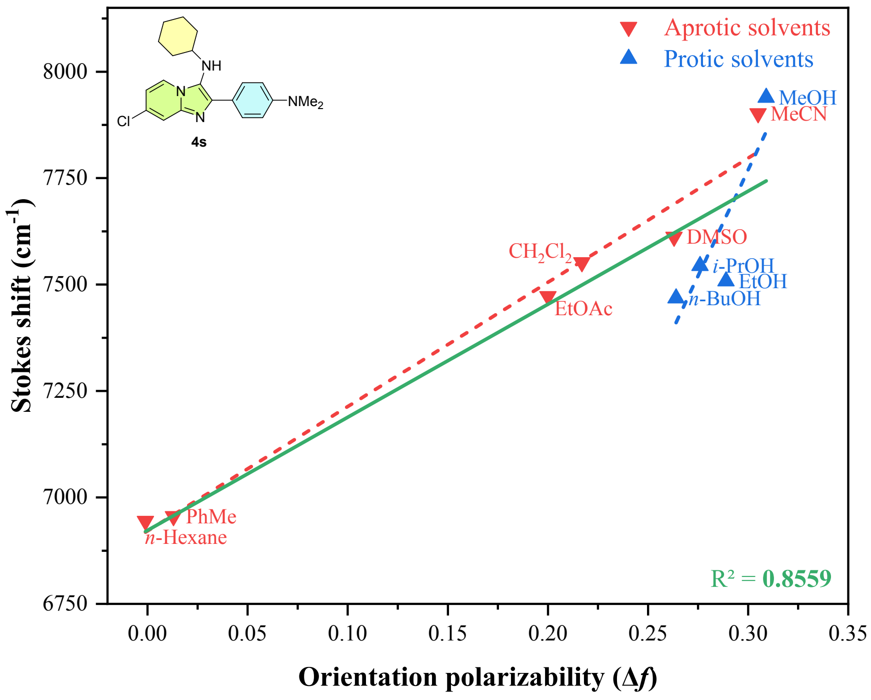

Figure S146. Lippert–Mataga plot showing Stokes shift as a function of solvent orientation polarizability (Δf) for compound **4s**.

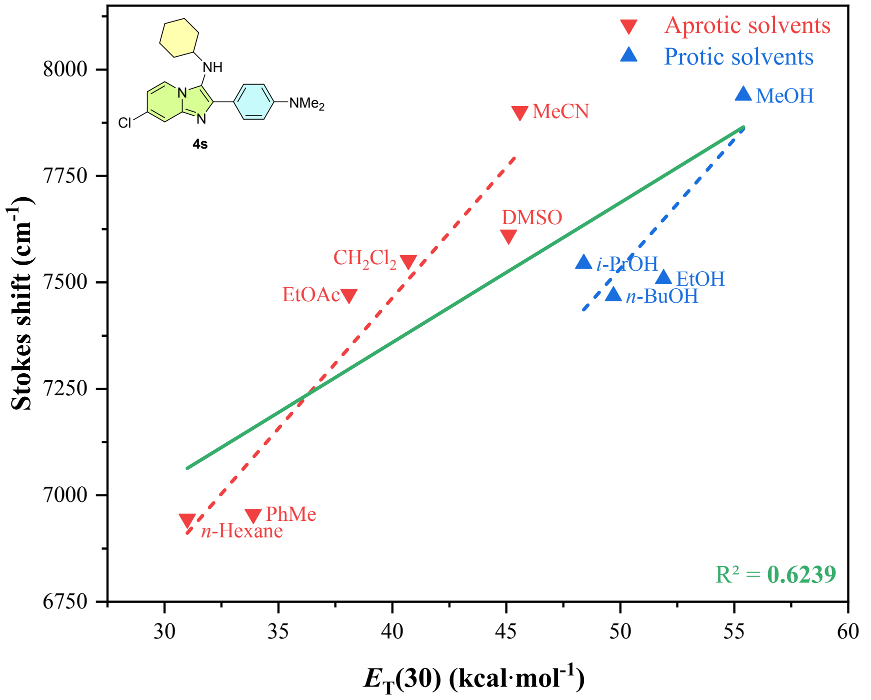

Figure S147. Dimroth-Reichardt plot showing Stokes shift against $E_T(30)$ parameter for compound **4s**.

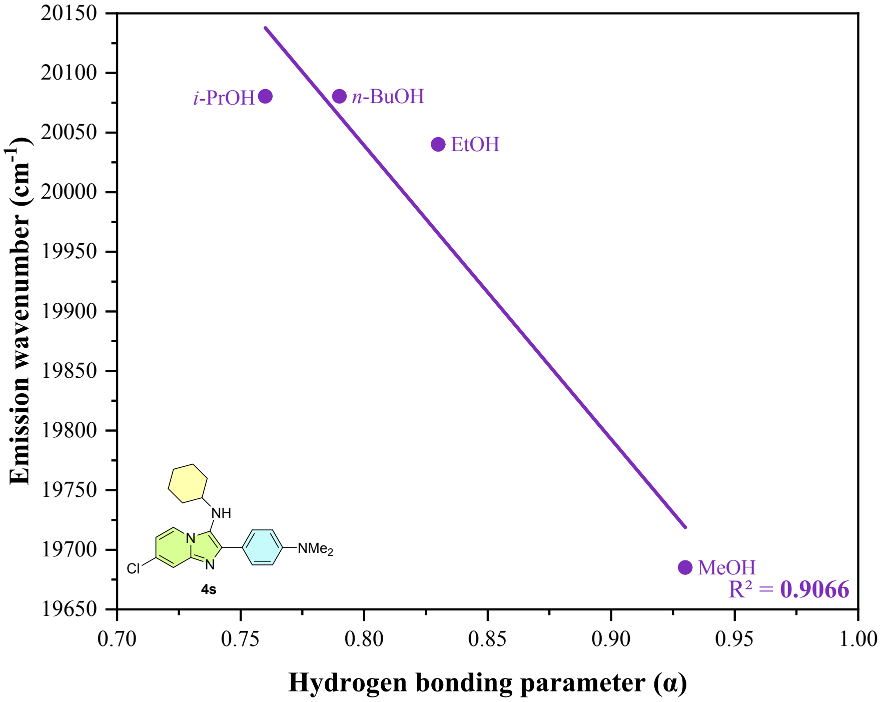

Figure S148. Plot showing hydrogen bonding parameter (α) as a function of emission wavenumber (cm^{-1}) for compound **4s**.

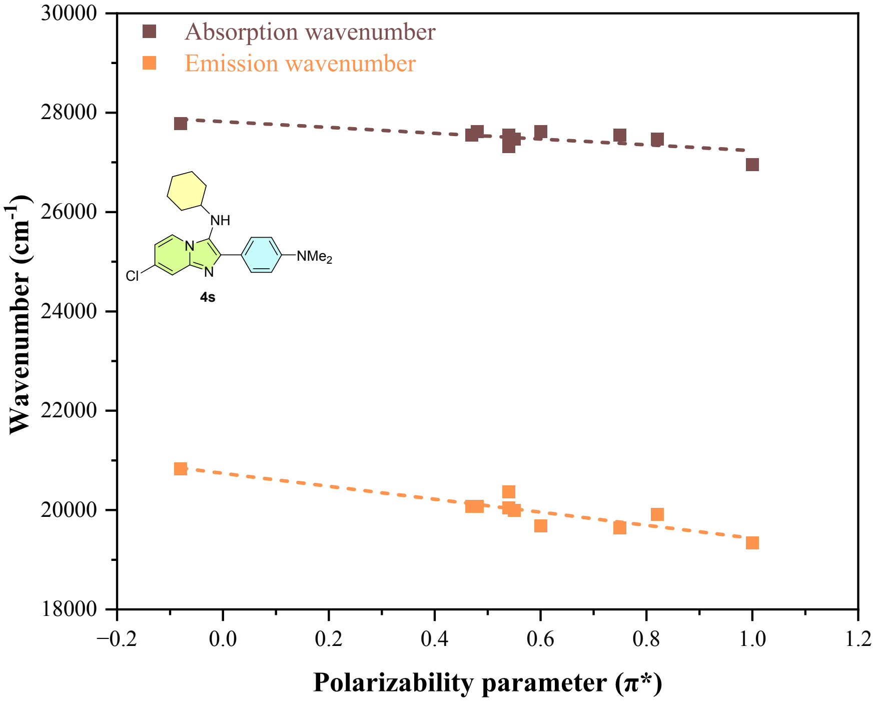

Figure S149. Plot showing polarizability parameter (π*) as a function of absorption and emission wavenumber (cm^{-1}) for compound **4s**.

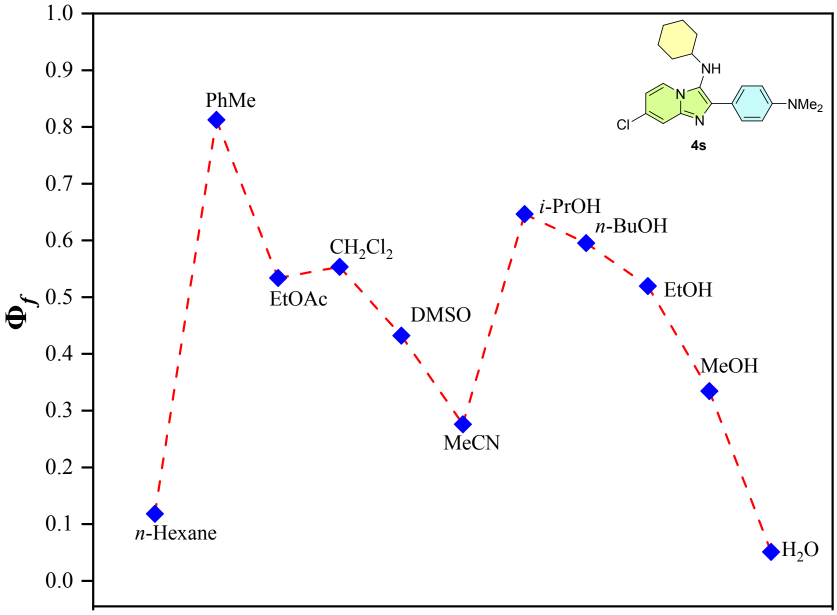

Figure S150. Variation of quantum yields of fluorescence (Φ_f) in different solvents for compound **4s**.

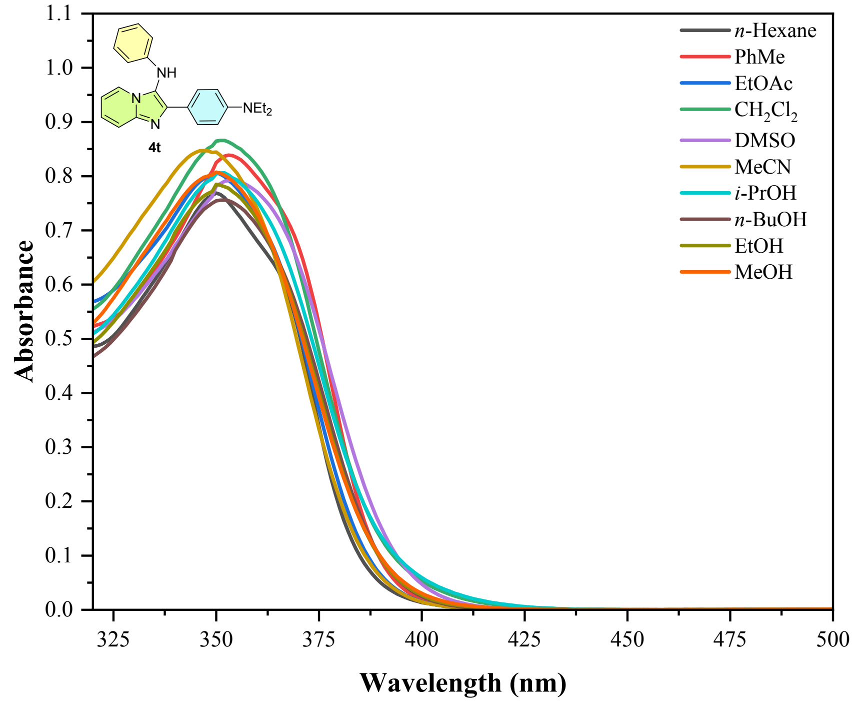

Figure S151. UV-Vis absorption spectra of **4t** in different solvents ($5 \cdot 10^{-5}$ M) at room temperature.

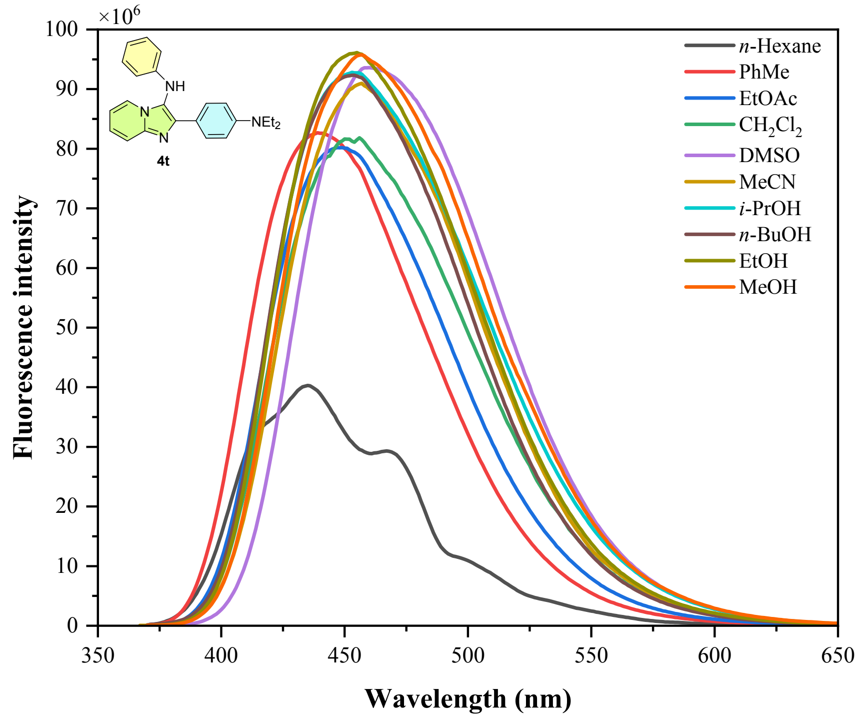

Figure S152. Normalized emission spectra of **4t** in different solvents ($5{\cdot}10^{-5}$ M) at room temperature.

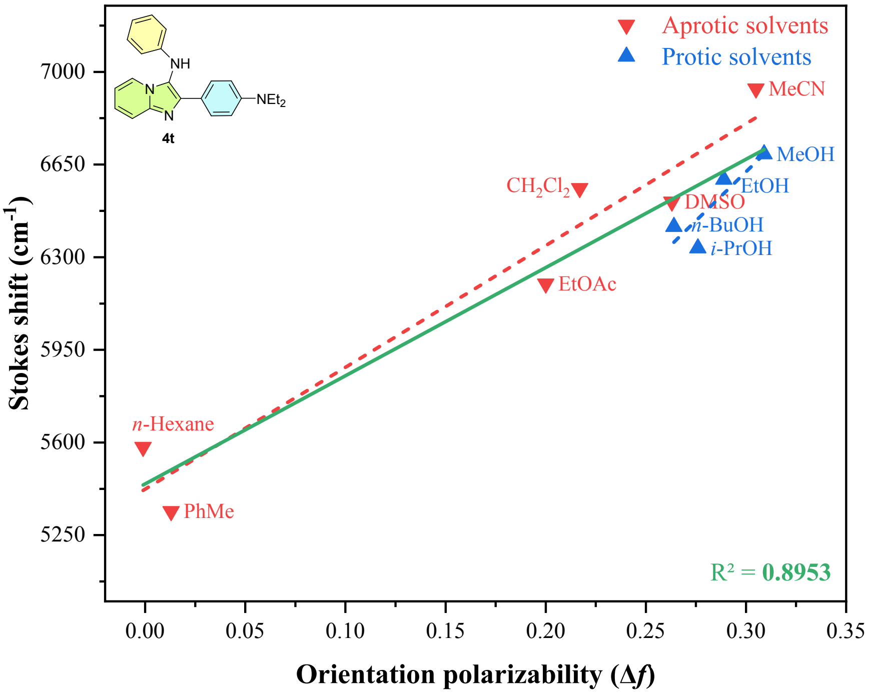

Figure S153. Lippert–Mataga plot showing Stokes shift as a function of solvent orientation polarizability (Δf) for compound **4t**.

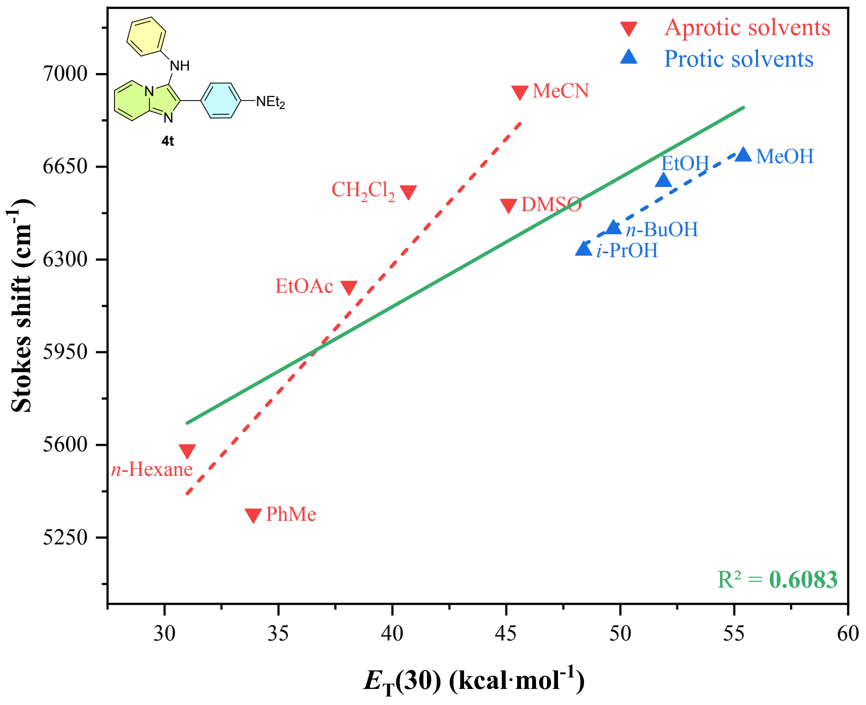

Figure S154. Dimroth-Reichardt plot showing Stokes shift against $E_T(30)$ parameter for compound **4t**.

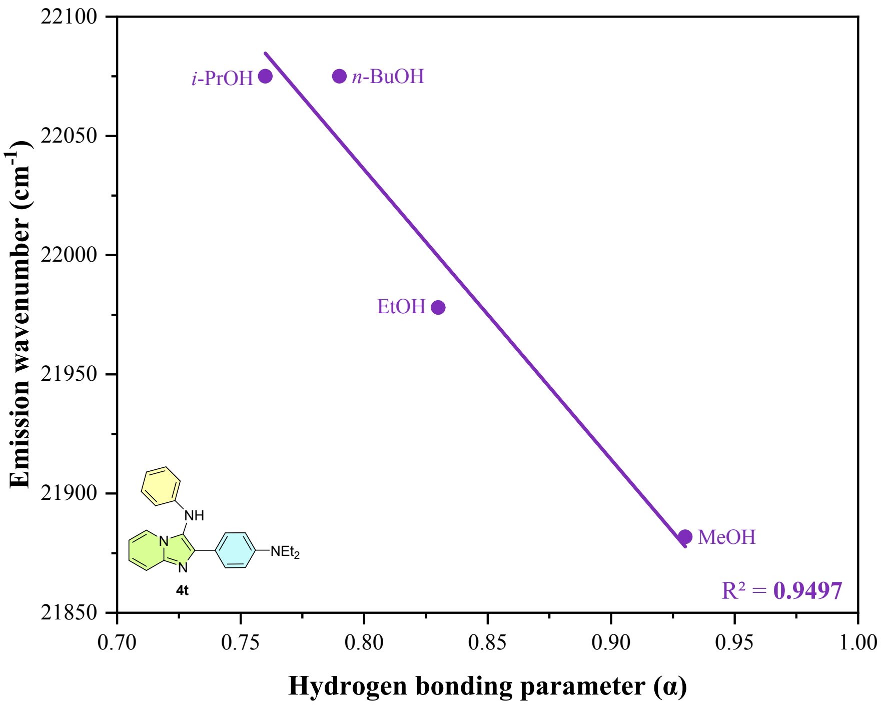

Figure S155. Plot showing hydrogen bonding parameter (α) as a function of emission wavenumber (cm^{-1}) for compound **4t**.

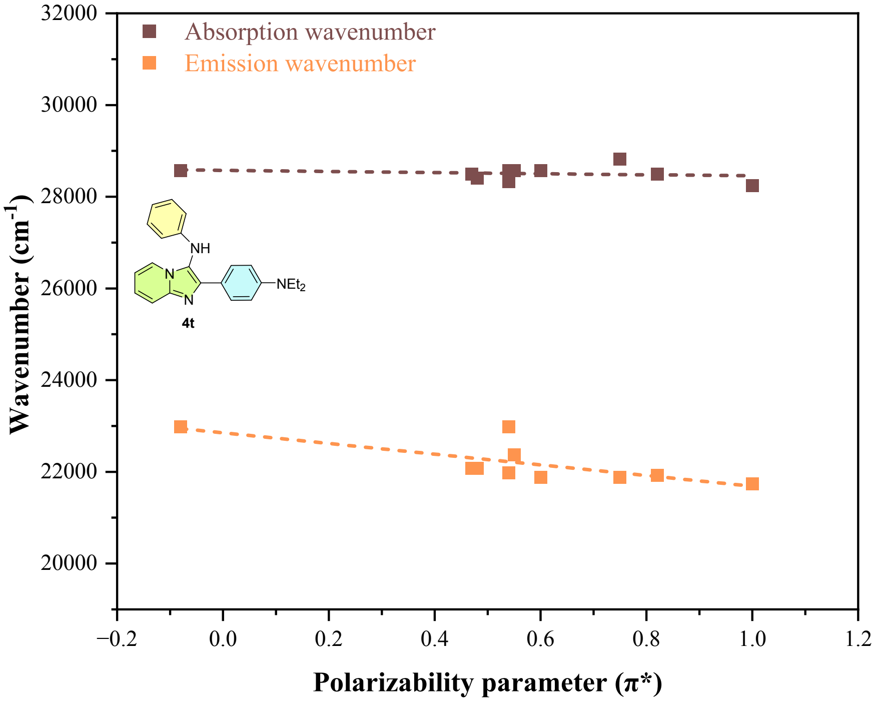

Figure S156. Plot showing polarizability parameter (π*) as a function of absorption and emission wavenumber (cm^{-1}) for compound **4t**.

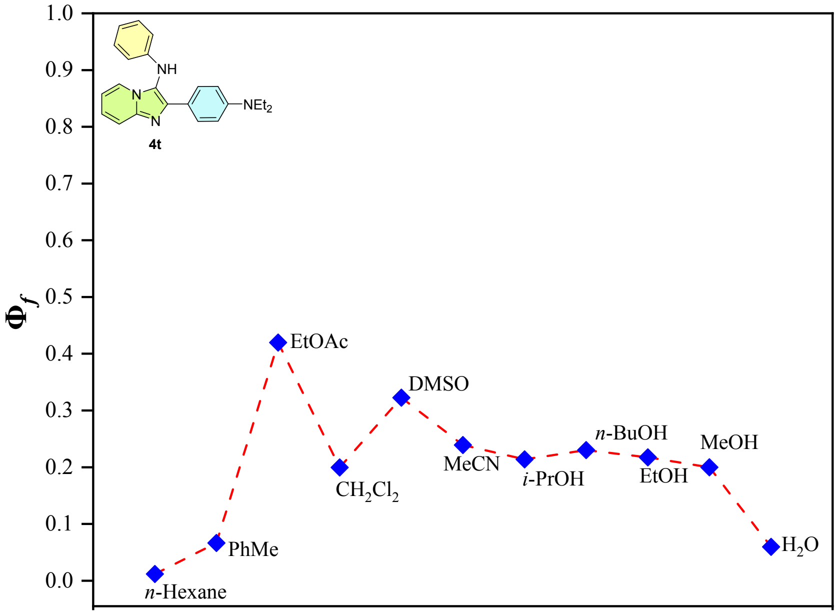

Figure S157. Variation of quantum yields of fluorescence (Φ_f) in different solvents for compound **4t**.

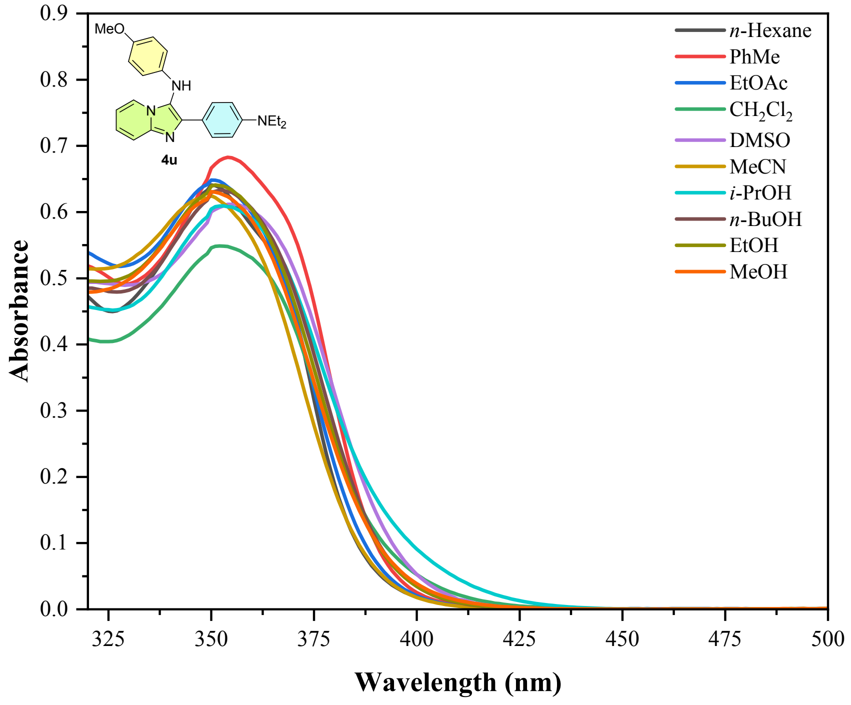

Figure S158. UV-Vis absorption spectra of **4u** in different solvents ($5{\cdot}10^{-5}$ M) at room temperature.

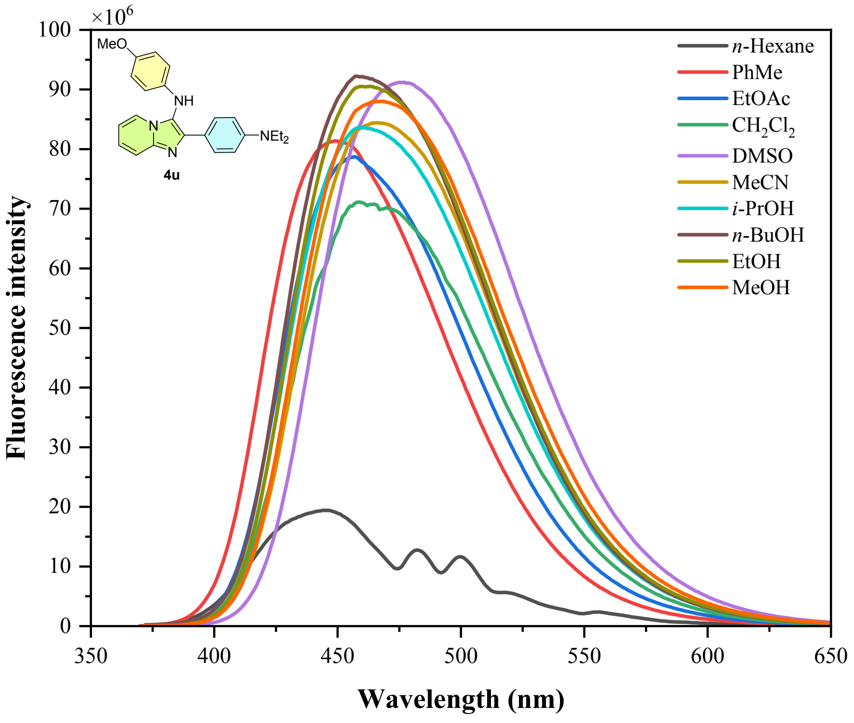

Figure S159. Normalized emission spectra of **4u** in different solvents ($5{\cdot}10^{-5}$ M) at room temperature.

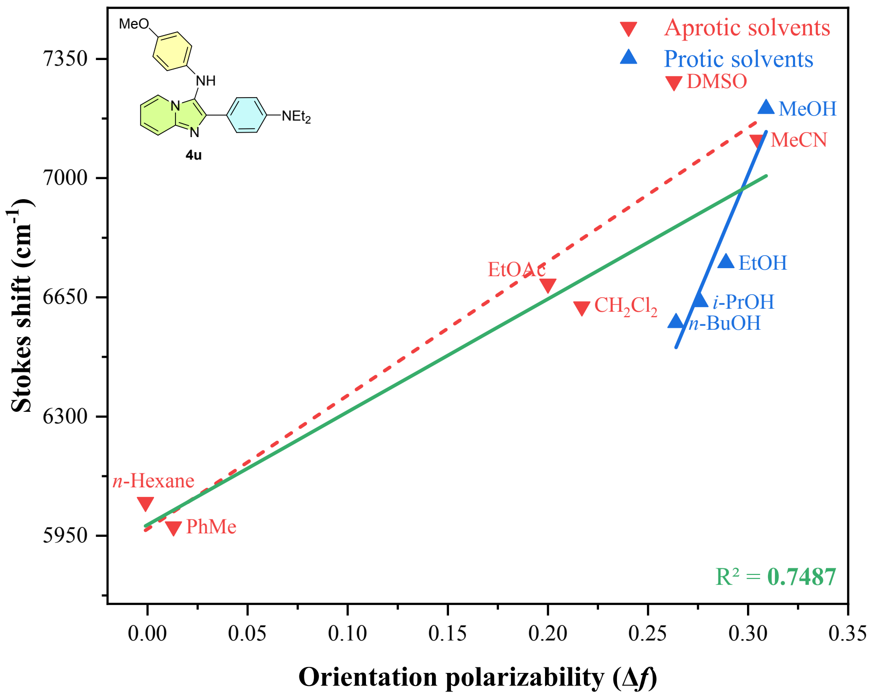

Figure S160. Lippert–Mataga plot showing Stokes shift as a function of solvent orientation polarizability (Δf) for compound **4u**.

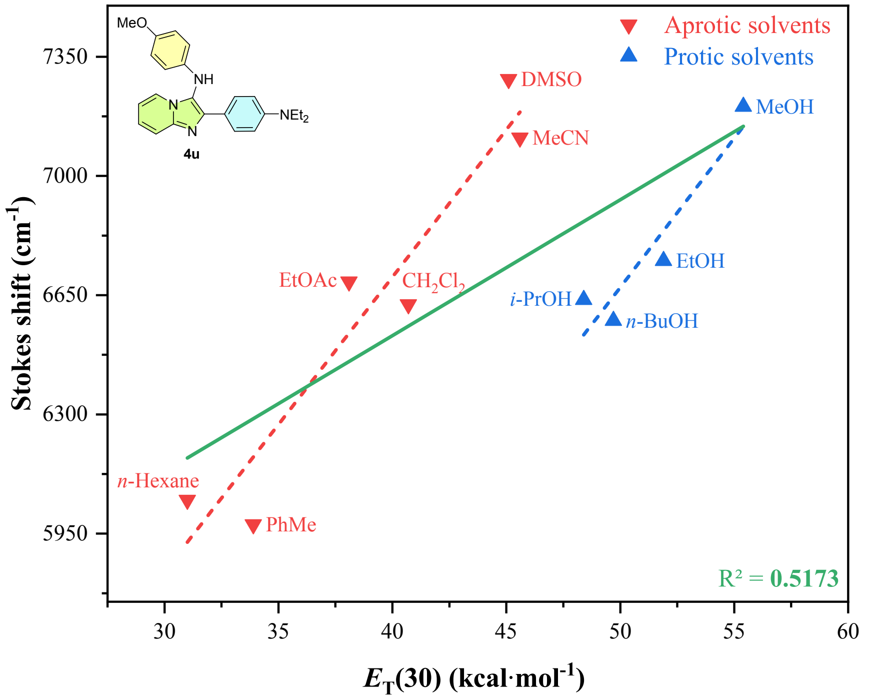

Figure S161. Dimroth-Reichardt plot showing Stokes shift against $E_T(30)$ parameter for compound **4u**.

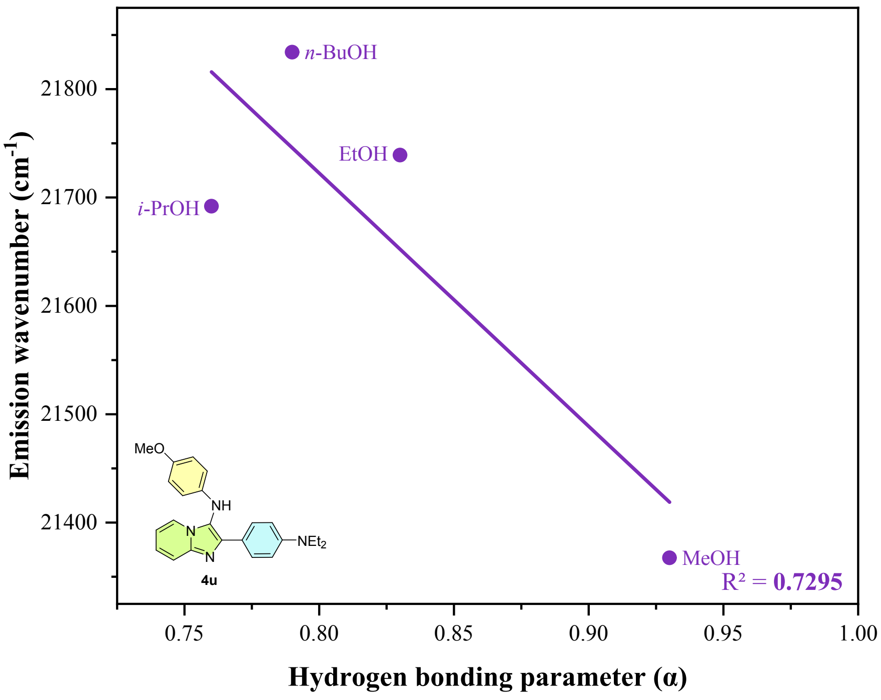

Figure S162. Plot showing hydrogen bonding parameter (α) as a function of emission wavenumber (cm^{-1}) for compound **4u**.

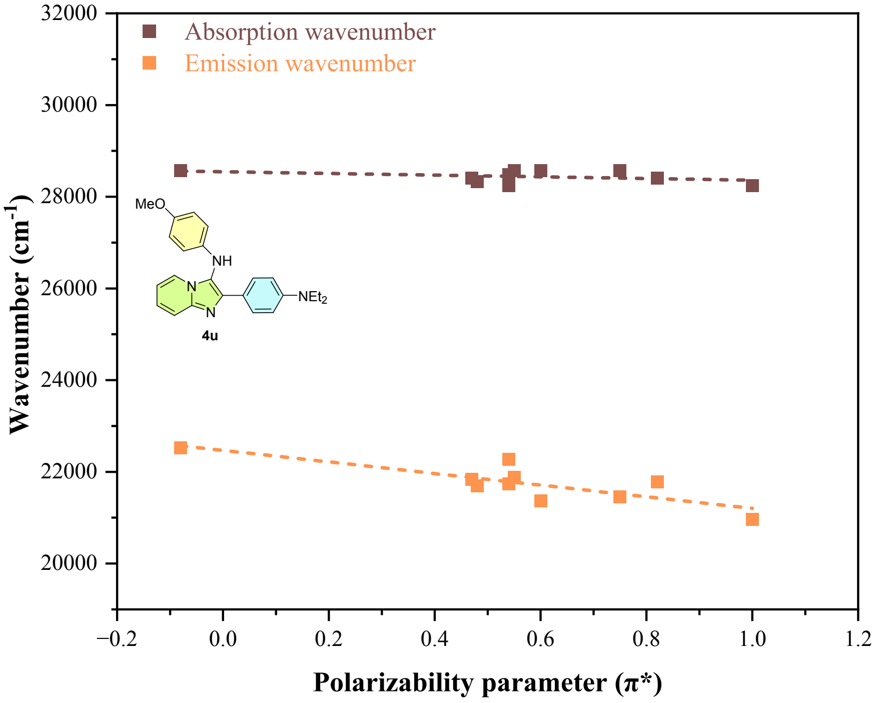

Figure S163. Plot showing polarizability parameter (π*) as a function of absorption and emission wavenumber (cm^{-1}) for compound **4u**.

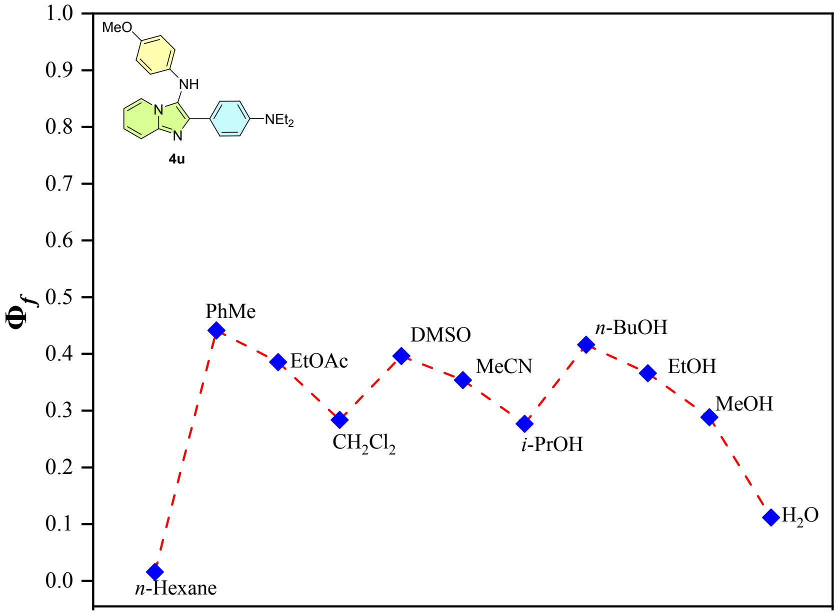

Figure S164. Variation of quantum yields of fluorescence (Φ_f) in different solvents for compound **4u**.

5. Photophysical study in aqueous medium

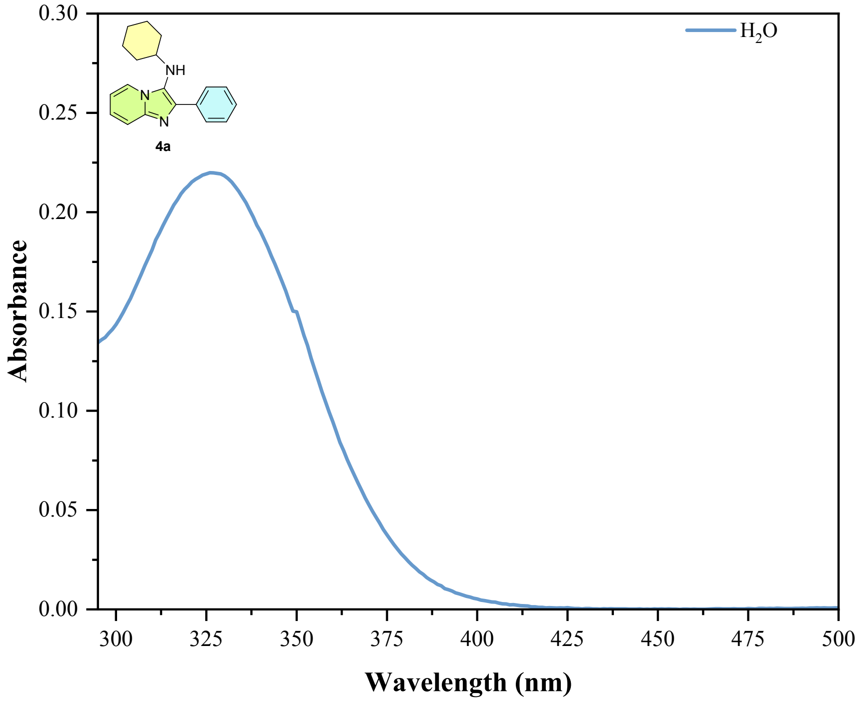

Figure S165. UV-Vis absorption spectra of **4a** in water ($5{\cdot}10^{-5}$ M) at room temperature.

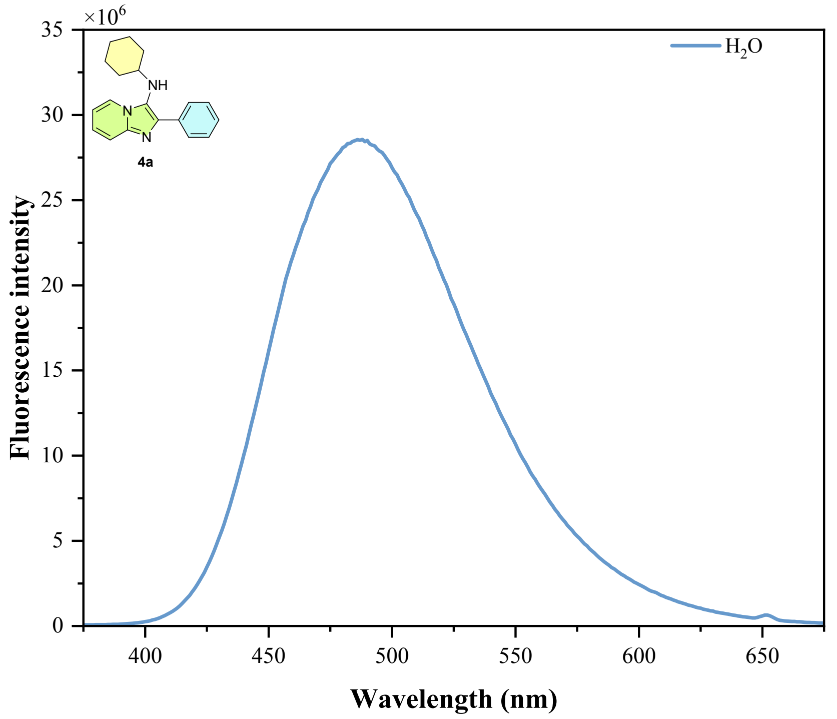

Figure S166. Normalized emission spectra of **4a** in water ($5{\cdot}10^{-5}$ M) at room temperature.

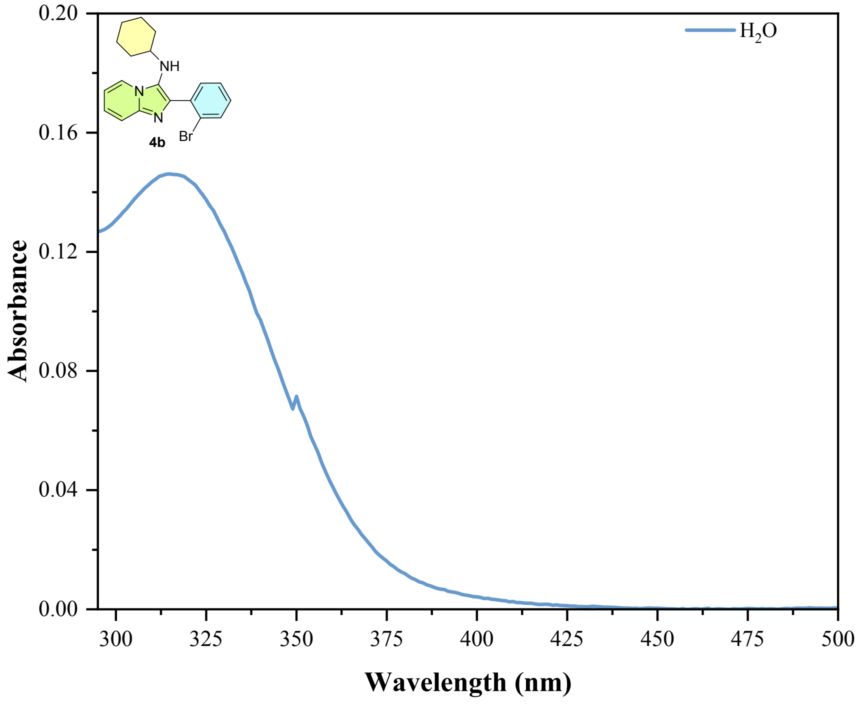

Figure S167. UV-Vis absorption spectra of **4b** in water (5·10^{-5} M) at room temperature.

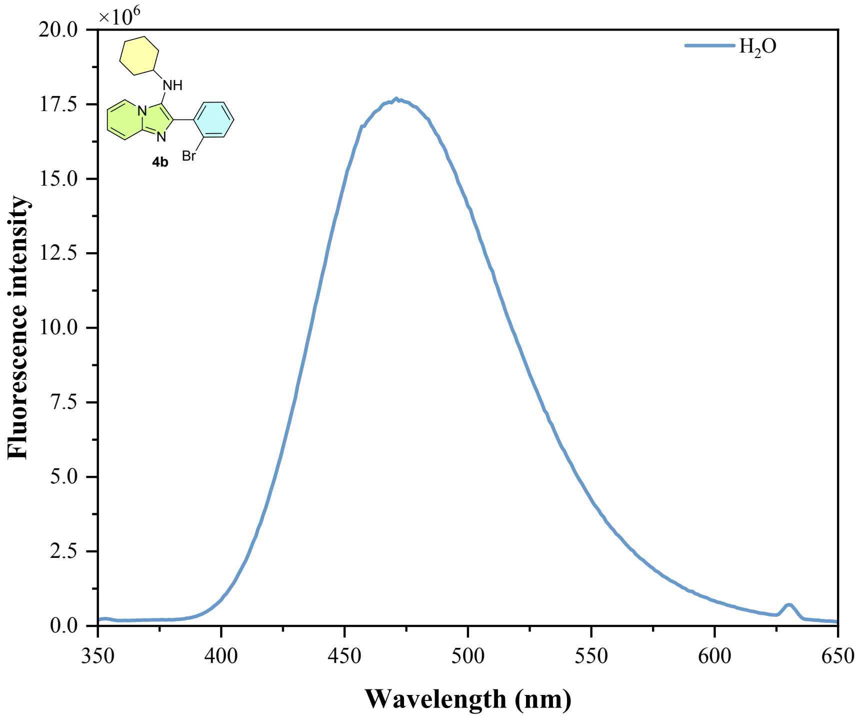

Figure S168. Normalized emission spectra of **4b** in water (5·10^{-5} M) at room temperature.

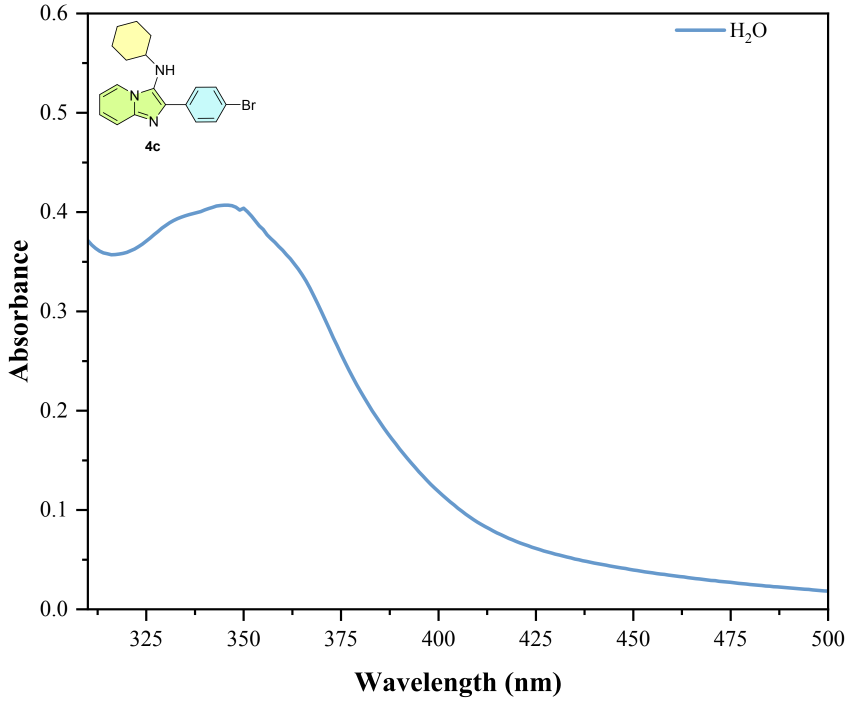

Figure S169. UV-Vis absorption spectra of **4c** in water ($5{\cdot}10^{-5}$ M) at room temperature.

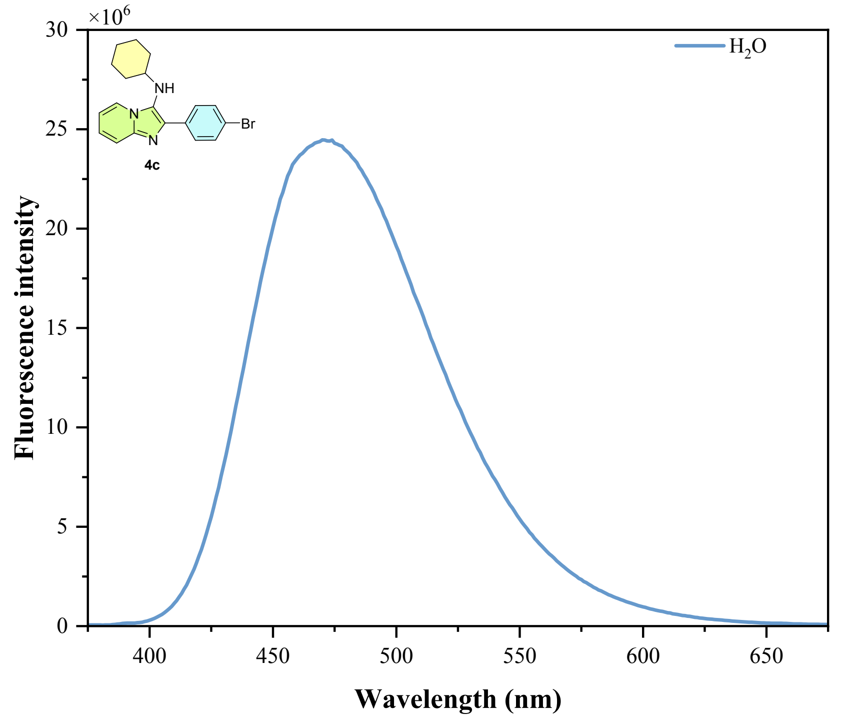

Figure S170. Normalized emission spectra of **4c** in water ($5{\cdot}10^{-5}$ M) at room temperature.

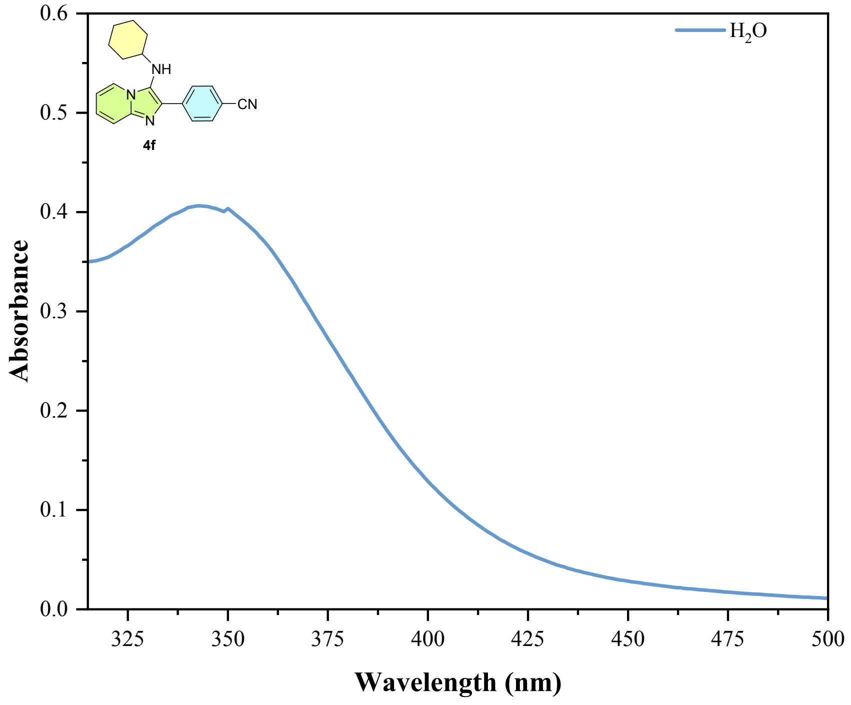

Figure S171. UV-Vis absorption spectra of **4f** in water (5·10^{-5} M) at room temperature.

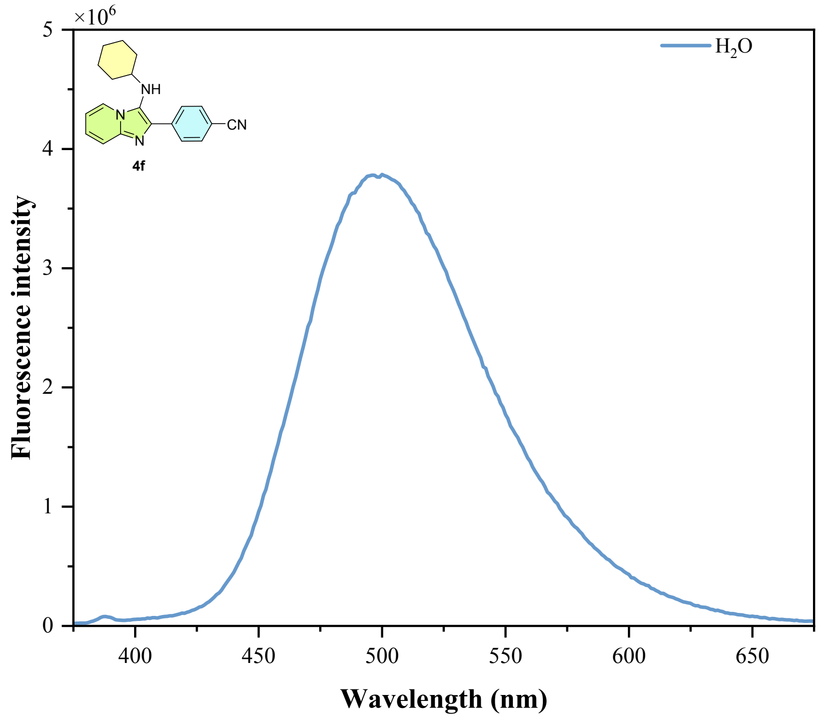

Figure S172. Normalized emission spectra of **4f** in water (5·10^{-5} M) at room temperature.

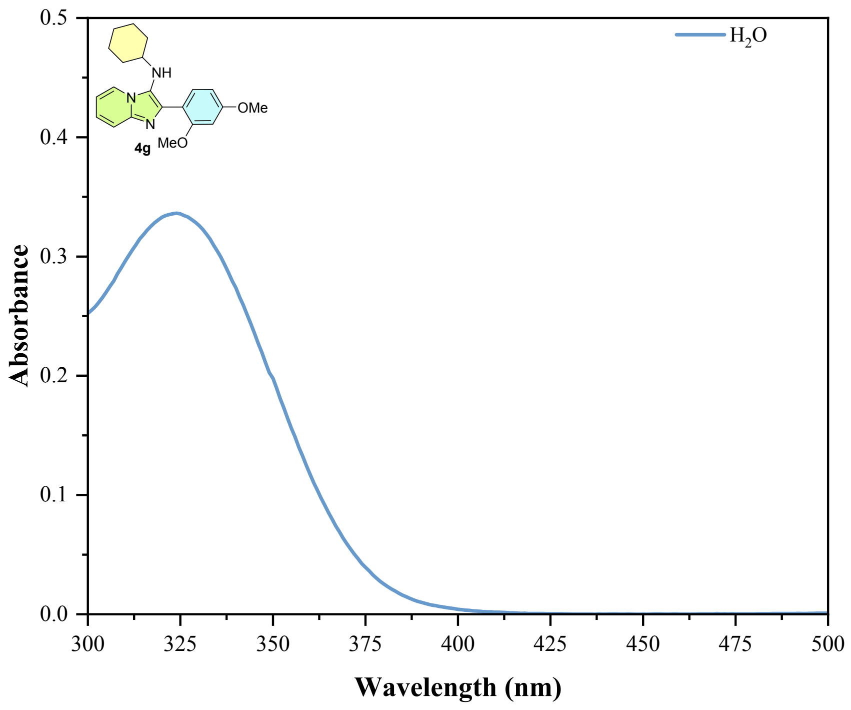

Figure S173. UV-Vis absorption spectra of **4g** in water ($5 \cdot 10^{-5}$ M) at room temperature.

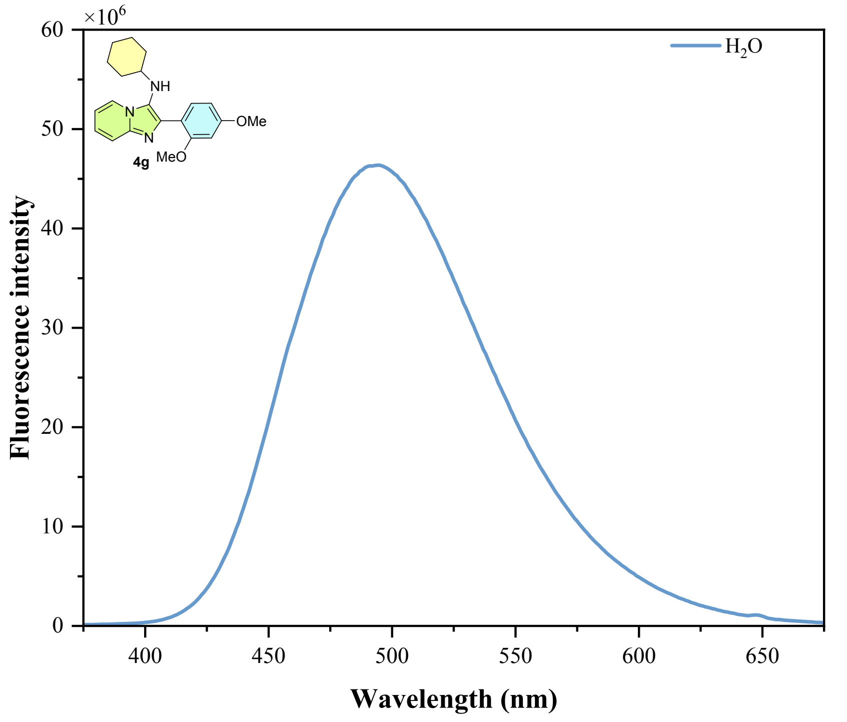

Figure S174. Normalized emission spectra of **4g** in water ($5 \cdot 10^{-5}$ M) at room temperature.

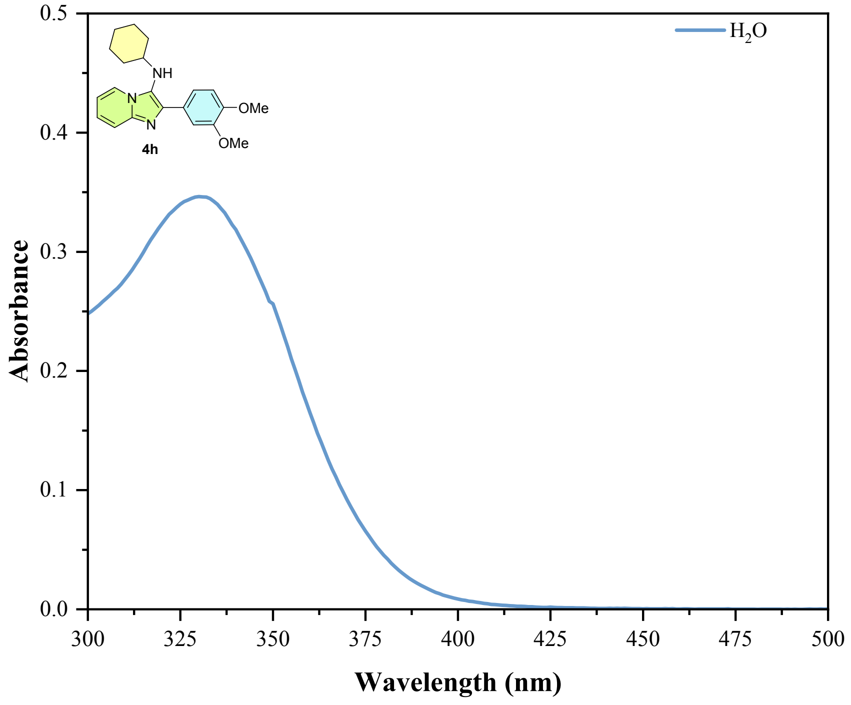

Figure S175. UV-Vis absorption spectra of **4h** in water (5·10^{-5} M) at room temperature.

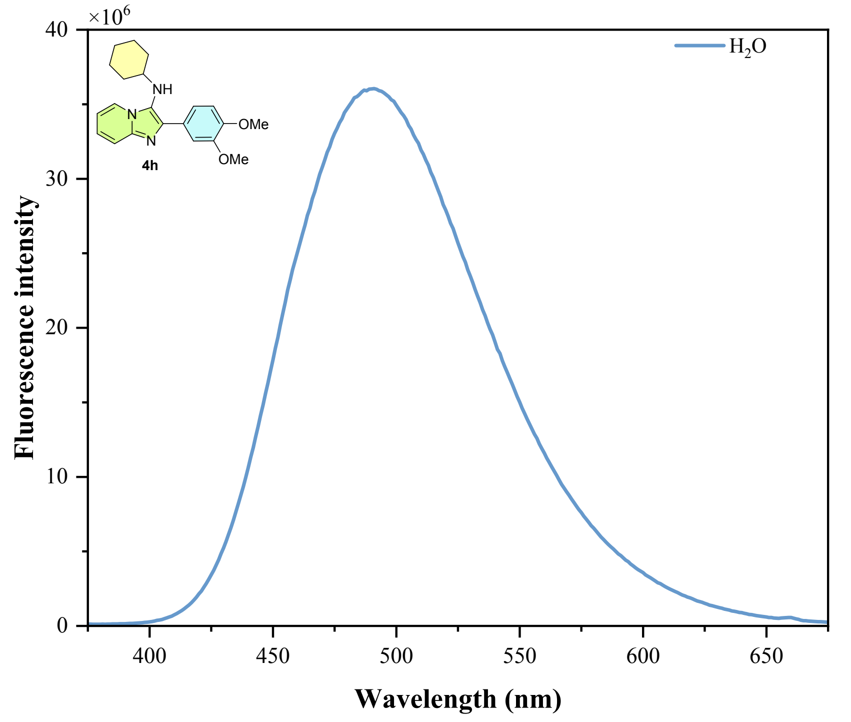

Figure S176. Normalized emission spectra of **4h** in water (5·10^{-5} M) at room temperature.

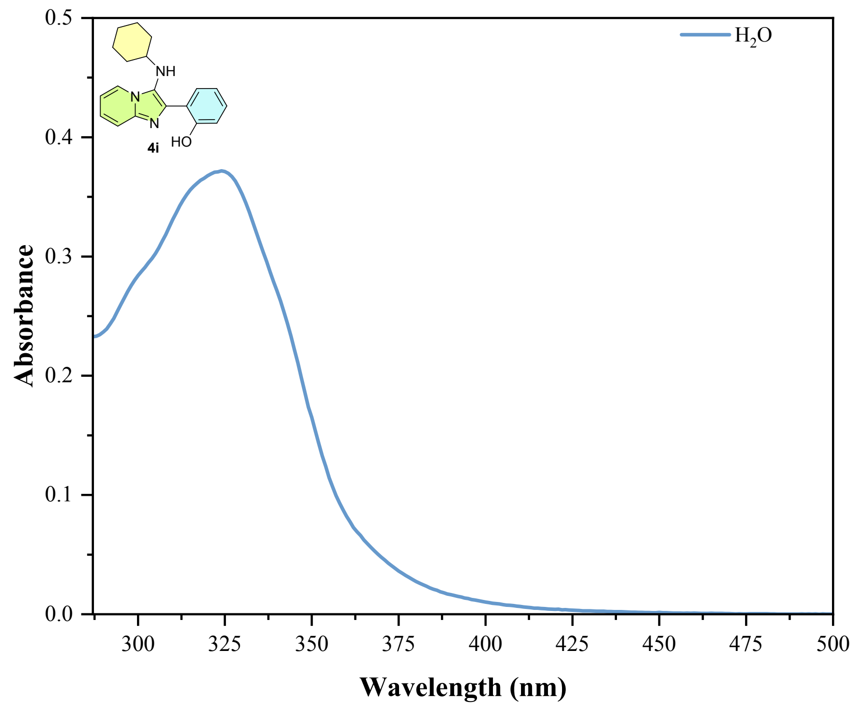

Figure S177. UV-Vis absorption spectra of **4i** in water (5·10^{-5} M) at room temperature.

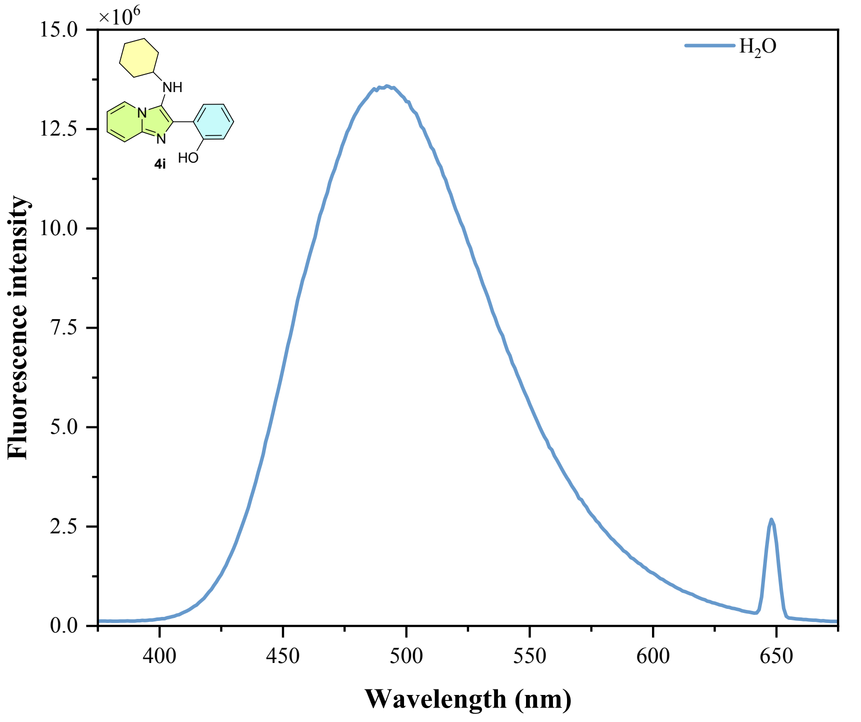

Figure S178. Normalized emission spectra of **4i** in water (5·10^{-5} M) at room temperature.

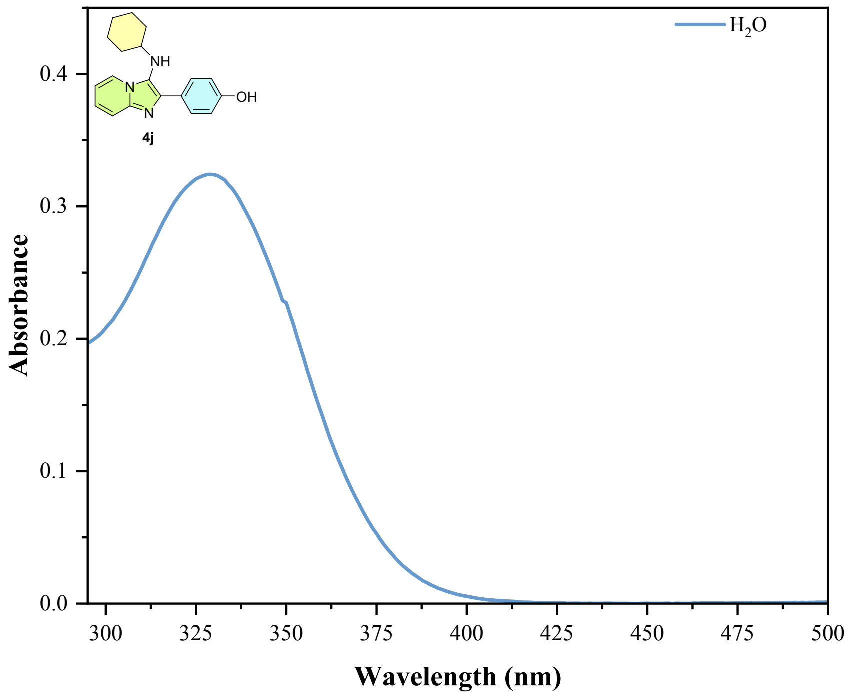

Figure S179. UV-Vis absorption spectra of **4j** in water (5·10^{-5} M) at room temperature.

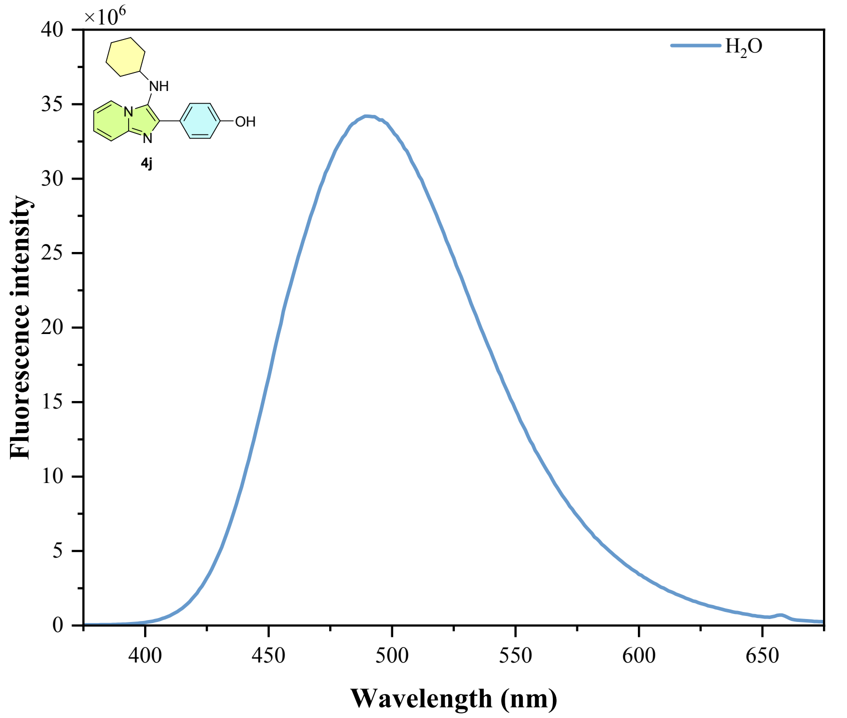

Figure S180. Normalized emission spectra of **4j** in water (5·10^{-5} M) at room temperature.

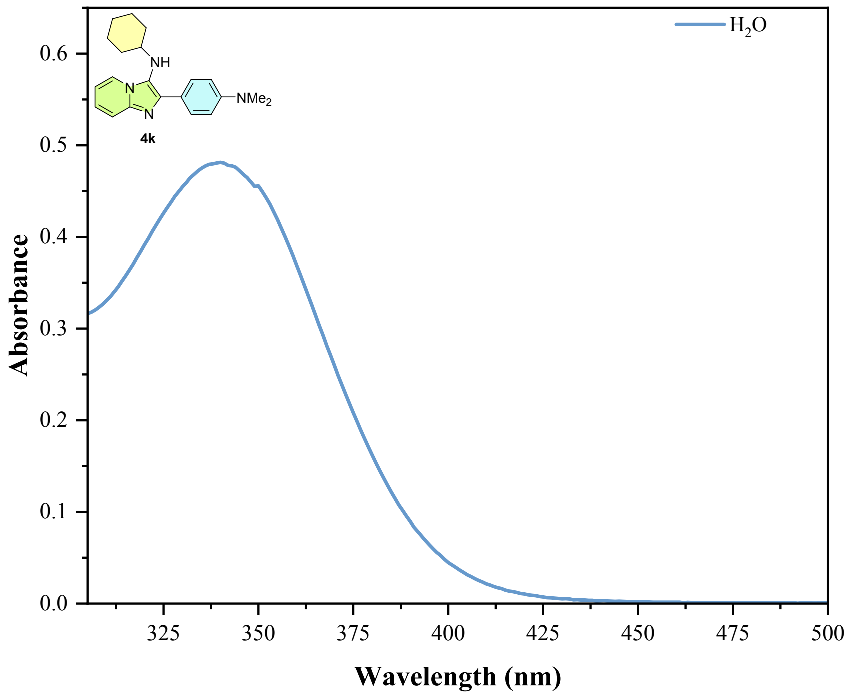

Figure S181. UV-Vis absorption spectra of **4k** in water ($5{\cdot}10^{-5}$ M) at room temperature.

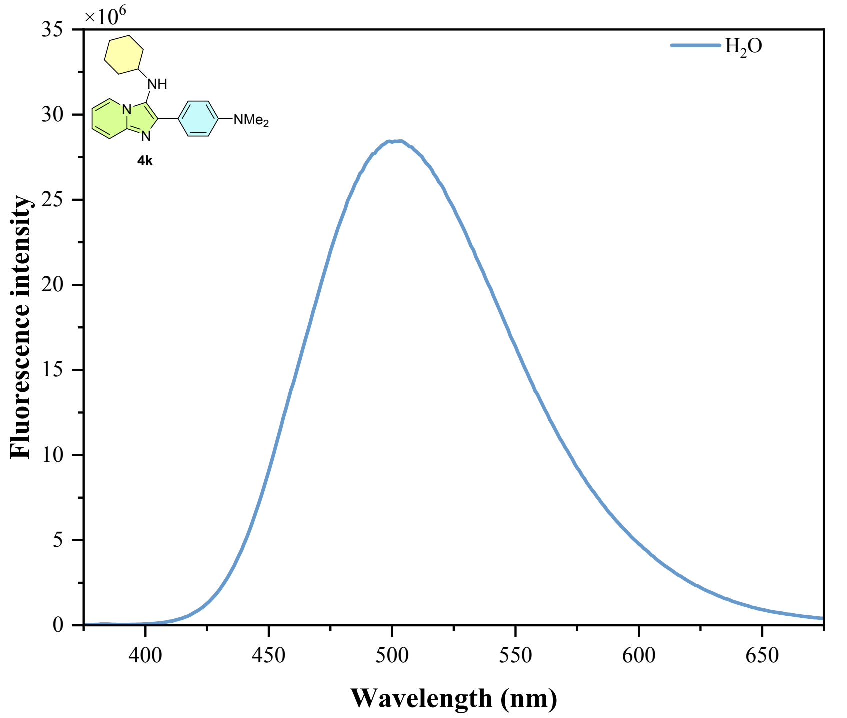

Figure S182. Normalized emission spectra of **4k** in water ($5{\cdot}10^{-5}$ M) at room temperature.

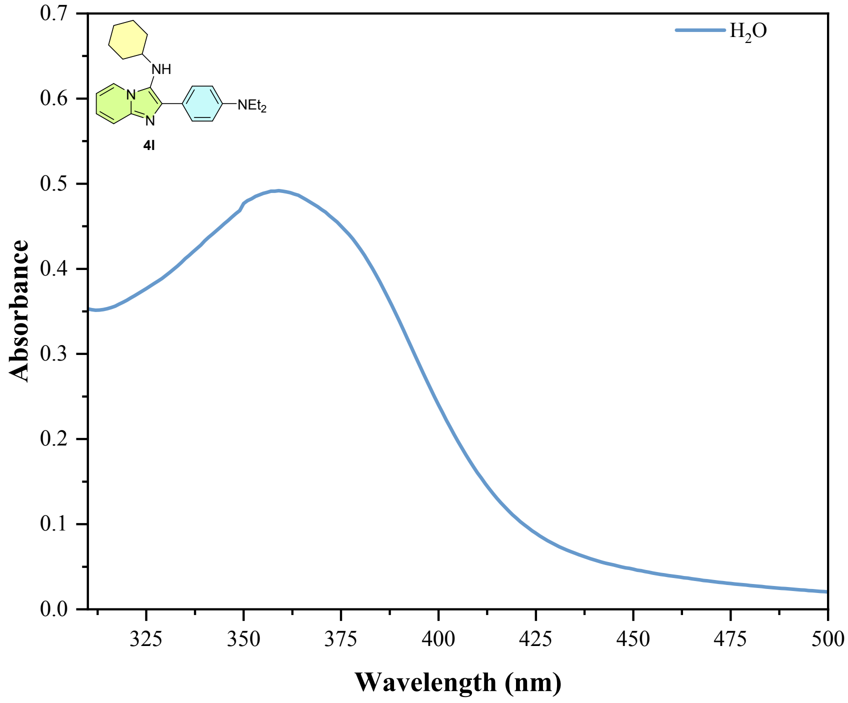

Figure S183. UV-Vis absorption spectra of **4l** in water (5·10^{-5} M) at room temperature.

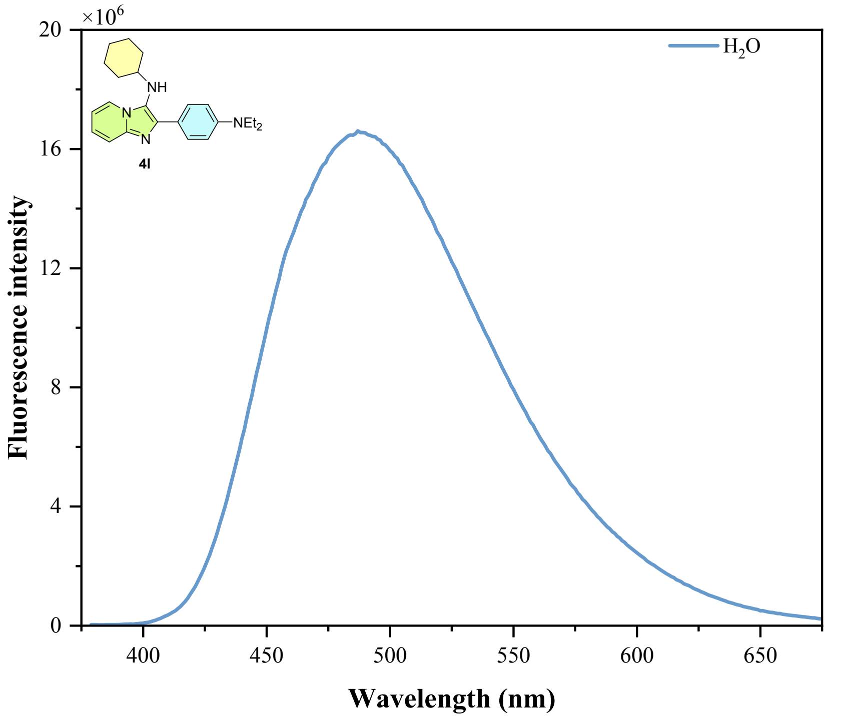

Figure S184. Normalized emission spectra of **4l** in water (5·10^{-5} M) at room temperature.

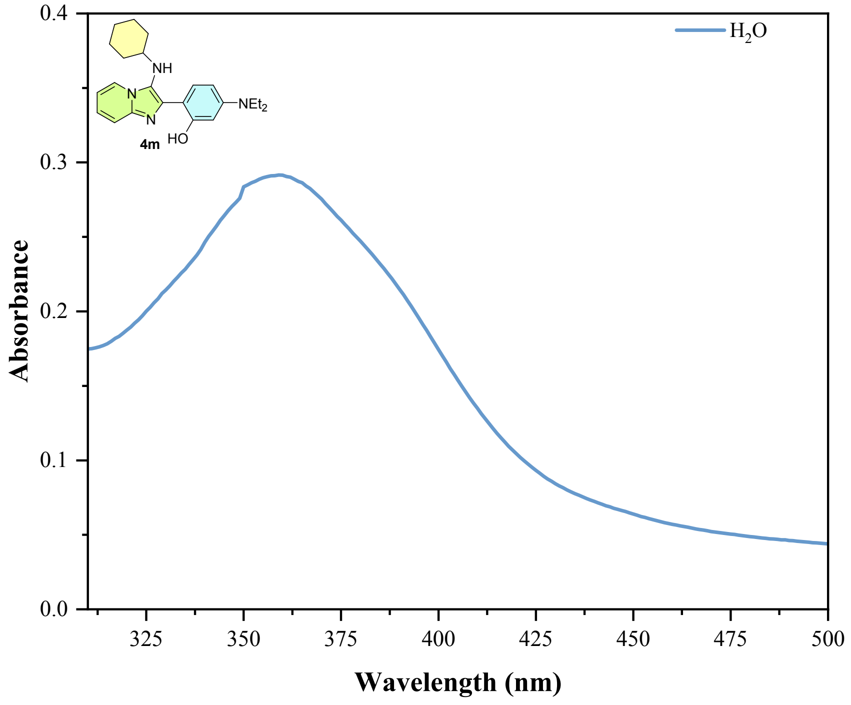

Figure S185. UV-Vis absorption spectra of **4m** in water ($5 \cdot 10^{-5}$ M) at room temperature.

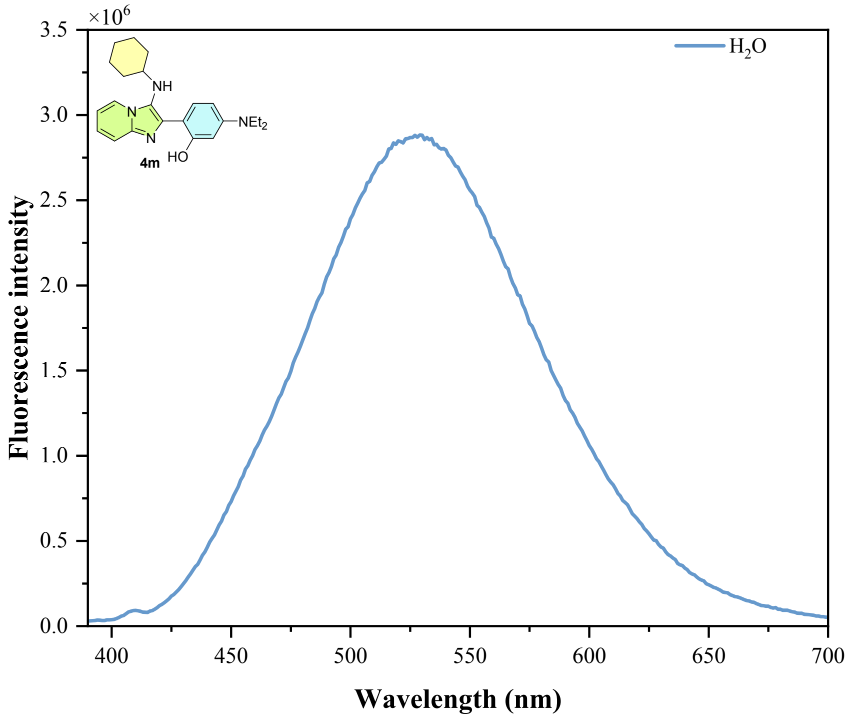

Figure S186. Normalized emission spectra of **4m** in water ($5 \cdot 10^{-5}$ M) at room temperature.

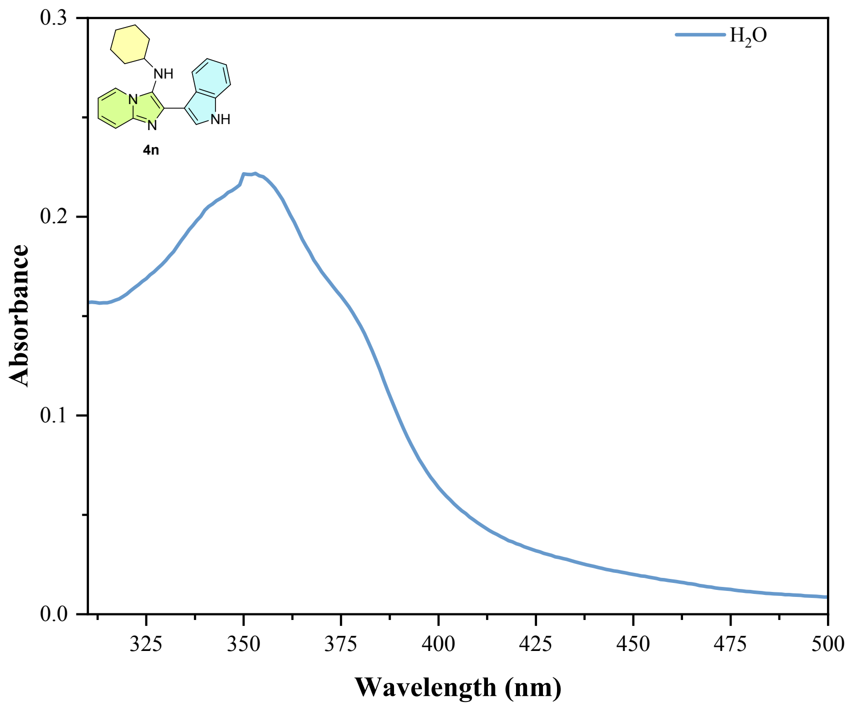

Figure S187. UV-Vis absorption spectra of **4n** in water ($5 \cdot 10^{-5}$ M) at room temperature.

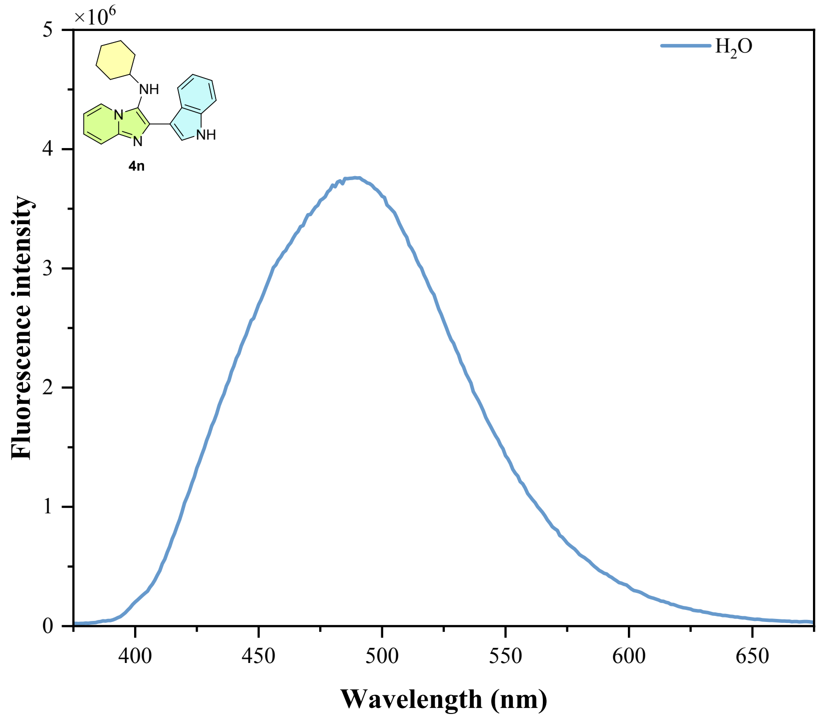

Figure S188. Normalized emission spectra of **4n** in water ($5 \cdot 10^{-5}$ M) at room temperature.

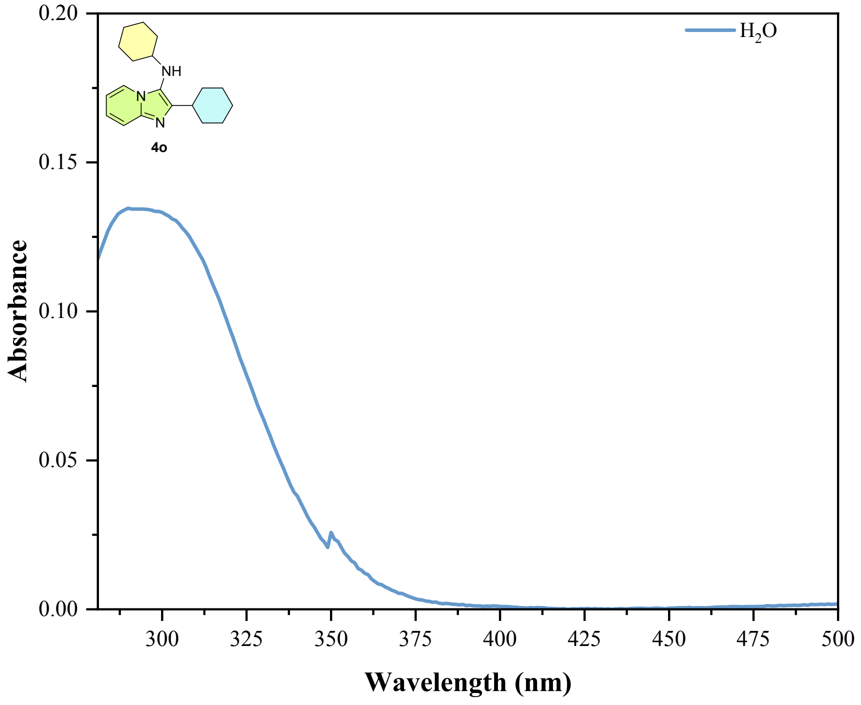

Figure S189. UV-Vis absorption spectra of **4o** in water (5·10^{-5} M) at room temperature.

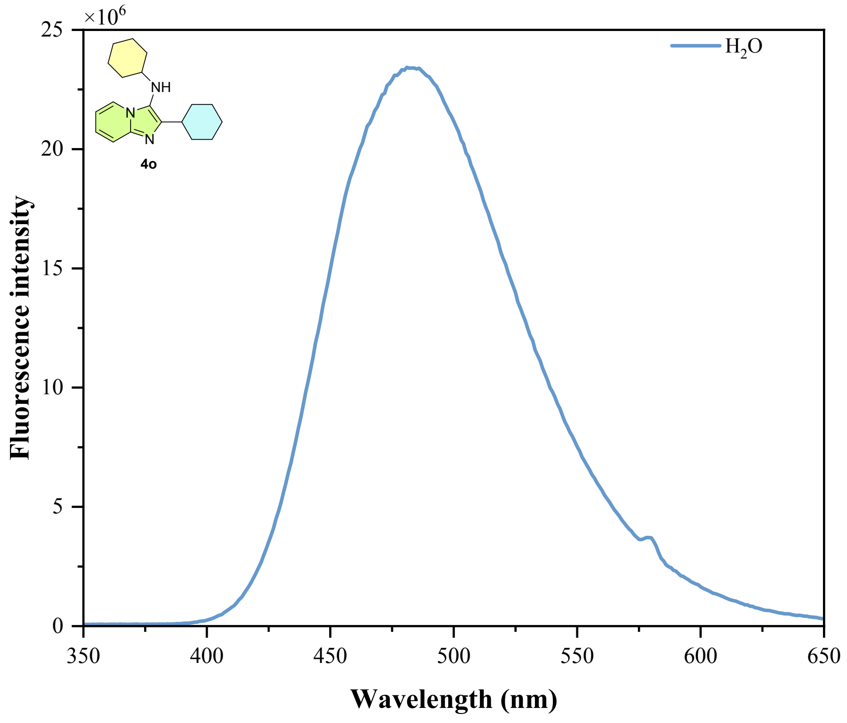

Figure S190. Normalized emission spectra of **4o** in water (5·10^{-5} M) at room temperature.

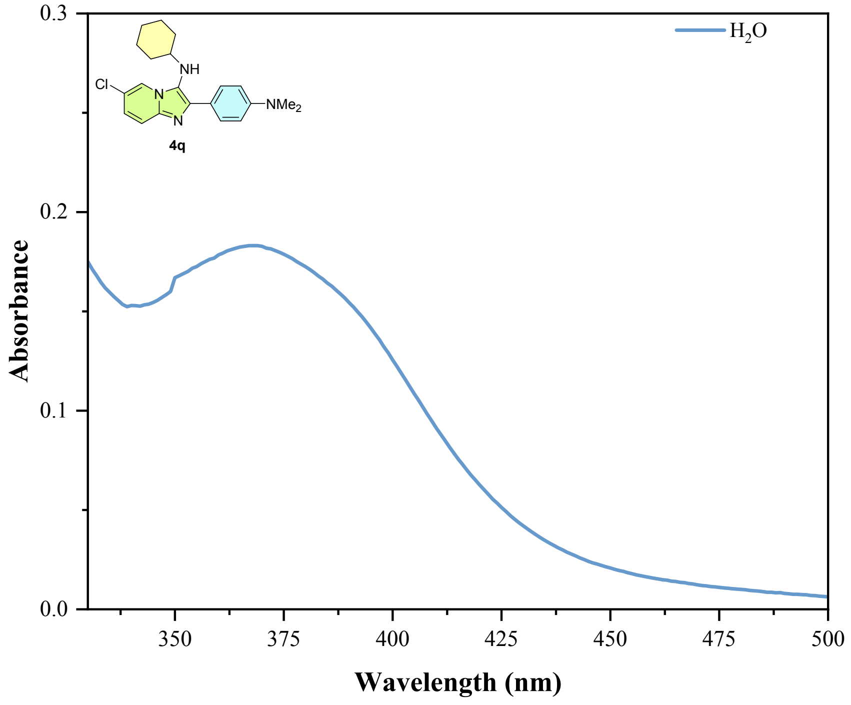

Figure S191. UV-Vis absorption spectra of **4q** in water ($5{\cdot}10^{-5}$ M) at room temperature.

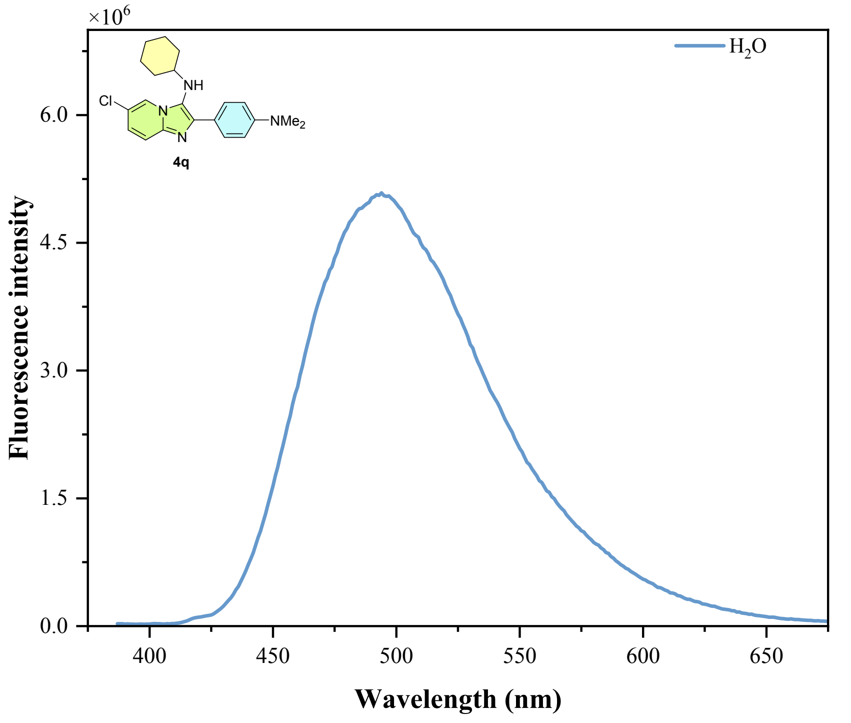

Figure S192. Normalized emission spectra of **4q** in water ($5{\cdot}10^{-5}$ M) at room temperature.

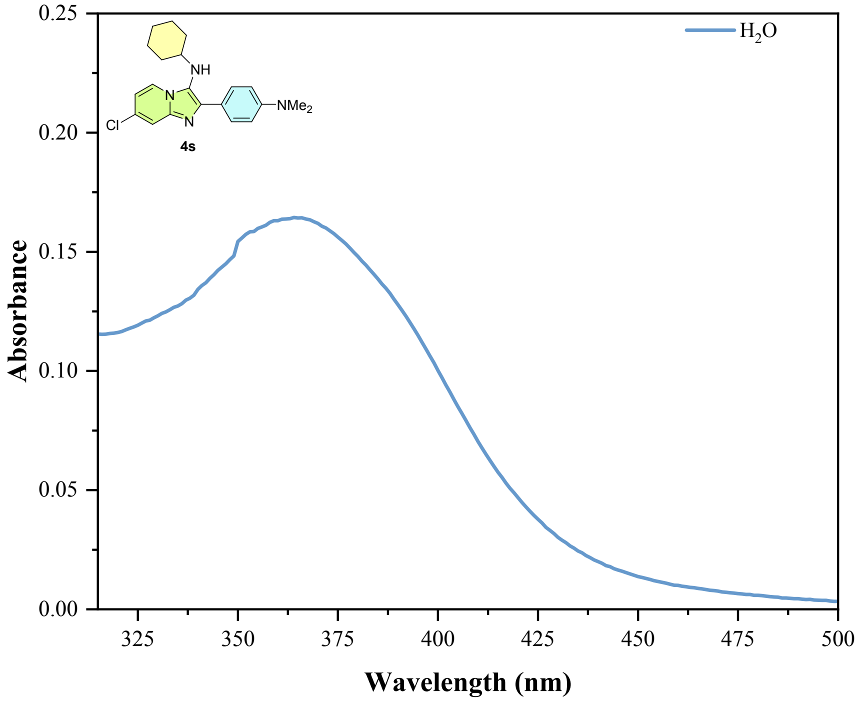

Figure S193. UV-Vis absorption spectra of **4s** in water (5·10^{-5} M) at room temperature.

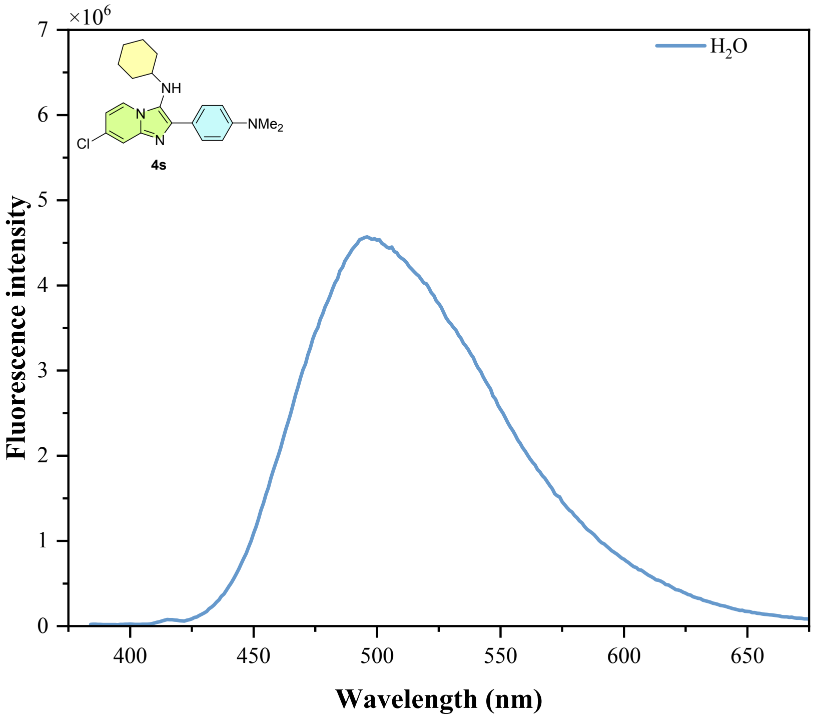

Figure S194. Normalized emission spectra of **4s** in water (5·10^{-5} M) at room temperature.

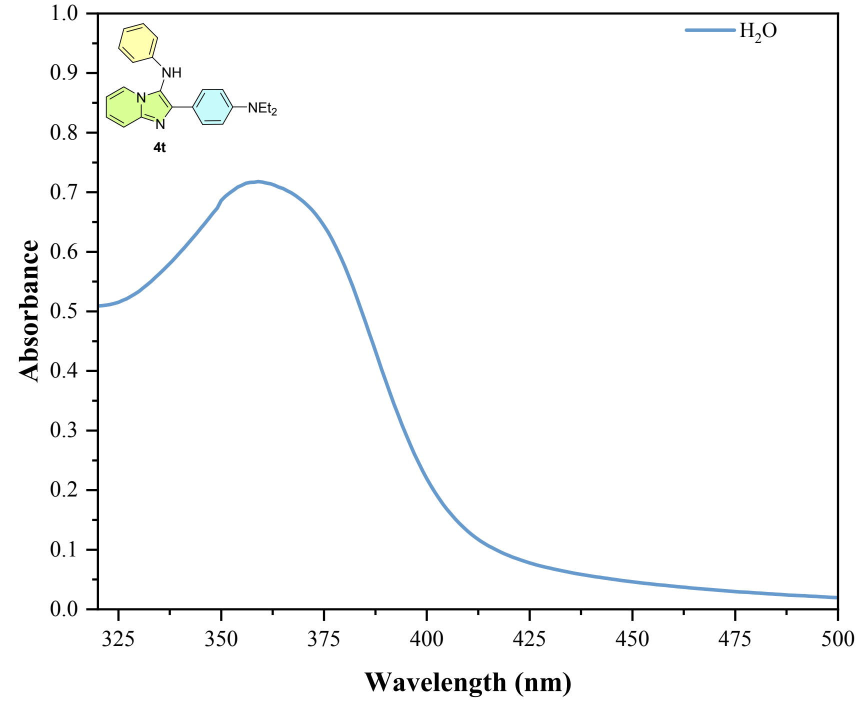

Figure S195. UV-Vis absorption spectra of **4t** in water (5·10^{-5} M) at room temperature.

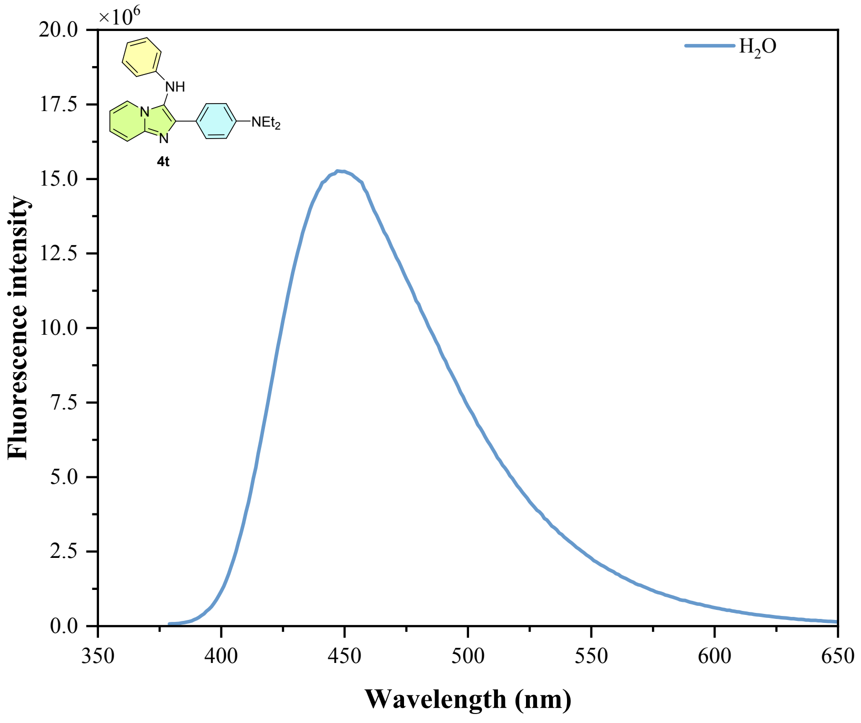

Figure S196. Normalized emission spectra of **4t** in water (5·10^{-5} M) at room temperature.

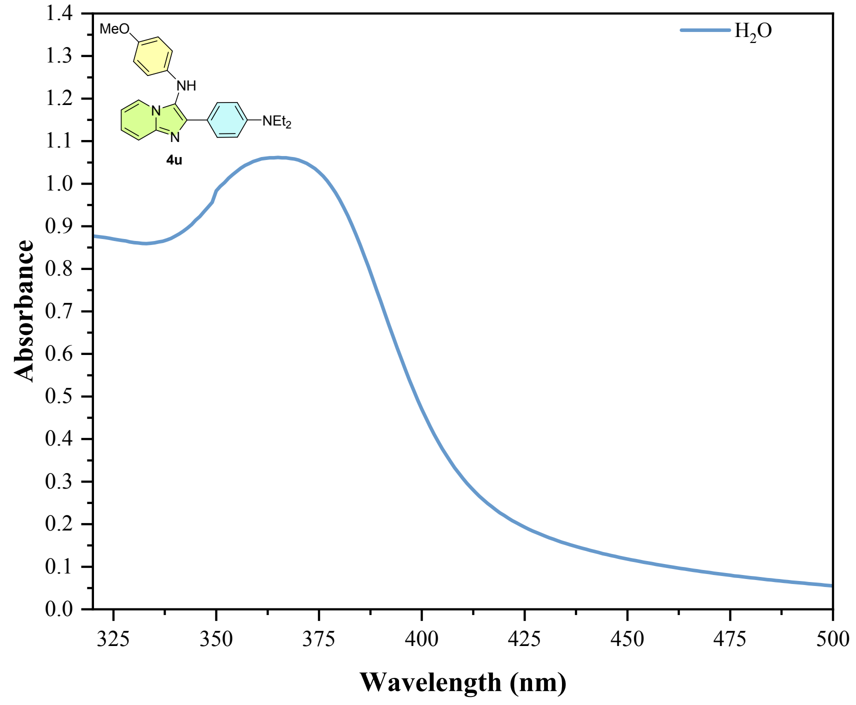

Figure S197. UV-Vis absorption spectra of **4u** in water (5·10^{-5} M) at room temperature.

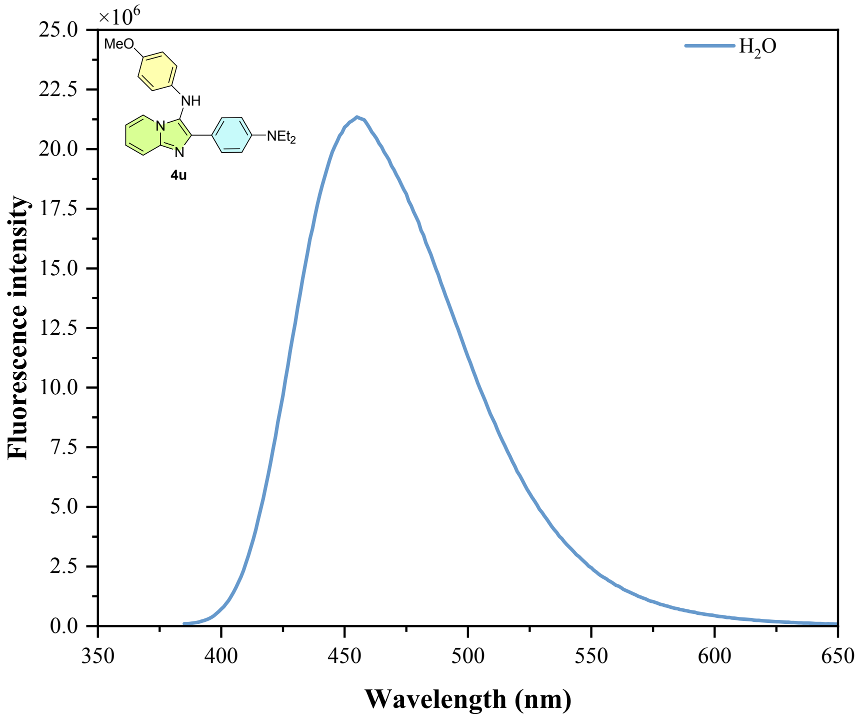

Figure S198. Normalized emission spectra of **4u** in water (5·10^{-5} M) at room temperature.

5.1 Influence of pH on the absorption and fluorescence spectra in aqueous solution

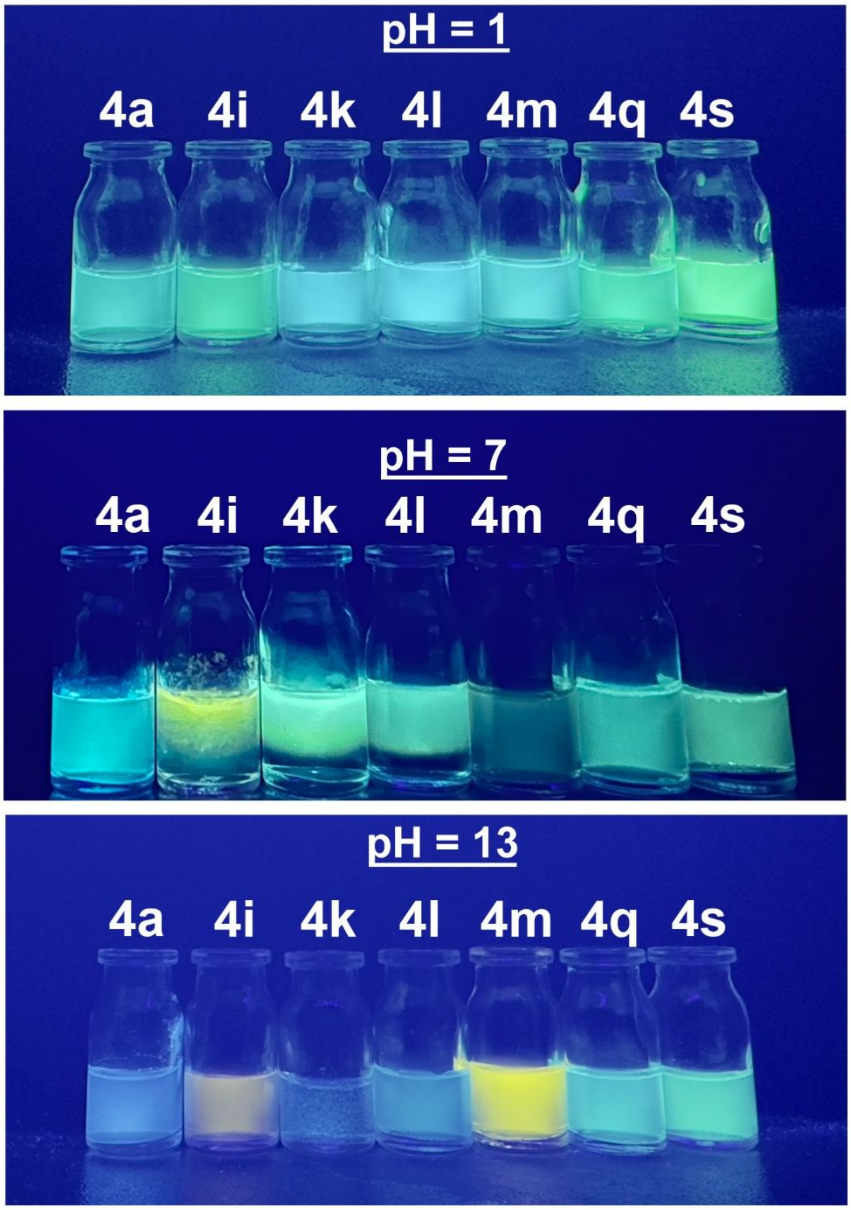

Figure S199. Emission for selected compounds in aqueous solution under different pH values ($5 \cdot 10^{-5}$ M) at room temperature; λ_{exc} = 365 nm. in a dark chamber with an UV lamp.

Table S5. Photophysical properties of selected imidazo[1.2-*a*]pyridine compounds in aqueous solution under different pH values.[a]

Comp.	pH[b]	λ_{abs}, nm[c]	λ_{em}, nm[d]	log ε (ε, $M^{-1} \cdot cm^{-1}$)[e]	Stokes Shift, cm^{-1}[f]
**4a**	1	312	480	3.90 (7886)	11218
	7	326	488	3.64 (4397)	10183
	13	350	454	2.67 (466)	6545
**4i**	1	315	489	4.01 (10250)	11296

	7	324	492	3.87 (7437)	10539
	13	350	550	4.05 (11154)	10390
**4k**	1	311	471	4.01 (10298)	10923
	7	340	503	3.98 (9626)	9531
	13	350	483	3.21 (1639)	7867
**4l**	1	312	471	3.98 (9526)	10820
	7	359	487	3.99 (9837)	7321
	13	392	472	3.80 (6248)	4324
**4m**	1	315	479	4.04 (11009)	10869
	7	359	529	3.77 (5832)	8952
	13	358	540	4.18 (15170)	9414
**4q**	1	317	488	4.00 (10105)	11054
	7	367	494	3.56 (3663)	7005
	13	392	489	3.81 (6389)	5060
**4s**	1	320	493	4.12 (13146)	10966
	7	364	496	3.52 (3288)	7311
	13	391	495	3.79 (6131)	5373

[a] Carried out at room temperature ($5 \cdot 10^{-5}$ M). [b] Measurements were performed in solutions of 1 M HCl (pH = 1), distilled water (pH = 7) and 1 M NaOH (pH = 13). [c] λ_{abs} = absorption maxima (nm). [d] λ_{em} = emission maxima (nm). [e] ε = molar absorptivity ($M^{-1} \cdot cm^{-1}$). [f] Stokes shifts difference between λ_{em} and λ_{abs}.

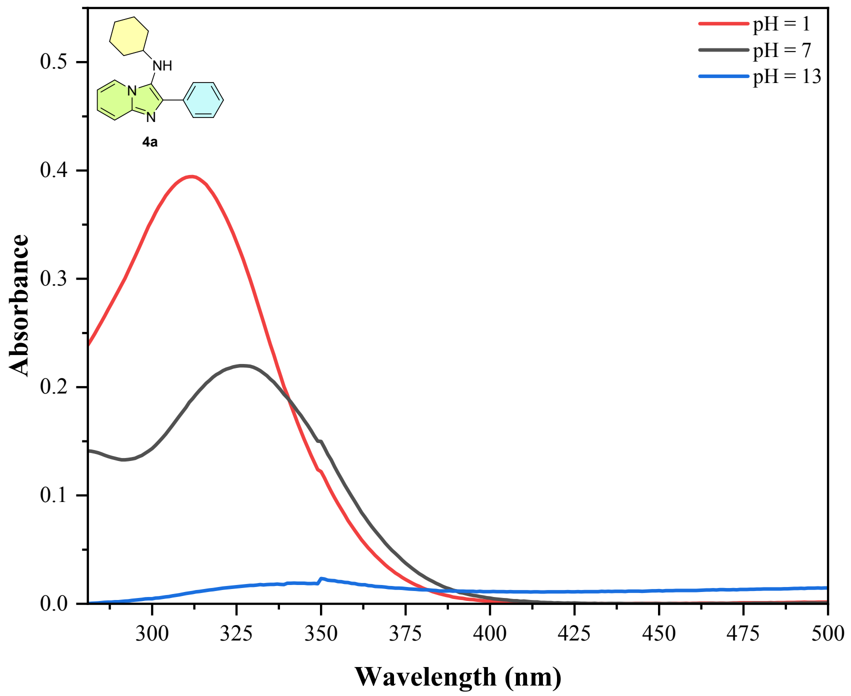

Figure S200. UV-Vis absorption spectra of **4a** in aqueous solution under different pH values ($5{\cdot}10^{-5}$ M) at room temperature.

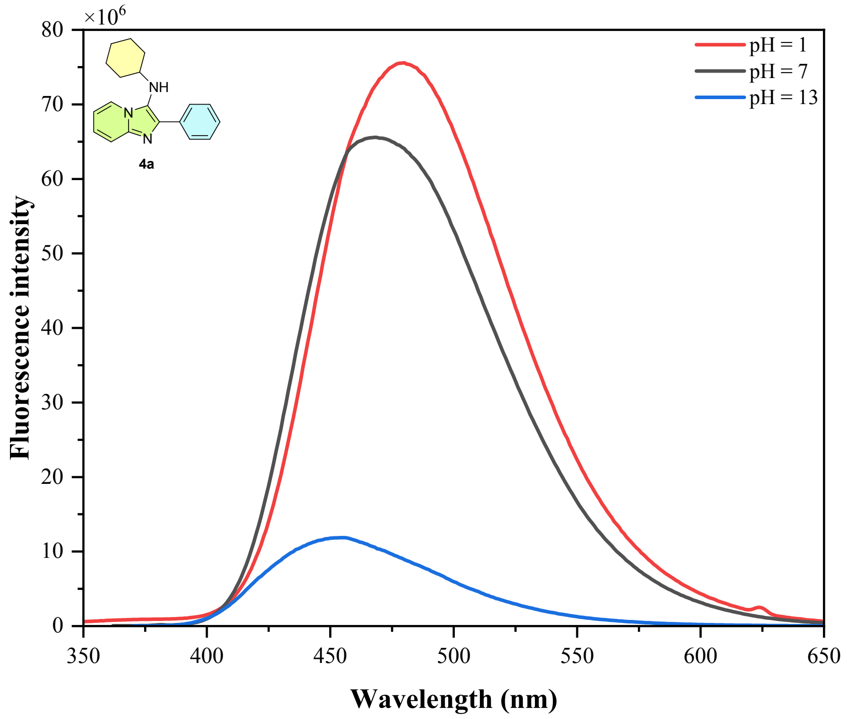

Figure S201. Normalized emission spectra of **4a** in aqueous solution under different pH values ($5{\cdot}10^{-5}$ M) at room temperature.

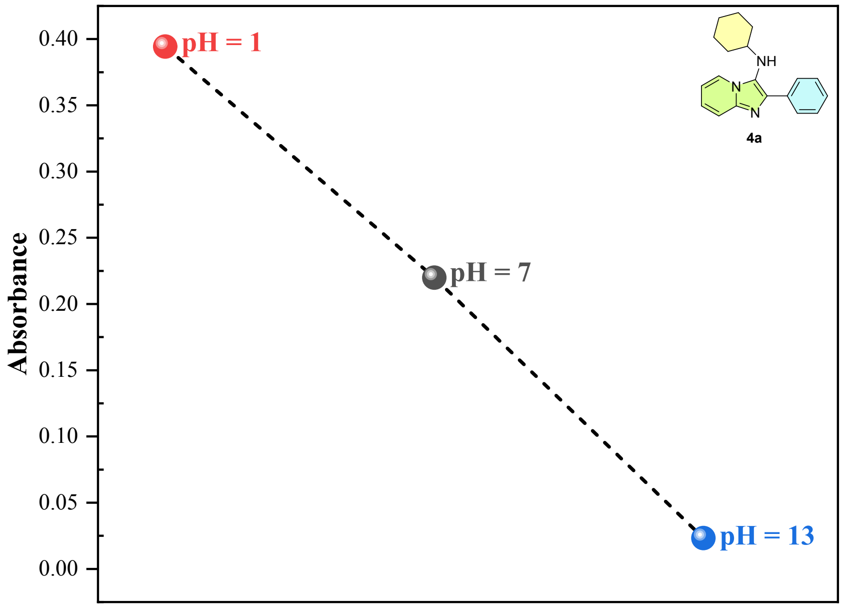

Figure S202. Plot showing different pH values as a function of absorbance for compound **4a**.

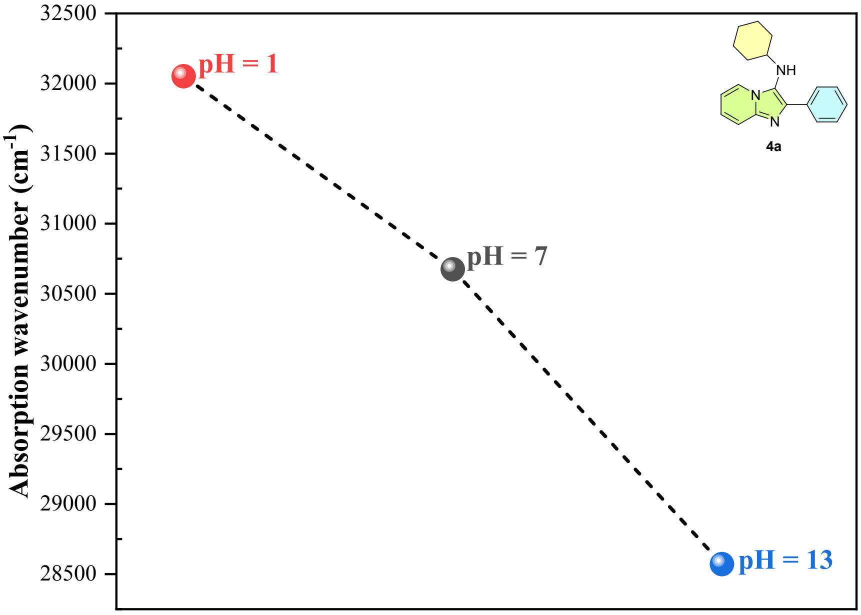

Figure S203. Plot showing different pH values as a function of absorption wavenumber (cm^{-1}) for compound **4a**.

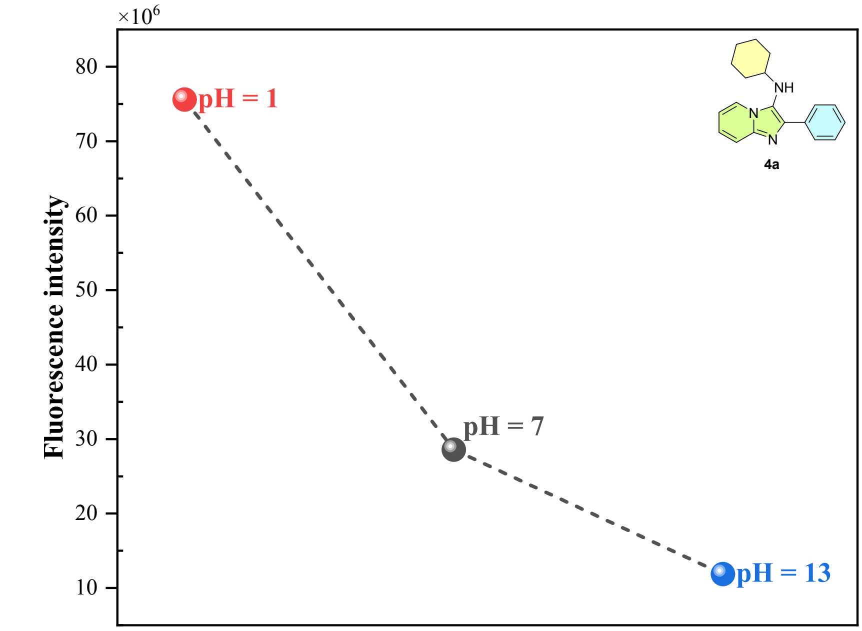

Figure S204. Plot showing different pH values as a function of fluorescence intensity for compound **4a**.

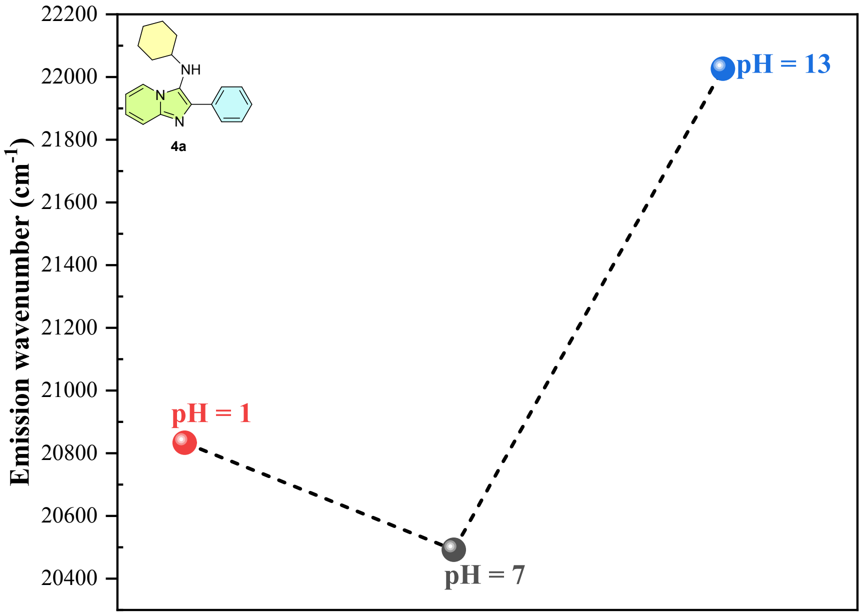

Figure S205. Plot showing different pH values as a function of emission wavenumber (cm^{-1}) for compound **4a**.

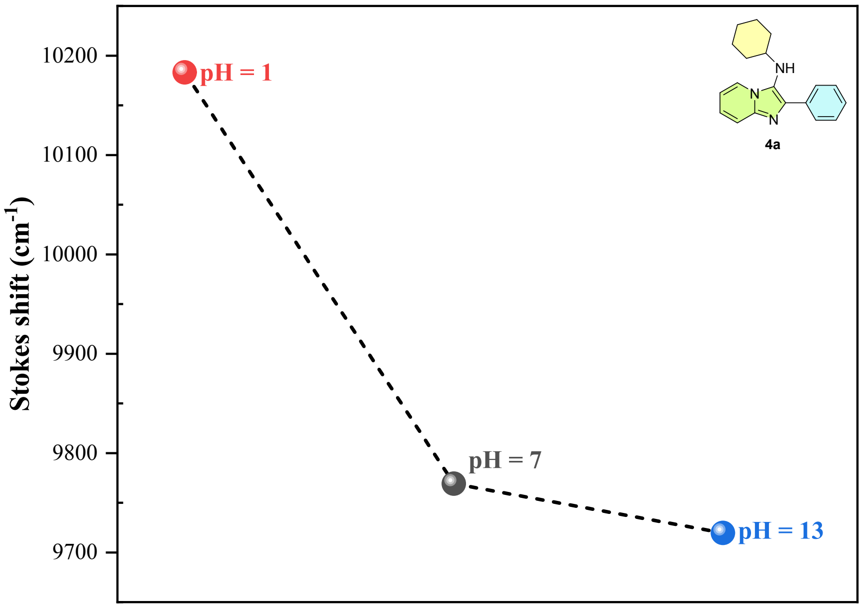

Figure S206. Plot showing different pH values as a function of Stokes shift (cm^{-1}) for compound **4a**.

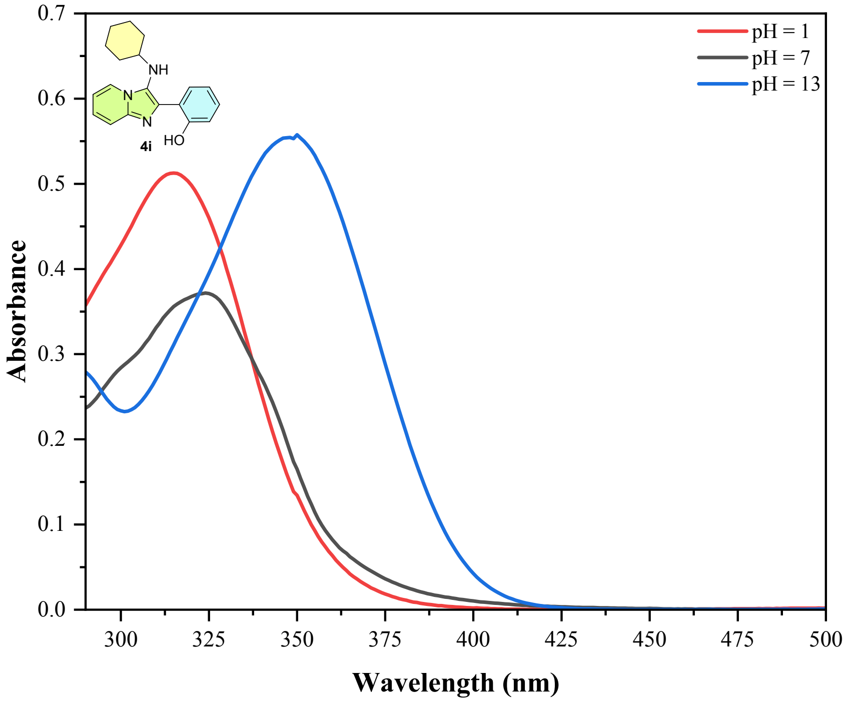

Figure S207. UV-Vis absorption spectra of **4i** in aqueous solution under different pH values ($5{\cdot}10^{-5}$ M) at room temperature.

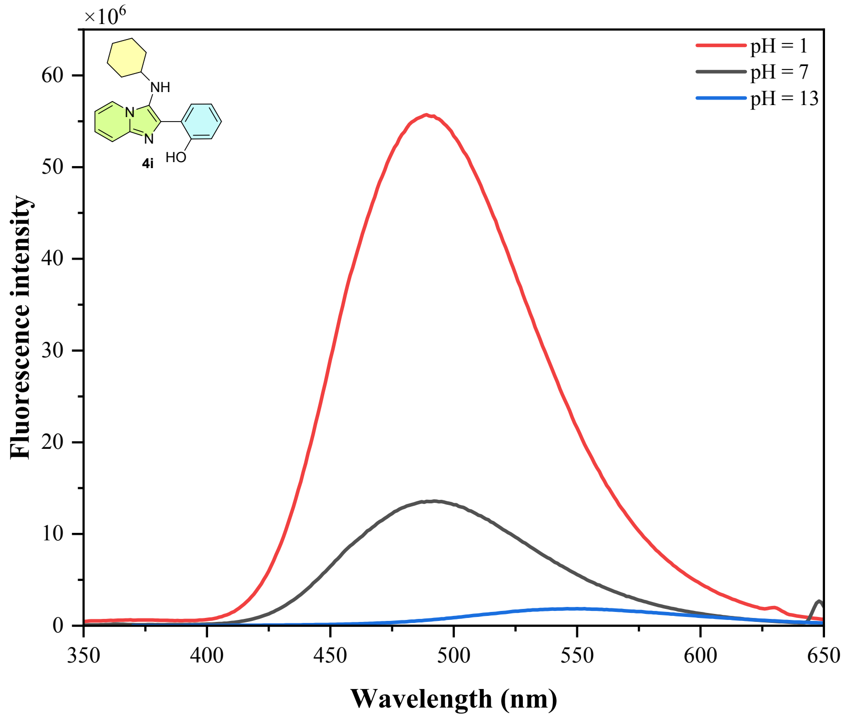

Figure S208. Normalized emission spectra of **4i** in aqueous solution under different pH values ($5{\cdot}10^{-5}$ M) at room temperature.

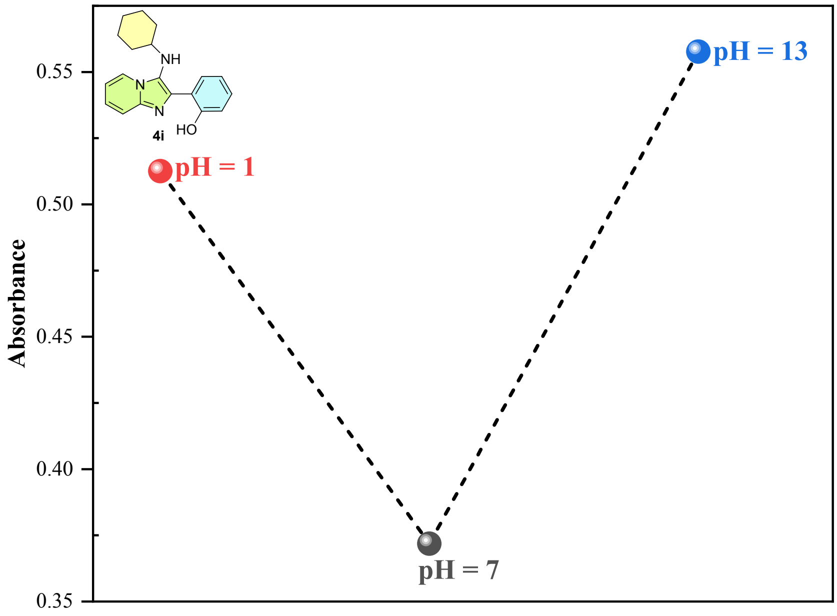

Figure S209. Plot showing different pH values as a function of absorbance for compound **4i**.

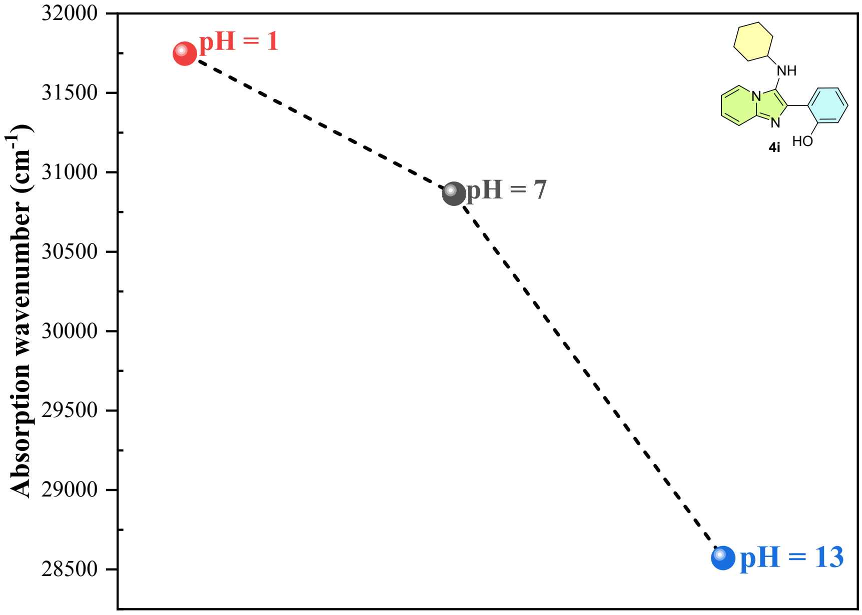

Figure S210. Plot showing different pH values as a function of absorption wavenumber (cm^{-1}) for compound **4i**.

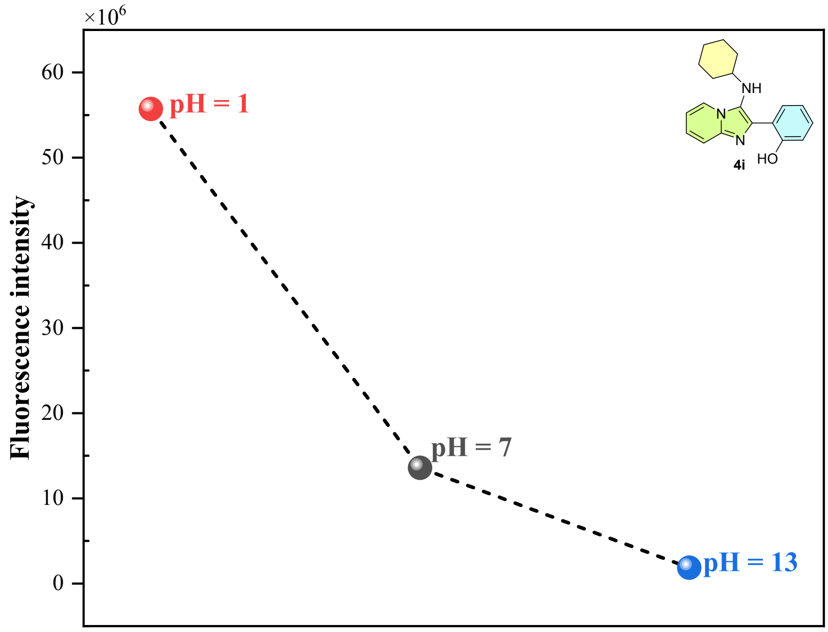

Figure S211. Plot showing different pH values as a function of fluorescence intensity for compound **4i**.

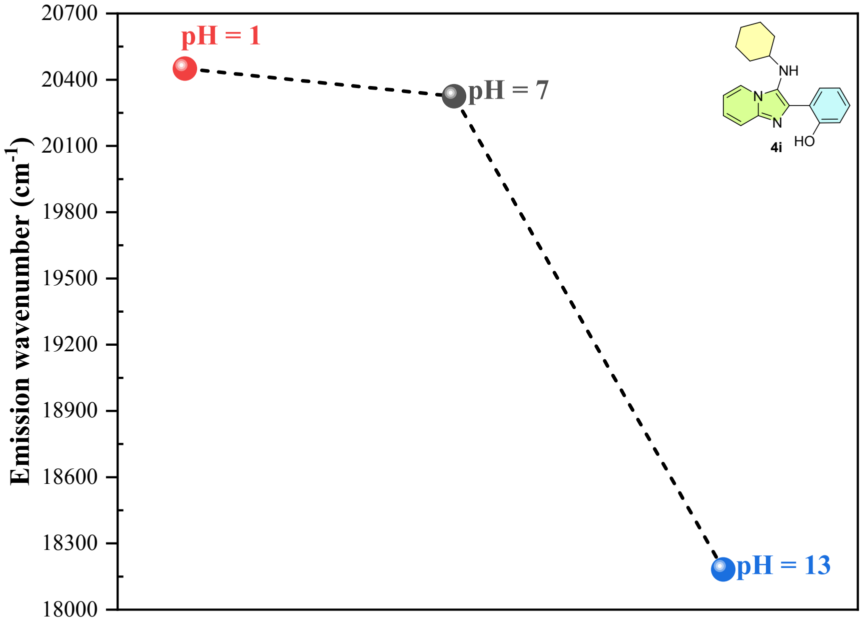

Figure S212. Plot showing different pH values as a function of emission wavenumber (cm^{-1}) for compound **4i**.

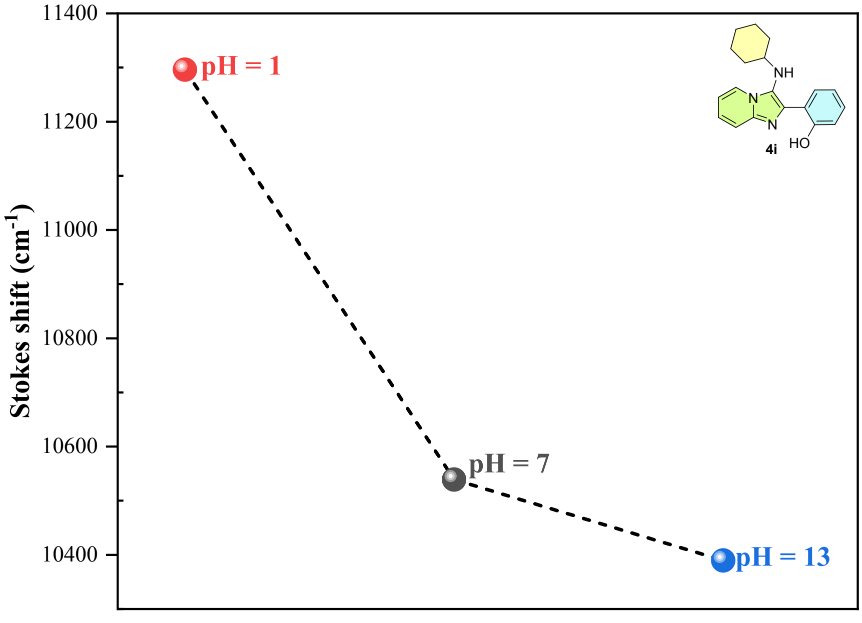

Figure S213. Plot showing different pH values as a function of Stokes shift (cm^{-1}) for compound **4i**.

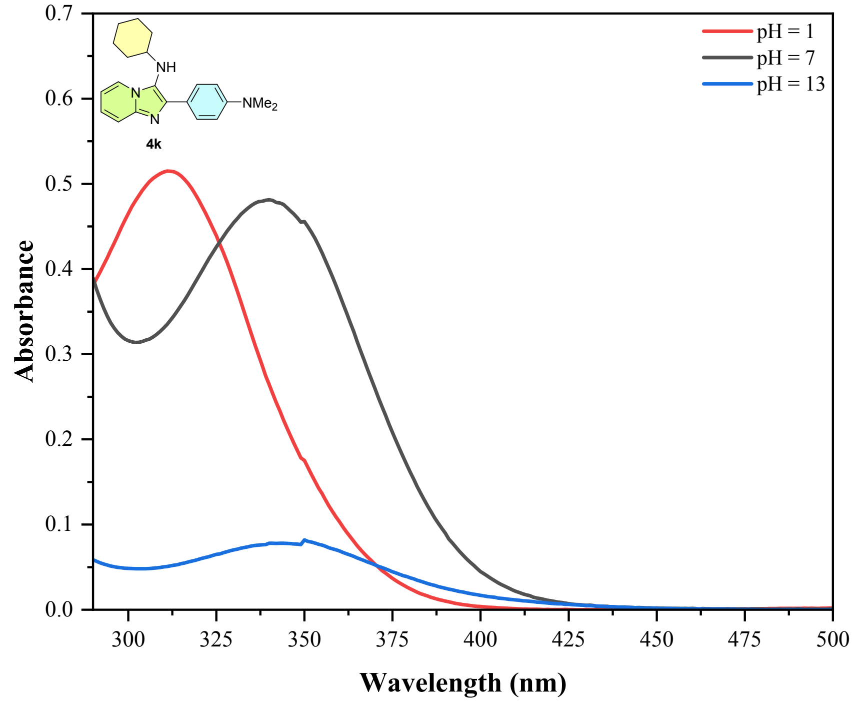

Figure S214. UV-Vis absorption spectra of **4k** in aqueous solution under different pH values (5·10^{-5} M) at room temperature.

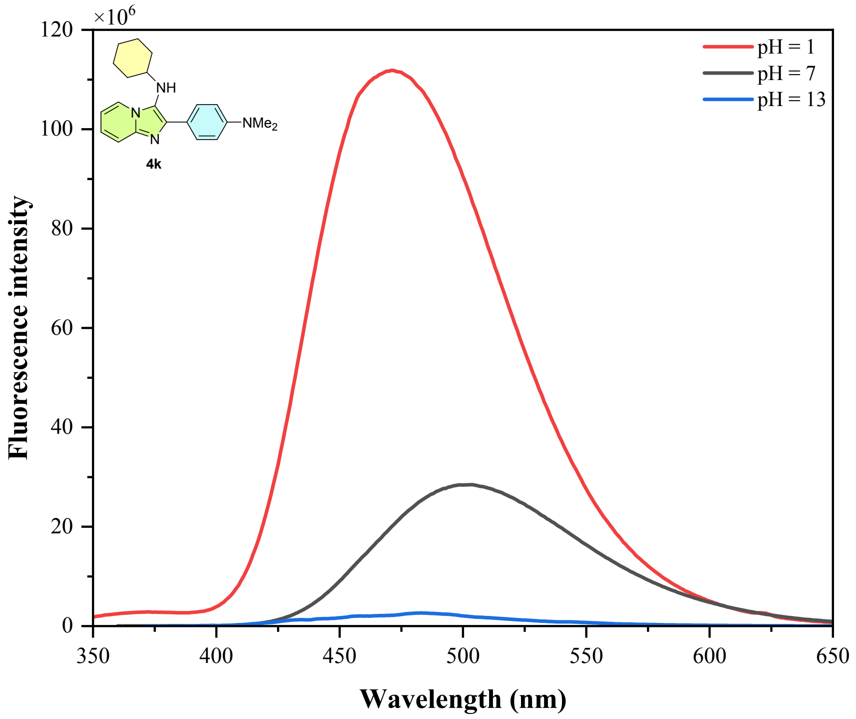

Figure S215. Normalized emission spectra of **4k** in aqueous solution under different pH values (5·10^{-5} M) at room temperature.

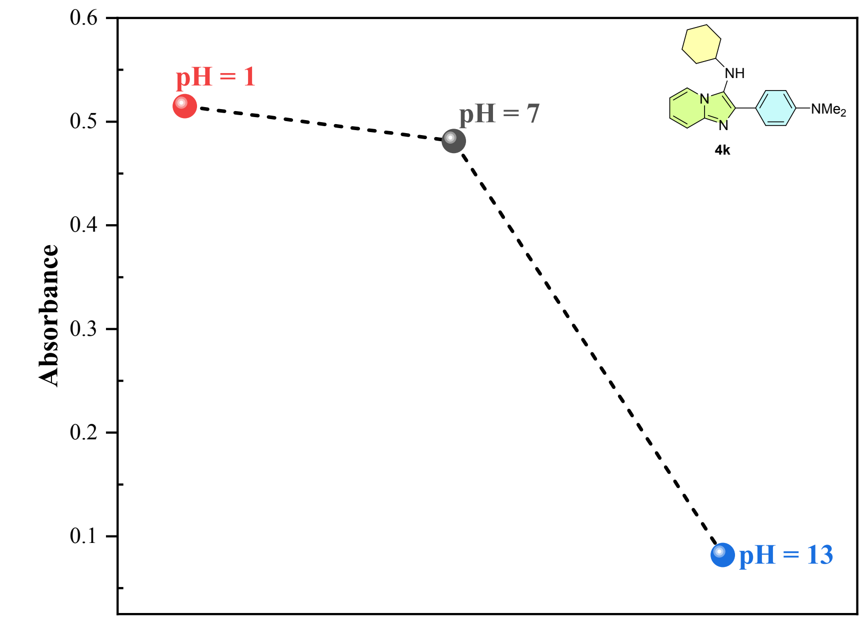

Figure S216. Plot showing different pH values as a function of absorbance for compound **4k**.

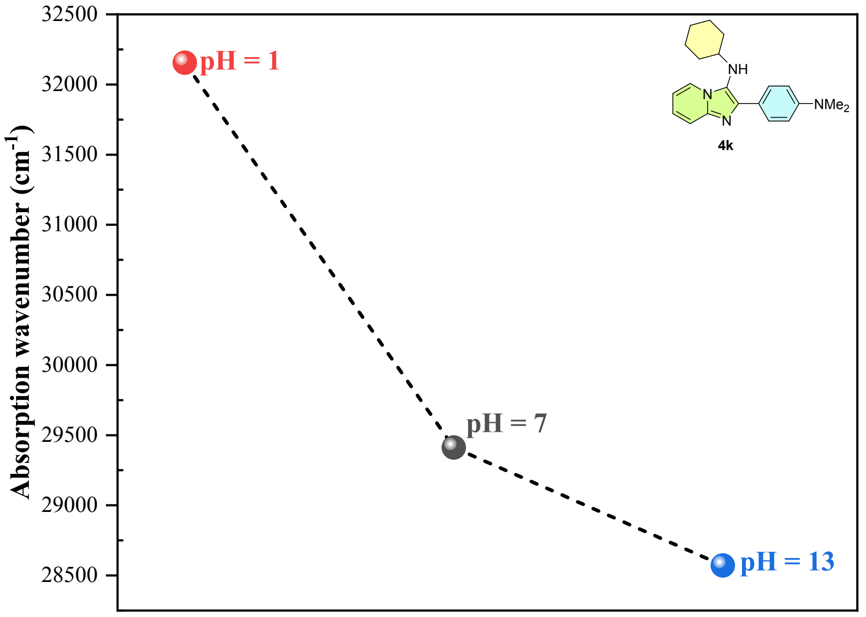

Figure S217. Plot showing different pH values as a function of absorption wavenumber (cm^{-1}) for compound **4k**.

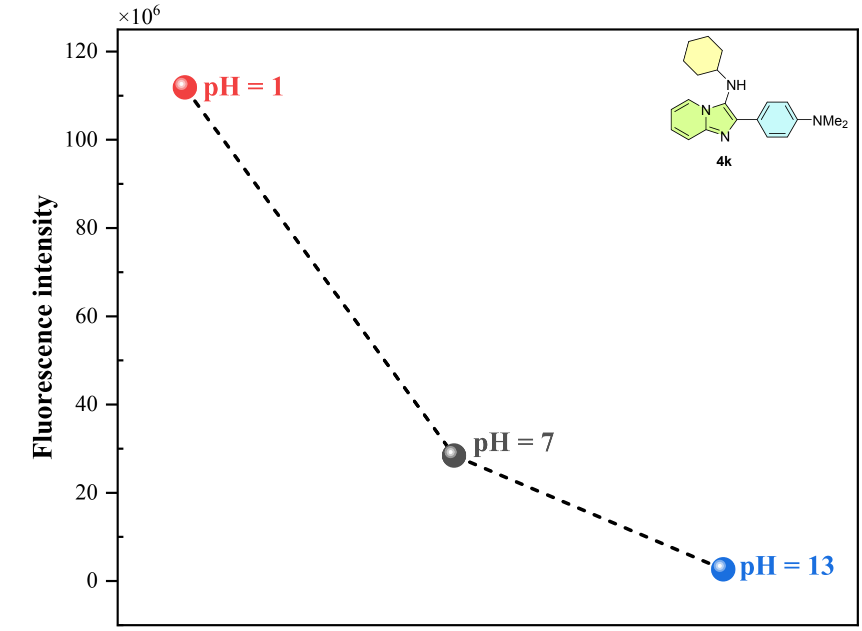

Figure S218. Plot showing different pH values as a function of fluorescence intensity for compound **4k**.

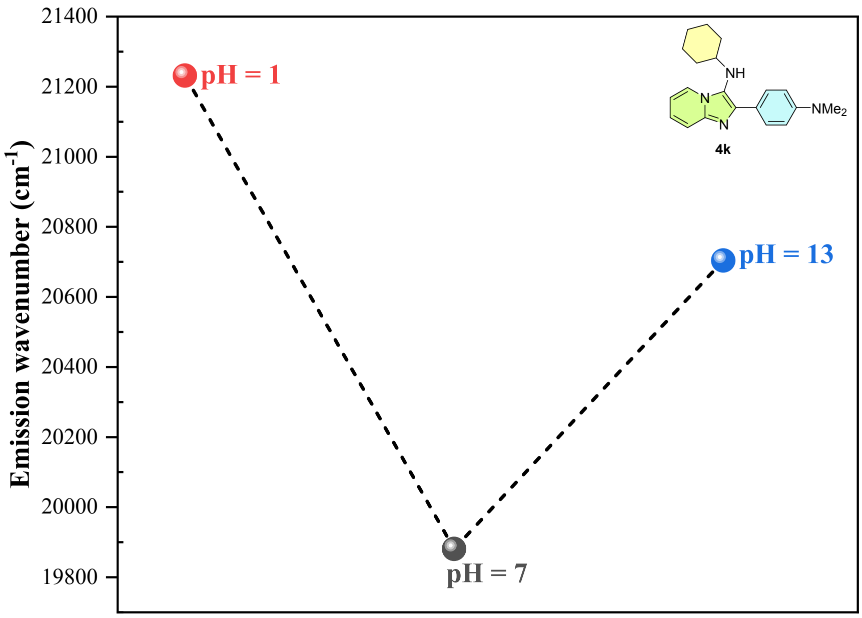

Figure S219. Plot showing different pH values as a function of emission wavenumber (cm^{-1}) for compound **4k**.

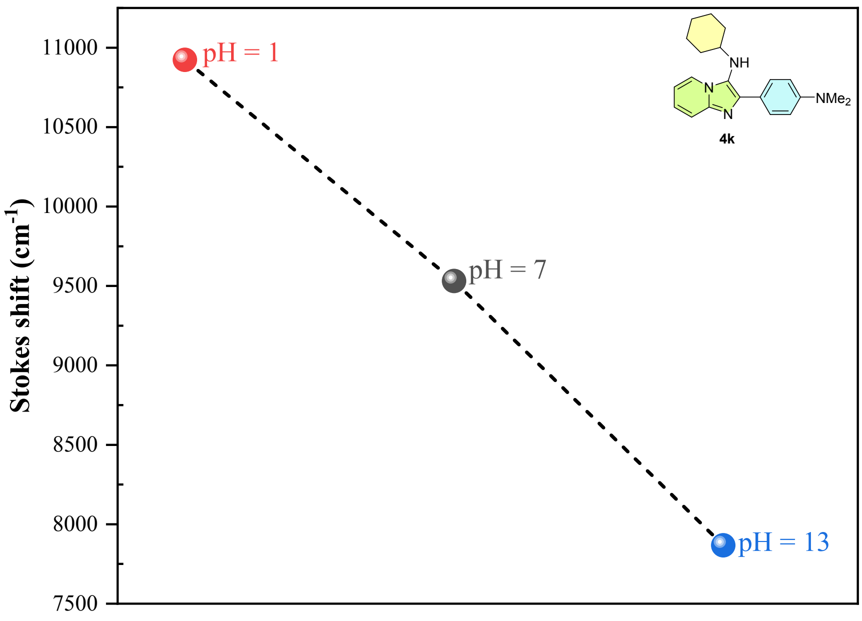

Figure S220. Plot showing different pH values as a function of Stokes shift (cm^{-1}) for compound **4k**.

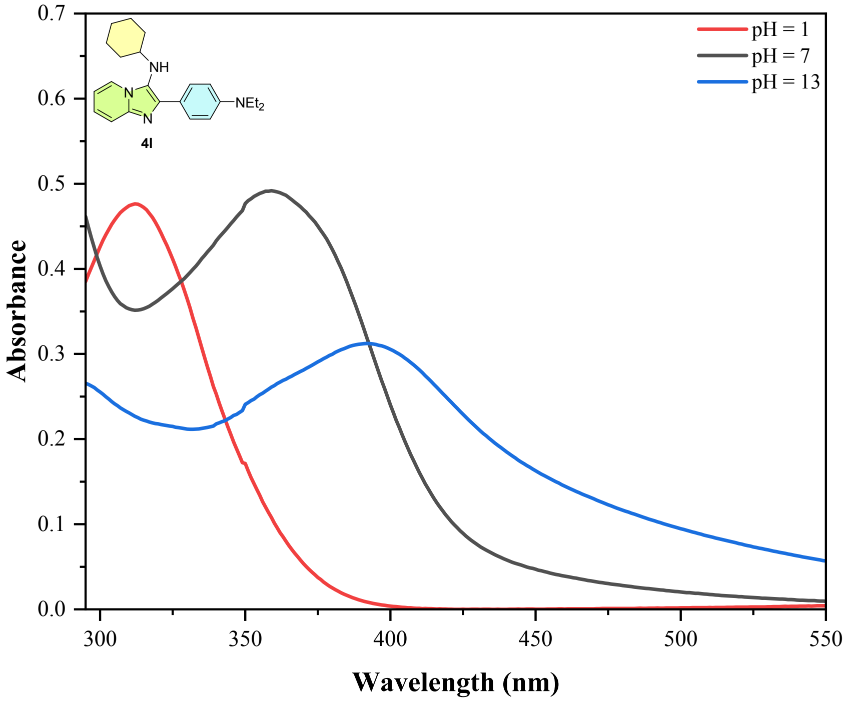

Figure S221. UV-Vis absorption spectra of **4l** in aqueous solution under different pH values ($5{\cdot}10^{-5}$ M) at room temperature.

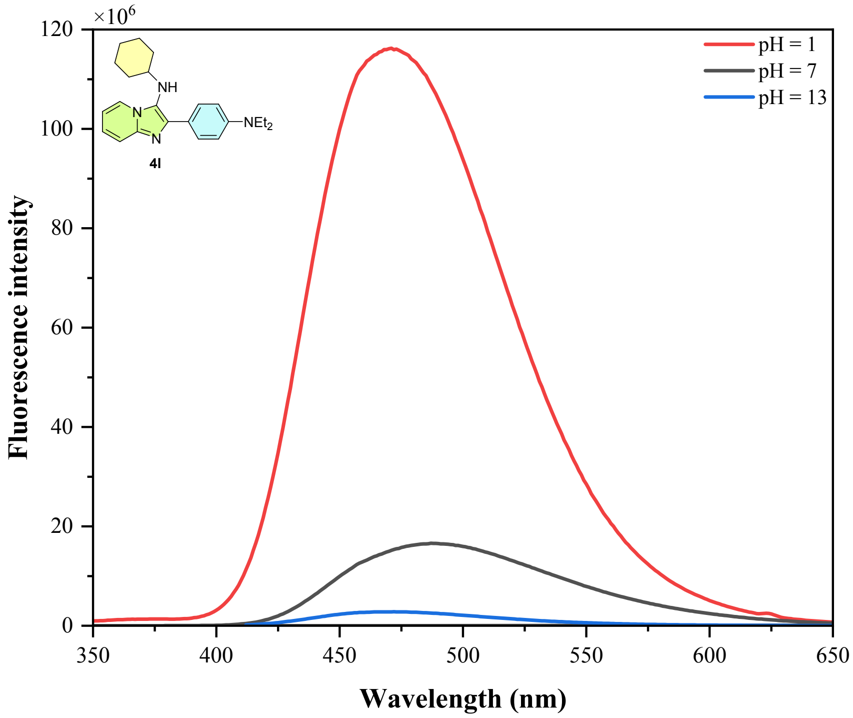

Figure S222. Normalized emission spectra of **4l** in aqueous solution under different pH values (5·10^{-5} M) at room temperature.

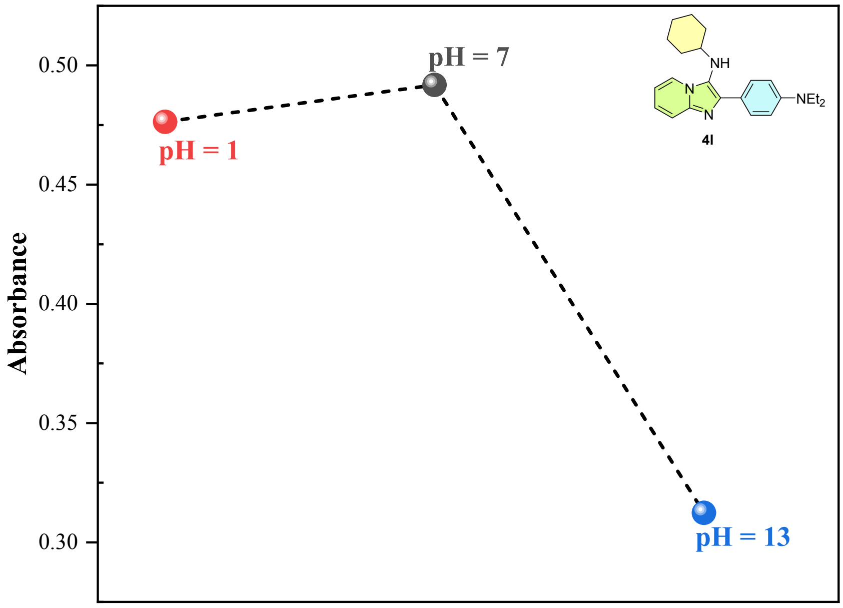

Figure S223. Plot showing different pH values as a function of absorbance for compound **4l**.

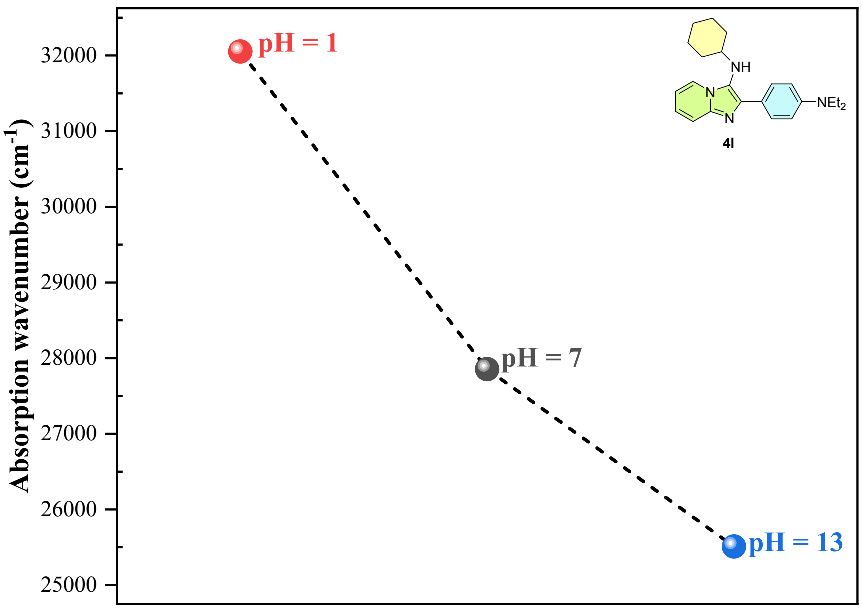

Figure S224. Plot showing different pH values as a function of absorption wavenumber (cm^{-1}) for compound **4l**.

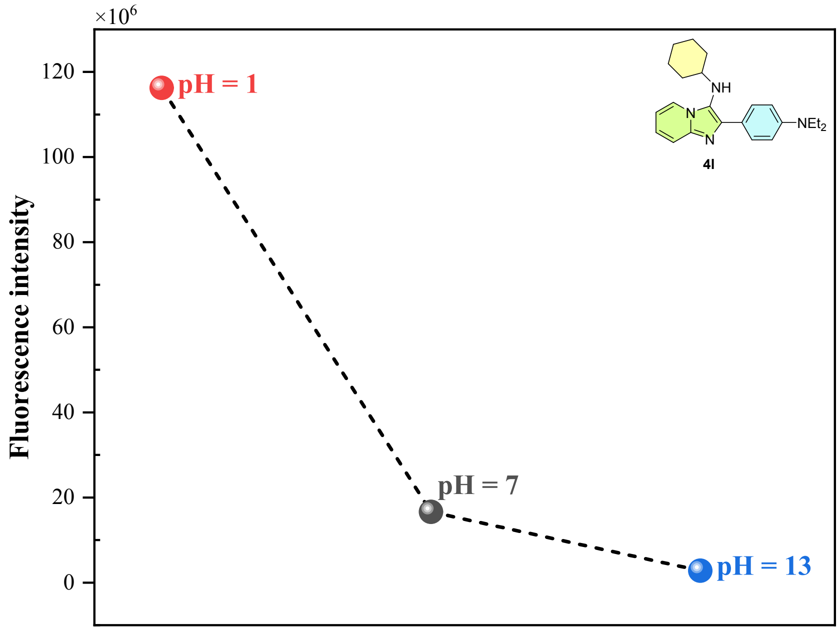

Figure S225. Plot showing different pH values as a function of fluorescence intensity for compound **4l**.

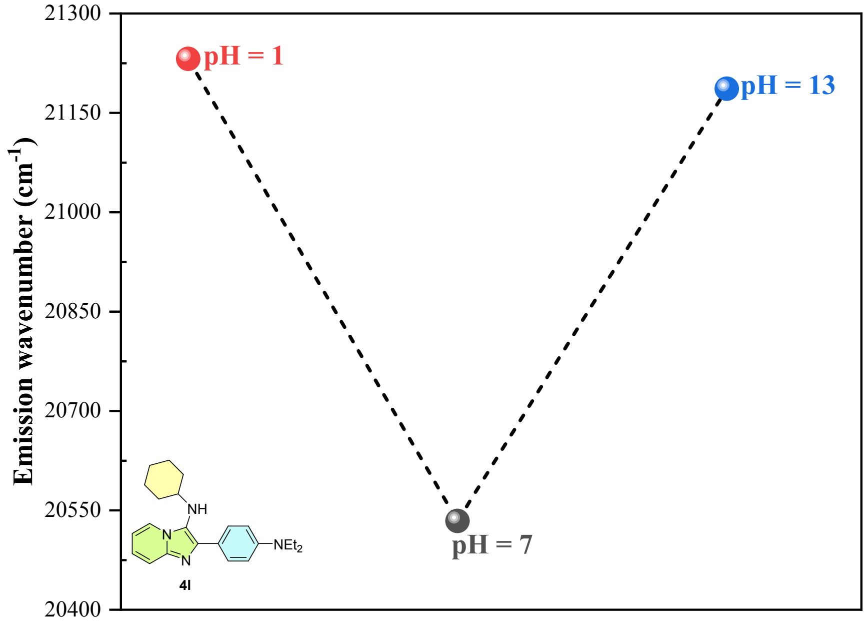

Figure S226. Plot showing different pH values as a function of emission wavenumber (cm^{-1}) for compound **4l**.

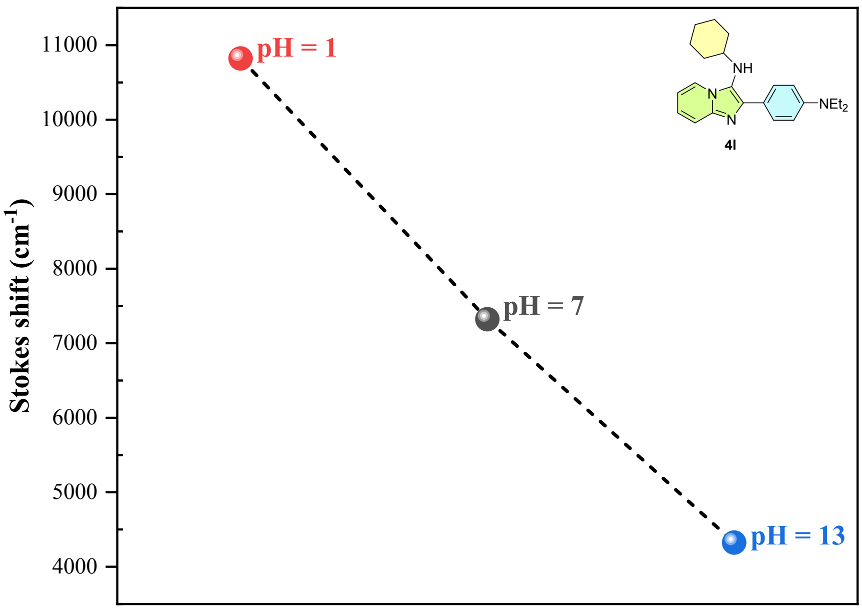

Figure S227. Plot showing different pH values as a function of Stokes shift (cm^{-1}) for compound **4l**.

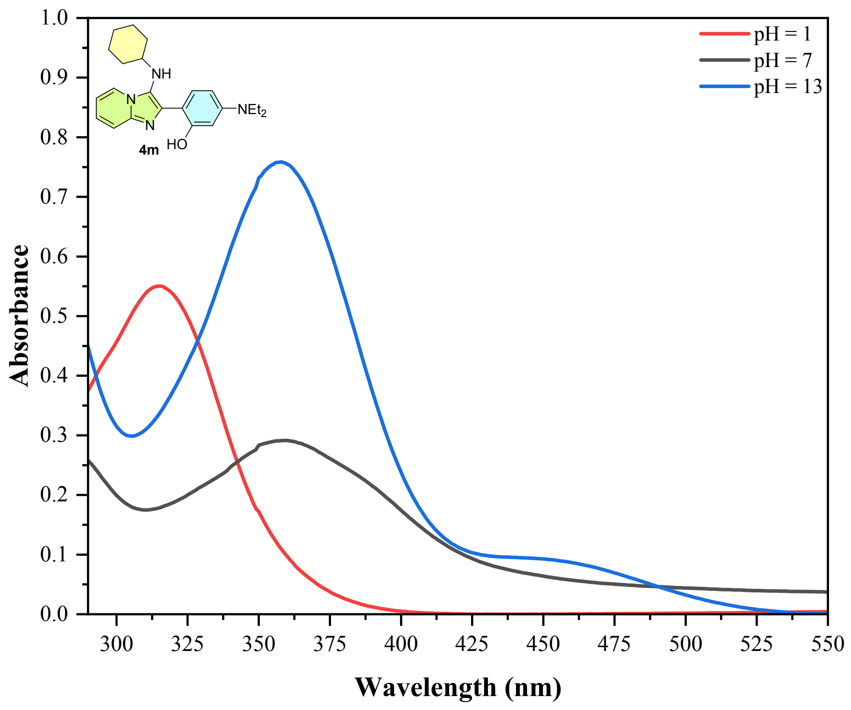

Figure S228. UV-Vis absorption spectra of **4m** in aqueous solution under different pH values (5·10^{-5} M) at room temperature.

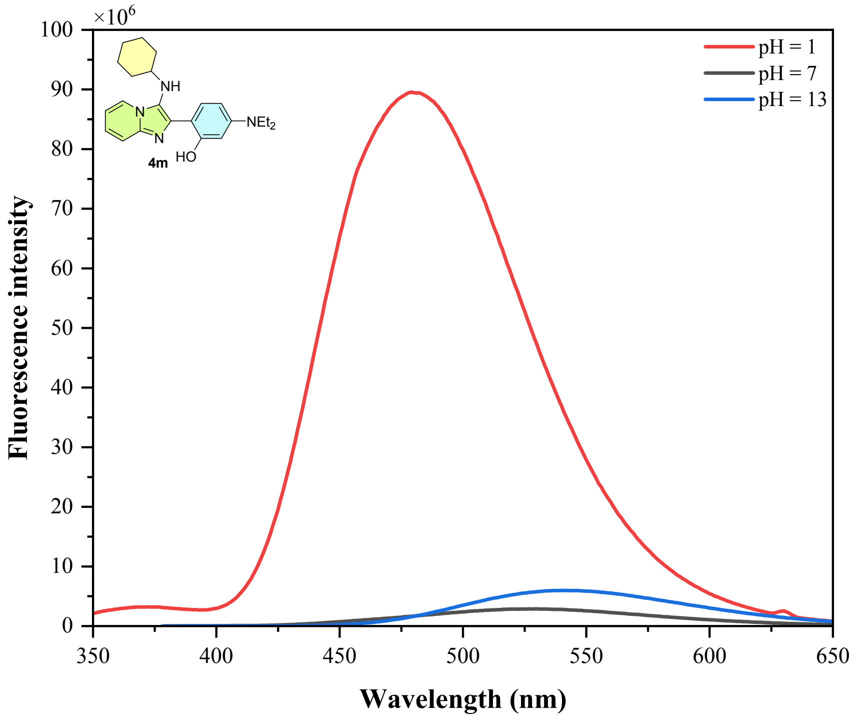

Figure S229. Normalized emission spectra of **4m** in aqueous solution under different pH values (5·10^{-5} M) at room temperature.

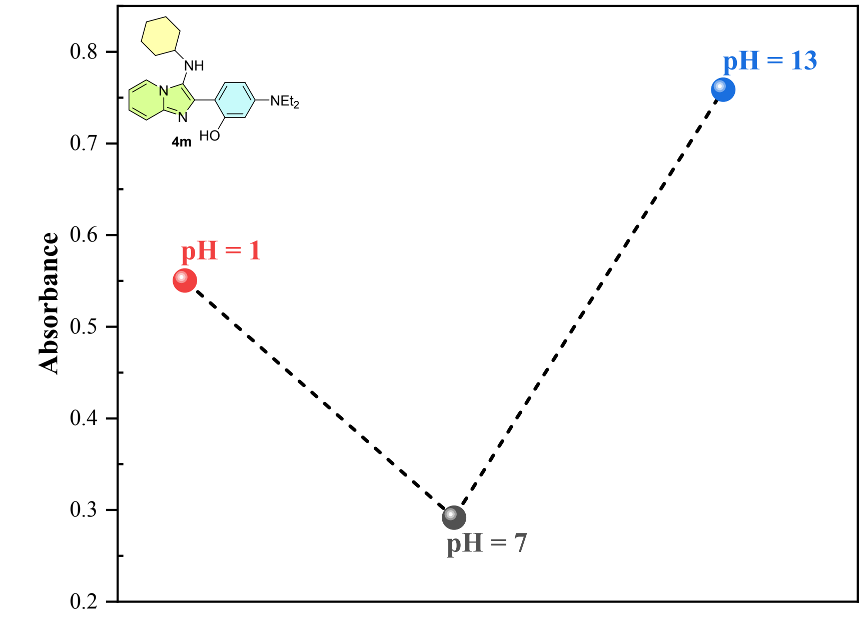

Figure S230. Plot showing different pH values as a function of absorbance for compound **4m**.

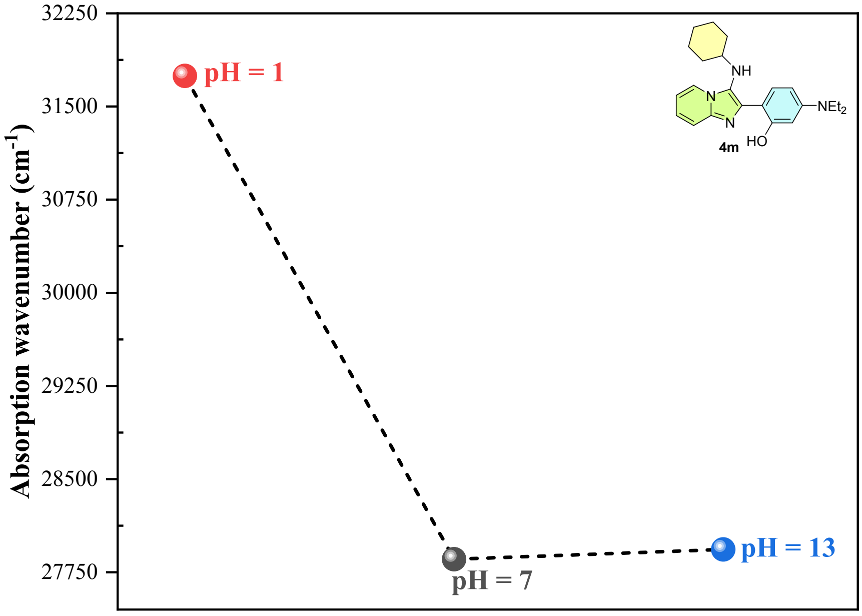

Figure S231. Plot showing different pH values as a function of absorption wavenumber (cm^{-1}) for compound **4m**.

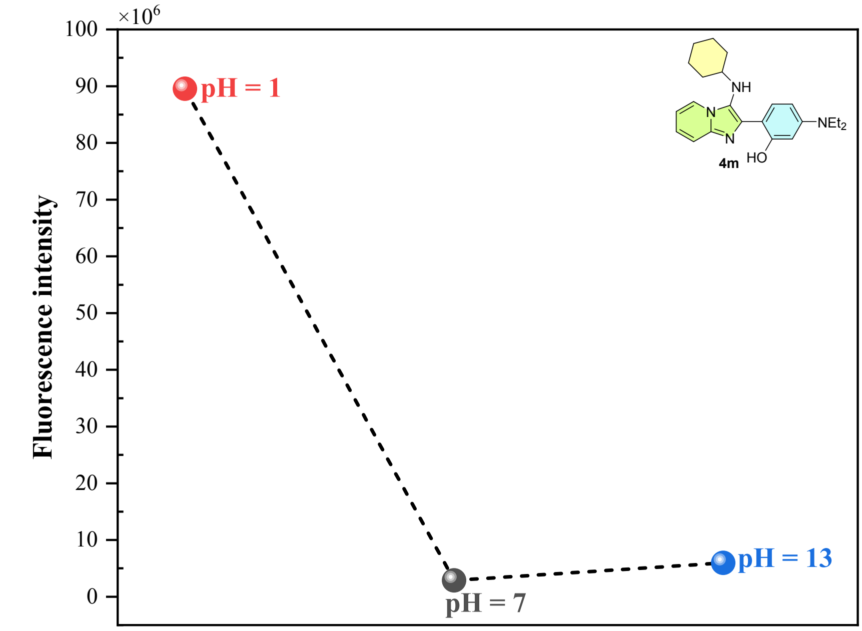

Figure S232. Plot showing different pH values as a function of fluorescence intensity for compound **4m**.

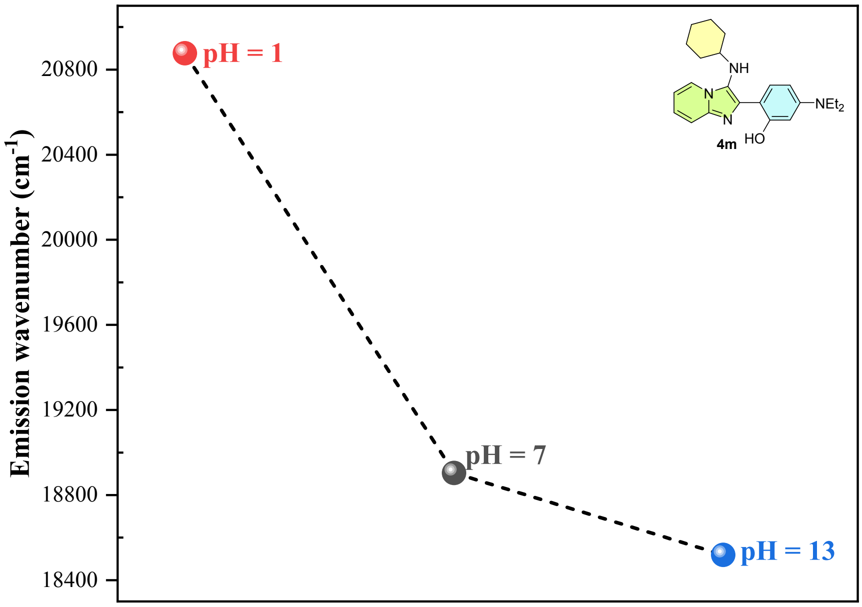

Figure S233. Plot showing different pH values as a function of emission wavenumber (cm^{-1}) for compound **4m**.

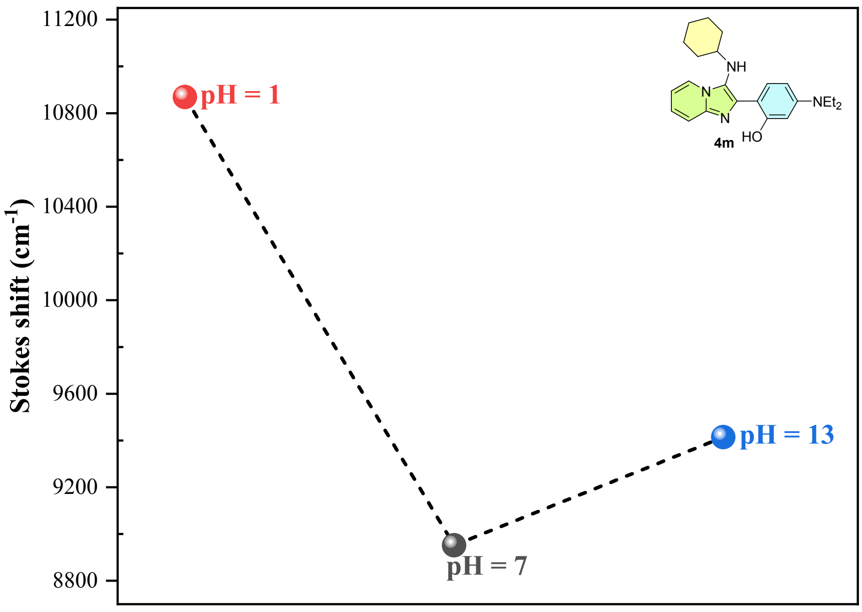

Figure S234. Plot showing different pH values as a function of Stokes shift (cm^{-1}) for compound **4m**.

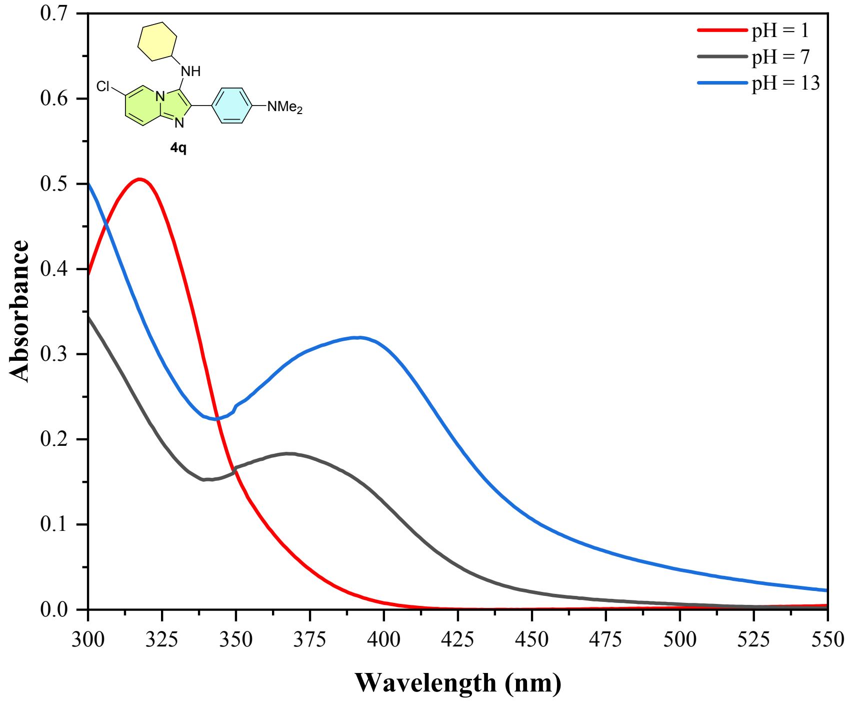

Figure S235. UV-Vis absorption spectra of **4q** in aqueous solution under different pH values ($5 \cdot 10^{-5}$ M) at room temperature.

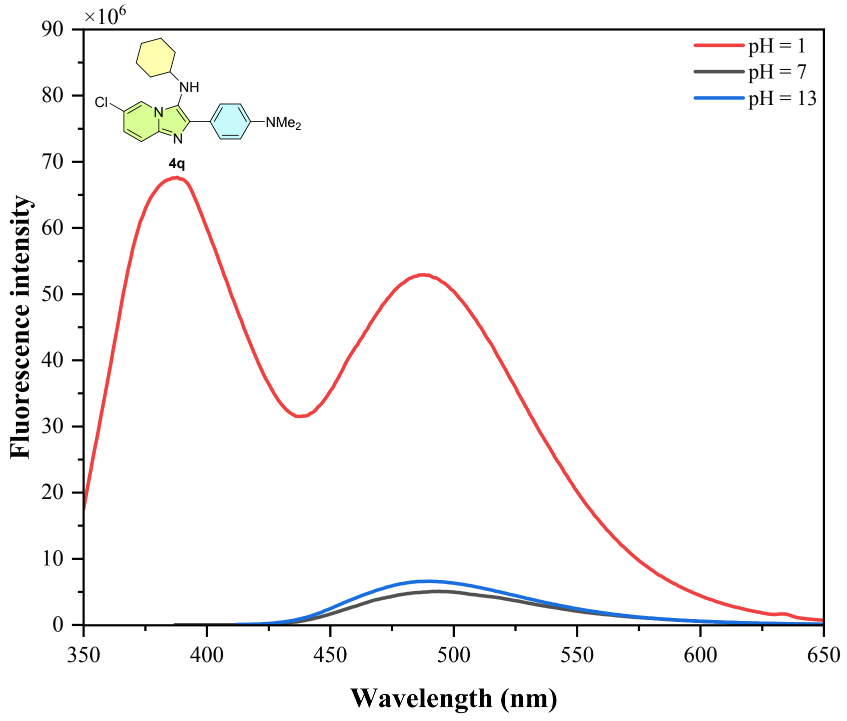

Figure S236. Normalized emission spectra of **4q** in aqueous solution under different pH values (5·10^{-5} M) at room temperature.

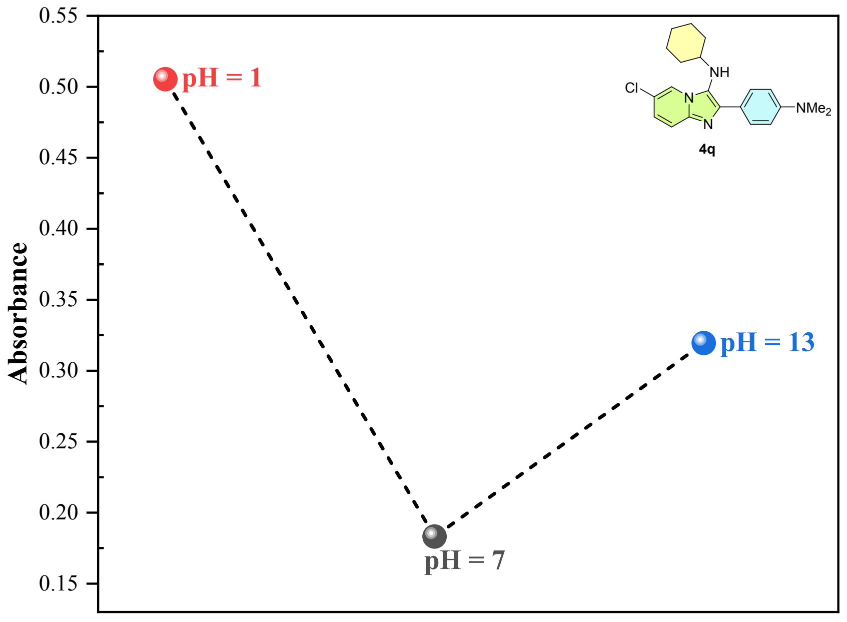

Figure S237. Plot showing different pH values as a function of absorbance for compound **4q**.

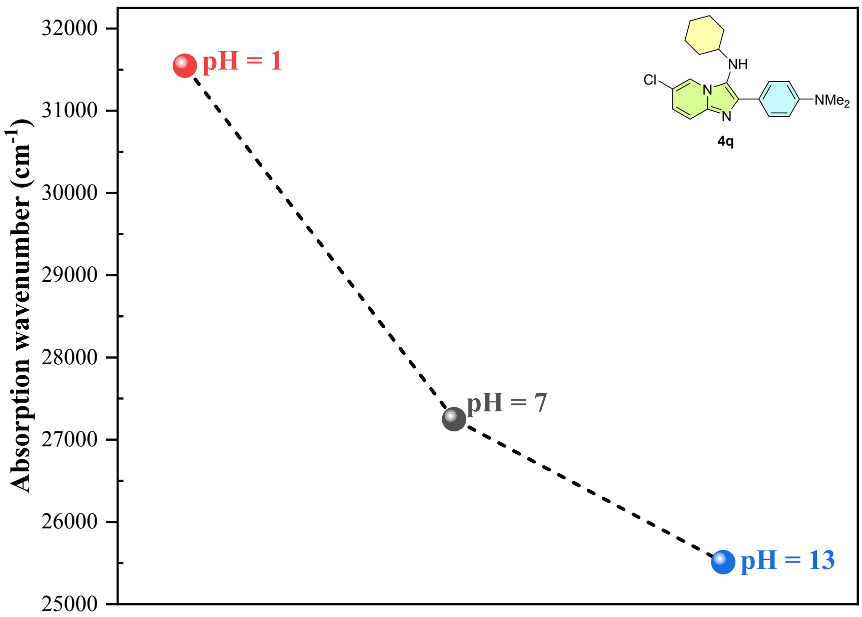

Figure S238. Plot showing different pH values as a function of absorption wavenumber (cm^{-1}) for compound **4q**.

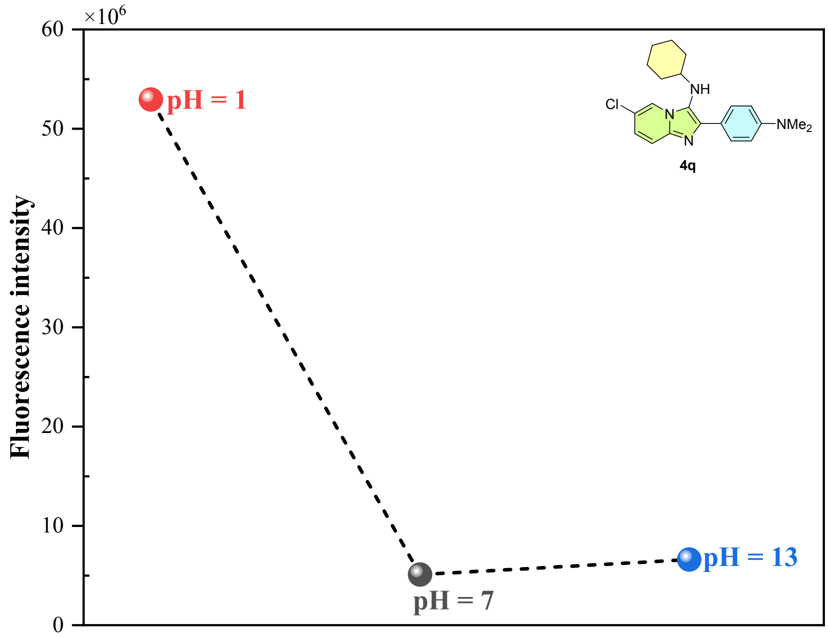

Figure S239. Plot showing different pH values as a function of fluorescence intensity for compound **4q**.

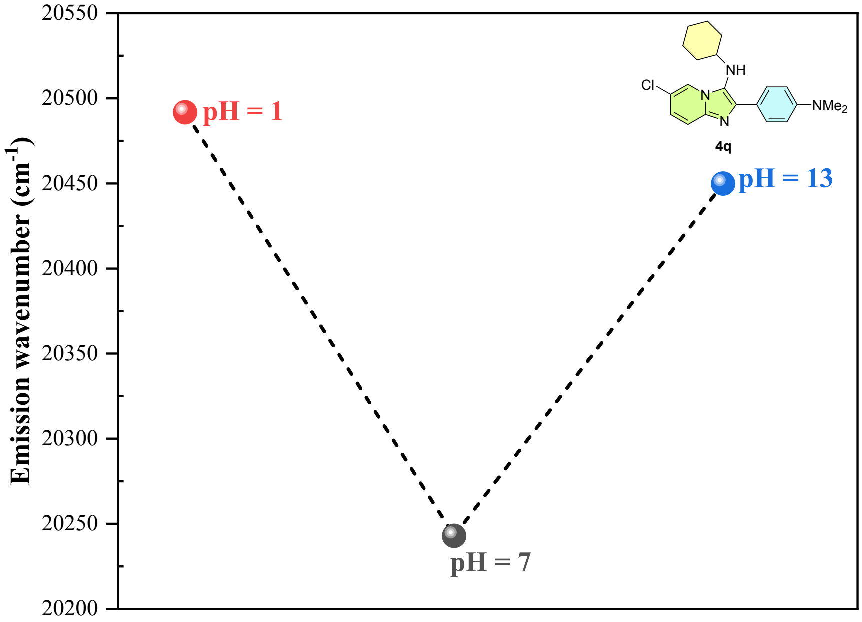

Figure S240. Plot showing different pH values as a function of emission wavenumber (cm^{-1}) for compound **4q**.

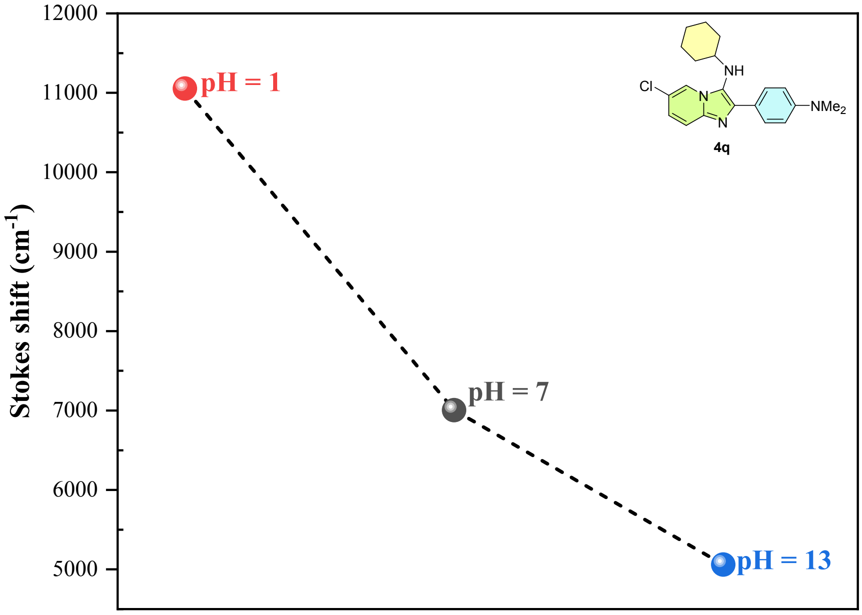

Figure S241. Plot showing different pH values as a function of Stokes shift (cm^{-1}) for compound **4q**.

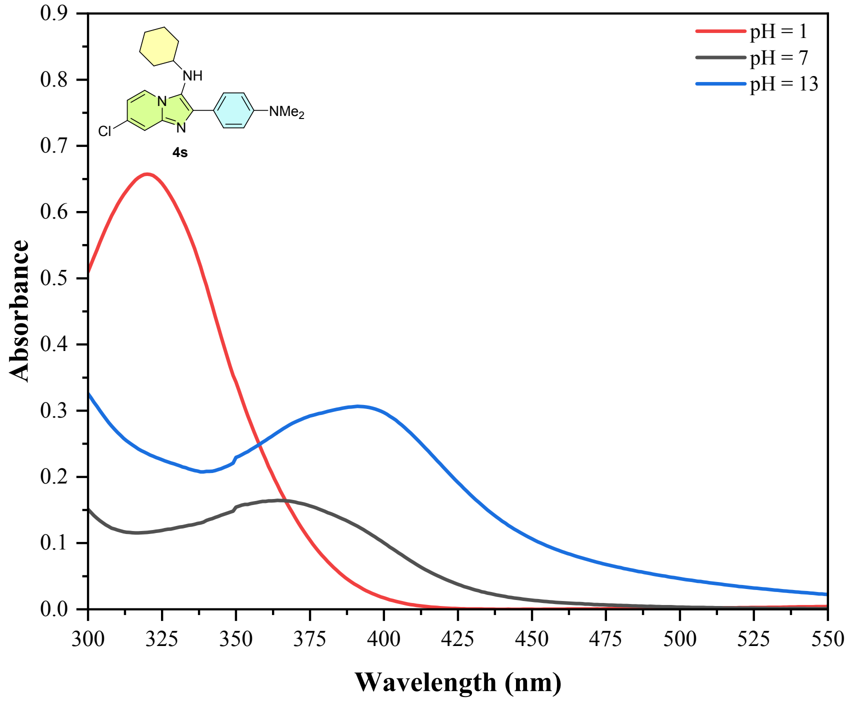

Figure S242. UV-Vis absorption spectra of **4s** in aqueous solution under different pH values ($5{\cdot}10^{-5}$ M) at room temperature.

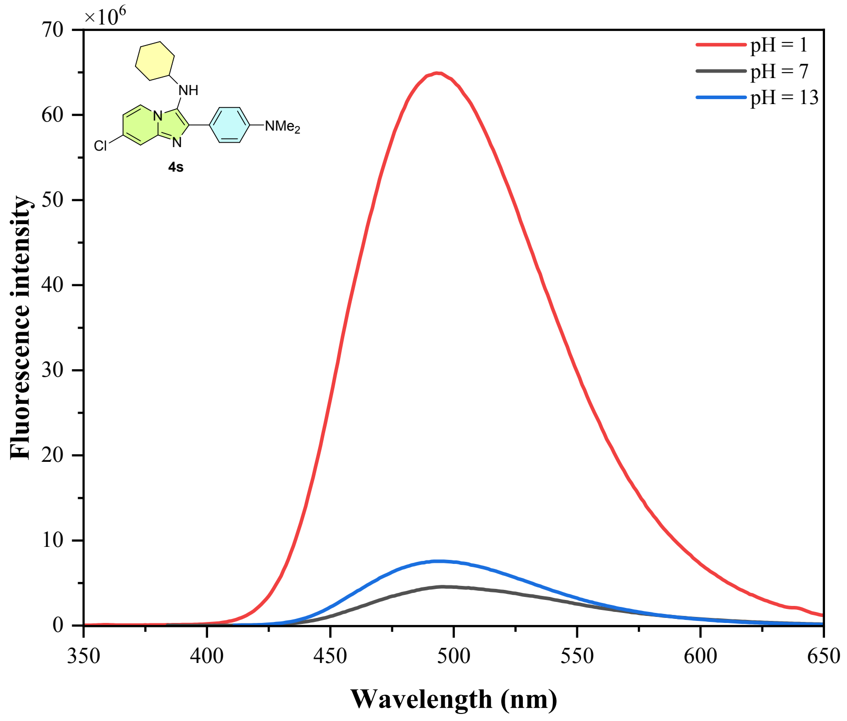

Figure S243. Normalized emission spectra of **4s** in aqueous solution under different pH values ($5{\cdot}10^{-5}$ M) at room temperature.

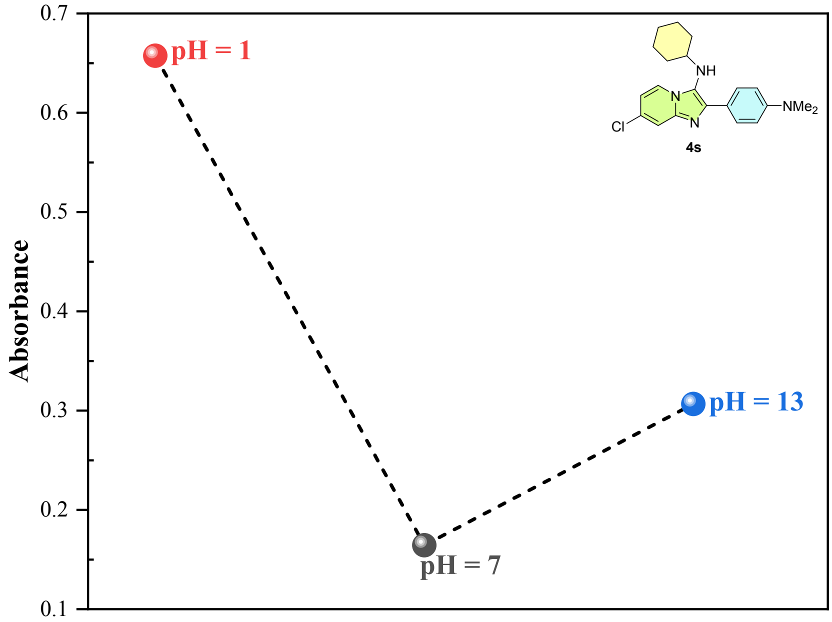

Figure S244. Plot showing different pH values as a function of absorbance for compound **4s**.

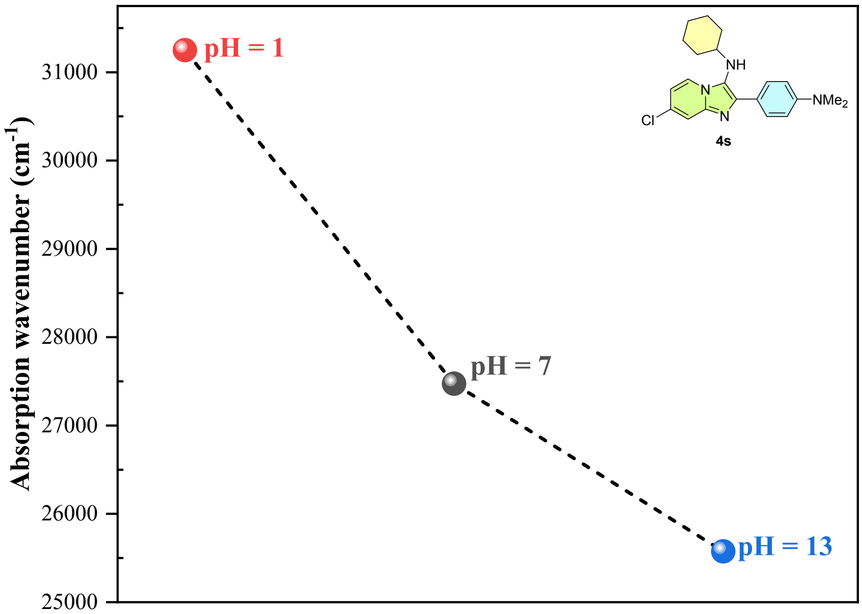

Figure S245. Plot showing different pH values as a function of absorption wavenumber (cm^{-1}) for compound **4s**.

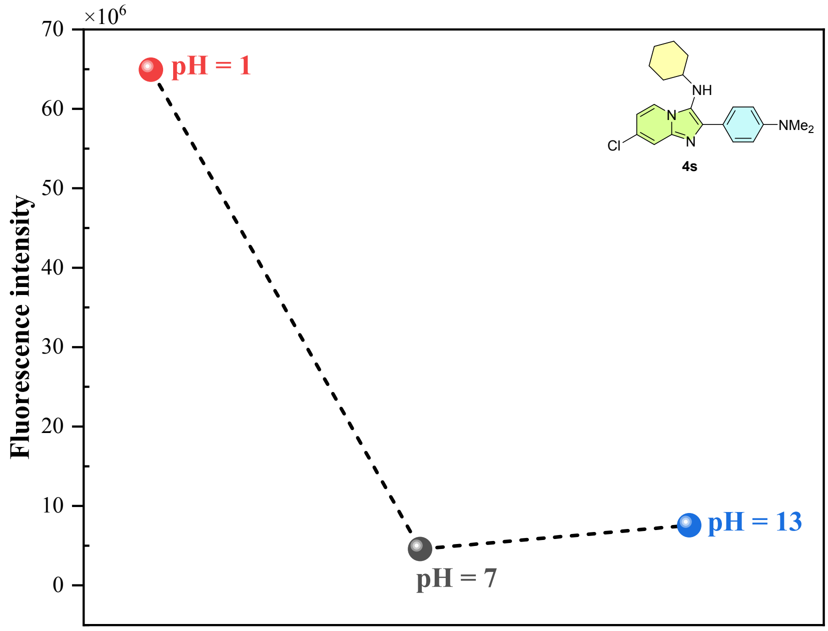

Figure S246. Plot showing different pH values as a function of fluorescence intensity for compound **4s**.

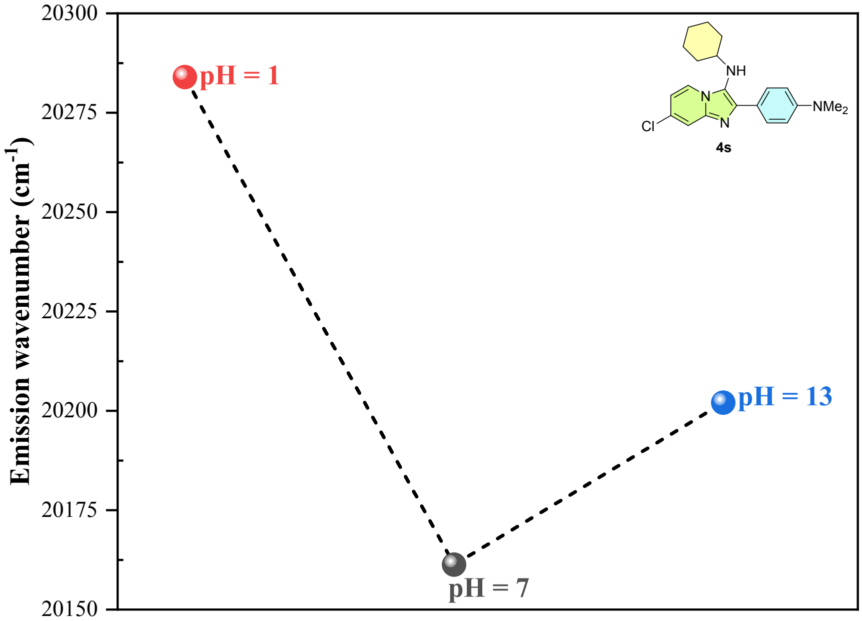

Figure S247. Plot showing different pH values as a function of emission wavenumber (cm^{-1}) for compound **4s**.

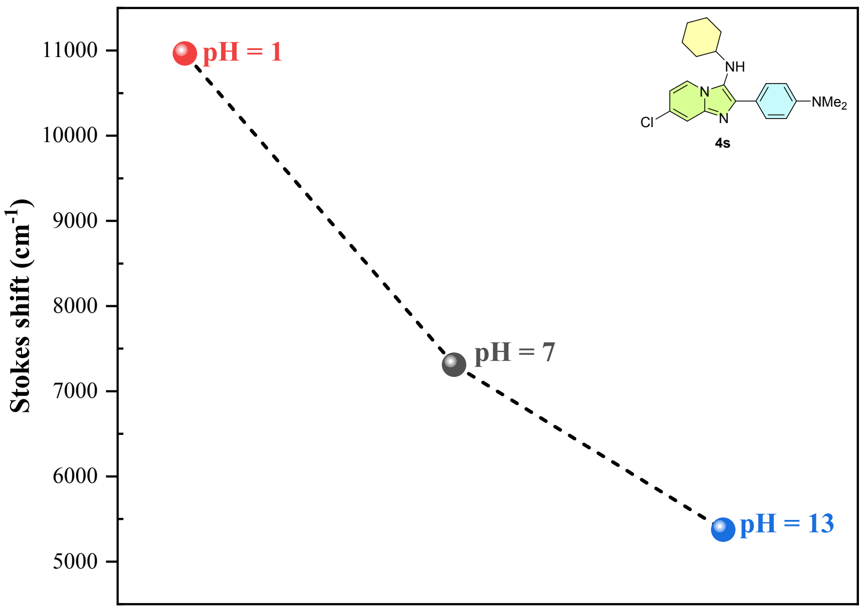

Figure S248. Plot showing different pH values as a function of Stokes shift (cm^{-1}) for compound **4s**.

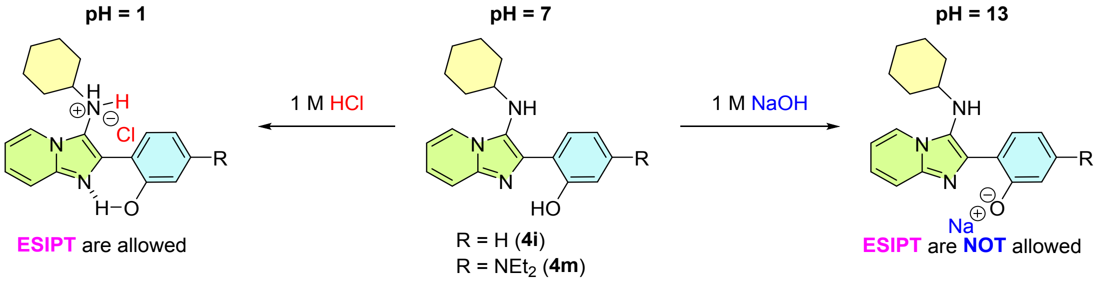

Scheme S3. Proposals of structures present at different pH values for compounds **4i** and **4m**.

5.2 Aqueous glycerol solution study

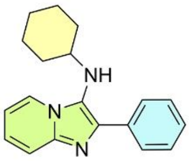

4a

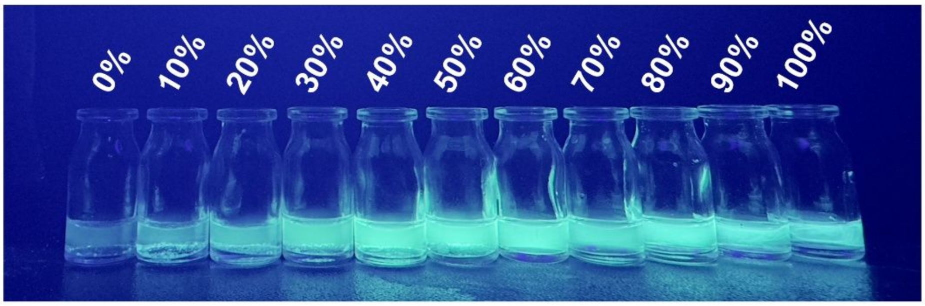

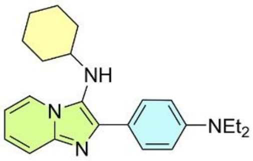

4l

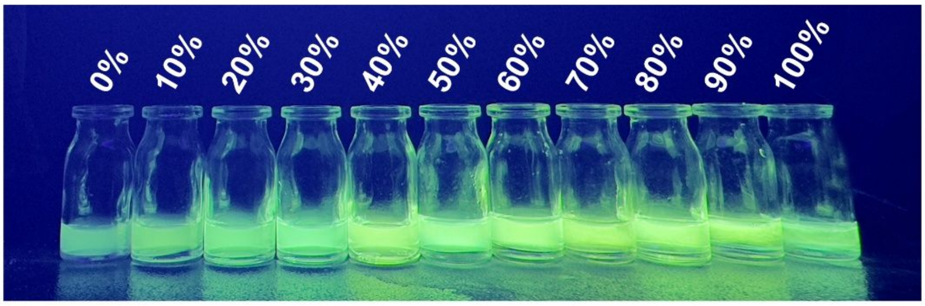

Figure S249. Emission of compounds **4a** and **4l** in glycerol/water increasing the glycerol content ($5{\cdot}10^{-5}$ M) at room temperature; λ_{exc} = 365 nm. in a dark chamber with an UV lamp.

Table S6. Photophysical properties of compounds **4a** and **4l** in glycerol/water increasing the glycerol content.[a]

Comp.	% Glycerol (*v*/*v*)	λ_{abs}, nm[b]	λ_{em}, nm[c]	log ε (ε, $M^{-1}{\cdot}cm^{-1}$)[d]	Stokes Shift, cm^{-1}[e]
**4a**	0	326	488	3.64 (4397)	10183
	10	330	487	3.26 (1819)	9769
	20	331	488	3.22 (1659)	9720
	30	331	487	3.61 (4074)	9678
	40	331	486	3.58 (3846)	9635
	50	332	486	3.63 (4290)	9544
	60	332	482	3.60 (3977)	9374
	70	332	480	3.37 (2351)	9287
	80	333	483	3.55 (3588)	9326
	90	331	484	3.65 (4451)	9550
	100	335	401	3.51 (3259)	4913
**4l**	0	359	487	3.99 (9837)	7321
	10	353	499	3.66 (4541)	8289
	20	350	501	3.65 (4494)	8611

	30	352	502	3.76 (5731)	8489
	40	353	502	3.97 (9328)	8408
	50	353	503	4.02 (10469)	8448
	60	357	506	3.98 (9637)	8248
	70	356	513	3.95 (8994)	8597
	80	357	522	3.63 (4247)	8854
	90	361	501	3.47 (2924)	7741
	100	349	416	3.09 (1236)	4615

[a] Carried out at room temperature ($5{\cdot}10^{-5}$ M). [b] λ_{abs} = absorption maxima (nm). [c] λ_{em} = emission maxima (nm). [d] ε = molar absorptivity ($M^{-1}{\cdot}cm^{-1}$). [e] Stokes shifts difference between λ_{em} and λ_{abs}.

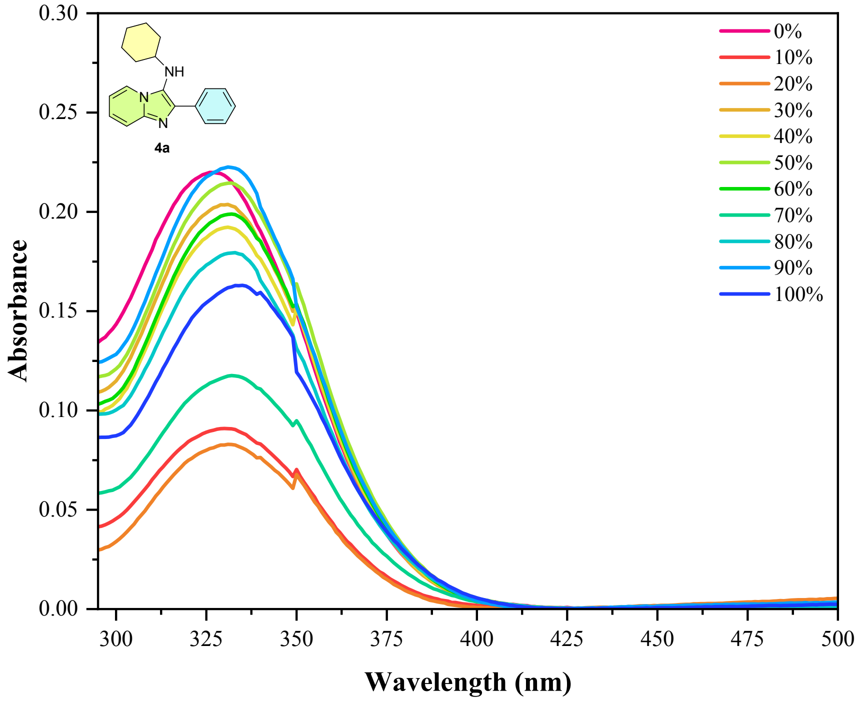

Figure S250. UV-Vis absorption spectra of **4a** in glycerol/water increasing the glycerol content ($5{\cdot}10^{-5}$ M) at room temperature.

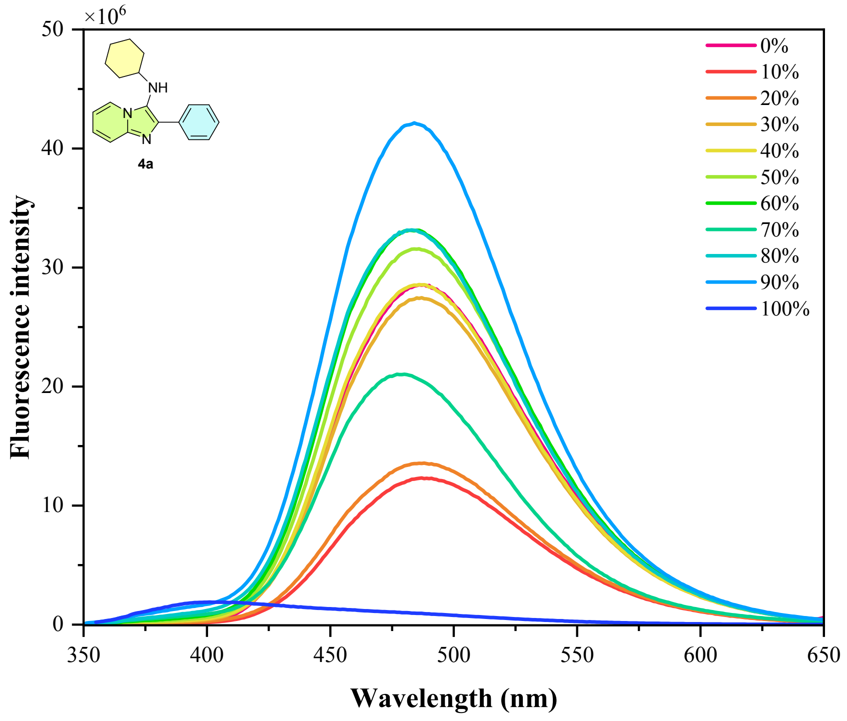

Figure S251. Normalized emission spectra of **4a** in glycerol/water increasing the glycerol content ($5 \cdot 10^{-5}$ M) at room temperature.

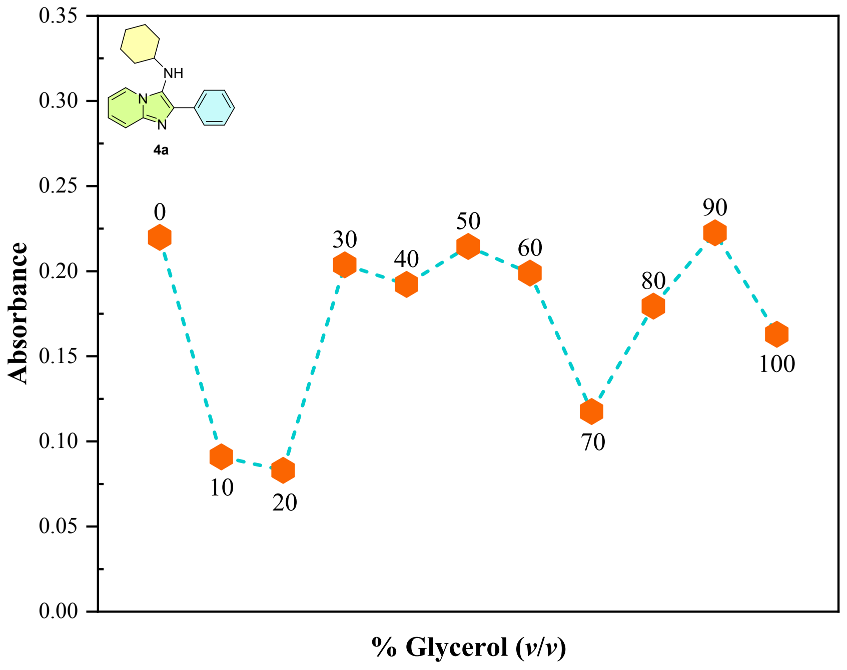

Figure S252. Plot showing glycerol/water increasing the glycerol content values as a function of absorbance for compound **4a**.

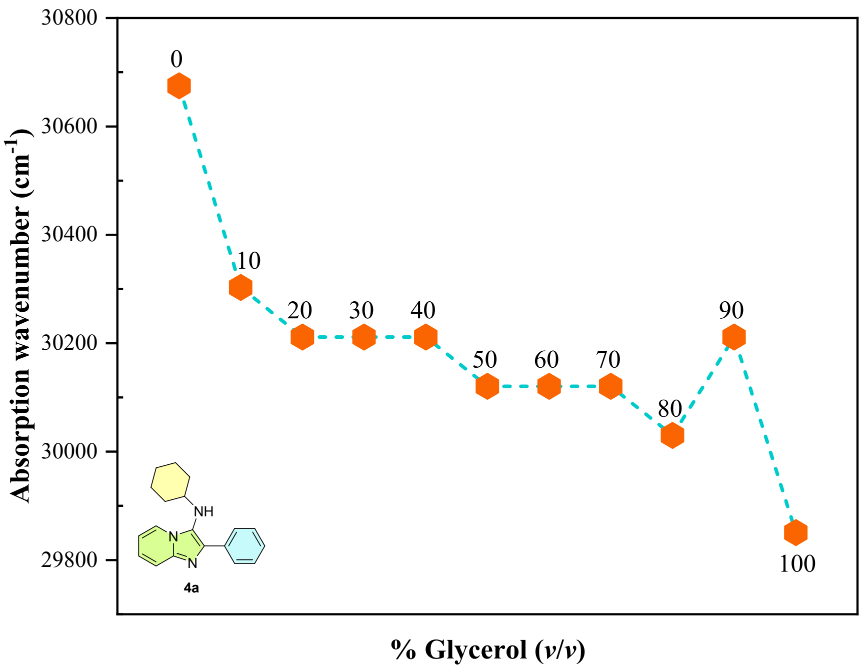

Figure S253. Plot showing glycerol/water increasing the glycerol content as a function of absorption wavenumber (cm^{-1}) for compound **4a**.

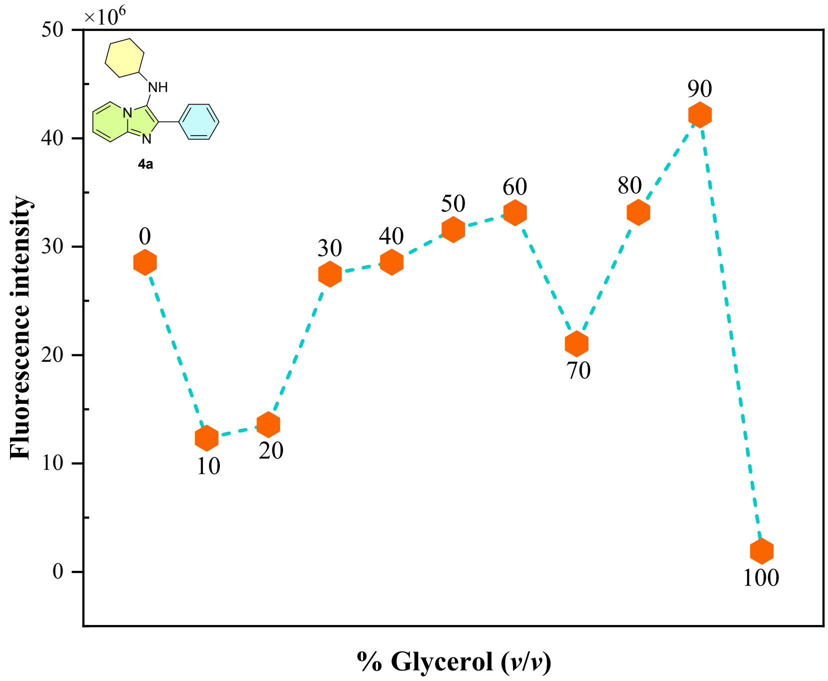

Figure S254. Plot showing glycerol/water increasing the glycerol content as a function of fluorescence intensity for compound **4a**.

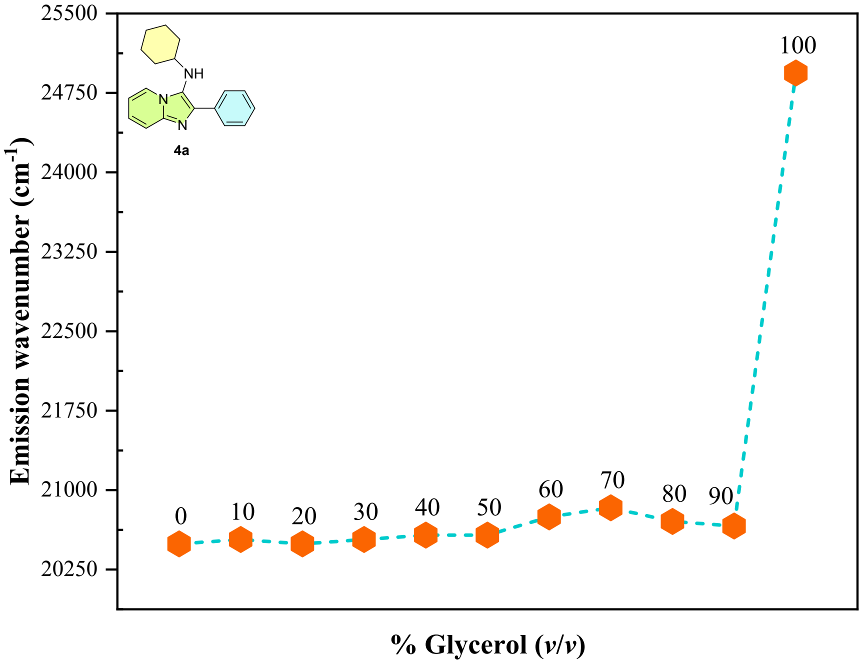

Figure S255. Plot showing glycerol/water increasing the glycerol content as a function of emission wavenumber (cm^{-1}) for compound **4a**.

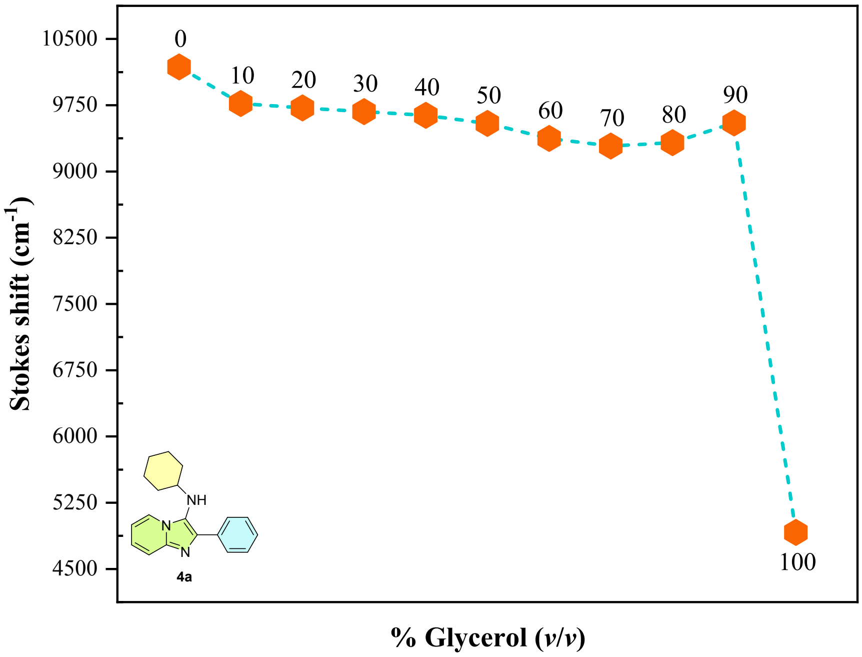

Figure S256. Plot showing glycerol/water increasing the glycerol content as a function of Stokes shift (cm^{-1}) for compound **4a**.

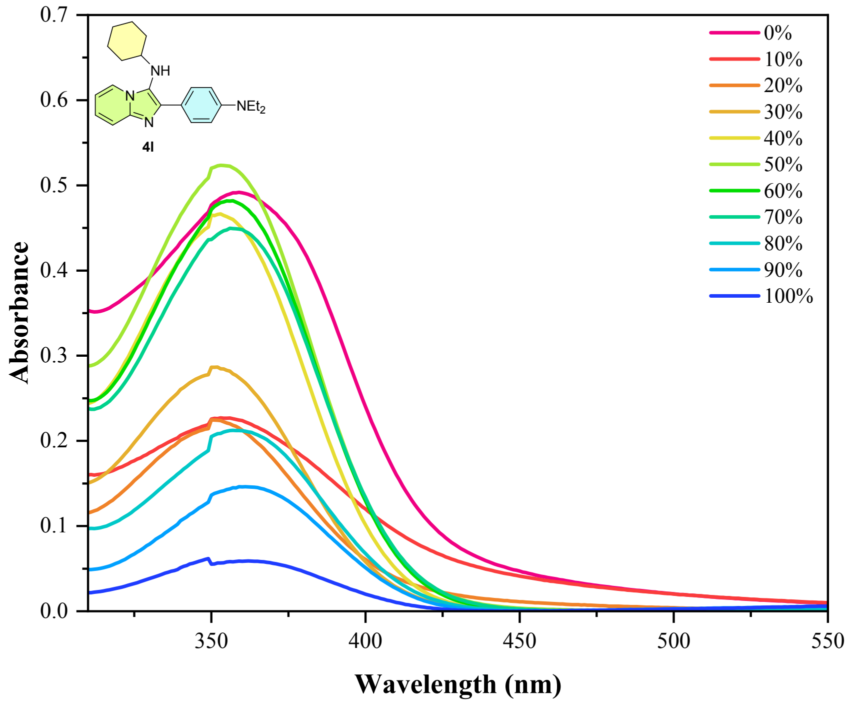

Figure S257. UV-Vis absorption spectra of **4l** in glycerol/water increasing the glycerol content ($5\cdot10^{-5}$ M) at room temperature.

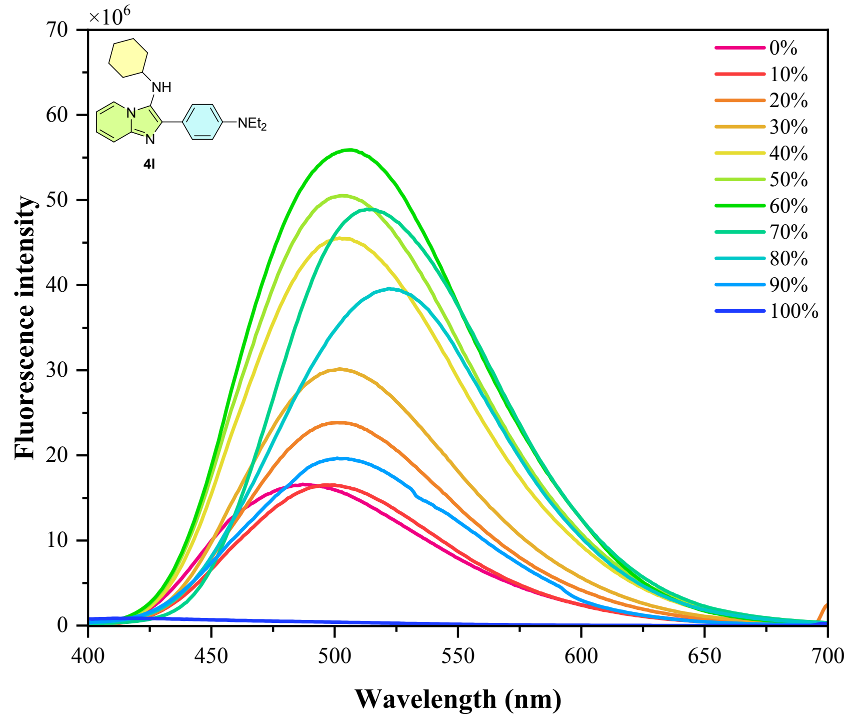

Figure S258. Normalized emission spectra of **4l** in glycerol/water increasing the glycerol content ($5\cdot10^{-5}$ M) at room temperature.

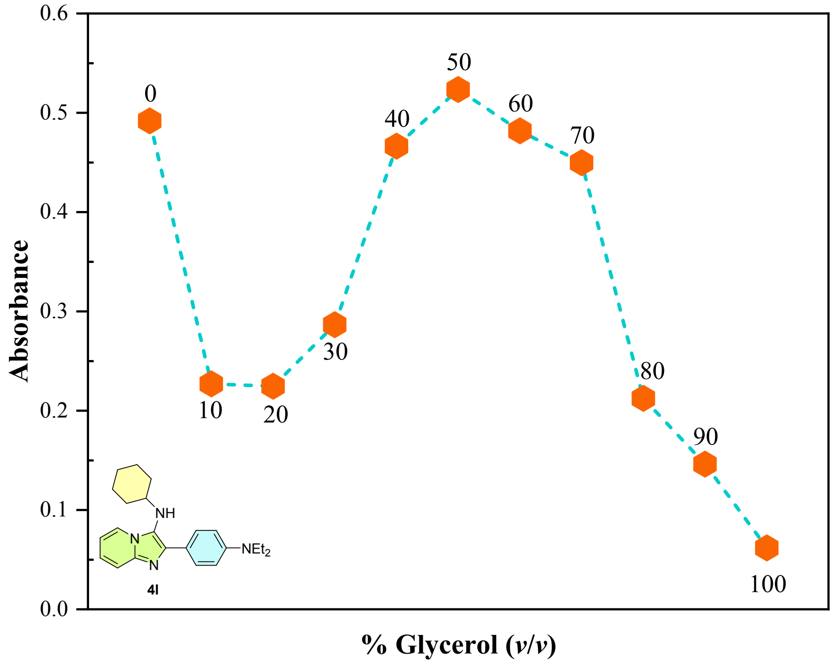

Figure S259. Plot showing glycerol/water increasing the glycerol content values as a function of absorbance for compound **4l**.

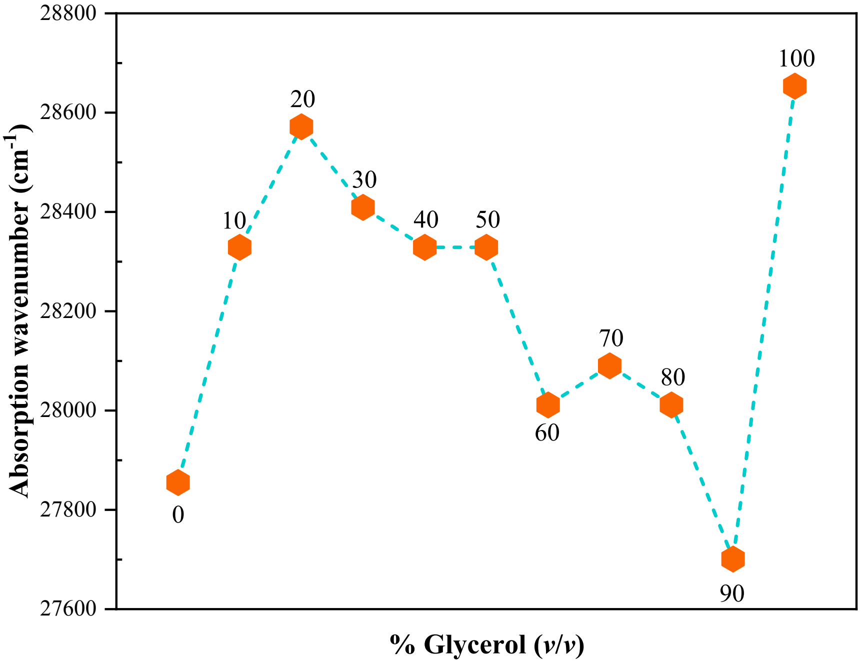

Figure S260. Plot showing glycerol/water increasing the glycerol content as a function of absorption wavenumber (cm^{-1}) for compound **4l**.

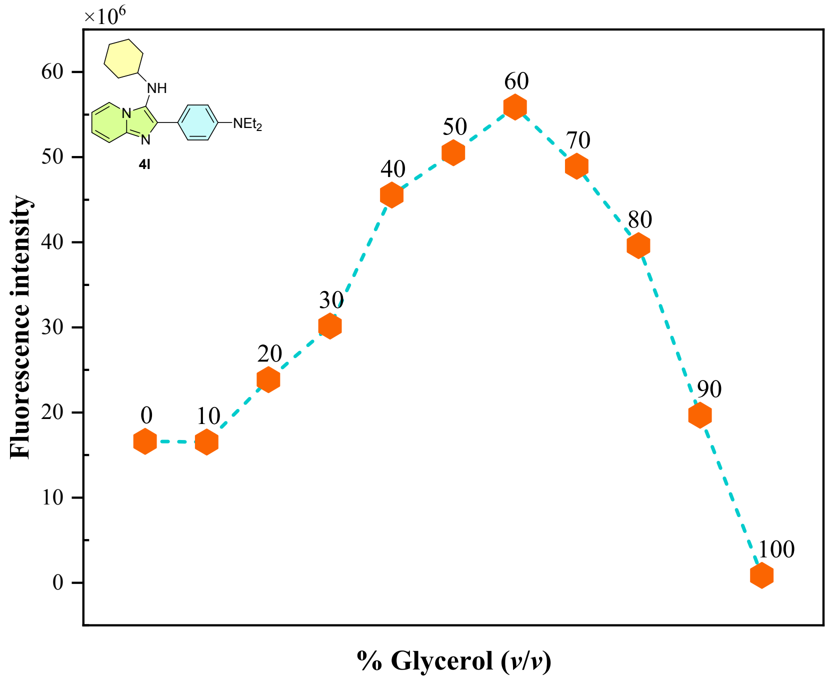

Figure S261. Plot showing glycerol/water increasing the glycerol content as a function of fluorescence intensity for compound **4l**.

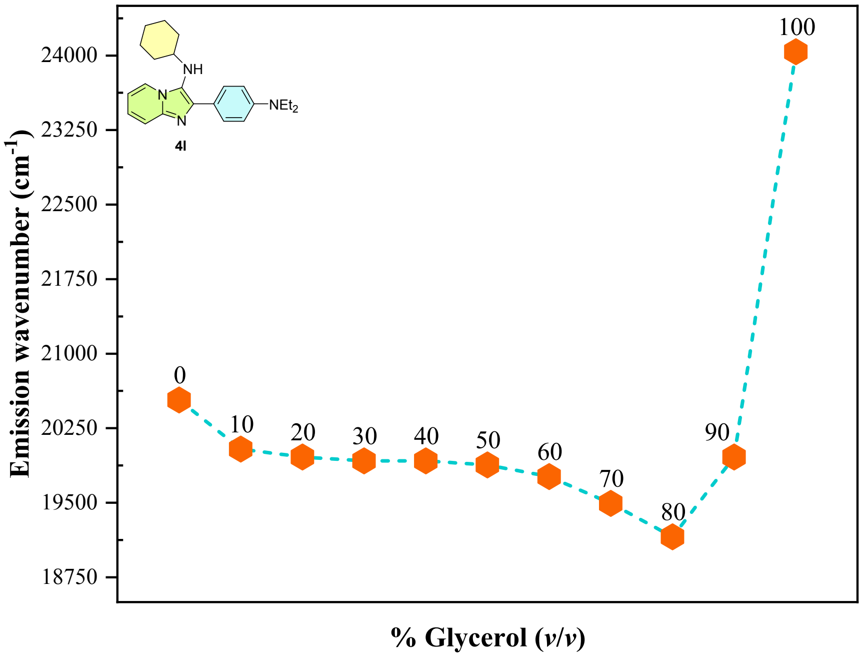

Figure S262. Plot showing glycerol/water increasing the glycerol content as a function of emission wavenumber (cm^{-1}) for compound **4l**.

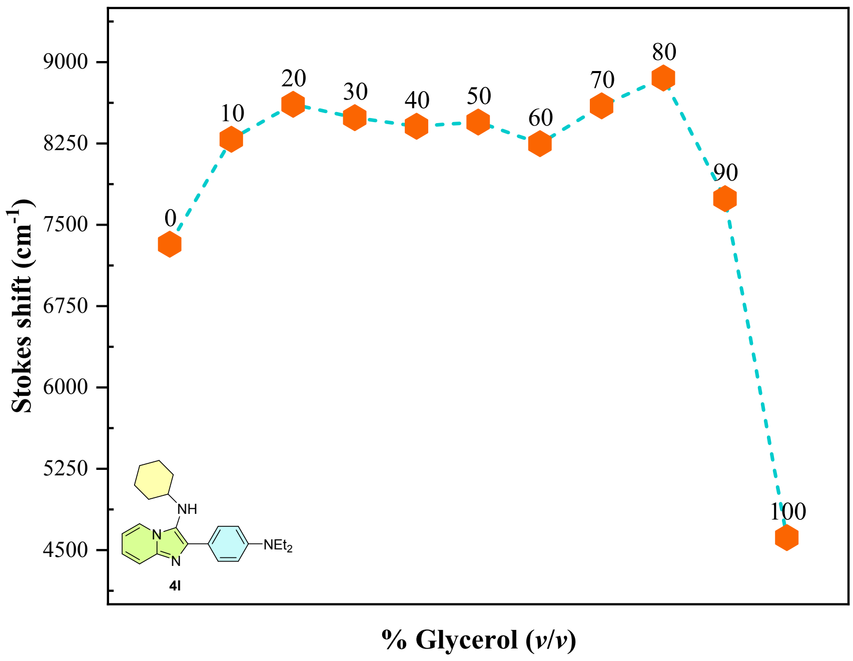

Figure S263. Plot showing glycerol/water increasing the glycerol content as a function of Stokes shift (cm^{-1}) for compound **4l**.

6. Computational studies

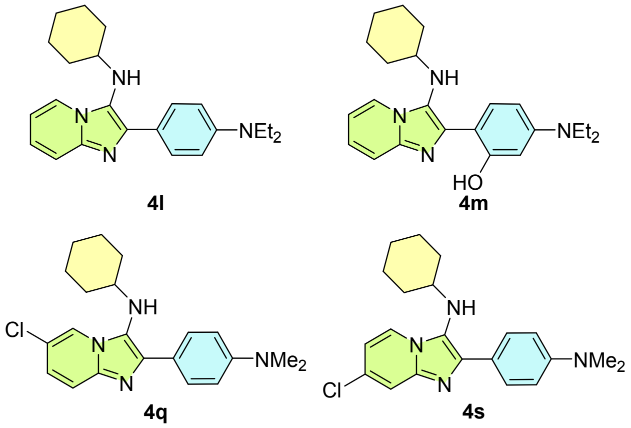

Figure S264. Four analogs chosen for computational calculations.

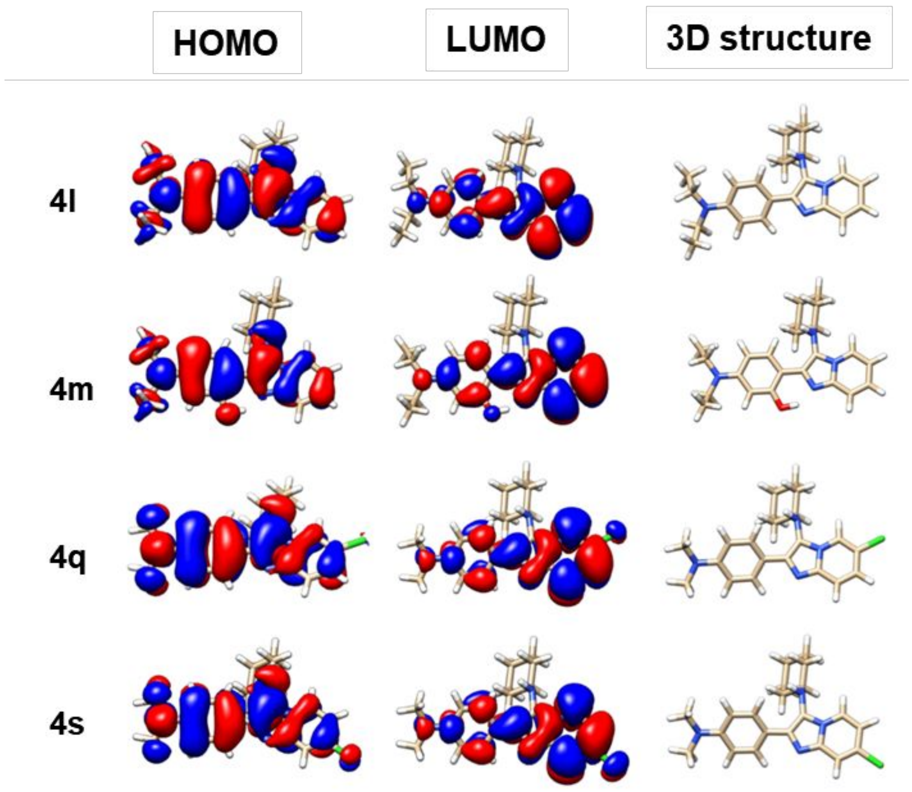

Figure S265. Frontier molecular orbitals (HOMO and LUMO) for molecules **4l**, **4m**, **4q** and **4s**.

References

(1) Ugi. I.; Meyr. R.; Lipinski. M.; Bodesheim. F.; Rosendahl. F. Cyclohexyl isocyanide. *Org. Synth.* **1961**. *41*. 13.

(2) (a) Martinho. L. A.; Andrade. C. K. Z. HPW-Catalyzed environmentally benign approach to imidazo[1.2-*a*]pyridines. *Beilstein J. Org. Chem.* **2024**. *20*. 628–637. (b) Martinho. L. A.; de Lima. D. M.; Praciano. V. H. J. G.; Oliveira. S. C. C.; Andrade. C. K. Z. Phytotoxicity Study of (Amino)Imidazo[1.2-*a*]Pyridine Derivatives Toward the Control of *Bidens Pilosa*. *Urochloa Decumbens*. and *Panicum Maximum* Weeds. *J. Agric. Food Chem.* **2025**. *73* (1). 298–317.

(3) Sheldrick. G. M. A Short History of *SHELX*. *Acta Crystallogr. Sect. A Found. Crystallogr.* **2008**. *64* (1). 112–122.

(4) Sheldrick. G. M. Crystal Structure Refinement with *SHELXL*. *Acta Crystallogr. C Struct. Chem.* **2015**. *71* (1). 3–8.

(5) Dolomanov. O. V.; Bourhis. L. J.; Gildea. R. J.; Howard. J. A. K.; Puschmann. H. *OLEX2*: A Complete Structure Solution. Refinement and Analysis Program. *J. Appl. Crystallogr.* **2009**. *42* (2). 339–341.

(6) Macrae. C. F.; Sovago. I.; Cottrell. S. J.; Galek. P. T. A.; McCabe. P.; Pidcock. E.; Platings. M.; Shields. G. P.; Stevens. J. S.; Towler. M.; Wood. P. A. *Mercury 4.0*: From Visualization to Analysis. Design and Prediction. *J. Appl. Crystallogr.* **2020**. *53* (1). 226–235.

(7) (a) Rhys Williams. A. T.; Winfield. S. A.; Miller. J. N. Relative Fluorescence Quantum Yields Using a Computer-Controlled Luminescence Spectrometer. *Analyst* **1983**. *108* (1290). 1067–1071. (b) Brouwer. A. M. Standards for Photoluminescence Quantum Yield Measurements in Solution (IUPAC Technical Report). *Pure Appl. Chem.* **2011**. pp 2213–2228. (c) Lagorio. M. G. Determination of Fluorescence Quantum Yields in Scattering Media. *Methods Appl. Fluoresc.* **2020**. *8* (4). 043001.

(8) (a) Renno. G.; Cardano. F.; Volpi. G.; Barolo. C.; Viscardi. G.; Fin. A. Imidazo[1.5-*a*]Pyridine-Based Fluorescent Probes: A Photophysical Investigation in Liposome Models. *Molecules* **2022**. *27* (12). 3856. (b) Shinde. V. N.; Kanchan Roy. T.; Jaspal. S.; Nipate. D. S.; Meena. N.; Rangan. K.; Kumar. D.; Kumar. A. Rhodium(III)-Catalyzed Annulation of 2-Arylimidazo[1.2-*a*]Pyridines with Maleimides: Synthesis of 1*H*-Benzo[*e*]Pyrido[1′.2′:1.2]Imidazo[4.5-*g*]Isoindole-1.3(2*H*)-Diones and Their Photophysical Studies. *Adv. Synth. Catal.* **2020**. *362* (24). 5751–5764.

(9) Wildman. S. A.; Crippen. G. M. Prediction of Physicochemical Parameters by Atomic Contributions. *J. Chem. Inf. Comput. Sci.* **1999**. *39* (5). 868–873.

(10) Neese. F. Software update: The ORCA program system—Version 5.0. *Wiley Interdiscip. Rev. Comput. Mol. Sci.* **2022**. *12* (5). e1606.

(11) Yanai. T.; Tew. D. P.; Handy. N. C. A New Hybrid Exchange–Correlation Functional Using the Coulomb-Attenuating Method (CAM-B3LYP). *Chem. Phys. Lett.* **2004**. *393* (1–3). 51–57.

(12) Gregory A. Landrum. RDKit: Open-Source Cheminformatics.

(13) Lu. T.; Chen. F. Multiwfn: A Multifunctional Wavefunction Analyzer. *J. Comput. Chem.* **2012**. *33* (5). 580–592.

(14) (a) Reichardt. C. Solvatochromic Dyes as Solvent Polarity Indicators. *Chem. Rev.* **1994**. *94* (8). 2319–2358. (b) Kamlet. M. J.; Abboud. J. L. M.; Abraham. M. H.; Taft. R. W. Linear Solvation Energy Relationships. 23. A Comprehensive Collection of the Solvatochromic Parameters. .Pi.*. .Alpha.. and .Beta.. and Some Methods for Simplifying the Generalized Solvatochromic Equation. *J. Org. Chem.* **1983**. *48* (17). 2877–2887. (c) Kamlet. M. J.; Abboud. J. L.; Taft. R. W. The Solvatochromic Comparison Method. 6. The .Pi.* Scale of Solvent Polarities. *J. Am. Chem. Soc.* **1977**. *99* (18). 6027–6038. (d) Reichardt. C. *Solvents and Solvent Effects in Organic Chemistry*; Wiley. 2002. (e) Lippert. E. Spektroskopische Bestimmung Des Dipolmomentes Aromatischer Verbindungen Im Ersten Angeregten Singulettzustand. *Ber. Bunsenges. Phys. Chem.* **1957**. *61* (8). 962–975. (f) Mataga. N.; Kaifu. Y.; Koizumi. M. Solvent Effects upon Fluorescence Spectra and the Dipole moments of Excited Molecules. *Bull. Chem. Soc. Jpn.* **1956**. *29* (4). 465–470.
